# Conformationally Programmable Chiral Foldamers with Compact and Extended Domains Controlled by Monomer Structure

**DOI:** 10.1002/anie.201802822

**Published:** 2018-06-12

**Authors:** Zachariah Lockhart, Peter C. Knipe

**Affiliations:** ^1^ School of Chemistry and Chemical Engineering Queen's University Belfast David Keir Building Belfast BT9 5AG UK

**Keywords:** conformational analysis, foldamers, oligomerization, protein–protein interactions

## Abstract

Foldamers are an important class of abiotic macromolecules, with potential therapeutic applications in the disruption of protein–protein interactions. The majority adopt a single conformational motif such as a helix. A class of foldamer is now introduced where the choice of heterocycle within each monomer, coupled with a strong conformation‐determining dipole repulsion effect, allows both helical and extended conformations to be selected. Combining these monomers into hetero‐oligomers enables highly controlled exploration of conformational space and projection of side‐chains along multiple vectors. The foldamers were rapidly constructed via an iterative deprotection‐cross‐coupling sequence, and their solid‐ and solution‐phase conformations were analysed by X‐ray crystallography and NMR and CD spectroscopy. These molecules may find applications in protein surface recognition where the interface does not involve canonical peptide secondary structures.

Nature's oligomers carry out many of the biological functions necessary to sustain life and carry genetic information. The majority of proteins adopt a structure determined entirely by the primary sequence of amino acids,^1^ containing α‐helical, β‐strand/sheet, and loop domains combined in a tertiary fold. A key determinant of protein conformation is the secondary structural propensity (SSP) of the constituent amino acids; that is, the structure of each monomer creates a thermodynamic driving force for it to occupy a particular secondary structural environment (Figure 1 A).^2^

**Figure 1:**
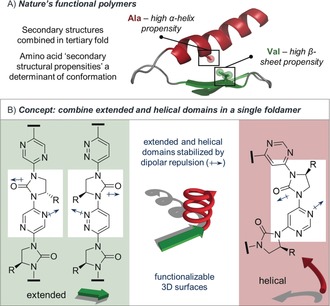
A) Nature's control of tertiary structure with amino acid secondary structural propensities (SSPs) as a determinant. Shown is a truncate of ephrin A2 receptor protein kinase (PDB: 5NKB). Side‐chains A664 and V681 are highlighted. B) Left: aryl‐imidazolidin‐2‐one monomers preferring an extended (linear) conformation due to dipolar repulsion. Selected dipoles are indicated. Right: pyrimidine‐imidazolidin‐2‐one monomers preferring a helical conformation. Centre: representation of multi‐domain foldamer structures.

For decades chemists have sought to mimic the structural and functional diversity of biopolymers using synthetic oligomers, a field now known as foldamer chemistry.^3^ As well as catalytic^4^ and signalling applications,^5^ foldamers have enjoyed success when applied to problems in chemical biology3b such as modulating protein–protein interactions (PPIs).^6^ One approach to controlling global conformation is to exploit non‐covalent interactions between adjacent monomers. Gong^7^ and Huc^8^ have used hydrogen bonding to control curvature in aryl amide foldamers, while Aggarwal^9^ employed the *syn*‐pentane interaction as a controlling force in simple hydrocarbon foldamers.^10^ Lehn pioneered the use of dipolar repulsion as a controlling element in foldamer design, and exploited aromatic heterocycles as shape codons^11^ for helix formation owing to their well‐defined bond angles and strong dipoles.^12^ Dipole repulsion between carbonyl groups has also been explored;^13^ Clayden used this effect as a stereochemical relay to achieve remote (1,23)‐asymmetric induction in an oligoxanthene foldamer.^14^ As a result of these and other approaches, chemists are now able to synthesize foldamers to reliably adopt conformations analogous to protein secondary structures.^15^

Efforts have been made to go beyond forming simple secondary structural motifs. Super‐secondary helical foldamer bundles and β‐helices have been reported,^16^ and recently Horne has used β‐, *N*‐methyl, and other modified amino acids to stabilize a natural zinc finger domain.^17^ Similarly, Kirshenbaum has used the presence of peptoids, in combination with cation–π interactions, to form stable β‐loop‐PPII helix tertiary peptidomimetics.^18^ The task of combining entirely unnatural secondary domains in a single foldamer capable of mimicking tertiary structure (called tyligomers by Moore3c) remains a central challenge, although Huc recently disclosed a remarkable helix–sheet–helix tertiary foldamer.^19^

Inspired by Thompson and Hamilton's use of dipole repulsion to stabilize extended foldamer β‐strand mimetics,15d,15e herein we report the rational design of a range of monomers with different innate folding preferences, analogous to amino acid SSPs.^20^ These allow the selective formation of helical or extended domains within a single foldamer, formed by an iterative cross‐coupling strategy. We theorized that use of three isomeric aromatic linkers (pyrazine, pyridazine, and pyrimidine) would lead to different conformational preferences in aryl‐linked imidazolidine‐2‐one oligomers, as determined by dipolar repulsion between the urea carbonyl groups and adjacent arene nitrogen lone pairs (Figure 1 B). The effect would be that the pyrazine and pyridazine linkers would stabilize extended conformations, while the pyrimidine linker would form curved or helical structures.^11^, ^12^

Our iterative approach to the foldamers required the synthesis of pyrimidine (**3**), pyridazine (**4**), and pyrazine (**5**) monomers, which was achieved from common intermediates **1** and **2** (Scheme 1).^21^ Buchwald–Hartwig coupling of **2** with 4,6‐dichloropyrimidine, and of **1** with 3,6‐dibromopyridazine and 2,5‐dibromopyrazine, afforded monomers **3**, **4**, and **5** in 73 %, 63 %, and 53 % yields, respectively. The conformations of monomers **3**–**5** were examined by single‐crystal X‐ray diffraction.^22^ In all cases the urea carbonyl is oriented *anti* to the *ortho*‐nitrogen atom on the adjacent heterocycle, in line with the dipole repulsion hypothesis.

**Scheme 1:**
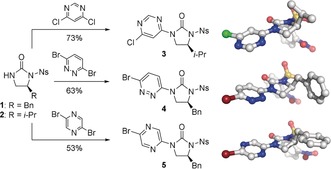
Synthesis of monomers **3**–**5** via Buchwald–Hartwig cross‐coupling. Single‐crystal X‐ray structures^22^ show that all compounds adopt the conformation in which dipoles are opposed. Coupling conditions: aryl dihalide (5 equiv), Pd_2_dba_3_ (5 mol %), Xantphos (15 mol %), Cs_2_CO_3_, PhMe, reflux, 30 min −4 h. Bn=benzyl, dba=dibenzylideneacetone, Ns=2‐nitrobenzenesulfonyl, Xantphos=4,5‐bis(diphenylphosphino)‐9,9‐dimethylxanthene.

The conformational preferences of the corresponding oligomers were then investigated. Ureas **6** and **8** were formed according to literature methods,^21^ and used as the starting point for the iterative synthesis of oligomers (Scheme 2), with the N−Ph acting as a terminal inert capping group and internal standard for subsequent nOe analyses (see below). Deprotection with thiophenol and K_2_CO_3_ liberated ureas **7** and **9**, and cross‐coupling of the former with **3**, and the latter with **4** and **5**, afforded dimeric compounds **10**, **11**, and **12** in 72 %, 99 %, and 91 % yields, respectively. The conformations of all dimers^23^ were examined by single‐crystal X‐ray diffraction (Figure 2).^22^ In all cases the urea C=O bonds are oriented *anti* to the *ortho*‐nitrogen lone pair of the adjacent heteroaromatic ring. For pyrimidine **10** this has the effect that the molecule is highly curved; the monomer induces an 86° turn in the backbone, such that a helix composed of this motif would contain about four residues per turn. For **11** and **12**, the presence of *para*‐substituted pyridazine and pyrazine linkers led to greater linearity, with monomer‐induced curvatures of 154° and 139° respectively. It is likely that longer pyrazine‐containing foldamers would form overall linear conformers due to the alternating disposition of the ureas (Figure 2, cartoon). The control exacted by dipole repulsion also leads to predictable positioning of the side‐chains (Figure 2, right). While the distances between the C_α_ positions are largely unchanged across the three homo‐dimers (8.1–8.7 Å), the facial projection of substituents from the plane of the foldamer is dependent on the heterocyclic linker. Thus, for the pyrimidine and pyridazine foldamers, side‐chains are projected from the same face of the molecule, while the pyrazine linker leads to adjacent side‐chains occupying opposite faces.

**Figure 2:**
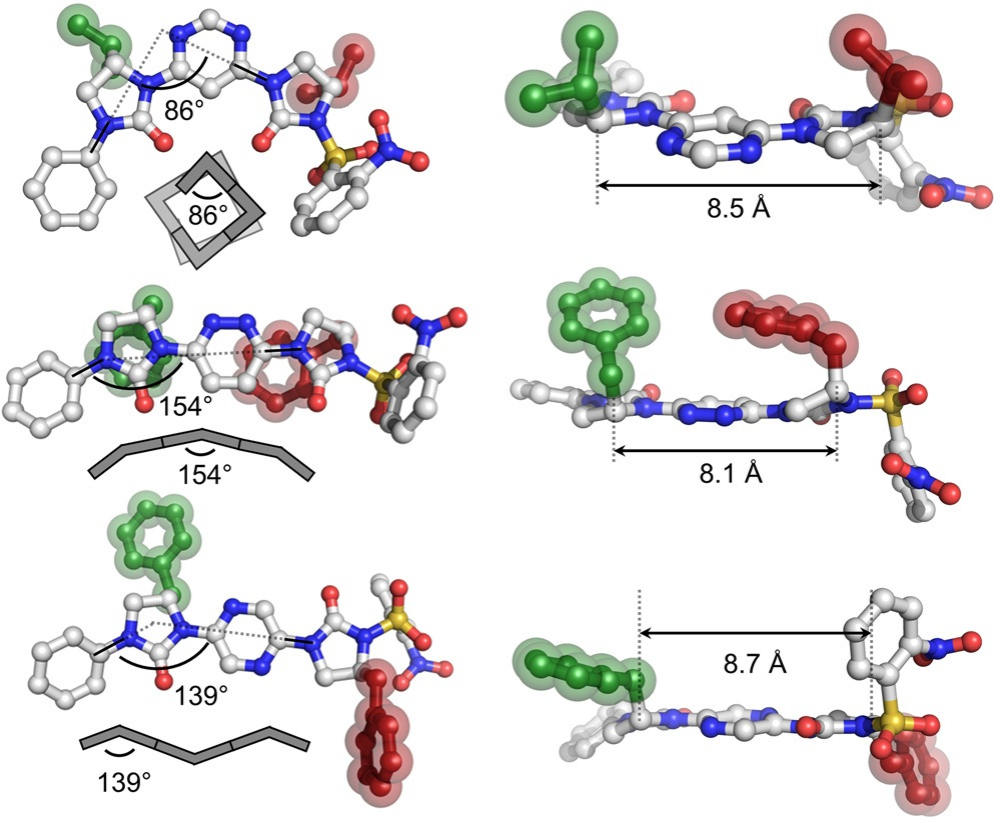
X‐ray crystal structures of dimeric foldamers **10** (top), **11** (middle) and **12** (bottom).^22^ Left: the top elevation gives the deviation (in degrees) each monomer induces in the foldamer, where an angle of 180° would represent perfect linearity. Side‐chains are highlighted in green and red. Cartoon representations of the extrapolated conformation of larger homo‐oligomers are given below each structure. Right: the edge elevation shows the disposition of side‐chain residues relative to the plane of the foldamer, and the distances between the *C*_α_ positions (in Å).

**Scheme 2:**
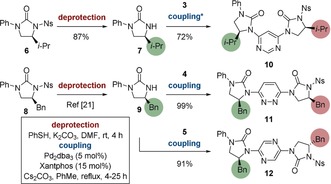
Synthesis of dimers **10** (top), **11** (middle), and **12** (bottom) from terminal monomers **7** and **9**. Imidazolidin‐2‐one side‐chains are highlighted in circles. *After 22 h an additional charge of Pd_2_dba_3_ (5 mol %) and Xantphos (15 mol %) was added. The reaction proceeded to completion after a further 3 h.

The solution‐phase conformational behaviour of the dimers was examined through nuclear Overhauser effect (nOe) correlations. For pyrimidine **10** these indicate a strong preference towards the dipole‐opposed, curved conformation (Figure 3).

**Figure 3:**
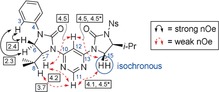
nOe correlations from the ROESY spectrum of **10** (CDCl_3_, 600 MHz, *t*_mix_ 200 ms). Solid black arrows are strong cross‐peaks; dashed red arrows are weak cross‐peaks. Selected atomic numbering given in blue. Numbers (in boxes) given to 1 d.p. indicate measured inter‐proton distances from the X‐ray crystal structure (in Å). *H15 and H15′ are isochronous in the ^1^H spectrum so distances for both diastereotopic hydrogens are given.

The rotating frame nuclear Overhauser effect (ROESY) cross‐peaks of both H^7^ and H^15^ with both H^11^ and H^12^ were of similar intensity, in agreement with the distances observed in the crystal structure. Similarly, the observed H^8^↔H^11^ nOe indicates that the solid‐ and solution‐phase conformations are in agreement, since these hydrogen atoms would be too distant for an observable nOe in the alternative, dipoles‐*syn* conformation. In line with previous analyses,15e, 21 the N−Ph group was used as an internal standard to assess conformation; it is assumed that a freely rotating N−C^13^ bond would lead to an observed nOe intensity ratio between H^12^↔H^15^ and H^3^↔H^6^ of 1:2. The observed ratio was 1:25 in CDCl_3_,^24^ indicating a strong biasing effect in solution. In 100 % [D_6_]DMSO at 298 K the H^12^↔H^15^:H^3^↔H^6^ nOe ratio was 1:41, and an additional H^3^↔H^12^ signal was observed, suggesting the molecule retains its strong preference for the dipole‐opposed conformation in highly polar solvents. When warmed to 355 k the H^12^↔H^15^:H^3^↔H^6^ nOe ratio in [D_6_]DMSO fell to 1:11, suggesting a reduction in (but not loss of) conformational rigidity at elevated temperature. Analogous results (excluding [D_6_]DMSO and variable‐temperature experiments) were obtained for pyridazine and pyrazine dimers **11** and **12** (see the Supporting Information).

Homo‐trimers **13**, **14**, and **15**, and tetramer **16** (Figure 4) were formed by the same iterative deprotection–coupling sequence outlined in Scheme 2. Their ROESY spectra were consistent with these foldamers adopting the conformations predicted by the dipole repulsion hypothesis, with indicative couplings analogous to those in Figure 3 (see also the Supporting Information).

**Figure 4:**
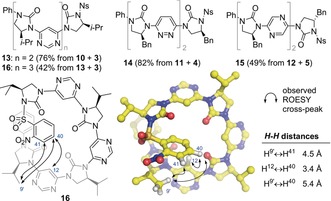
Top: library of homo‐oligomers **13**–**16** synthesized through iterative cross‐coupling. Bottom: arrows indicate observed long‐range nOes of pyrimidine tetramer **16** (left) and its unconstrained lowest energy conformation (middle).^25^ Atom numbers are indicated in blue. The distances given (right) are from the energy‐minimized structure. Where distances are measured to H^9′^, the values given are an average across the three hydrogen atom positions.

Tetramer **16** gave several long‐range nOe correlations (Figure 4), indicating that its ends sit in close proximity, as expected on the basis of the 86° turn per monomer outlined in Figure 2. When compared with the computed low‐energy structure,^25^ these nOes correspond to close contacts in the global minimum, suggesting the (*P*)‐helical conformation is significantly populated in solution. The circular dichroism spectrum of **16** displayed positive and negative Cotton effects at 300 nm and 285 nm respectively that were absent from the spectra of homologous pyrimidine dimer **10** and trimer **13**, and may be diagnostic of (*P*)‐helix formation.^26^

Lastly we examined whether the conformational preferences established above were borne out in hetero‐oligomers. These are expected to form structures containing distinct conformational domains, as determined by the constituent monomers. Trimers **21** and **22** were synthesized by the iterative route detailed in Scheme 2, while pentamer **20** was formed in 50 % yield via a convergent strand‐coupling approach from trimeric fragment **17** and dimeric **19** (Figure 5; for complete synthetic details refer to the Supporting Information).

**Figure 5:**
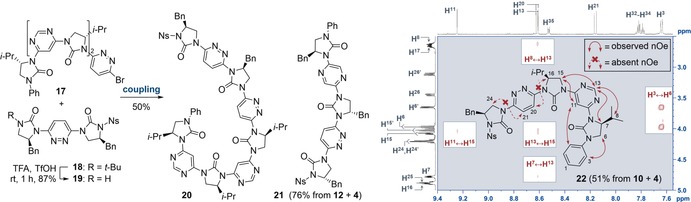
Left: fragment coupling approach to pyrimidine‐pyridazine pentamer **20**. For coupling conditions, see Scheme 2. Middle and right: hetero‐trimers **21** and **22** formed via iterative deprotection‐cross‐coupling. Structures are drawn in the dipole‐opposed conformations. Right: structure and truncated ROESY spectrum of **22** (CDCl_3_, 600 MHz, *t*_mix_ 200 ms). Solid arrows indicate selected, conformationally relevant nOe correlations. Dashed arrows indicate nOe signals which were absent from the spectrum. Regions of the 2D spectrum are highlighted in white to show the relative intensity of peaks.

The solution‐phase conformations of these foldamers were probed by examining their ROESY spectra.

On the basis of the results obtained for the homo‐oligomers, hetero‐trimer **22** (Figure 5, right) was expected to favour a conformation curved at the pyrimidine and linear at the pyridazine linkers along its backbone. This was found to be the case, with the observation of several key nOe correlations, namely H^3^↔H^11^, H^7^↔H^13^, H^8^↔H^13^, and H^13^↔H^15^, indicating a strong preference for the predicted conformations about both N−C_pyrimidine_ bonds (as shown). Similarly, the absence of H^16^↔H^20^ and H^21^↔H^24^ correlations is consistent with the illustrated conformation around both N−C_pyridazine_ bonds. Foldamers **20** and **21** displayed analogous spectral features, indicating that they adopt the dipole‐opposed conformations depicted in Figure 5 (see also the Supporting Information). This confirms that the design strategy described herein allows the construction of bespoke oligomers with predictable, non‐repetitive conformations.

In conclusion, we have developed a chiral amino alcohol‐derived foldamer backbone incorporating pyrimidine, pyridazine, and pyrazine linkers, synthesized primarily through an iterative deprotection‐coupling sequence. These foldamers reliably adopt conformations which can be predicted using a simple dipolar repulsion argument, even in highly polar solvents. As well as giving control of the overall backbone shape, this permits the projection of side‐chains from multiple faces of the foldamer by choice of linker, enabling rapid and programmable exploration of macromolecular conformational space.

## Conflict of interest

The authors declare no conflict of interest.

## Supporting information

As a service to our authors and readers, this journal provides supporting information supplied by the authors. Such materials are peer reviewed and may be re‐organized for online delivery, but are not copy‐edited or typeset. Technical support issues arising from supporting information (other than missing files) should be addressed to the authors.

Supplementary
