# Supplementary material for: Conformationally Programmable Chiral Foldamers with Compact and Extended Domains Controlled by Monomer Structure
Source: Angew Chem Int Ed Engl. 2018 Jun 12;57(28):8478–82. doi: 10.1002/anie.201802822 (PMC6055681; doi:10.1002/anie.201802822)
Supplement: Supplementary file 1 — Supplementary [file ANIE-57-8478-s001.pdf]

Angewandte Chemie
Eine Zeitschrift der Gesellschaft Deutscher Chemiker

Supporting Information

Conformationally Programmable Chiral Foldamers with Compact and Extended Domains Controlled by Monomer Structure

*Zachariah Lockhart and Peter C. Knipe**

anie_201802822_sm_miscellaneous_information.pdf

Supplementary Information

Contents

General Experimental 4

Naming and Numbering of Compounds 4

Solvents and Reagents 4

Chromatography 4

Spectroscopy 4

Crystallography 5

Melting Points 5

Experimental Procedures and Characterization Data 6

Solution-Phase Conformational Analysis by NMR 24

Pyrimidine Dimer 10 25

Pyridazine Dimer 11 33

Pyrazine Dimer 12 35

Pyrimidine Trimer 13 38

Pyridazine Trimer 14 41

Pyrazine Trimer 15 45

Pyrimidine Tetramer 16 48

Mixed Pentamer 20 52

Pyridazine-Pyrazine Trimer 21 55

Pyrimidine-Pyrazine Trimer 22 58

X-Ray Crystallography 61

Pyrimidine Monomer 3 61

Crystal Data 61

Data Collection 61

Refinement 62

Pyridazine Monomer 4 63

Crystal Data......63
Data Collection......63
Refinement......63
Pyrazine Monomer 5......65
Crystal Data......65
Data Collection......65
Refinement......65
Pyrimidine Dimer 10......67
Crystal Data......67
Data Collection......67
Refinement......67
Pyridazine Dimer 11......69
Crystal Data......69
Data Collection......69
Refinement......69
Pyrazine Dimer 12......71
Crystal Data......71
Data Collection......71
Refinement......71
Circular Dichroism......73
Homo-Pyrimidine Series......73
Homo-Pyridazine Series......74
Homo-Pyrazine Series......75
Mixed Pyridazine-Pyrazine Trimer 21......76
Mixed Pyrimidine-Pyridazine Trimer 22......77
Mixed Pyrimidine-Pyridazine Pentamer 20......78
Computation......79
Tetramer 16......79
Atomic Coordinates of (*P*)-helical energy minimum of 16......80

MOPAC 2016 Output for (*P*)-helical energy minimum of 16 81

Atomic Coordinates of lowest energy (*M*)-helical conformer of 16 82

MOPAC 2016 Output for lowest energy (*M*)-helical conformer of 16 83

References 84

NMR Spectra 85

General Experimental

Naming and Numbering of Compounds

Systematic compound names are those generated by ChemBioDraw™ Ultra version 15.1.0.144 (Perkin Elmer) following IUPAC nomenclature.

Solvents and Reagents

Reactions were carried out under a nitrogen atmosphere in oven-dried glassware unless otherwise stated. Standard inert atmosphere techniques were used in handling all air- and moisture-sensitive reagents. Where necessary, toluene and *N*,*N*-DMF (from commercial sources) were de-gassed prior to use by sparging with argon or nitrogen for at least 15 min. Other solvents and reagents were used directly as received from commercial suppliers.

Chromatography

Flash column chromatography was carried out using Fluorochem 60 40-63 micron silica gel. Thin-layer chromatography was carried out using Merck Kieselgel 60 F254 (230-400 mesh) fluorescent treated silica, visualized under UV light (254 nm) or by staining with ninhydrin, ceric ammonium molybdate or aqueous potassium permanganate solutions.

Spectroscopy

^{1}H and ^{13}C NMR spectra were recorded using a Bruker 600, 400 or 300 MHz spectrometer running TopSpin™ software and are quoted in parts per million (ppm) for measurement against tetramethylsilane. Where no tetramethylsilane was present, spectra are referenced relative to the residual non-deuterated solvent peaks. Unless otherwise stated spectra were acquired at 298 K. Topspin™ was used for processing and viewing NMR data. Chemical shifts (δ) are given in ppm, and coupling constants (*J*) are given in Hertz (Hz). The ^{1}H NMR spectra are reported as follows: δ / ppm (number of protons, multiplicity, coupling constant *J* / Hz (where appropriate), assignment). Multiplicity is abbreviated as follows: s = singlet, br = broad, d = doublet, t = triplet, q = quartet, m = multiplet. Where peaks are not fully resolved but 2D experiments unambiguously identify the regions of multiplet corresponding to particular hydrogens, this is indicated with a subscript denoting the portion of the multiplet. For example, $H15_{RHS}$ would indicate that the right-hand side of the multiplet corresponds to H15. The numbering scheme used for NMR assignment is arbitrary and does not follow any particular convention. The ^{13}C NMR spectra are reported in δ / ppm. Where necessary or appropriate, two-dimensional (COSY, HSQC, HMBC, NOESY or ROESY) NMR experiments were used to assist the assignment of signals in the ^{1}H and ^{13}C NMR spectra. In some cases, complete assignment of spectra was not possible (in particular, where aromatic CHs corresponding to multiple phenyl groups overlapped significantly); in these cases only a partial assignment is reported.

Infra-red (IR) spectra were recorded on a Perkin-Elmer Spectrum 100 FT-IR spectrometer equipped with a Perkin-Elmer Universal ATR Sampling Accessory, or an Agilent Cary 630 spectrometer equipped with a DialPath accessory. Samples were deposited on the ATR and DialPath accessories as a thin film. Only selected maximum absorbances (v_{max}) of the most intense peaks are reported (cm^{-1}).

High resolution mass spectra (HRMS) were recorded by Analytical Services and Environmental Projects (ASEP) at Queen's University Belfast on a Waters LCT Premier ToF mass spectrometer using the electrospray ionisation (ESI) technique.

Optical rotations were recorded at the sodium D-line (589 nm) using a Perkin Elmer 341 polarimeter at a temperature of 20 °C and are reported in degrees using concentrations (*c*) in g·100 mL^{-1}. Reported values are the average of eight readings.

Circular dichroism (CD) spectra were acquired at 293 K on a Jasco J-815 CD Spectrometer. Samples were placed in a quartz cuvette with a path length of 1 mm.

Crystallography

Low temperature[1] single crystal X-ray diffraction studies were carried out using CuK$_\alpha$ radiation on an Agilent Supernova diffractometer equipped with an area detector and graphite monochromator. Raw frame data were reduced using CrysAlisPRO[2] solved using Superflip.[3] Full-matrix least-squares refinement of the structures were carried out using CRYSTALS.[4,5] Full refinement details are given in the supplementary material (CIF). CCDC 1824641 (**3**), 1824637 (**4**), 1824639 (**5**), 1824642 (**10**), 1824638 (**11**), 1825640 (**12**) contain the supplementary crystallographic data for this paper. These data are provided free of charge by The Cambridge Crystallographic Data Centre and copies can be obtained free of charge *via* www.ccdc.cam.ac.uk/data_request/cif.

Melting Points

Melting points were determined for compounds where a preparative recrystallization was carried out. These were acquired on a Stuart SMP10 digital melting point apparatus. Values are given in °C and are uncorrected.

Experimental Procedures and Characterization Data

Compounds **1**, **6** and **8** were prepared according to previously reported procedures.[6]

General Procedure A (*N*-Nosyl Deprotection)

To a stirred, room-temperature suspension of *N*-nosyl urea (1.0 eq.) and K_2CO_3 (3.0 eq.) in *N*,*N*-DMF (*ca.* 0.1 M) was added thiophenol (1.5 eq.). The rapid development of a deep orange colour was invariably observed upon the addition of thiophenol; the reaction mixture also turned cloudy over time in some cases. After complete consumption of the *N*-nosyl-protected starting material by TLC analysis, the reaction mixture was concentrated *in vacuo* at 60 °C, and the crude residue was taken up in dichloromethane (*ca.* 20 mL/mmol urea). The solution was washed with $NaHCO_3$ (sat. aq., *ca*. 10 mL/mmol urea), and the aqueous layer was extracted with dichloromethane (*ca*. 2 x 10 mL/mmol urea). The combined organic extracts were dried over anhydrous magnesium sulfate, filtered, and concentrated *in vacuo*. The crude product was then purified by flash column chromatography.

General Procedure B (Palladium-Catalysed Coupling of Deprotected Ureas with Aryl Halides)

To a Schlenk tube equipped with a magnetic stir bar was added deprotected urea (1.0 eq.), aryl halide (1.1-5.0 eq.), $Pd_2(dba)_3$ (5-10 mol%), and Xantphos (15-30 mol%). Toluene (*ca.* 0.1 M) was added to the flask, and the resulting suspension was de-gassed by sparging with nitrogen gas for 15-30 min. Cs_2CO_3 (2.5 eq.) was then added in one portion to the flask, and the reaction mixture was heated to reflux under a nitrogen atmosphere. After complete consumption of the urea starting material by TLC analysis, the reaction was cooled to room temperature, and diluted with dichloromethane (*ca*. 20 mL/mmol deprotected urea). The solution was washed with water (*ca*. 10 mL/mmol deprotected urea), and the aqueous layer was extracted with dichloromethane (*ca*. 2 x 10 mL/mmol deprotected urea). The combined organic extracts were dried over anhydrous magnesium sulfate, filtered, and concentrated *in vacuo*. The crude product was then purified by flash column chromatography.

(*S*)-2-Isopropyl-1-((2-nitrophenyl)sulfonyl)aziridine (S1)

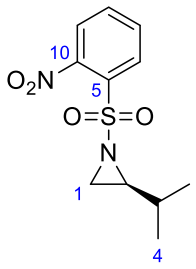

2-Nitrobenzenesulfonyl chloride (16.15 g, 72.9 mmol) was added in five portions to a stirred 0 °C solution of L-valinol (3.00 g, 29.1 mmol) and pyridine (12 mL) in dichloromethane (30 mL). The reaction was allowed to warm to room temperature. After 24 h, the volatiles were removed *in vacuo*. The concentrated reaction mixture was then taken up in diethyl ether (80 mL), and the organic layer was washed with HCl (1 M aq.) until the aqueous washings were acidic (approximately 6 x 40 mL). KOH (2 M aq., 160 mL) was added to the organic layer, and the resulting biphasic mixture was stirred vigorously for 6 h. The layers were separated, and the organic layer washed with KOH (2 M aq., 2 x 40 mL). The organic layer was dried over anhydrous magnesium sulfate, filtered, and concentrated *in vacuo*. The crude residue was passed over a plug of silica (eluent: dichloromethane) and concentrated *in vacuo* to afford **S1** (4.93 g, 63%) as a pale yellow oil.* δ_H (400 MHz, $CDCl_3$): 8.22-8.16 (1H, m, H9), 7.80-7.68 (3H, m, H6, H7 & H8), 2.86-2.79 (2H, m, H1 & H2), 2.36-2.29 (1H, m, H1'), 1.64-1.51 (1H, m, H3), 0.96 (3H, d, *J* 3.8, H4), 0.94 (3H, d, *J* 3.7, H4'); δ_C (75 MHz, $CDCl_3$): 148.6 (C10), 134.6 (Ar CH [nosyl]), 132.0 (Ar CH [nosyl]), 131.5 (C5), 131.1 (C9), 124.2 (Ar CH [nosyl]), 47.1 (C2), 35.0

* It has been previously reported that *N*-nosyl-protected aziridines are prone to polymerisation upon standing.[6] Aziridine **S1**, however, was indefinitely stable when stored as a dilute solution in EtOAc.

(C1), 30.0 (C3), 19.4 & 18.8 (C4 & C4’); HRMS (ESI+): found 293.0580; $C_{11}H_{14}N_2NaO_4S$, $[M+Na]^+$ requires 293.0572; v_{max} (thin film): 1542, 1331, 1163, 751, 605, 596 cm^{-1}; $[\alpha]_D^{20}$ +85.6 (c = 1.17, $CHCl_3$).

(*S*)-*N*-(3-Methyl-1-(phenylamino)butan-2-yl)-2-nitrobenzenesulfonamide (S2)

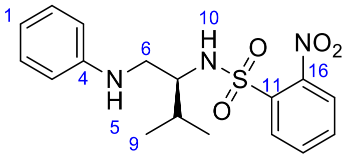

Aziridine **S1** (1.62 g, 5.99 mmol) was dissolved in aniline (6 mL). The resulting solution was stirred at room temperature for 18 h, and then concentrated *in vacuo* at 60 °C. The crude residue was purified by flash column chromatography (silica gel, dichloromethane:petrol:ethyl acetate, 7:2:1) then heated to 60 °C under high vacuum to remove residual aniline, affording diamine **S2** (2.13 g, 98%) as an orange-red solid. δ_H (400 MHz, $CDCl_3$): 8.00 (1H, dd, J 7.5, 1.7, H15), 7.79 (1H, dd, J 7.7, 1.6, H12), 7.64-7.53 (2H, m, $H13_{LHS}$ & $H14_{RHS}$), 7.10-7.02 (2H, m, H2), 6.66 (1H, tt, J 7.3, 1.0, H1), 6.36-6.31 (2H, m, H3), 5.38 (1H, d, J 8.3, H10), 3.80 (1H, br s, H5), 3.59-3.50 (1H, m, H7), 3.28 (1H, dd, J 13.6, 4.5, H6), 3.11 (1H, dd, J 13.6, 8.7, H6’), 2.01-1.88 (1H, m, H8), 0.97 (3H, d, J 6.9, H9), 0.91 (3H, d, J 6.8, H9’); δ_C (101 MHz, $CDCl_3$): 147.6 & 147.3 (C4 & C16), 135.0 (C11), 133.3 & 133.0 (C13 & C14), 130.6 (C15), 129.3 (C2), 125.3 (C12), 117.8 (C1), 112.7 (C3), 59.7 (C7), 46.0 (C6), 30.8 (C8), 19.1 (C9’), 17.8 (C9); HRMS (ESI+): found 364.1328; $C_{17}H_{22}N_3O_4S$, $[M+H]^+$ requires 364.1331; v_{max} (thin film): 3400, 3387, 1530, 1517, 1359, 1340, 1166; $[\alpha]_D^{20}$ -37.2 (c = 1.02, $CHCl_3$).

(*S*)-*N*-(1-Amino-3-methylbutan-2-yl)-2-nitrobenzenesulfonamide (S3)

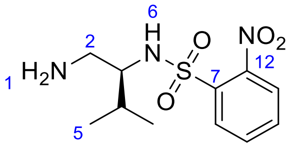

NH_3 (38% aq., 20 mL) was added to a stirred solution of aziridine **S1** (2.61 g, 9.66 mmol) in methanol (20 mL) at room temperature. After 2 h, the volatiles were removed *in vacuo*, and the product was extracted from the resulting aqueous mixture with dichloromethane (3 x 50 mL). The combined organic extracts were dried over anhydrous magnesium sulfate, filtered, and concentrated *in vacuo*. The crude residue was purified by flash column chromatography (silica gel, dichloromethane:methanol:triethylamine, 95:5:1) to afford diamine **S3** (1.98 g, 71%) as a yellow solid. δ_H (400 MHz, $CDCl_3$): 8.16-8.10 (1H, m, H11), 7.88-7.82 (1H, m, H8), 7.76-7.67 (2H, m, H9 & H10), 3.19 (1H, app. td, J 6.2, 4.8, H3), 2.74 (1H, dd, J 13.4, 6.4, H2), 2.68 (1H, dd, J 13.4, 4.8, H2’), 1.89-1.76 (1H, m, H4), 0.87 (3H, d, J 6.8, H5), 0.82 (3H, d, J 6.8, H5’); δ_C (101 MHz, $CDCl_3$): 147.9 (C12), 135.3 (C7), 133.4 & 132.9 (C9 & C10), 130.6 (C11), 125.4 (C8), 63.1 (C3), 43.4 (C2), 30.2 (C4), 19.3 (C5’), 18.4 (C5); HRMS (ESI+): found 288.1030; $C_{11}H_{18}N_3O_4S$, $[M+H]^+$ requires 288.1018; v_{max} (thin film): 2695 (br), 1549, 1533, 1368, 1324, 1155.cm^{-1}; $[\alpha]_D^{20}$ +27.5 (c = 1.05, $CHCl_3$).

(*S*)-5-Isopropyl-1-((2-nitrophenyl)sulfonyl)imidazolidin-2-one (2)

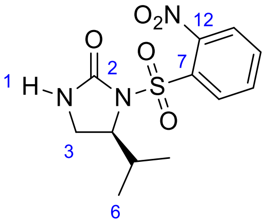

A solution of triphosgene (803 mg, 2.71 mmol) in acetonitrile (20 mL) was added over 1 h *via* syringe pump to a stirred, room-temperature solution of diamine **S3** (1.90 g, 6.61 mmol) and Hünig’s base (3.50 mL, 20.1 mmol) in acetonitrile (40 mL). After 1 h, an additional portion of Hünig’s base (0.88 mL, 0.51 mmol) was charged to the reaction mixture, followed by triphosgene (200 mg, 0.674 mmol) in acetonitrile (6 mL). After a further 30 min, the reaction mixture was

concentrated *in vacuo*. The crude residue was taken up in dichloromethane (50 mL), and washed with HCl (1 N aq., 50 mL). The layers were separated, and the aqueous phase was extracted with dichloromethane (3 x 25 mL). The combined organic extracts were dried over anhydrous magnesium sulfate, filtered, and concentrated *in vacuo*. Flash column chromatography (silica gel, dichloromethane:ethyl acetate, 4:1) followed by trituration with diethyl ether afforded urea **2** (1.89 g, 91%) as an off-white solid. δ_H (400 MHz, d_6-DMSO): 8.21 (1H, dd, *J* 7.7, 1.6, H11), 8.04 (1H, dd, *J* 7.8, 1.5, H8), 7.96 (1H, td, *J* 7.6, 1.6, H9), 7.91 (1H, td, *J* 7.6, 1.6, H10), 7.72 (1H, br s, H1), 4.21 (1H, ddd, *J* 9.3, 4.0, 2.8, H4), 3.49 (1H, t, *J* 9.6, H3), 3.23 (1H, dd, *J* 9.9, 2.6, H3'), 2.19-2.07 (1H, m, H5), 0.93 (3H, d, *J* 4.6, H6), 0.91 (3H, d, *J* 4.7, H6'); δ_C (101 MHz, d_6-DMSO): 154.1 (C2), 147.2 (C12), 135.5 (Ar CH [nosyl]), 133.0 (Ar CH [nosyl]), 132.2 (Ar CH [nosyl]), 130.9 (C7), 124.4 (Ar CH [nosyl]), 61.6 (C4), 38.6 (C3), 32.0 (C5), 17.3 (C6'), 14.9 (C6); HRMS (ESI+): found 314.0819; $C_{12}H_{16}N_3O_5S$, $[M+H]^+$ requires 314.0811; ν_{max} (thin film): 3368, 1753, 1709, 1553, 1538, 1164, 1125; $[\alpha]_D^{20}$ +472.4 (*c* = 0.82, EtOAc).

(*S*)-1-(6-Chloropyrimidin-4-yl)-4-isopropyl-3-((2-nitrophenyl)sulfonyl)imidazolidin-2-one (3)

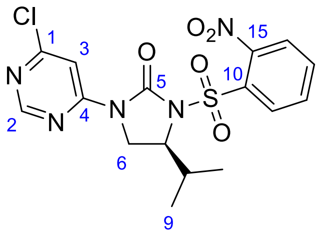

Prepared according to **General Procedure B** using urea **2** (1.36 g, 4.34 mmol), 4,6-dichloropyrimidine (3.22 g, 21.6 mmol), $Pd_2(dba)_3$ (200 mg, 0.218 mmol), Xantphos (379 mg, 0.655 mmol), toluene (40 mL), and Cs_2CO_3 (3.53 g, 10.8 mmol). Reaction time = 4 h. Flash column chromatography (silica gel, petrol:diethyl ether, 3:2) followed by recrystallisation from hot isopropanol afforded chloropyrimidine **3** (1.34 g, 73%) as a white solid. Diffraction-quality crystals were grown by the vapour diffusion method (chloroform/petrol). δ_H (600 MHz, $CDCl_3$): 8.67 (1H, d, *J* 1.1, H2), 8.50-8.46 (1H, m, H14), 8.08 (1H, d, *J* 1.1, H3), 7.85-7.76 (3H, m, H11, H12 & H13), 4.49 (1H, ddd, *J* 8.6, 4.0, 2.8, H7), 4.14-4.06 (2H, m, H6), 2.45-2.37 (1H, m, H8), 1.07 (3H, d, *J* 7.0, H9), 1.02 (3H, d, *J* 6.9, H9'); δ_C (151 MHz, $CDCl_3$): 161.6 (C1), 158.1 (C2), 157.6 (C4), 151.2 (C5), 148.2 (C15), 135.4 (C11/C12/C13), 135.3 (C14), 132.3 (C11/C12/C13), 131.4 (C10), 124.8 (C11/C12/C13), 109.4 (C3), 59.8 (C7), 43.2 (C6), 32.6 (C8), 18.0 (C9), 15.1 (C9'); HRMS (ESI+): found 426.0748; $C_{16}H_{17}ClN_5O_5S$, $[M+H]^+$ requires 426.0639; ν_{max} (thin film): 1738, 1538, 1360, 1173, 1113, 708 cm^{-1}; $[\alpha]_D^{20}$ +194.8 (c = 1.20, $CHCl_3$); MP: 155-157 °C (*i*-PrOH).

(*S*)-2-Benzyl-1-((2-nitrophenyl)sulfonyl)aziridine (S4)

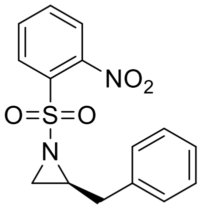

2-Nitrobenzenesulfonyl chloride (74.4 g, 336 mmol) was added in five portions to a stirred, room-temperature solution of L-phenylalaninol (20.00 g, 132.2 mmol) and pyridine (80 mL) in dichloromethane (200 mL). After 16 h, the reaction mixture was diluted with diethyl ether (400 mL), and the organic layer was washed with HCl (1 M aq.) until the aqueous washings were acidic (approximately 6 x 100 mL). KOH (2 M aq., 150 mL) was added to the organic layer, and the resulting biphasic mixture was stirred vigorously for 6 h. The layers were separated, and the organic layer was washed successively with KOH (2 M aq., 2 x 100 mL) and brine (100 mL). The organic layer was dried over anhydrous magnesium sulfate, filtered and concentrated *in vacuo*. The solid residue was passed over a plug of silica (eluent: dichloromethane) and concentrated *in vacuo*. The crude solid product was recrystallized from hot toluene (~120 mL) to

afford aziridine **S4** (20.8 g, 50%) as a pale yellow crystalline solid.* δ_H (400 MHz, $CDCl_3$): 8.07 (1H, dd, *J* 7.9, 1.4), 7.78-7.61 (3H, m), 7.26-7.13 (5H, m), 3.29-3.21 (1H, m), 2.98 (1H, dd, *J* 14.4, 6.1), 2.93 (1H, d, *J* 6.8), 2.83 (1H, dd, *J* 14.6, 6.0), 2.37 (1H, d, *J* 4.8); δ_C (101 MHz, $CDCl_3$): 136.5. 134.4, 132.0, 131.8, 131.1, 128.9, 128.5, 126.9, 124.3, 42.8, 37.4, 35.0; HRMS (ESI+): found 341.0570; $C_{15}H_{14}N_2O_4NaS$, $[M+Na]^+$ requires 341.0572; $[\alpha]_D^{20}$ +40.3 (*c* = 1.13, $CHCl_3$); ν_{max} (thin film): 3092, 1545, 1336, 1169, 751 cm^{-1}; MP: 93-95 °C (PhMe).

(*S*)-4-Benzyl-1-(6-bromopyridazin-3-yl)-3-((2-nitrophenyl)sulfonyl)imidazolidin-2-one (4)

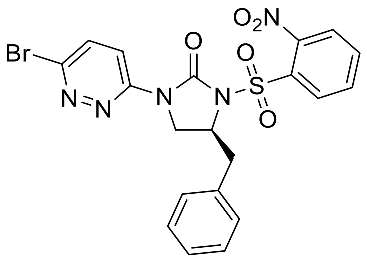

Prepared twice on different scales according to **General Procedure B**. Using urea **1** (100 mg, 0.277 mmol, 1.0 eq.), 3,6-dibromopyridazine (328 mg, 1.38 mmol, 5.0 eq.), $Pd_2(dba)_3$ (26 mg, 0.028 mmol, 10 mol%), Xantphos (48 mg, 0.083 mmol, 30 mol%), toluene (3 mL) and Cs_2CO_3 (225 mg, 0.691 mmol, 2.5 eq.) afforded bromopyridazine **4** (100 mg, 70%) as a white solid after purification by flash column chromatography (silica gel, petrol:ethyl acetate, 3:1). Using urea **1** (349 mg, 0.966 mmol, 1.0 eq.), 3,6-dibromopyridazine (573 mg, 2.41 mmol, 2.5 eq.), $Pd_2(dba)_3$ (44 mg, 0.048 mmol, 5 mol%), Xantphos (85 mg, 0.15 mmol, 15 mol%), toluene (10 mL) and Cs_2CO_3 (787 mg, 2.42 mmol, 2.5 eq.) afforded bromopyridazine **4** (276 mg, 55%) as a yellow foam solid after purification by flash column chromatography (silica gel, petrol:ethyl acetate, 7:3). Average yield = 63% (*n* = 2). Diffraction-quality crystals were grown by the vapour diffusion method (chloroform/petrol). δ_H (600 MHz, $CDCl_3$): 8.52-8.47 (1H, m), 8.00 (1H, d, *J* 9.4), 7.84-7.77 (3H, m), 7.45 (1H, d, *J* 9.4), 7.35-7.32 (2H, app. d, *J* 7.0), 7.29 (2H, app. t, *J* 7.6), 7.21 (1H, tt, *J* 7.1, 1.4), 4.91-4.86 (1H, m), 4.24-4.19 (2H, m), 3.36 (1H, dd, *J* 13.7, 3.4), 3.14 (1H, dd, *J* 13.7, 8.1); δ_C (151 MHz, $CDCl_3$): 153.1, 150.9. 148.0, 143.0, 135.3, 134.7, 134.4, 132.2, 132.1, 131.3, 129.7, 128.9, 127.5, 124.7, 119.7, 55.8, 46.3, 41.5; HRMS (ESI+): found 518.0115; $C_{20}H_{17}BrN_5O_5S$, $[M(^{79}Br)+H]^+$ requires 518.0134; ν_{max} (thin film): 1733, 1539, 1418, 1228, 1172, 1109 cm^{-1}; $[\alpha]_D^{20}$ +196.8 (*c* = 0.70, $CHCl_3$).

(*S*)-4-Benzyl-1-(5-bromopyrazin-2-yl)-3-((2-nitrophenyl)sulfonyl)imidazolidin-2-one (5)

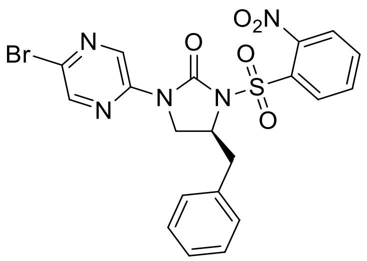

Prepared twice on different scales according to **General Procedure B**. Using urea **1** (100 mg, 0.277 mmol, 1.0 eq.), 2,5-dibromopyrazine (328 mg, 1.38 mmol, 5.0 eq.), $Pd_2(dba)_3$ (26 mg, 0.028 mmol, 10 mol%), Xantphos (48 mg, 0.083 mmol, 30 mol%), toluene (3 mL) and Cs_2CO_3 (225 mg, 0.691 mmol, 2.5 eq.) afforded bromopyrazine **5** (95 mg, 66%) as a white solid after purification by flash column chromatography (silica gel, petrol:ethyl acetate, 4:1). Using urea **1** (250 mg, 0.692 mmol, 1.0 eq.), 3,6-dibromopyridazine (541 mg, 2.27 mmol, 3.3 eq.), $Pd_2(dba)_3$ (32 mg, 0.035 mmol, 5 mol%), Xantphos (60 mg, 0.10 mmol, 15 mol%), toluene (7 mL) and Cs_2CO_3 (564 mg, 1.73 mmol, 2.5 eq.) afforded

* It has previously been reported that this molecule, prepared by an alternative route, undergoes spontaneous polymerization on storage.[6] In our hands this was the case if it is stored as the amorphous glassy solid obtained directly from flash column chromatography. However, we have found the recrystallized compound to be entirely bench stable and no longer prone to decomposition.

bromopyrazine **5** (142 mg, 40%) as a white solid after purification by flash column chromatography (silica gel, petrol:ethyl acetate, 9:1→4:1) and trituration with diethyl ether. Average yield = 53% (*n* = 2). Diffraction-quality crystals were grown by the vapour diffusion method (chloroform/petrol). δ_H (400 MHz, $CDCl_3$): 8.96 (1H, d, *J* 1.5), 8.56-8.50 (1H, m), 8.27 (1H, d, *J* 1.3), 7.86-7.76 (3H, m), 7.36-7.28 (4H, m), 7.26-7.20 (1H, m), 4.87 (1H, tdd, *J* 8.3, 3.5, 1.8), 4.05 (1H, dd, *J* 11.0, 8.5), 3.95 (1H, dd, *J* 11.0, 1.8), 3.38 (1H, dd, *J* 13.7, 3.4), 3.14 (1H, dd, *J* 13.8, 8.3); δ_C (101 MHz, $CDCl_3$) 150.4, 148.0, 146.4, 144.0, 136.0, 135.3, 134.9, 133.3, 132.2, 131.4, 129.6, 128.9, 127.5, 124.6, 55.9, 45.6, 41.6; HRMS (ESI+): found 518.0116; $C_{20}H_{17}BrN_5O_5S$, $[M(^{79}Br)+H]^+$ requires 518.0134; ν_{max} (thin film): 1736, 1535, 1443, 1358, 1167, 1108 cm^{-1}; $[\alpha]_D^{20}$ +162.7 (*c* = 1.35, $CHCl_3$).

(*S*)-4-Isopropyl-3-((2-nitrophenyl)sulfonyl)-1-phenylimidazolidin-2-one (6)

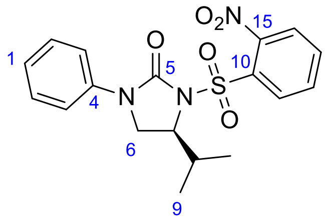

A solution of triphosgene (685 mg, 2.31 mmol) in acetonitrile (20 mL) was added over 1 h *via* syringe pump to a stirred solution of diamine **S2** (2.09 g, 5.75 mmol) and Hünig's base (3.0 mL, 17 mmol) in acetonitrile (40 mL) at 0 °C. The solution was then allowed to warm to room temperature. After 3 h, an additional portion of triphosgene (169 mg, 0.570 mmol) in acetonitrile (5 mL) was charged to the reaction mixture. After a further 30 min,* the reaction mixture was concentrated *in vacuo*. The crude residue was taken up in dichloromethane (50 mL), and washed with HCl (1 N aq., 50 mL). The layers were separated, and the aqueous phase was extracted with dichloromethane (3 x 25 mL). The combined organic extracts were dried over anhydrous magnesium sulfate, filtered, and concentrated *in vacuo*. The crude residue was purified by flash column chromatography (silica gel, petrol:ethyl acetate, 3:1) to afford urea **6** (1.72 g, 77%) as a cream-coloured solid. δ_H (400 MHz, $CDCl_3$): 8.51-8.45 (1H, m, H14), 7.79-7.70 (3H, m, H11, H12 & H13), 7.45-7.39 (2H, m, H3), 7.36-7.29 (2H, m, H2), 7.11 (1H, tt, *J* 7.3, 1.1, H1), 4.45 (1H, ddd, *J* 9.1, 4.2, 2.0, H7), 4.21 (1H, t, *J* 9.2, H6), 3.59 (1H, dd, *J* 9.4, 2.0, H6'), 2.46-2.33 (1H, m, H8), 1.06 (6H, app. d, *J* 7.0, H9 & H9'); δ_C (101 MHz, $CDCl_3$): 151.4 (C5), 148.1 (C15), 138.2 (C4), 135.3 (C14), 134.8 (C11/C12/C13), 132.1(7) (C10), 132.1(2) (C11/C12/C13), 129.2 (C2), 124.7 (C1), 124.4 (C11/C12/C13), 118.9 (C3), 59.3 (C7), 45.1 (C6), 32.8 (C8), 18.0 & 15.4 (C9 & C9'); HRMS (ESI+): found 390.1108; $C_{18}H_{20}N_3O_5S$, $[M+H]^+$ requires 390.1124.; ν_{max} (thin film): 1718, 1541, 1364, 1350, 1165, 1125; $[\alpha]_D^{20}$ +347.3 (*c* = 1.05, $CHCl_3$).

(*S*)-4-Isopropyl-1-phenylimidazolidin-2-one (7)

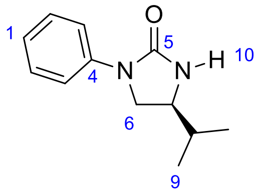

Prepared according to **General Procedure A** using *N*-nosyl urea **6** (1.71 g, 4.39 mmol), K_2CO_3 (1.82 g, 13.2 mmol), *N*,*N*-DMF (40 mL), and thiophenol (0.68 mL, 6.6 mmol). Reaction time = 16 h. Purification by flash column chromatography (silica gel, dichloromethane:ethyl acetate, 17:3) afforded the deprotected urea **7** (845 mg, 94%) as a white solid. δ_H (400 MHz, $CDCl_3$): 7.57-7.52 (2H, m, H3), 7.37-7.30 (2H, m, H2), 7.05 (1H, tt, *J* 7.4, 1.1, H1), 4.97 (1H, br s, H10), 3.95 (1H, t, *J* 8.7, H6), 3.63-3.49 (2H, m, $H6'_{LHS}$ & $H7_{RHS}$), 1.83-1.70 (1H, m, H8), 0.99 (3H, d, *J* 6.7, H9), 0.96 (3H, d, *J* 6.8, H9'); δ_C (101 MHz, $CDCl_3$): 159.2 (C5), 140.2 (C4), 128.9 (C2), 122.6 (C1), 117.8 (C3), 55.0 (C7),

* TLC indicated the complete consumption of **S2** within 15 min of charging the additional portion of triphosgene.

49.2 (C6), 33.3 (C8), 18.2 & 17.9 (C9 & C9’); HRMS (ESI+): found 205.1340; $C_{12}H_{17}N_2O$ $[M+H]^+$ requires 205.1341; v_{max} (thin film): 3238 (br), 1694, 1410, 1319, 1259, 1148; $[\alpha]_D^{20}$ -14.0 (c = 0.99, $CHCl_3$).

(*S*)-4-Isopropyl-1-(6-((*S*)-5-isopropyl-2-oxo-3-phenylimidazolidin-1-yl)pyrimidin-4-yl)-3-((2-nitrophenyl)sulfonyl)imidazolidin-2-one (10)

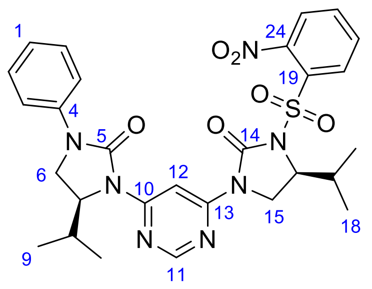

Prepared according to **General Procedure B** using deprotected urea **7** (300 mg, 1.47 mmol), chloropyrimidine **3** (718 mg, 1.69 mmol), $Pd_2(dba)_3$ (67 mg, 0.073 mmol), Xantphos (127 mg, 0.219 mmol), toluene (15 mL), and Cs_2CO_3 (1.20 g, 3.68 mmol). Additional portions of $Pd_2(dba)_3$ (67 mg, 0.073 mmol) and Xantphos (127 mg, 0.219 mmol) were charged after 22 h; TLC analysis revealed complete conversion of the urea starting material after a further 3 h. Purification by flash column chromatography (silica gel, dichloromethane:ethyl acetate, 49:1) afforded pyrimidine dimer **10** (624 mg, 72%) as a pale yellow solid. Diffraction-quality crystals were grown by the vapour diffusion method (ethyl acetate/hexane). δ_H (600 MHz, $CDCl_3$): 8.93 (1H, d, *J* 1.2, H12), 8.57 (1H, d, *J* 1.2, H11), 8.54-8.50 (1H, m, H23), 7.79-7.70 (3H, m, H20, H21 & H22), 7.59-7.55 (2H, m, H3), 7.40-7.35 (2H, m, H2), 7.13 (1H, tt, *J* 7.4, 1.1, H1), 4.74 (1H, ddd, *J* 9.3, 3.4, 2.8, H7), 4.45 (1H, ddd, *J* 8.5, 3.9, 2.9, H16), 4.14-4.06 (2H, m, H15), 3.94 (1H, t, *J* 9.4, H6), 3.62 (1H, dd, *J* 9.4, 2.8, H6’), 2.62-2.53 (1H, m, H8), 2.45-2.37 (1H, m, H17), 1.07 (3H, d, *J* 6.9, H18), 1.03 (3H, d, *J* 6.8, H18’), 0.99 (3H, d, *J* 7.0, H9), 0.81 (3H, d, *J* 6.9, H9’); δ_C (151 MHz, $CDCl_3$): 158.4 (C10/C13), 157.2 (C11 & C10/C13),* 154.0 (C5), 150.9 (C14), 148.1 (C24), 139.1 (C4), 135.7 (C23), 134.9 (C20/C21/C22), 132.4 (C20/C21/C22), 131.7 (C19), 129.1 (C2), 124.4 (C20/C21/C22), 124.1 (C1), 119.1 (C3), 97.0 (C12), 59.4 (C16), 55.5 (C7), 43.1(1) & 43.0(8) (C6 & C15), 32.6 (C17), 28.4 (C8), 18.3 (C9), 18.0 (C18), 15.1 (C18’), 14.3 (C9’); δ_H (600 MHz, d_6-DMSO): 8.83 (1H, d,*J* 1.2, H12), 8.70 (1H, d, *J* 1.1, H11), 8.37-8.35 (1H, m, H23), 8.12-8.09 (1H, m, H20), 8.03-7.97 (2H, m, H21 & H22), 7.64 (2H, d, *J* 7.7, H3), 7.31 (2H, t, *J* 8.2, H2), 7.14 (1H, t, *J* 7.2, H1), 4.69 (1H, dt, *J* 9.3, 2.8, H7), 4.37 (1H, ddd, *J* 9.1, 3.9, 2.4, H16), 4.15 (1H, dd, *J* 11.2, 9.2, H15), 4.04 (1H, dd, *J* 11.4, 2.4, H15’), 4.01 (1H, t, *J* 9.6, H6), 3.75 (1H, dd, *J* 9.8, 2.3, H6’), 2.49† (H8), 2.31-2.23 (1H, m, H17), 1.00 (3H, d, *J* 6.9, H18), 0.96 (3H, d, *J* 7.0, H9), 0.94 (3H, d, *J* 6.8, H18’), 0.72 (3H, d, *J* 7.2, H9’); HRMS (ESI+): found 594.2291; $C_{28}H_{32}N_7O_6S$, [M+H]+ requires 594.2134; v_{max} (thin film): 2970, 1718 (br), 1542, 1170, 1109, 598 cm^{-1}; $[\alpha]_D^{20}$ +25.2 (c = 0.64, $CHCl_3$).

---

* Signals overlap; identified through HSQC and HMBC cross-peaks.

† Peak overlaps with residual protic DMSO peak. Chemical shift obtained from COSY correlations to H9 and H9’.

(*S*)-4-Benzyl-1-(6-((*S*)-5-benzyl-2-oxo-3-phenylimidazolidin-1-yl)pyridazin-3-yl)-3-((2-nitrophenyl)sulfonyl)imidazolidin-2-one (11)

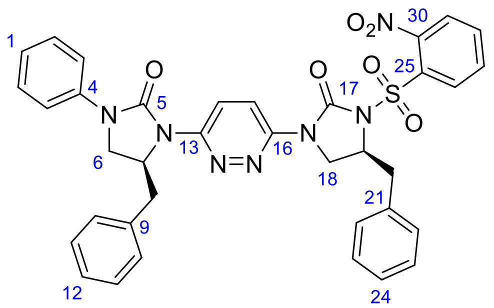

Prepared according to **General Procedure B** using deprotected urea **9** (67 mg, 0.27 mmol), bromopyridazine **4** (150 mg, 0.289 mmol), $Pd_2(dba)_3$ (12 mg, 0.013 mmol), Xantphos (23 mg, 0.040 mmol), toluene (3 mL), and Cs_2CO_3 (212 mg, 0.651 mmol). Reaction time = 4 h. Purification by flash column chromatography (silica gel, dichloromethane:petrol:ethyl acetate, 11:9:2) afforded pyridazine dimer **11** (182 mg, 99%) as a yellow solid. Diffraction-quality crystals were grown by the vapour diffusion method (ethyl acetate/petrol). δ_H (600 MHz, $CDCl_3$): 8.56 (1H, d, *J* 10.0, H14), 8.54-8.50 (1H, m, H29), 8.16 (1H, d, *J* 10.0, H15), 7.85-7.76 (3H, m, H26, H27 & H28), 7.42-7.36 (4H, m, H3 & H22), 7.35-7.27 (6H, m, $H2_{LHS}$, $H23_{LHS}$ & $H11_{RHS}$), 7.27-7.21 (4H, m, $H12_{LHS}$, $H24_{LHS}$ & $H10_{RHS}$),* 7.10 (1H, tt, *J* 7.3, 1.2, H1), 5.17 (1H, app. tt, *J* 8.8, 3.1, H7), 4.93-4.87 (1H, m, H19), 4.29-4.21 (2H, m, H18 & H18'), 3.96 (1H, t, *J* 9.0, H6), 3.65 (1H, dd, *J* 9.3, 2.8, H6'), 3.46 (1H, app. t, *J* 3.5, H8), 3.44 (1H, app. t, *J* 3.5, H20), 3.16 (1H, dd, *J* 13.7, 8.7, H20'), 2.95 (1H, dd, *J* 13.6, 8.7, H8'); δ_C (151 MHz, $CDCl_3$): 154.1 (C5), 152.2 (C13), 151.0 (C17), 150.3 (C16), 148.1 (C30), 139.1 (C4), 136.2 (C9), 135.3 (C26/C27/C28), 134.9 (C21 & C29),† 132.3 (C26/C27/C28), 131.7 (C25), 129.8 (C22), 129.6 (C10), 129.0(4), 128.9(6) & 128.8 (C2, C11 & C23), 127.5 (C24), 127.2 (C12), 124.7 (C26/C27/C28), 124.1 (C1), 120.4 (C14), 119.7 (C15), 119.0 (C3), 55.9 (C19), 52.3 (C7), 47.0 (C6), 46.5 (C18), 41.8 (C20), 37.9 (C8); HRMS (ESI+): found 690.2134; $C_{36}H_{32}N_7O_6S$, $[M+H]^+$ requires 690.2135; ν_{max} (thin film): 1732, 1713, 1541, 1398, 1172, 734 cm^{-1}; $[\alpha]_D^{20}$ +117.7 (*c* = 1.25, $CHCl_3$).

(*S*)-4-Benzyl-1-(5-((*S*)-5-benzyl-2-oxo-3-phenylimidazolidin-1-yl)pyrazin-2-yl)-3-((2-nitrophenyl)sulfonyl)imidazolidin-2-one (12)

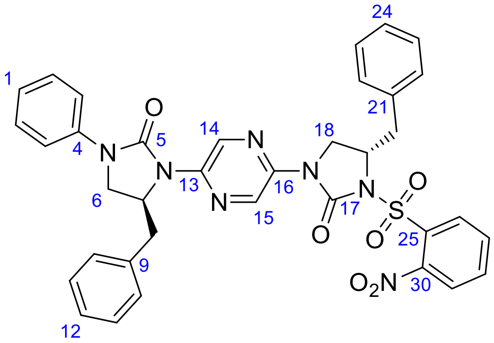

Prepared twice on different scales according to **General Procedure B** using deprotected urea **9** (1.0 eq.), bromopyrazine **5** (1.1 eq.), $Pd_2(dba)_3$ (5 mol%), Xantphos (15 mol%), toluene (*ca.* 0.1 M) and Cs_2CO_3 (2.5 eq.). Purification by flash column chromatography (silica gel, petrol:ethyl acetate, 3:1, then dichloromethane:ethylacetate,

* Overlap with $CHCl_3$ leads to over-integration of this multiplet. The integral of 4 was obtained by Lorentzian deconvolution of the multiplet within Topspin®.

† Signals overlap; identified through HSQC and HMBC cross-peaks.

20:1)* afforded pyrazine dimer **12** as a beige solid in yields of 80 mg (98%) and 103 mg (84%) from 30 and 45 mg, respectively, of deprotected urea **9**. Average yield = 91% (*n* = 2). Diffraction-quality crystals were grown by the vapour diffusion method (chloroform/petrol). δ_H (600 MHz, $CDCl_3$): 9.25 (1H, d, *J* 1.6, H14), 8.92 (1H, d *J* 1.6, H15), 8.57-8.53 (1H, m H29), 7.82-7.75 (3H, m, H26, H27 & H28), 7.47-7.43 (2H, m, H3), 7.38-7.31 (6H, m, $H22_{LHS}$, H2 & $H23_{RHS}$), 7.31-7.27 (2H, m, H11), 7.27-7.22 (app. 3H,† m, H12 & H24), 7.20-7.17 (2H, m, H10), 7.10 (1H, tt, *J* 7.4, 1.1, H1), 4.93 (1H, app. tt, *J* 9.1, 3.1, H7), 4.90-4.84 (1H, m, H19), 4.16 (1H, dd, *J* 10.8, 8.5, H18), 3.99 (1H, dd, *J* 10.8, 1.8, H18'), 3.92 (1H, t, *J* 9.0, H6), 3.64 (1H, dd, *J* 9.2, 3.1, H6'), 3.40 (1H, dd, *J* 13.7, 3.5, H20), 3.28 (1H, dd, *J* 13.3, 3.0, H8), 3.16 (1H, dd, *J* 13.7, 8.6, H20'), 2.69 (1H, dd, *J* 13.3, 9.4, H8'); δ_C (151 MHz, $CDCl_3$): 153.9 (C5), 150.8 (C17), 148.1 (C30), 144.4 (C13), 142.2 (C16), 139.4 (C4), 136.5 (C9), 135.2 (C26/C27/C28), 135.0 (C21), 134.9 (C29), 133.7 (C14), 133.0 (C15), 132.2 (C26/C27/C28), 131.8 (C25), 129.8 (C22), 129.4 (C10), 129.1, 129.0 & 128.9 (C2, C11 & C23), 127.5 (C24), 127.2 (C12), 124.6 (C26/C27/C28), 123.9 (C1), 118.7 (C3), 56.1 (C19), 52.4 (C7), 47.2 (C6), 46.0 (C18), 41.8 (C20), 38.3 (C8); HRMS (ESI+): found 690.2138; $C_{36}H_{32}N_7O_6S$, $[M+H]^+$ requires 690.2135; ν_{max} (thin film): 1738, 1708, 1542, 1375, 1173, 732 cm^{-1}; $[\alpha]_D^{20}$ +99.8 (*c* = 0.91, $CHCl_3$).

(*S*)-4-Isopropyl-3-(6-((S)-4-isopropyl-2-oxoimidazolidin-1-yl)pyrimidin-4-yl)-1-phenylimidazolidin-2-one (S5)

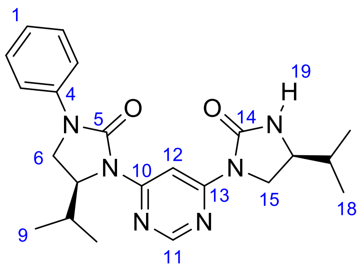

Prepared according to **General Procedure A** using *N*-nosyl urea **10** (543 mg, 0.915 mmol), K_2CO_3 (379 mg, 2.74 mmol), *N*,*N*-DMF (10 mL), and thiophenol (0.14 mL, 1.4 mmol). Reaction time = 4 h. Purification by flash column chromatography (silica gel, dichloromethane:ethyl acetate, 4:1) afforded the deprotected urea **S5** (338 mg, 90%) as a white solid. δ_H (600 MHz, $CDCl_3$): 9.15 (1H, d, *J* 1.2, H12), 8.56 (1H, d, *J* 1.2, H11), 7.66-7.62 (2H, m, H3), 7.38-7.33 (2H, m, H2), 7.10 (1H, tt, *J* 7.4, 1.0, H1), 6.26 (1H, br s, H19), 4.79-4.73 (1H, m, H7), 4.15 (1H, dd, *J* 10.8, 9.1, H15), 3.93 (1H, t, *J* 9.4, H6), 3.77 (1H, dd, *J* 10.8, 6.7, H15'), 3.63 (1H, dd, *J* 9.3, 3.0, H6'), 3.58-3.52 (1H, m, H16), 2.63-2.55 (1H, m, H8), 1.81-1.72 (1H, m, H17), 1.03 (3H, d, *J* 6.7, H18), 1.00 (3H, d, *J* 7.0, H9), 0.98 (3H, d, *J* 6.7, H18'), 0.81 (3H, d, *J* 6.9, H9'); δ_C (151 MHz, $CDCl_3$): 158.5 (C13), 158.0 (C14), 157.9 (C10), 157.0 (C11), 154.0 (C5), 139.5 (C4), 128.9 (C2), 123.6 (C1), 118.5 (C3), 96.4 (C12), 55.3 (C7), 54.8 (C16), 47.6 (C15), 42.8 (C6), 33.4 (C17), 28.5 (C8), 18.3 (C9), 18.2 (C18), 17.8 (C18'), 14.3 (C9'); HRMS (ESI+): found 409.2419; $C_{22}H_{29}N_6O_2$, $[M+H]^+$ requires 409.2352; ν_{max} (thin film): 3312 (br), 2955, 1732, 1705, 1578, 1246, 872, 748 cm^{-1}; $[\alpha]_D^{20}$ +71.4 (c = 1.42, $CHCl_3$).

* TLC indicated that the product should elute in the petrol/ethyl acetate solvent system. However, after extended flushing with this solvent no product was observed. It was noted that a significant amount of insoluble white material remained on top of the column. When the solvent was switched to dichloromethane/ethyl acetate this material dissolved and the product spot eluted rapidly. A dichloromethane/ethyl acetate solvent mixture is therefore recommended.

† Multiplet integration larger than expected due to spectral overlap with residual $CHCl_3$ peak.

(*S*)-4-Benzyl-3-(6-((*S*)-4-benzyl-2-oxoimidazolidin-1-yl)pyridazin-3-yl)-1-phenylimidazolidin-2-one (S6)

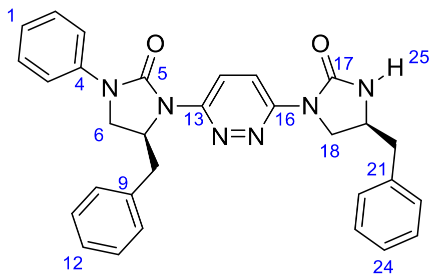

Prepared according to **General Procedure A** using *N*-nosyl urea **11** (124 mg, 0.180 mmol), K_2CO_3 (75 mg, 0.54 mmol), *N*,*N*-DMF (2 mL), and thiophenol (28 μL, 0.27 mmol). Reaction time = 2 h. Purification by flash column chromatography (silica gel, dichloromethane:ethyl acetate, 4:1) afforded the deprotected urea **S6** (77 mg, 85%) as a white solid. δ_H (600 MHz, $CDCl_3$): 8.65-8.54 (2H, m, H14 & H15), 7.46-7.39 (2H, m, H3), 7.39-7.20 (app. 13H,* m, H2, H10, H11, H12, H22, H23 & H24), 7.13-7.07 (1H, m, H1), 5.29-5.20 (1H, m, H7), 5.18 (1H, br s, H25), 4.44-4.32 (1H, m, H18), 4.17-4.06 (2H, m, H19$_{LHS}$ & H18'$_{RHS}$), 3.97 (1H, t, *J* 9.0, H6), 3.67 (1H, dd, *J* 9.2, 3.1, H6'), 3.49 (1H, dd, *J* 13.6, 3.2, H8), 3.01 (1H, dd, J 13.5, 5.2, H20), 2.97 (1H, dd, J 13.6, 8.9, H8'), 2.94-2.86 (1H, m, H20'); δ_C (151 MHz, $CDCl_3$): 157.8 (C17), 154.3 (C5), 151.9 & 151.4 (C13 & C16), 139.3 (C4), 136.6 (C21), 136.4 (C9), 129.7 (C10), 129.2 (C22), 129.1, 129.0, 128.8, 127.3 & 127.1 (C2, C11, C12, C23 & C24), 123.9 (C1), 120.6 & 119.7 (C14 & C15), 118.9 (C3), 52.2 (C7), 50.7 (C19), 49.5 (C18), 47.0 (C6), 42.6 (C20), 38.0 (C8). HRMS (ESI+): found 527.2325; $C_{30}H_{28}N_6NaO_2$, $[M+Na]^+$ requires 527.2171; ν_{max} (thin film): 3267 (br), 1720, 1443, 1389, 1227, 734, 691 cm^{-1}; $[\alpha]_D^{20}$ -26.1 (*c* = 1.00, $CHCl_3$).

(*S*)-4-Benzyl-3-(5-((*S*)-4-benzyl-2-oxoimidazolidin-1-yl)pyrazin-2-yl)-1-phenylimidazolidin-2-one (S7)

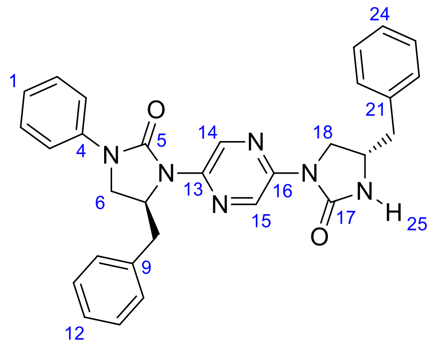

Prepared twice on different scales according to **General Procedure A** from *N*-nosyl urea **12**. Purification by flash column chromatography (silica gel, dichloromethane:ethyl acetate, 9:1) afforded the deprotected urea **S7** as a white solid in yields of 28 mg (96%) and 55 mg (86%) from 40 and 87 mg, respectively, of *N*-nosyl urea **12**. Average yield = 91% (*n* = 2). δ_H (600 MHz, $CDCl_3$): 9.33 (1H, d, *J* 1.6, H14/H15), 9.26 (1H, d, *J* 1.6, H14/H15), 7.52-7.46 (2H, m, H3), 7.41-7.20 (app. 13H,† m, H2, H10, H11, H12, H22, H23 & H24), 7.10 (1H, app. tt, *J* 7.3, 1.0, H1), 4.99 (1H, app. tt, *J* 9.1, 3.1, H7), 4.82 (1H, br s), 4.26 (1H, dd, *J* 10.5, 8.6, H18), 4.13-4.03 (1H, m, H19), 3.95 (1H, t, *J* 9.0, H6), 3.91 (1H, dd, *J* 10.6, 5.6, H18'), 3.67 (1H, dd, *J* 9.2, 3.2, H6'), 3.40 (1H, dd, *J* 13.4, 3.1, H8), 3.00 (1H, dd, *J* 13.5, 5.4, H20), 2.88 (1H, dd, *J* 13.5, 8.6, H20'), 2.77 (1H, dd, *J* 13.4, 9.4, H8'); δ_C (151 MHz, $CDCl_3$): 157.7 (C17), 154.1 (C5), 144.2 & 143.2 (C13 & C16), 139.6 (C4), 136.8 (C9), 136.7 (C21), 133.5 & 132.7 (C14 & C15), 129.6 (C10), 129.2 (C22), 129.1, 129.0 & 128.9 (C2, C11 & C23), 127.3 & 127.1 (C12 & C24), 123.6 (C1), 118.7 (C3), 52.4 (C7), 51.0 (C19), 49.1 (C18), 47.3 (C6), 42.6

* Multiplet integration larger than expected due to spectral overlap with residual $CHCl_3$ peak. HSQC analysis revealed this multiplet to contain 5 x 2H and 2 x 1H signals.

† Multiplet integration larger than expected due to spectral overlap with residual $CHCl_3$ peak. HSQC analysis revealed this multiplet to contain 5 x 2H and 2 x 1H signals.

(C20), 38.6 (C8); HRMS (ESI+): found 505.2353; $C_{30}H_{29}N_6O_2$, $[M+H]^+$ requires 505.2352; ν_{max} (thin film): 3235 (br), 1706 (br), 1470, 1375, 1143, 1002 cm^{-1}; $[\alpha]_D^{20}$ -63.1 (c = 0.68, $CHCl_3$).

(*S*)-4-Isopropyl-1-(6-((*S*)-5-isopropyl-2-oxo-3-phenylimidazolidin-1-yl)pyrimidin-4-yl)-3-(6-((*S*)-4-isopropyl-3-((2-nitrophenyl)sulfonyl)-2-oxoimidazolidin-1-yl)pyrimidin-4-yl)imidazolidin-2-one (13)

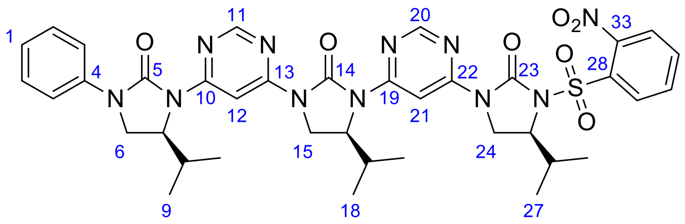

Prepared according to **General Procedure B** using deprotected urea **S5** (302 mg, 0.739 mmol), chloropyrimidine **3** (362 mg, 0.850 mmol), $Pd_2(dba)_3$ (34 mg, 0.037 mmol), Xantphos (64 mg, 0.11 mmol), toluene (8 mL), and Cs_2CO_3 (602 mg, 1.85 mmol). Reaction time = 18 h. Purification by flash column chromatography (silica gel, dichloromethane:ethyl acetate, 9:1) afforded pyrimidine trimer **13** (495 mg, 84%) as an off-white solid. δ_H (600 MHz, $CDCl_3$): 9.16 (1H, d, *J* 1.2, H12), 8.88 (1H, d, *J* 1.2, H21), 8.60 (1H, d, *J* 1.2, H11), 8.59 (1H, d, *J* 1.2, H20), 8.55-8.52 (1H, m, H32), 7.78-7.73 (1H, m, H31), 7.71-7.66 (4H, m, $H29_{LHS}$, $H30_{LHS}$ & $H3_{RHS}$), 7.44-7.39 (2H, m, H2), 7.15 (1H, tt, *J* 7.4, 1.1, H1), 4.78 (1H, app. dt, *J* 9.4, 3.1, H7), 4.73 (1H, app. dt, *J* 9.4, 3.3, H16), 4.45 (1H, ddd, *J* 8.9, 4.1, 2.5, H25), 4.11 (1H, dd, *J* 11.0, 8.9, H24), 4.08-4.03 (2H, m $H24'_{LHS}$ & $H15_{RHS}$), 3.99 (1H, t, *J* 9.4, H6), 3.92 (1H, dd, *J* 11.0, 9.4, H15'), 3.66 (1H, dd, *J* 9.3, 2.8, H6'), 2.66-2.58 (1H, m, H8), 2.57-2.49 (1H, m, H17), 2.45-2.37 (1H, m, H26), 1.07 (3H, d, *J* 7.0, H27), 1.04 (3H, d, *J* 6.9, H27'), 1.02-0.98 (6H, m, H9 & H18), 0.84 (3H, d, *J* 6.9, H9'), 0.77 (3H, d, *J* 6.9, H18'); δ_C (151 MHz, $CDCl_3$): 158.3 (C10), 158.0 (C19), 157.7 & 157.4 (C13 & C22), 157.0(9) & 157.0(5) (C11 & C20), 154.0 (C5), 153.2 (C14), 150.8 (C23), 148.0 (C33), 139.3 (C4), 135.8 (C32), 135.1 (C29/C30), 132.5 (C31), 131.5 (C28), 129.0 (C2), 124.4 (C29/C30), 123.9 (C1), 118.9 (C3), 98.0 (C21), 97.2 (C12), 59.4 (C25), 55.6 (C16), 55.5 (C7), 43.2 (C24), 43.0 (C6), 41.3 (C15), 32.6 (C26), 28.5 (C8), 28.4 (C17), 18.3 (C9), 18.2 (C18), 18.0 (C27), 15.2 (C27'), 14.4 (C9'), 14.2 (C18'). HRMS (ESI+): found 798.3146; $C_{38}H_{44}N_{11}O_7S$, $[M+H]^+$ requires 798.3146; ν_{max} (thin film): 2963, 1722 (br), 1577, 1365, 1172, 1111, 598 cm^{-1}; $[\alpha]_D^{20}$ -22.5 (c = 0.94, $CHCl_3$).

(*S*)-4-Benzyl-1-(6-((*S*)-5-benzyl-2-oxo-3-phenylimidazolidin-1-yl)pyridazin-3-yl)-3-(6-((*S*)-4-benzyl-3-((2-nitrophenyl)sulfonyl)-2-oxoimidazolidin-1-yl)pyridazin-3-yl)imidazolidin-2-one (14)

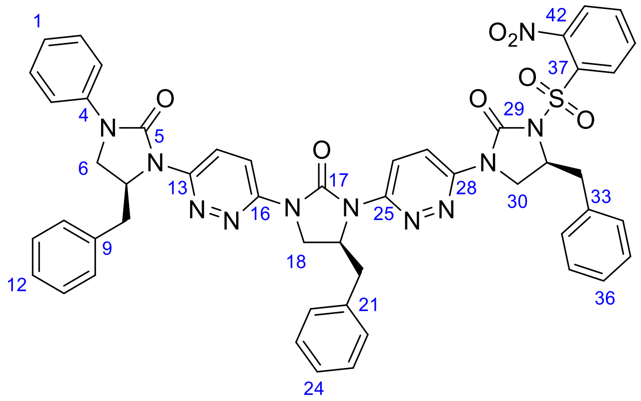

Prepared according to **General Procedure B** using deprotected urea **S6** (38 mg, 0.075 mmol), bromopyridazine **4** (43 mg, 0.083 mmol), $Pd_2(dba)_3$ (3.5 mg, 3.8 μmol), Xantphos (6.8 mg, 0.012 mmol), toluene (1 mL), and Cs_2CO_3 (62 mg, 0.19 mmol). Reaction time = 2 h. Purification by flash column chromatography (silica gel, dichloromethane:ethyl acetate, 19:1) afforded pyridazine trimer **14** (68 mg, 96%) as a pale yellow solid. δ_H (600 MHz, $CDCl_3$): 8.60 (1H, d, *J*, 10.0, H14), 8.55-8.51 (1H, m, H41), 8.49 (1H, d, *J* 9.9, H26), 8.37 (1H, d, *J* 10.0, H15), 8.21 (1H, d, *J* 9.9, H27), 7.85-7.77

(3H, m, H38, H39 & H40), 7.42-7.36 (4H, m, $H3_{LHS}$ & $H34_{RHS}$), 7.36-7.20 (app. 16H,* m, H2, H10, H11, H12, H22, H23, H24, H35, H36), 7.10 (1H, app. tt, *J* 7.4, 1.0, H1), 5.28-5.23 (1H, m, H19), 5.23-5.18 (1H, m, H7), 4.94-4.88 (1H, m, H31), 4.29-4.22 (3H, m, $H30_{LHS}$, $H30'_{LHS}$ & $H18_{RHS}$), 4.19 (1H, dd, *J* 10.9, 8.7, H18'), 3.97 (1H, t, *J* 9.0, H6), 3.67 (1H, dd, *J* 9.2, 2.9, H6'), 3.48 (1H, dd, *J* 13.5, 3.2, H8), 3.46-3.40 (2H, m, $H32_{LHS}$ & $H20_{RHS}$), 3.17 (1H, dd, *J* 13.8, 8.6, H32'), 3.04 (1H, dd, *J* 13.7, 8.4, H20'), 2.97 (1H, dd, *J* 13.6, 8.8, H8'); δ_C (151 MHz, $CDCl_3$): 154.2 (C5), 153.8 (C17), 151.9 (C13), 151.8 (C25), 151.2 (C16), 151.1 (C29), 150.7 (C28), 148.1 (C42), 139.2 (C4), 136.3 (C9), 135.8 (C21), 135.3 (C38/C39/C40), 134.9(2) & 134.8(8) (C33 & C41), 132.3 (C38/C39/C40), 131.7 (C37), 129.8 (C34), 129.7 (C10), 129.6 (C22), 129.0(4), 128.9(7), 128.8(2) & 128.7(8) (C2, C11, C23 & C35), 127.5 (C36), 127.2 & 127.1 (C12 & C24), 124.7 (C38/C39/C40), 124.0 (C1), 120.6 (C26), 120.4 (C14), 119.8(3) & 119.7(8) (C15 & C27), 118.9 (C3), 56.0 (C31), 52.6 (C19), 52.3 (C7), 47.0 (C6), 46.5 (C30), 45.8 (C18), 41.8 (C32), 38.1 (C20), 38.0 (C8); HRMS (ESI+): found 942.3158; $C_{50}H_{44}N_{11}O_7S$, $[M+H]^+$ requires 942.3146; ν_{max} (thin film): 1716 (br), 1431, 1389, 1284, 1112, 739 cm^{-1}; $[\alpha]_D^{20}$ +56.5 (*c* = 1.00, $CHCl_3$).

(*S*)-4-Benzyl-1-(5-((*S*)-5-benzyl-2-oxo-3-phenylimidazolidin-1-yl)pyrazin-2-yl)-3-(5-((*S*)-4-benzyl-3-((2-nitrophenyl)sulfonyl)-2-oxoimidazolidin-1-yl)pyrazin-2-yl)imidazolidin-2-one (15)

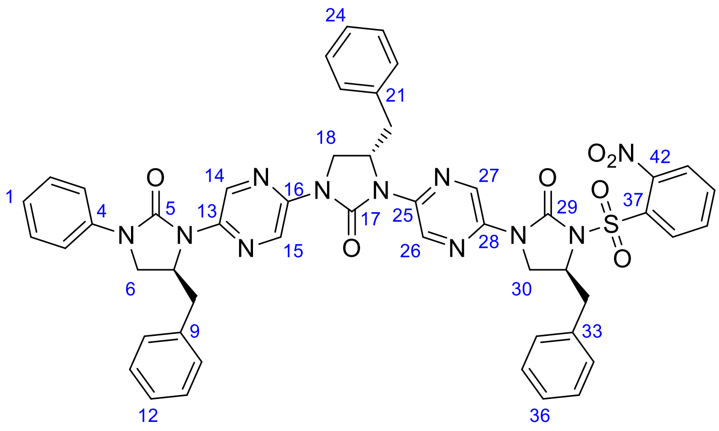

Prepared according to **General Procedure B** using deprotected urea **S7** (24 mg, 0.048 mmol), bromopyrazine **5** (27 mg, 0.052 mmol), $Pd_2(dba)_3$ (2.2 mg, 2.4 μmol), Xantphos (4.1 mg, 7.1 μmol), toluene (0.5 mL), and Cs_2CO_3 (39 mg, 0.12 mmol). Additional portions of $Pd_2(dba)_3$ (2.2 mg, 2.4 μmol), Xantphos (4.1 mg, 7.1 μmol) and Cs_2CO_3 (39 mg, 0.12 mmol) were charged after 7 h; TLC analysis revealed complete conversion of the urea starting material after a further 14 h. Purification by flash column chromatography (silica gel, dichloromethane:petrol:ethyl acetate, 12:6:1) afforded pyrazine trimer **15** (24 mg, 54%) as a yellow solid. δ_H (600 MHz, $CDCl_3$): 9.26 (2H, app. d, *J* 1.5, H14/H15 & H26), 9.22 (1H, d, *J* 1.6, H14/H15), 8.96 (1H, d, *J*, 1.6, H27), 8.58-8.53 (1H, m, H41), 7.84-7.75 (3H, m, H38, H39 & H40), 7.50-7.46 (2H, m, H3), 7.41-7.23 (app. 16 H,† m, H2, H10, H11, H12/H24, H23, H34, H35 & H36), 7.23-7.20 (1H, m, H12/H24), 7.19-7.16 (2H, m, H22), 7.10 (1H, app. tt, *J* 7.4, 1.0, H1), 5.03-4.96 (2H, m, $H19_{LHS}$ & $H7_{RHS}$), 4.92-4.86 (1H, m, H31), 4.17 (1H, dd, *J* 10.8, 8.6, H30), 4.05 (1H, dd, *J* 10.7, 8.5, H18), 4.03-3.99 (2H, m, $H18'_{LHS}$ & $H30'_{RHS}$), 3.95 (1H, t, *J* 9.0, H6), 3.68 (1H, dd, *J* 9.2, 3.2, H6'), 3.44-3.37 (2H, m, $H32_{LHS}$ & $H8_{RHS}$), 3.23 (1H, dd, *J* 13.3, 2.9, H20), 3.17 (1H, dd, *J* 13.8, 8.5, H32'), 2.81-2.74 (2H, m, $H20'_{LHS}$ & $H8'_{RHS}$); δ_C (151 MHz, $CDCl_3$): 154.1 (C5), 153.4 (C17), 150.8 (C29), 148.1 (C42), 144.0 (C25), 143.8 & 143.5 (C13 & C16), 142.5 (C28), 139.5 (C4), 136.6 (C9), 136.1 (C21), 135.2 (C38/C39/C40), 134.9(5) & 134.9(1) (C33 & C41), 133.8 (C14/C15), 133.7 (C26), 133.1 (C27), 132.9 (C14/C15), 132.2 (C38/C39/C40), 131.8 (C37), 129.8 (C34), 129.5(5) & 129.5(0) (C10 & C22), 129.0(6), 129.9(9), 128.9(0) & 128.8(5) (C2, C11, C23 & C35), 127.5 (C36), 127.2 & 127.1 (C12 & C24), 124.6 (C38/C39/C40), 123.7 (C1), 118.7 (C3), 56.1 (C31), 52.8 (C19), 52.4 (C7), 47.3 (C6), 46.0 (C30), 45.6 (C18), 41.8 (C32), 38.5 (C8), 38.4 (C20); HRMS (ESI+):

---

* Multiplet integration larger than expected due to spectral overlap with residual $CHCl_3$ peak. HSQC analysis revealed this multiplet to contain 6 x 2H and 3 x 1H signals

† Observed integration greater than predicted due to co-incidence with the residual $CHCl_3$ peak. HSQC analysis revealed this multiplet to contain 6 x 2H and 2 x 1H signals.

found 942.3147; $C_{50}H_{44}N_{11}O_7S$, $[M+H]^+$ requires 942.3146; ν_{max} (thin film): 1733, 1710, 1467, 1368, 1173, 725 cm^{-1}; $[\alpha]_D^{20}$ +63.8 (*c* = 0.86, $CHCl_3$).

(*S*)-4-Isopropyl-1-(6-((*S*)-5-isopropyl-2-oxo-3-phenylimidazolidin-1-yl)pyrimidin-4-yl)-3-(6-((*S*)-4-isopropyl-2-oxoimidazolidin-1-yl)pyrimidin-4-yl)imidazolidin-2-one (S8)

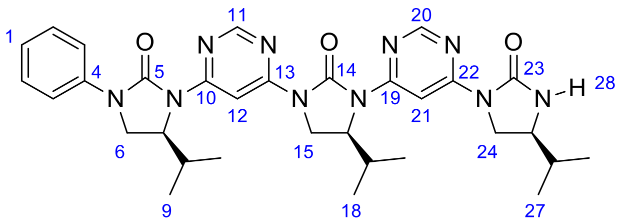

Prepared according to **General Procedure A** using *N*-nosyl urea **13** (422 mg, 0.529 mmol), K_2CO_3 (220 mg, 1.59 mmol), *N*,*N*-DMF (6 mL), and thiophenol (82 *µ*L, 0.80 mmol). Reaction time = 4 h. Purification by flash column chromatography (silica gel, dichloromethane:ethyl acetate, 7:3) afforded the deprotected urea **S8** (310 mg, 96%) as a white solid. δ_H (600 MHz, $CDCl_3$): 9.22 (1H, d, *J* 1.1, H12), 9.08 (1H, d, *J* 1.1, H21), 8.60 (1H, d, *J* 1.1, H11), 8.57 (1H, d, *J* 1.1, H20), 7.68-7.63 (2H, m, H3), 7.38-7.33 (2H, m, H2), 7.10 (1H, tt, *J* 7.4, 1.0, H1), 6.27 (1H, br s, H28), 4.82-4.74 (2H, m, $H16_{LHS}$ & $H7_{RHS}$), 4.11 (1H, dd, *J* 10.3, 9.4, H24), 4.05 (1H, dd, *J* 11.0, 3.3, H15), 3.99-3.92 (2H, m, $H15'_{LHS}$ & $H6_{RHS}$), 3.74 (1H, dd, *J* 10.7, 6.7, H24'), 3.65 (1H, dd, *J* 9.3, 2.9, H6'), 3.59-3.52 (1H, m, H25), 2.65-2.58 (1H, m, H8), 2.58-2.50 (1H, m, H17), 1.76-1.68 (1H, m, H26), 1.03-0.98 (9H, app. d, *J* 6.8, H9, H18 & H27), 0.91 (3H, d, *J* 6.8, H27'), 0.82 (3H, d, *J* 6.9, H9'), 0.79 (3H, d, *J* 6.9, H18'); δ_C (151 MHz, $CDCl_3$): 158.7 (C19/C22), 158.2 & 157.9 (C10 & C13), 157.8 (C23), 157.4 (C19/C22), 156.9(6) & 156.9(2) (C11 & C20), 153.7 (C5), 153.3 (C14), 139.4 (C4), 129.0 (C2), 123.6 (C1), 118.6 (C3), 97.4(4) (C21), 97.3(6) (C12), 55.4 (C7 & C16),* 54.8 (C25), 47.7 (C24), 42.8 (C6), 41.3 (C15), 33.4 (C26), 28.4(9) & 28.4(6) (C8 & C17), 18.3, 18.2 & 18.1 (C9, C18 & C27), 17.8 (C27'), 14.3 (C9'), 14.2 (C18'). HRMS (ESI+): found 613.3368; $C_{32}H_{41}N_{10}O_3$, $[M+H]^+$ requires 613.3363; ν_{max} (thin film): 3244 (br), 2960, 1718 (br), 1578, 1364, 1239, 748 cm^{-1}; $[\alpha]_D^{20}$ +88.2 (*c* = 0.88, $CHCl_3$).

(*S*)-4-Isopropyl-1-(6-((*S*)-5-isopropyl-2-oxo-3-phenylimidazolidin-1-yl)pyrimidin-4-yl)-3-(6-((*S*)-4-isopropyl-3-(6-((*S*)-4-isopropyl-3-((2-nitrophenyl)sulfonyl)-2-oxoimidazolidin-1-yl)pyrimidin-4-yl)-2-oxoimidazolidin-1-yl)pyrimidin-4-yl)imidazolidin-2-one (16)

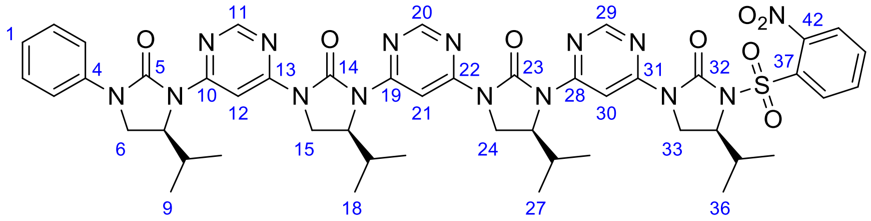

Prepared according to **General Procedure B** using deprotected urea **S8** (35 mg, 57 *µ*mol), chloropyrimidine **3** (27 mg, 63 *µ*mol), $Pd_2(dba)_3$ (2.8 mg, 3.1 *µ*mol), Xantphos (5.2 mg, 9.0 *µ*mol), toluene (0.6 mL), and Cs_2CO_3 (47 mg, 0.14 mmol). Reaction time = 15 h. Purification by flash column chromatography (silica gel, dichloromethane:ethyl acetate, 4:1) afforded pyrimidine tetramer **16** (25 mg, 44%) as a yellow solid. δ_H (600 MHz, $CDCl_3$): 9.16 (1H, d, *J* 1.2, H12), 8.90 (1H, d, *J* 1.2, H21), 8.80 (1H, d, *J* 1.1, H30), 8.65(1) (1H, d, *J* 1.1, H11), 8.64(9) (1H, d, *J* 1.2, H20), 8.60 (1H, d, *J* 1.1, H29), 8.41 (1H, dd, *J* 8.1, 1.3, H41), 7.69 (1H, td, *J* 7.8, 1.3, H40), 7.64-7.61 (2H, m, H3), 7.45 (1H, dd, *J* 7.9, 1.3, H38), 7.40 (1H, td, *J* 7.7, 1.3, H39), 7.36-7.33 (2H, m, H2), 7.10 (1H, tt, *J* 7.5, 0.9, H1), 4.84-4.79 (2H, m, $H16_{LHS}$ & $H7_{RHS}$), 4.75 (1H, tt, *J* 9.5, 3.4, H25), 4.42 (1H, dt, *J* 7.8, 3.9, H34), 4.11-4.03 (4H, m, H15, H24, H33 & H33'), 4.01 (1H, dd, *J* 10.9, 9.6, H15'), 3.94-3.90 (2H, m, H6 & H24'), 3.70 (1H, dd, *J* 9.3, 3.3, H6'), 2.69-2.61 (1H, m, H8), 2.60-2.50 (2H, m, $H17_{LHS}$ & $H26_{RHS}$), 2.39-2.32 (1H, m, H35), 1.06-0.98 (15H, m, H9, H18, H27, H36 & H36'), 0.85 (3H,

* Signals overlap; identified through HSQC and HMBC cross-peaks.

d, J 5.1, H9'/H18'), 0.84 (3H, d, J 5.1, H9'/H18'), 0.78 (3H, d, J 6.9, H27'); δ_C (151 MHz, $CDCl_3$): 158.3, 157.9(15), 157.9(05), 157.8(6), 157.8(5) & 157.4 (C10, C13, C19, C22, C28 & C31), 157.0(3), 156.9(5) & 156.9 (C11, C20 & C29), 153.8 (C5), 153.2 (C14), 153.0 (C23), 150.7 (C32), 147.6 (C42), 139.2 (C4), 135.7 (C41), 135.0 (C39), 132.4 (C40), 130.8 (C37), 129.1 (C2), 123.9(1) & 123.9(0) (C1 & C38), 119.0 (C3), 99.0 (C21), 98.0 (C30), 97.5 (C12), 59.2 (C34), 55.5(2), 55.4(5) & 55.3 (C7, C16 & C25), 43.1 (C33), 43.0 (C6), 41.4 & 41.3 (C15 & C24), 32.5 (C35), 28.3 (C8), 28.2(1) & 21.1(9) (C17 & C26), 18.1(1), 18.0(8) & 18.0(6) (C9, C18 & C27), 17.9 (C36), 15.1 (C36'), 14.3, 14.2 & 14.1 (C9', C18' & C27'); HRMS (ESI+): found 1002.4157; $C_{48}H_{56}N_{15}O_8S$, $[M+H]^+$ requires 1002.4157; v_{max} (thin film): 1724 (br), 1577, 1362, 1212, 1172, 739 cm^{-1}; $[\alpha]_D^{20}$ +107.2 (c = 0.78, $CHCl_3$).

(*S*)-*N*-(1-(*tert*-Butylamino)-3-phenylpropan-2-yl)-2-nitrobenzenesulfonamide (S9)

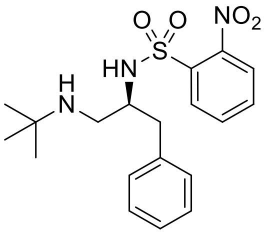

tert-Butylamine (1.5 mL, 14 mmol) was added in one portion to a stirred, room-temperature solution of aziridine **S4** (1.00 g, 3.14 mmol) in acetonitrile (3 mL). After 1 h the reaction was complete (TLC: 5% MeOH/CH_2Cl_2), and the volatiles were removed *in vacuo*. The crude residue was purified by flash column chromatography (silica gel, 2.5→5% methanol in dichloromethane) to afford diamine **S9** (1.18 g, 96%) as a light yellow oil. δ_H (600 MHz, $CDCl_3$): 8.01-7.98 (1H, m), 7.80-7.76 (1H, m), 7.66-7.63 (2H, m), 7.15-7.11 (2H, m), 7.11-7.07 (3H, m), 3.63 (1H, ddd, J 12.2, 6.9, 5.3), 2.89 (1H, dd, J 14.0, 6.8), 2.83 (1H, dd, J 13.8, 7.2), 2.60 (1H, dd, J 12.2, 4.9), 2.56 (1H, dd, J 12.1, 5.6), 0.95 (9H, s); δ_C (151 MHz, $CDCl_3$): 147.5, 137.4, 134.7, 133.1, 132.8, 130.5, 129.2, 128.4, 126.6, 125.4, 57.2, 50.2, 45.4, 40.0, 29.0; HRMS (ESI+): found 392.1646; $C_{19}H_{26}N_3O_4S$, $[M+H]^+$ requires 392.1644; $[\alpha]_D^{20}$ -0.9 (c = 1.14, $CHCl_3$); v_{max} (thin film): 3343 (br), 2965, 1541, 1362, 1170 cm^{-1}.

(*S*)-4-Benzyl-1-(*tert*-butyl)-3-((2-nitrophenyl)sulfonyl)imidazolidin-2-one (S10)

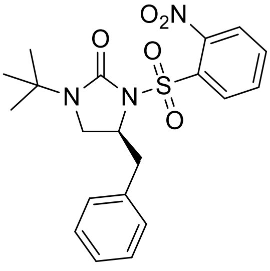

A solution of triphosgene (266 mg, 0.896 mmol) in dry acetonitrile (10 mL) was added dropwise over 15 min to a stirred 0 °C solution of diamine **S9** (1.00 g, 2.55 mmol) and Hünig's base (1.34 mL, 7.69 mmol) in dry acetonitrile (15 mL). After stirring for a further 30 min the reaction mixture was diluted with diethyl ether (100 mL) and the organic layer was washed with ammonium chloride (sat. aq., 2 x 25 mL) and brine (1 x 25 mL), dried over anhydrous magnesium sulfate, filtered, and concentrated *in vacuo*. The residue was dissolved in diethyl ether (100 mL) and passed through a short plug of silica (~10 g), and the plug was washed with diethyl ether (50 mL). The ether was removed *in vacuo* to afford **S10** (880 mg, 83%) as a viscous pale yellow oil. δ_H (600 MHz, $CDCl_3$): 8.48-8.44 (1H, m), 7.77-7.73 (2H, m), 7.73 -7.70 (1H, m), 7.38-7.32 (4H, m), 7.30-7.27 (1H, m), 4.53 (1H, dddd, J 8.6, 7.8, 3.8, 1.8), 3.54 (1H, t, J 8.9), 3.24 (1H, dd, J 9.3, 1.9), 3.19 (1H, dd, J 13.7, 3.7), 3.10 (1H, dd, J 13.8, 7.8), 1.10 (9H, s); δ_C (151 MHz, $CDCl_3$) 152.2, 147.8, 135.5, 134.4, 134.3, 132.5, 132.0, 129.8, 128.8, 127.2, 124.1, 54.6, 53.9, 44.7, 41.3, 27.1; HRMS (ESI+): found 418.1439; $C_{20}H_{24}N_3O_5S$, $[M+H]^+$ requires 418.1437; $[\alpha]_D^{20}$ +323.3 (c = 1.00, $CHCl_3$); v_{max} (thin film): 2976, 1724, 1541, 1364, 1169, 1126 cm^{-1}.

(*S*)-4-Benzyl-1-(*tert*-butyl)imidazolidin-2-one (S11)

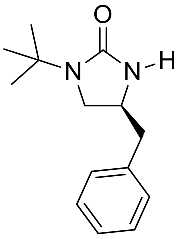

This reaction was carried out by analogy to the published procedure.[6] A stirred, room-temperature suspension of *N*-nosylurea **S10** (314 mg, 0.752 mmol) and K_2CO_3 (311 mg, 2.25 mmol) in *N*,*N*-DMF (8 mL) was de-gassed by sparging with nitrogen for 15 min. Thiophenol (115 μL, 1.12 mmol) was added, with the immediate development of a rich yellow colour. After 1 h the reaction mixture was concentrated *in vacuo* and taken up in dichloromethane (50 mL). Sodium bicarbonate solution (sat. aq., 25 mL) was added, the layers were separated, and the aqueous phase was extracted with dichloromethane (2 x 25 mL). The combined organic layers were dried over anhydrous magnesium sulfate, filtered, and concentrated *in vacuo*. The crude residue was purified by flash column chromatography (silica gel, dichloromethane:methanol, 100:1) to afford **S11** (142 mg, 81%) as a beige crystalline solid. *The ^1H data obtained agree with those obtained for the racemic compound*.[7] δ_H (600 MHz, $CDCl_3$): 7.31 (2H, t, *J* 7.7), 7.26-7.22 (1H, m), 7.18 (2H, d, *J* 7.6), 4.50-4.37 (1H, br m), 3.77-3.70 (1H, m), 3.50 (1H, td, *J* 8.4, 2.4), 3.16 (1H, dd, *J* 8.6, 6.3), 2.83-2.74 (2H, m), 1.34 (9H, s); δ_C (151 MHz, $CDCl_3$): 161.7, 137.4, 129.0, 128.8, 126.8, 52.8, 50.8, 48.7, 41.7, 27.6; HRMS (ESI+): found 233.1656; $C_{14}H_{20}N_2O$, $[M+H]^+$ requires 233.1654; $[\alpha]_D^{20}$ -29.3 (c = 1.05, $CHCl_3$); ν_{max} (thin film): 3231 (br), 2972, 1694, 1257, 703 cm^{-1}.

(*S*)-3-(6-Bromopyridazin-3-yl)-4-isopropyl-1-(6-((*S*)-5-isopropyl-3-(6-((*S*)-5-isopropyl-2-oxo-3-phenylimidazolidin-1-yl)pyrimidin-4-yl)-2-oxoimidazolidin-1-yl)pyrimidin-4-yl)imidazolidin-2-one (17)

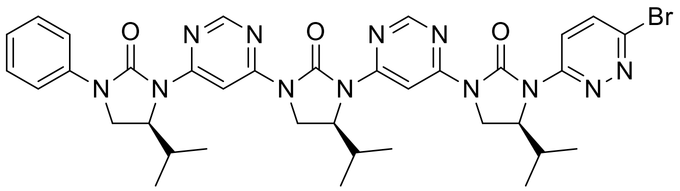

Prepared according to **General Procedure B** using deprotected urea **S8** (35 mg, 0.057 mmol), 3,6-dibromopyridazine (68 mg, 0.29 mmol), $Pd_2(dba)_3$ (2.6 mg, 2.8 μmol), Xantphos (5.0 mg, 8.6 μmol), toluene (0.5 mL), and Cs_2CO_3 (47 mg, 0.14 mmol). Reaction time = 1 h. Purification by flash column chromatography (silica gel, dichloromethane:ethyl acetate, 17:3) afforded bromopyridazine **17** (38 mg, 86%) as a beige solid. δ_H (600 MHz, $CDCl_3$): 9.21 (1H, d, *J* 1.1), 9.20 (1H, d, *J* 1.1), 8.71 (1H, d, *J* 9.4), 8.65 (1H, d, *J* 1.1), 8.62 (1H, d, *J* 1.1), 7.63 (2H, app. d, *J* 7.7), 7.58 (1H, d, *J* 9.4), 7.40 (2H, app. t, *J* 8.0), 7.15 (1H, app. t, *J* 7.4), 4.96 (1H, dt, *J* 9.4, 3.5), 4.82-4.77 (2H, m), 4.11 (2H, app. dt, *J* 10.9, 3.3), 4.05 (1H, dd, *J* 11.0, 9.5), 4.01-3.95 (2H, m), 3.66 (1H, dd, *J* 9.2, 2.8), 2.74-2.67 (1H, m), 2.67-2.57 (2H, m), 1.05-1.00 (9H, m), 0.85 (3H, d, *J* 6.9), 0.82 (3H, d, *J* 6.9), 0.80 (3H, d, *J* 6.9); δ_C (151 MHz, $CDCl_3$): 158.2, 157.8, 157.7, 157.0, 156.9, 153.9(4), 153.8(9), 153.4, 153.2, 142.4, 139.2, 132.4, 129.0, 123.9, 121.3, 119.0, 97.6, 97.3, 55.6, 55.5(3), 55.4(8), 43.0, 41.4, 41.3, 29.7, 28.3(9), 28.3(6), 27.3, 18.2, 18.1, 18.0, 14.3, 14.1, 14.0; $[\alpha]_D^{20}$ -8.3 (c = 0.82, $CHCl_3$);

v_{max} (thin film): 2961, 2929, 1727, 1578, 1407, 1245 cm^{-1}; HRMS (ESI+): found 769.2687; $C_{36}H_{42}BrN_{12}O_3$, [M(^{79}Br)+H]$^+$ requires 769.2686.

(*S*)-4-Benzyl-3-(6-((*S*)-4-benzyl-3-((2-nitrophenyl)sulfonyl)-2-oxoimidazolidin-1-yl)pyridazin-3-yl)-1-(*tert*-butyl)imidazolidin-2-one (18)

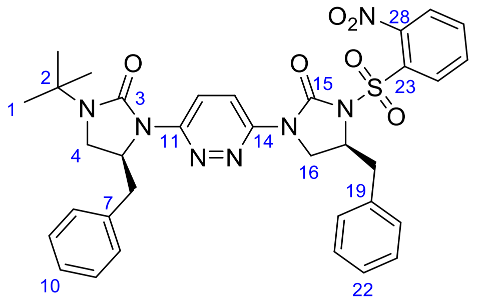

Prepared according to **General Procedure B** using deprotected urea **S11** (49 mg, 0.21 mmol), bromopyridazine **4** (100 mg, 0.193 mmol), $Pd_2(dba)_3$ (9.0 mg, 9.8 μmol), Xantphos (17 mg, 0.029 mmol), toluene (2 mL), and Cs_2CO_3 (157 mg, 0.482 mmol. Reaction time = 16 h. Purification by flash column chromatography (silica gel, dichloromethane:ethyl acetate, 50:1) afforded *N*-*tert*-butyl urea **18** (62 mg, 48%) as a yellow solid. δ_H (600 MHz, $CDCl_3$): 8.55-8.51 (2H, m, H12$_{LHS}$ & H27$_{RHS}$), 8.08 (1H, d, *J* 10.0, H13), 7.83-7.76 (3H, m, H24, H25 & H26), 7.38-7.35 (2H, m, H20), 7.32-7.28 (4H, m, H9 & H21), 7.27-7.24 (3H, m, H10),* 7.24-7.20 (1H, m, H22), 7.19-7.16 (2H, m, H8), 4.95-4.90 (1H, m, H5), 4.88 (1H, app. dtd, *J* 8.8, 5.3, 3.6, H17), 4.23-4.20 (2H, m, H16 & H16'), 3.47-3.41 (2H, m, H4$_{LHS}$ & H18$_{RHS}$), 3.30 (1H, dd, *J* 9.1, 2.6, H4'), 3.19-3.11 (2H, m, H6$_{LHS}$ & H18'$_{RHS}$), 3.01 (1H, dd, *J* 13.6, 7.6, H6'), 1.23 (9H, s, H1); δ_C (151 MHz, $CDCl_3$): 155.9 (C3), 152.4 (C11), 150.9 (C15), 149.6 (C14), 148.0 (C28), 136.6 (C7), 135.1 (C24/C25/C26), 134.8(2) (C19), 134.7(5) (C27), 132.1 (C24/C25/C26), 131.7 (C23), 129.7 & 129.6 (C8 & C20), 128.8 & 128.6 (C9 & C21), 127.3 (C22), 126.8 (C10), 124.6 (C24/C25/C26), 119.9 (C12), 119.4 (C13), 55.8 (C17), 53.5 (C2), 51.5 (C5), 46.3 (C16), 44.1 (C4), 41.7 (C18), 37.2 (C6), 27.3 (C1); HRMS (ESI+): found 670.2449; $C_{34}H_{36}N_7O_6S$, [M+H]$^+$ requires 670.2448; v_{max} (thin film): 1733, 1695, 1542, 1440, 1360, 1173 cm^{-1}; $[\alpha]_D^{20}$ +147.0 (*c* = 0.33, $CHCl_3$).

(*S*)-4-Benzyl-1-(6-((*S*)-5-benzyl-2-oxoimidazolidin-1-yl)pyridazin-3-yl)-3-((2-nitrophenyl)sulfonyl)imidazolidin-2-one (19)

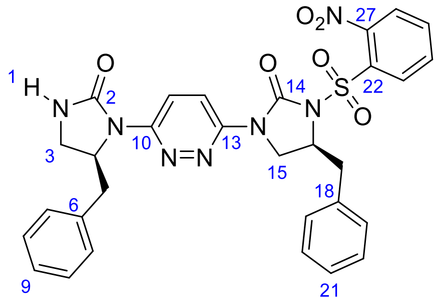

To a stirred, 0 °C solution of *N*-*tert*-butyl urea **18** (45 mg, 0.067 mmol) in TFA (1 mL) was carefully added TfOH (*ca.* 0.5 mL, 6 mmol) dropwise by glass pipette over 5 min. The reaction was allowed to warm to room temperature. After 1 h, the reaction mixture was concentrated *in vacuo*, and the crude residue was taken up in dichloromethane (20 mL). The solution was washed with $NaHCO_3$ (sat. aq., 10 mL), and the aqueous layer was extracted with dichloromethane (2 x 10 mL). The combined organic extracts were dried over anhydrous magnesium sulfate, filtered, and concentrated *in vacuo*. The crude residue was purified by flash column chromatography (silica gel, dichloromethane:ethyl acetate, 4:1) to afford the deprotected urea **19** (36 mg, 87%) as an off-white solid. δ_H (600 MHz, $CDCl_3$): 8.54-8.50 (1H, m, H26),

* Observed integration greater than predicted due to co-incidence with the residual $CHCl_3$ peak. HSQC analysis revealed this multiplet to contain 1 x 1H signal.

8.48 (1H, d, *J* 9.9, H11), 8.11 (1H, d, *J* 9.9, H12), 7.83-7.76 (3H, m, H23, H24 & H25), 7.36 (2H, app. d, *J* 7.2, H19), 7.31 (4H, app. t, *J* 7.5, H8 & H20), 7.26-7.21 (4H, m, H7, H9 & H21), 5.13 (1H, tt, *J* 8.8, 3.3, H4), 4.88 (1H, app. tt, *J* 8.4, 3.0, H16), 4.64 (1H, s, H1), 4.24 (1H, dd, *J* 11.0, 8.1, H15), 4.21 (1H, dd, *J* 11.0, 2.5, H15'), 3.50 (1H, t, *J* 8.9, H3), 3.42 (2H, app. td, *J* 13.1, 3.3, $H5_{RHS}$ & $H17_{LHS}$), 3.32 (1H, ddd, *J* 9.0, 3.1, 0.8, H3'), 3.14 (1H, dd, *J* 13.7, 8.8, H17'), 2.91 (1H, dd, *J* 13.4, 9.1, H5'); δ_C (151 MHz, $CDCl_3$): 158.2 (C2), 152.2 (C10), 151.0 (C14), 150.2 (C13), 148.1 (C27), 136.6 (C6), 135.2 (C23/C24/C25), 134.9(2) & 134.9(0) (C18 & C26), 132.2 (C23/C24/C25), 131.7 (C22), 129.8 (C19), 129.7 (C7), 129.0 & 128.8 (C8 & C20), 127.5 (C21), 127.0 (C9), 124.7 (C23/C24/C25), 120.2 (C11), 119.7 (C12), 55.9 (C16), 55.7 (C4), 46.5 (C15), 41.8 (C17), 41.7 (C3), 37.5 (C5); HRMS (ESI+): found 614.1826; $C_{30}H_{28}N_7O_6S$, $[M+H]^+$ requires 614.1822; v_{max} (thin film): 3428 (br), 2921, 1731 (br), 1542, 1440, 1172 cm^{-1}; $[\alpha]_D^{20}$ +137.6 (*c* = 1.14, $CHCl_3$).

(*S*)-4-Benzyl-3-(6-((*S*)-4-benzyl-3-((2-nitrophenyl)sulfonyl)-2-oxoimidazolidin-1-yl)pyridazin-3-yl)-1-(6-((*S*)-5-isopropyl-3-(6-((*S*)-5-isopropyl-3-(6-((*S*)-5-isopropyl-2-oxo-3-phenylimidazolidin-1-yl)pyrimidin-4-yl)-2-oxoimidazolidin-1-yl)pyrimidin-4-yl)-2-oxoimidazolidin-1-yl)pyridazin-3-yl)imidazolidin-2-one (20)

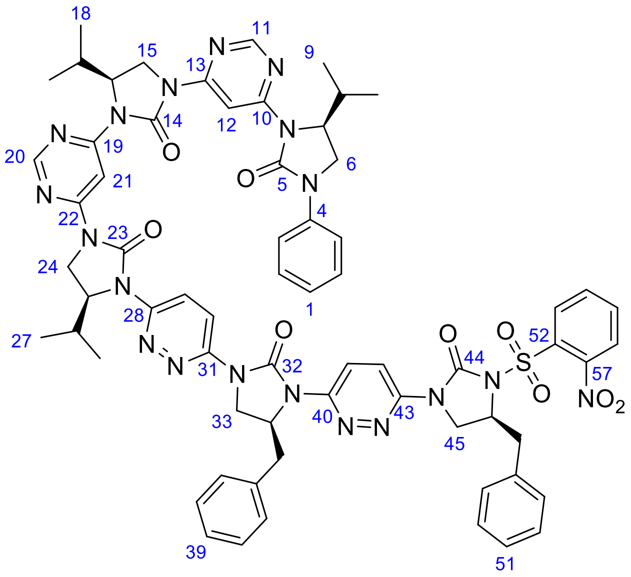

Prepared according to **General Procedure B** using deprotected urea **19** (20 mg, 32 μmol), bromopyridazine **17** (30 mg, 39 μmol), $Pd_2(dba)_3$ (2.0 mg, 2.2 μmol), Xantphos (4.0 mg, 6.9 μmol), toluene (0.5 mL), and Cs_2CO_3 (26 mg, 0.080 mmol). Reaction time = 16 h. Purification by flash column chromatography (silica gel, toluene:acetone, 10:1→7:1) afforded mixed pentamer **20** (21 mg, 50%) as a yellow glassy solid. δ_H (600 MHz, $CDCl_3$): 9.20 (1H, d, *J* 1.1, H12), 9.16 (1H, d, *J* 1.1, H21), 8.67 (1H, d, *J* 9.9, H29), 8.63 (1H, d, *J* 1.1, H20), 8.62 (1H, d, *J* 1.1, H11), 8.55-8.52 (1H, m, H56), 8.48 (1H, d, *J* 9.9, H41), 8.38 (1H, d, *J* 10.0, H30), 8.20 (1H, d, *J* 10.0, H42), 7.84-7.77 (3H, m, H53-H55), 7.64 (1H, dd, *J* 8.7, 1.0, H3), 7.42-7.36 (4H, m, H2 & 2 x Ar CH), 7.32 (2H, t, *J* 7.6, H38/H50), 7.28-7.24 (8H, m, 3 x Ar CH)*, 7.23-7.19 (3H, m, 3 x Ar CH), 7.13 (1H, tt, *J* 7.4, 0.9, H1), 5.21 (1H, tt, *J* 8.5, 3.5, H34), 4.95-4.88 (2H, m, $H25_{LHS}$ & $H46_{RHS}$), 4.79 (2H, app. dq, *J* 9.6, 3.2, $H7_{RHS}$ & $H16_{LHS}$), 4.29-4.25 (2H, m, H45 & H45'), 4.22 (1H, dd, *J* 11.0, 3.4, H33), 4.15 (1H, dd, *J* 11.1, 8.7, H33'), 4.09 (2H, app. td, *J* 11.0, 3.4, $H15_{LHS}$ & $H24_{RHS}$), 4.04 (1H, dd, *J* 11.0, 9.3, H24'), 4.00-3.95 (2H, m, $H6_{LHS}$ & $H15'_{RHS}$), 3.66 (1H, dd, *J* 9.1, 2.9, H6'), 3.47-3.42 (2H, m, H35 & H47), 3.17 (1H, dd, *J* 13.8, 8.7, H47'), 3.00 (1H, dd, *J* 13.6, 8.4, H35'), 2.74 -2.67 (1H, m, H26), 2.67-2.57 (2H, m, $H8_{LHS}$ & $H17_{RHS}$), 1.03 (9H, app. t, *J* 7.0, H9, H18 & H27), 0.85 (3H, d, *J* 6.9, H9'), 0.82 (6H, app. dd, J 6.9, 3.0, $H18'_{LHS}$ & $H27'_{RHS}$); δ_C (151 MHz, $CDCl_3$) 158.2, 158.0, 157.7(1) & 157.6(8) (C10, C13, C19 & C22), 157.0 & 156.9 (C11 & C20), 153.8 (C5), 153.6, 153.5 & 153.2 (C14, C23 & C32), 151.7 (C43), 151.4 (C31), 151.3 (C28), 150.9 (C44), 150.6 (C40), 148.0 (C57), 139.2 (C4), 135.7 (ArC), 135.2 (C54/C55), 134.7(9) (C56),134.7(5) (ArC), 132.1 (C54/C55), 131.6 (ArC), 129.7 (Ar CH), 129.5 (Ar CH), 129.0 (Ar CH), 128.9 (Ar CH), 128.7 (Ar CH), 127.4 (Ar CH), 127.1 (Ar CH), 124.6 (C53), 123.8 (C1), 121.1 (C29), 120.6 (C41), 119.7 & 118.8 (C30 & C42), 118.8 (C3), 97.7 (C21), 97.4 (C12), 55.8 (C46), 55.5(1), 55.4(9) & 55.4(7) (C7, C16 & C25), 52.5

* Observed integration greater than predicted due to co-incidence with the residual $CHCl_3$ peak. HSQC analysis revealed this multiplet to contain 1 x 2H and 1 x 1H signals.

(C34), 46.4 (C45), 45.7 (C33), 43.0 (C6), 41.7 (C47), 41.4 & 41.3 (C15 & C24), 38.0 (C35), 31.9 (C26), 28.3(8) & 28.3(6) (C8 & C17), 18.2, 18.1(3) & 18.0(9) (C9, C18 & C27), 14.3, 14.2 & 14.0 (C9', C18' & C27'); HRMS (ESI+): found 1302.5188; $C_{66}H_{68}N_{19}O_9S$, $[M+H]^+$ requires 1302.5168; v_{max} (thin film): 2961, 2928, 1726, 1582, 1543, 1390, 1241 cm^{-1}; $[\alpha]_D^{20}$ −0.4 (c = 0.55, $CHCl_3$).

(*S*)-4-Benzyl-1-(5-((*S*)-5-benzyl-2-oxo-3-phenylimidazolidin-1-yl)pyrazin-2-yl)-3-(6-((*S*)-4-benzyl-3-((2-nitrophenyl)sulfonyl)-2-oxoimidazolidin-1-yl)pyridazin-3-yl)imidazolidin-2-one (21)

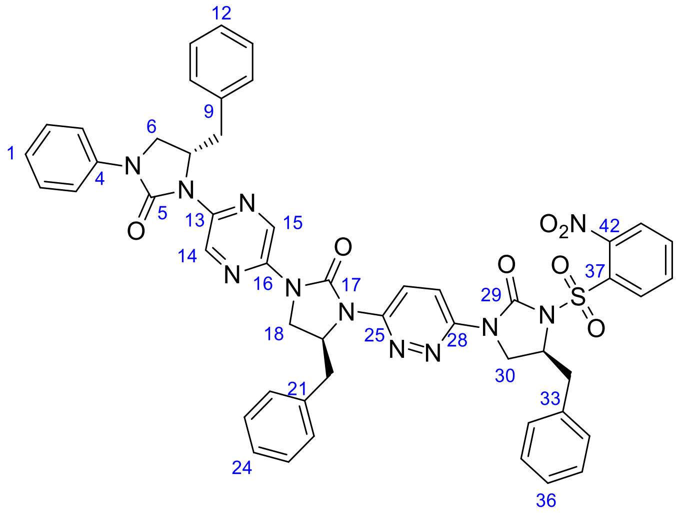

Prepared according to **General Procedure B** using deprotected urea **S7** (18 mg, 0.036 mmol), bromopyridazine **4** (20 mg, 0.039 mmol), $Pd_2(dba)_3$ (1.6 mg, 1.7 μmol), Xantphos (3.1 mg, 5.4 μmol), toluene (0.5 mL), and Cs_2CO_3 (29 mg, 0.089 mmol). Reaction time = 16 h. Purification by flash column chromatography (silica gel, dichloromethane:ethyl acetate, 19:1) afforded pyridazine-pyrazine trimer **21** (28 mg, 83%) as a white solid. δ_H (600 MHz, $CDCl_3$): 9.27 (1H, d, J 1.6, H14), 9.12 (1H, d, J 1.6, H15), 8.56-8.52 (2H, m, H26 & H41), 8.21 (1H, d, J 9.9, H27), 7.85-7.78 (3H, m, H38, H39 & H40), 7.48 (2H, dd, J 8.8, 1.1, H3), 7.40 (2H, d, J 7.0, 2 x Ar CH), 7.38-7.27 (9H, m, 9 x Ar CH), 7.26-7.20 (6H, m, 6 x Ar CH), 7.12 (1H, tt, J 7.3, 1.0, H1), 5.24 (1H, tt, J 8.4, 3.2, H19), 4.99 (1H, tt, J 9.1, 3.2, H7), 4.92 (1H, ddt, J 8.6, 7.2, 3.5, H31), 4.30-4.23 (2H, m, H30 & H30'), 4.09 (1H, dd, J 10.9, 8.4, H18), 4.04 (1H, dd, J 10.9, 3.1, H18'), 3.96 (1H, t, J 8.7, H6), 3.68 (1H, dd, J 9.2, 3.3, H6'), 3.45 (1H, dd, J 13.7, 3.5, H32) 3.41-3.35 (2H, m, $H20_{LHS}$, $H8_{RHS}$), 3.18 (1H, dd, J 14.0, 8.8, H32'), 3.07 (1H, dd, J 13.6, 8.1, H20'), 2.76 (1H, dd, J 13.5, 9.6, H8'); δ_C (151 MHz, $CDCl_3$): 153.9 (C5), 153.5 (C17), 151.8 (C25/C28), 151.0, 150.5 (C25/C28), 148.0, 143.8 (C13/C16), 143.1 (C13/C16), 139.4 (C4), 136.5, 135.8, 135.2, 134.8(0), 134.7(7), 133.7 (C14), 132.7 (C15), 132.1, 131.6, 129.7, 129.5, 129.4, 129.0, 128.9, 128.8, 128.7, 127.4, 127.1 (C10), 127.0, 124.6, 123.7 (C1), 120.3 (C26), 119.7 (C27), 118.6 (C3), 55.9 (C31), 52.7 (C19), 52.3 (C7), 47.2 (C6), 46.4 (C30), 45.2 (C18), 41.7 (C32), 38.4 (C8), 37.9 (C20); HRMS (ESI+): found 942.3124; $C_{50}H_{44}N_{11}O_7S$, $[M+H]^+$ requires 942.3146; v_{max} (thin film): 2924, 1739, 1714, 1442, 1403, 1375 cm^{-1}; $[\alpha]_D^{20}$ +23.0 (c = 0.30, $CHCl_3$).

(*S*)-4-Benzyl-1-(6-((*S*)-5-isopropyl-3-(6-((*S*)-5-isopropyl-2-oxo-3-phenylimidazolidin-1-yl)pyrimidin-4-yl)-2-oxoimidazolidin-1-yl)pyridazin-3-yl)-3-((2-nitrophenyl)sulfonyl)imidazolidin-2-one (22)

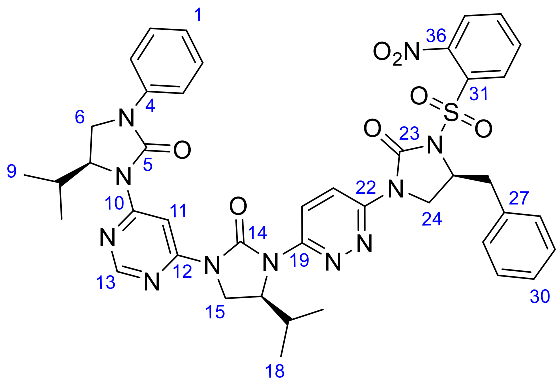

Prepared according to **General Procedure B** using deprotected urea **S5** (21 mg, 0.052 mmol), bromopyridazine **4** (29 mg, 0.057 mmol), $Pd_2(dba)_3$ (2.3 mg, 2.5 μmol), Xantphos (4.5 mg, 7.8 μmol), toluene (0.5 mL), and Cs_2CO_3 (42 mg, 0.13 mmol). Reaction time = 16 h. Purification by flash column chromatography (silica gel, dichloromethane:ethyl acetate, 10:1→5:1) afforded pyrimidine-pyridazine trimer **22** (25 mg, 57%) as a pale yellow glassy solid. δ_H (600 MHz, $CDCl_3$): 9.24 (1H, d, *J* 1.2, H11), 8.62 (1H, d, *J* 10.0, H20), 8.61 (1H, d, *J* 1.2, H13), 8.54-8.51 (1H, m, H35), 8.15 (1H, d, *J* 10.0, H21), 7.84-7.80 (2H, m, $H33_{RHS}$ & $H34_{LHS}$), 7.80-7.75 (1H, m, H32), 7.65-7.63 (2H, m, H3), 7.39-7.33 (4H, m, $H2_{LHS}$ & $H28_{RHS}$), 7.30 (2H, t, *J* 7.6, H29), 7.23 (1H, tt, *J* 7.3, 1.3, H30), 7.12 (1H, tt, *J* 7.4, 1.0, H1), 4.91-4.85 (2H, m, $H16_{LHS}$ & $H25_{RHS}$), 4.78 (1H, ddd, *J* 9.5, 3.4, 2.9, H7), 4.22 (1H, dd, *J* 11.0, 2.9, H24), 4.19 (1H, dd, *J* 11.0, 7.9, H24’), 4.08 (1H, dd, *J* 11.1, 3.8, H15), 4.02 (1H, dd, *J* 11.1, 9.6, H15’), 3.97 (1H, t, *J* 9.4, H6), 3.65 (1H, dd, *J* 9.4, 2.7, H6’), 3.45 (1H, dd, *J* 13.7, 3.5, H26), 3.11 (1H, dd, *J* 13.7, 9.0, H26’), 2.70-2.60 (2H, m, $H8_{RHS}$ & $H17_{LHS}$), 1.04-1.01 (6H, m, $H9_{RHS}$ & $H18_{LHS}$), 0.84 (3H, d, *J* 6.9, H9’), 0.80 (3H, d, *J* 6.9, H18’); δ_C (151 MHz, $CDCl_3$): 158.1 & 157.6 (C10 & C12), 157.1 (C13), 154.0 (C5), 153.7 (C14), 151.7 (C19), 150.9 (C23), 150.5 (C22), 148.0 (C36), 139.1 (C4), 135.2 (C33), 134.8(4) & 134.8(1) (C27 & C35), 132.2 (C34), 131.6 (C31), 129.7 (C28), 128.9 (C2), 128.8 (C29), 127.4 (C30), 124.6 (C32), 123.8 (C1), 121.1 (C20), 119.5 (C21), 118.7 (C3), 96.6 (C11), 55.8 (C25), 55.5 (C16), 55.3 (C7), 46.4 (C24), 42.9 (C6), 41.7 (C26), 41.3 (C15), 28.4 (C8), 27.3 (C17), 18.2 & 18.0 (C9 & C18), 14.2 (C9’), 13.9 (C18’); HRMS (ESI+): found 846.3143; $C_{42}H_{44}N_{11}O_7S$, $[M+H]^+$ requires 846.3146; ν_{max} (thin film): 2956, 1720, 1580, 1388, 1254, 757 cm^{-1}; $[\alpha]_D^{20}$ +9.4 (*c* = 0.50, $CHCl_3$).

Solution-Phase Conformational Analysis by NMR

All NOESY and ROESY data were acquired on a Bruker 600 MHz spectrometer with a mixing time (t_{mix}) of 200 ms. Unless otherwise stated, spectra were acquired in $CDCl_3$ at room temperature. Full NOESY/ROESY spectra are given, followed by a zoomed view of the cross-peak region of greatest interest. Integrations were carried out manually. Where coloured bands are placed over spectra and with alternating red/blue colouration, this is done to aid visualization and the colours are arbitrary (unless otherwise stated). Since many of the signals examined are very weak, bands of noise are frequently present at similar levels of intensity. Where these can be identified they are marked on spectra with a green coloured band.

Pyrimidine Dimer 10

ROESY, $CDCl_3$, 600 MHz, t_{mix} = 0.2 s

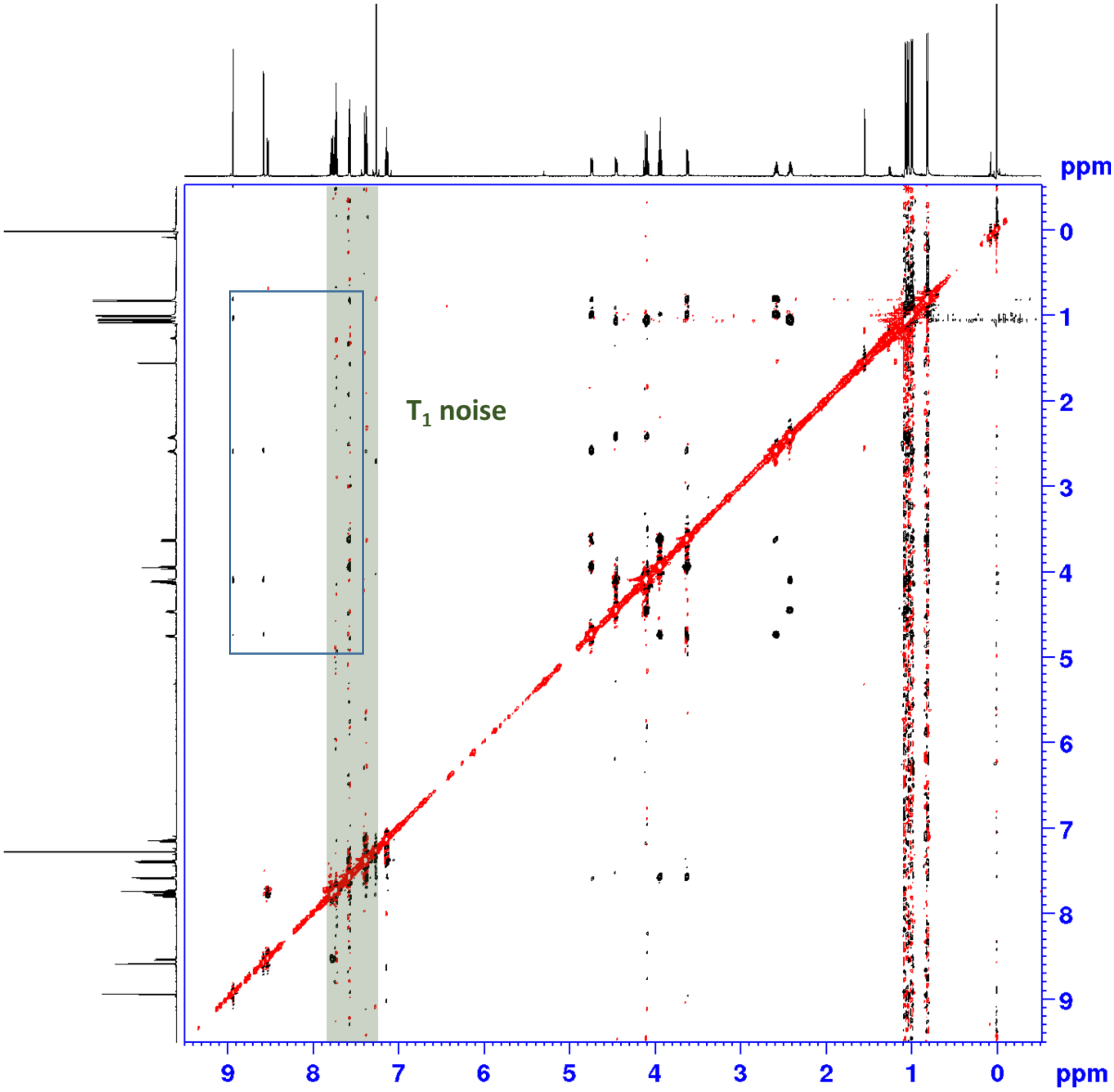

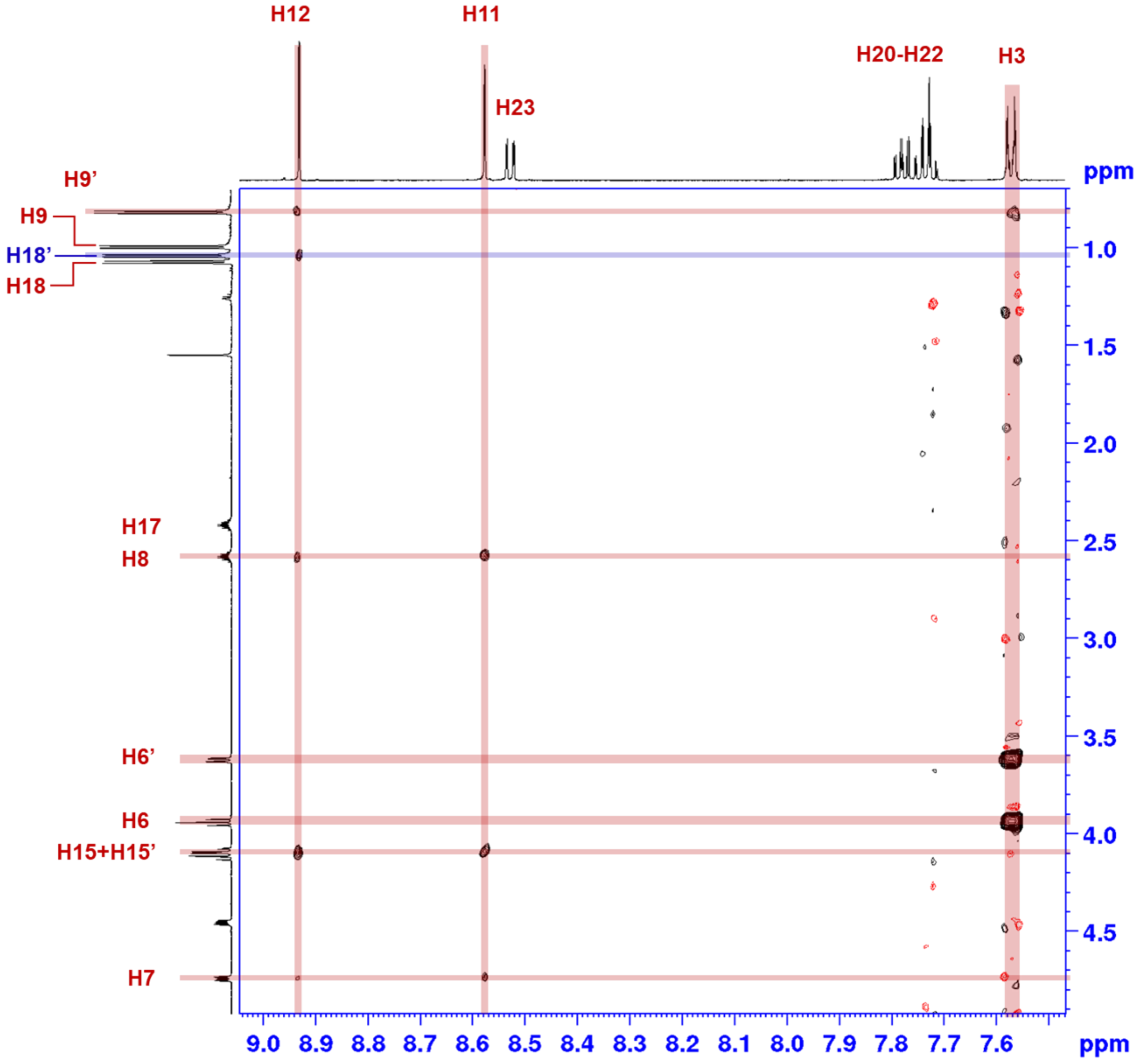

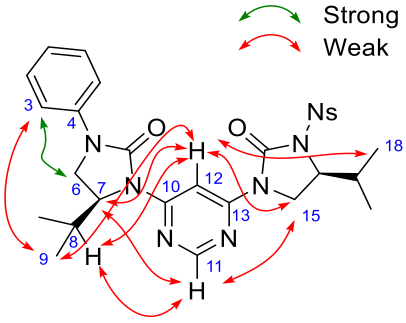

Table S1. Selected ranked nOe cross-peak intensities for dimer **10** in CDCl3.

Peak	Normalized Intensity
H3 - H6'	1.000
H3 - H6	0.949
H12 - [H15+H15'][a]	0.079
H3 - H9'	0.069
H8 - H11	0.043
H12 - H18'	0.033
H7 - H11	0.029
H8 - H12	0.027
H9' - H12	0.027
H11 - [H15+H15'][a]	0.026
H7 - H12	0.016

[a] Peaks isochronous.

The strong nOe between H3 and H6' was used as an internal standard for integration of peaks. Relative to this and H3↔H6, the H12↔[H15+H15'] resonance was the next strongest peak, with an intensity ~8% of H3↔H6'. Due to H15

and H15’ being isochronous, the true integral per hydrogen atom is likely closer to 0.04. The presence of cross-peaks along the ‘outer edge’ of the molecule (H7↔H11, H11↔H15) provide evidence that the conformation adopted is as depicted, since these hydrogens would be distant in alternative conformations where the pyrimidine and urea dipoles are aligned. As noted in the main text, the combined intensities of the H3↔H6 and H3↔H6’ resonances would be expected to have an intensity ratio with the combined H12↔H15 and H12↔H15’ resonances of 2:1 in an unbiased system. In $CDCl_3$, this ratio is 25:1, indicating the presence of a strong conformational biasing effect.

Comparison of this data to the data obtained by X-ray crystallography is given in the main text.

In addition to the above studies in $CDCl_3$ (dielectric constant ε 4.8), the conformation of **10** was examined in the much more polar d_6-DMSO (ε 46.7), and at elevated temperature, to determine whether the dipole repulsion-mediated conformational control would be retained.

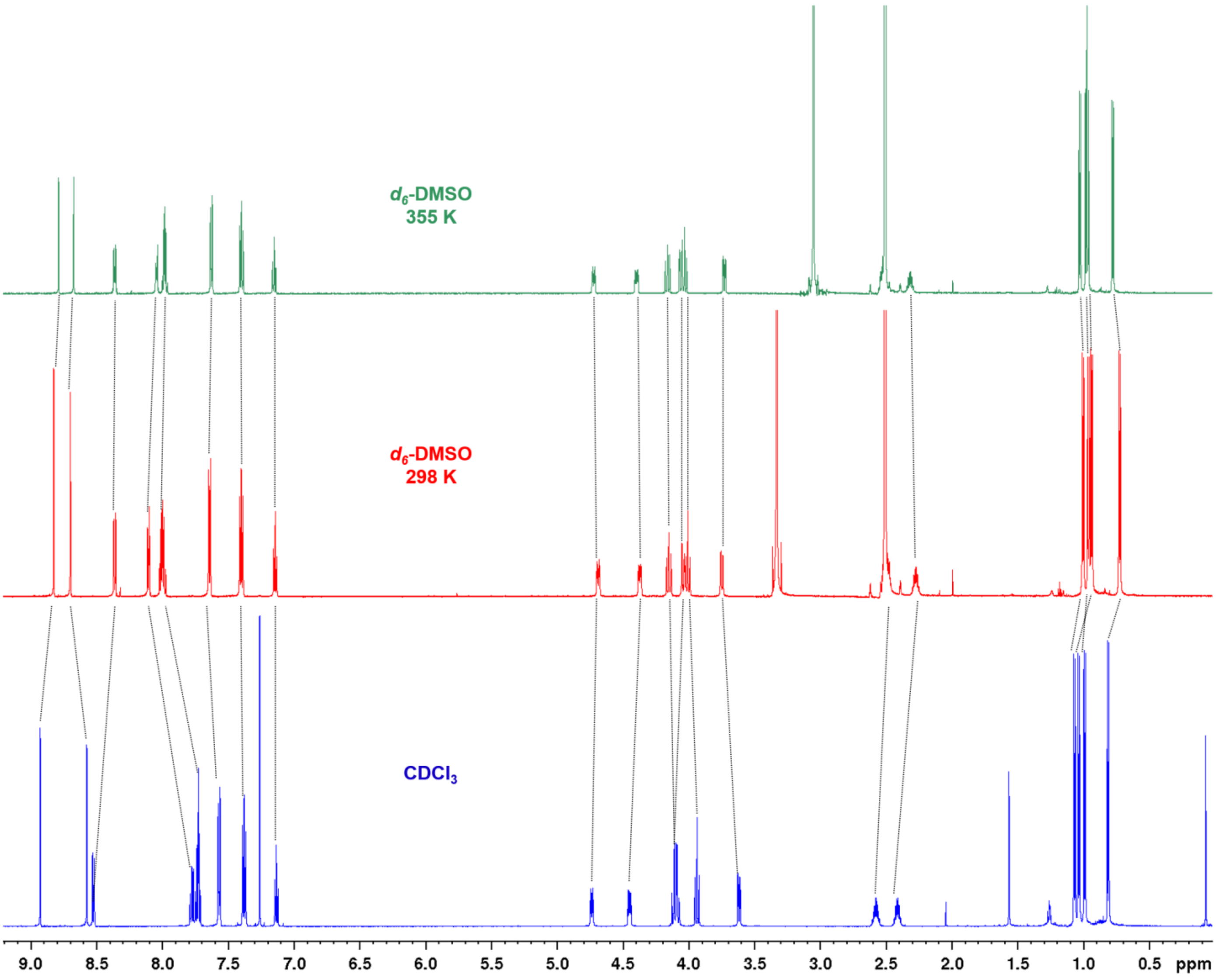

ROESY, d_6-DMSO, 600 MHz, t_{mix} = 0.2 s.

298 K

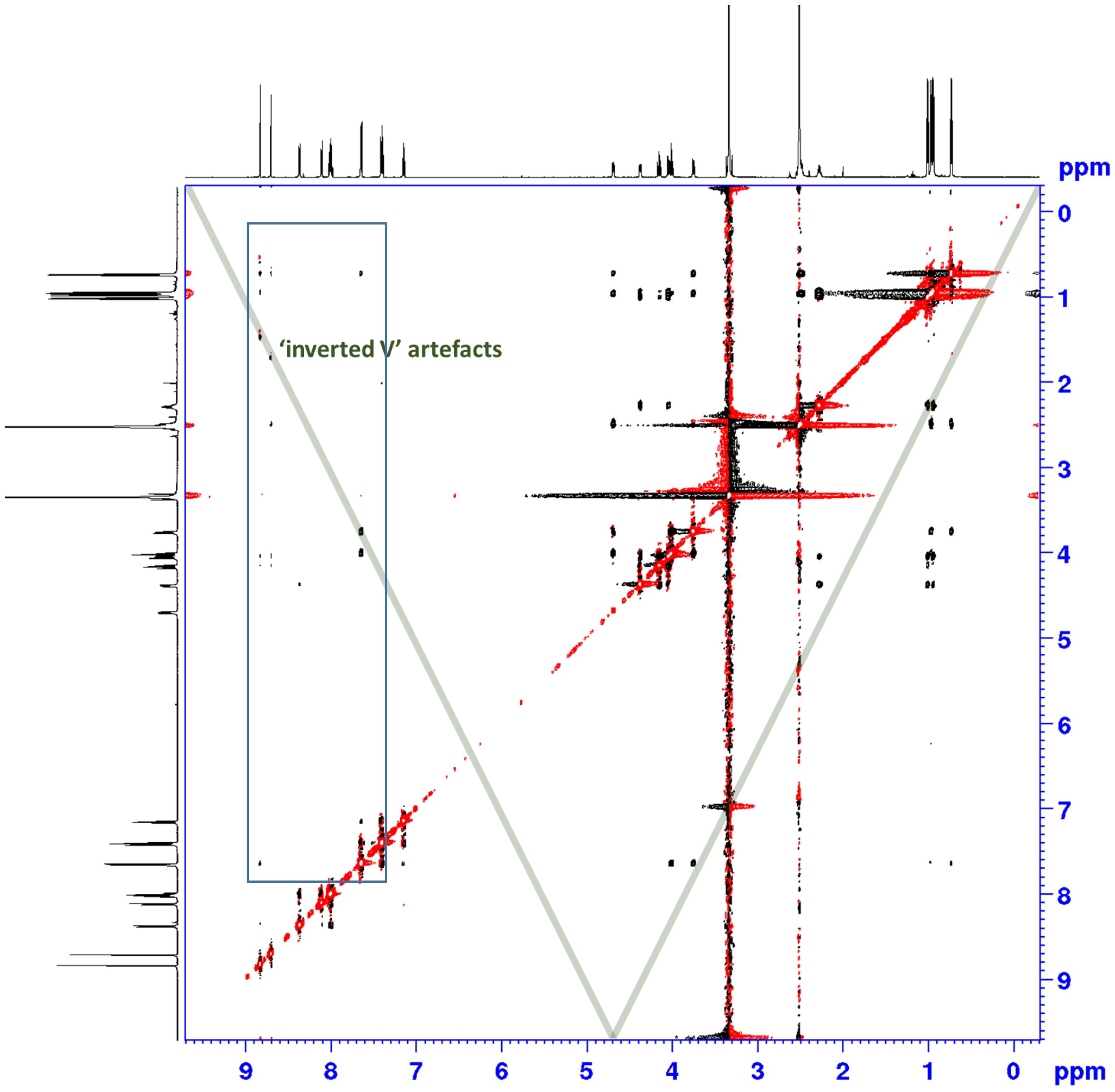

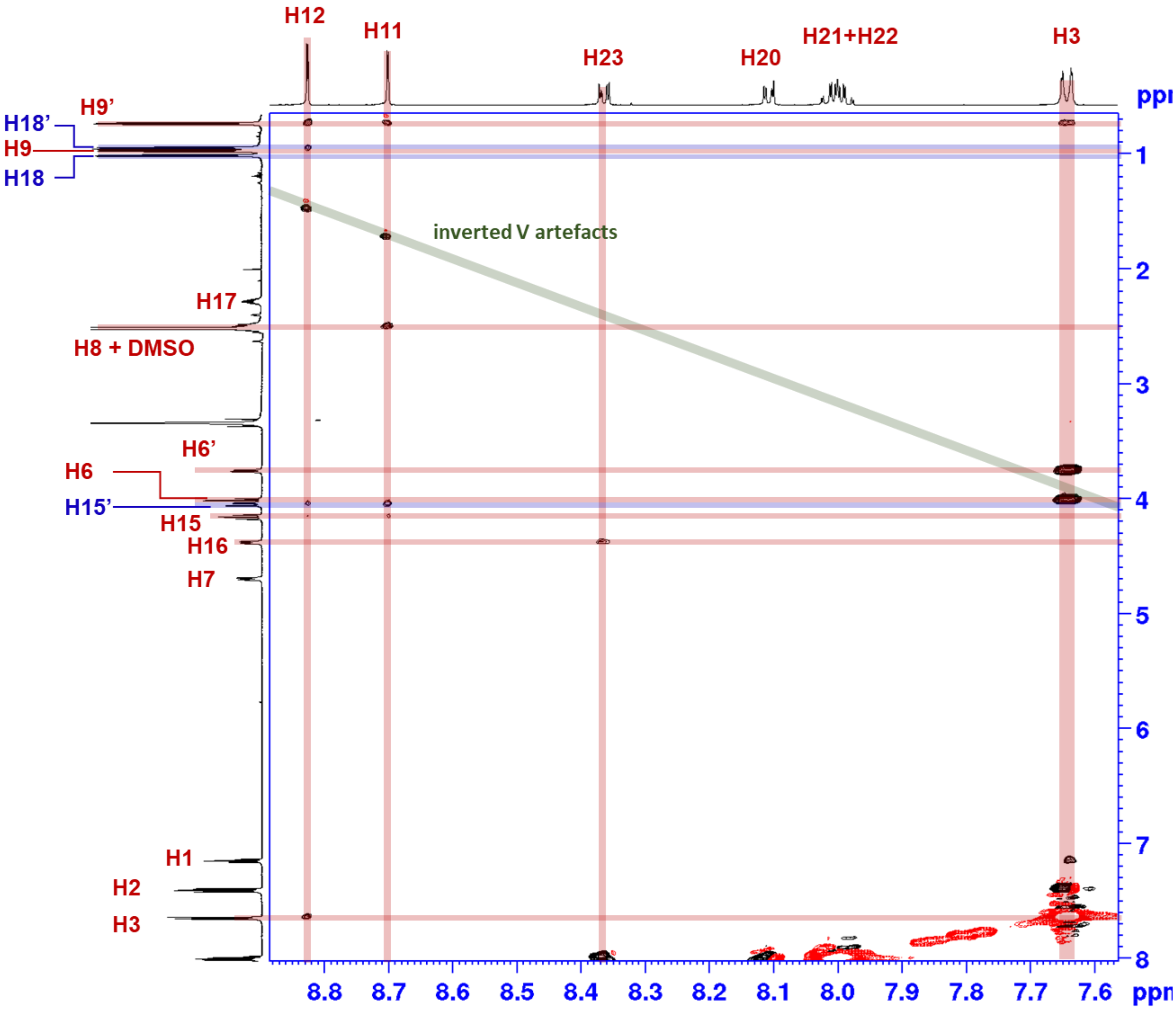

H12
H11
H23
H20
H21+H22
H3
H9'
H18'
H9
H18
inverted V artefacts
H17
H8 + DMSO
H6'
H6
H15'
H15
H16
H7
H1
H2
H3
8.8
8.7
8.6
8.5
8.4
8.3
8.2
8.1
8.0
7.9
7.8
7.7
7.6
ppm
1
2
3
4
5
6
7
8

355 K

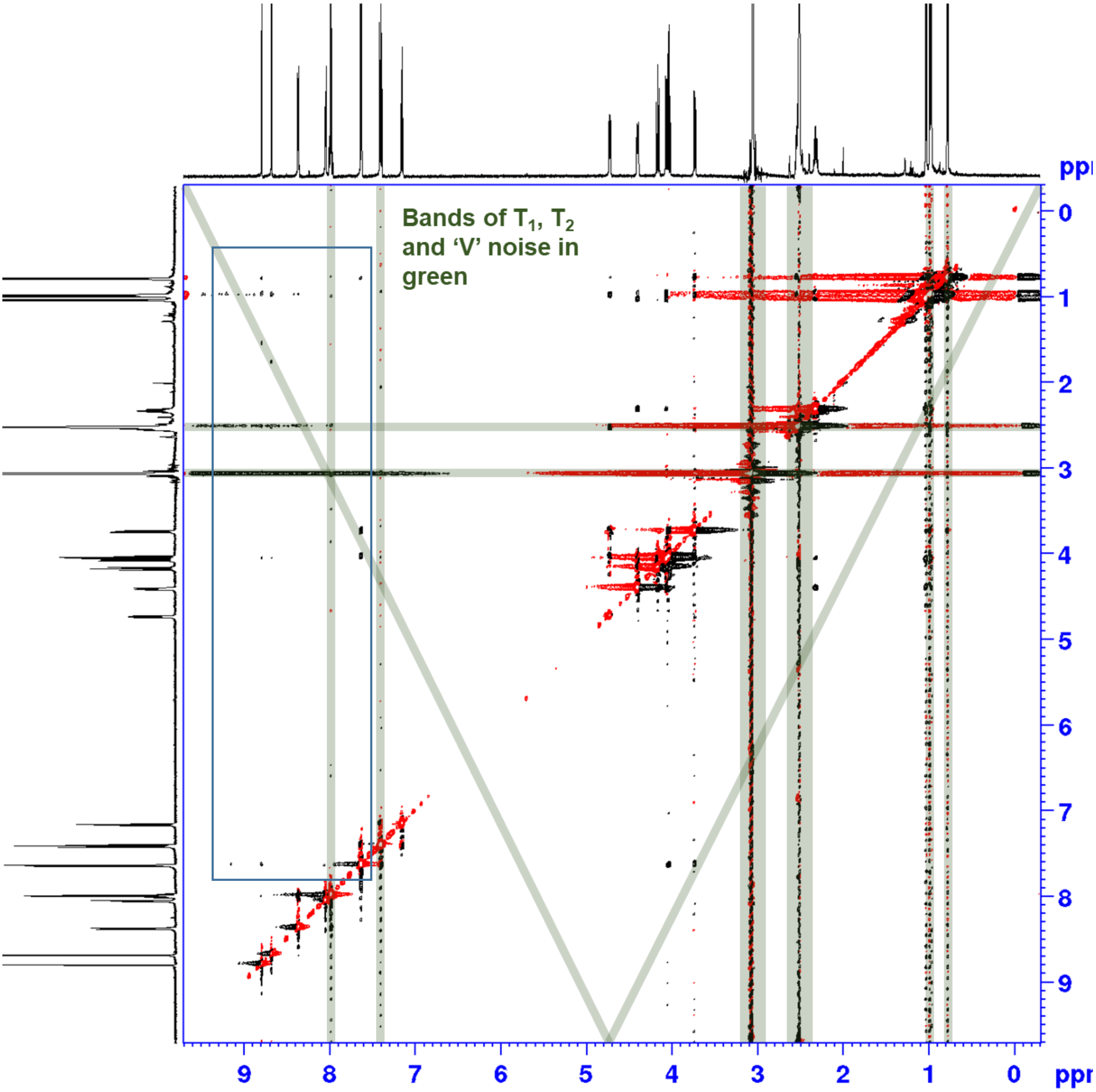
Bands of T_1, T_2 and ‘V’ noise in green
ppm
0
1
2
3
4
5
6
7
8
9
9
8
7
6
5
4
3
2
1
0
ppm

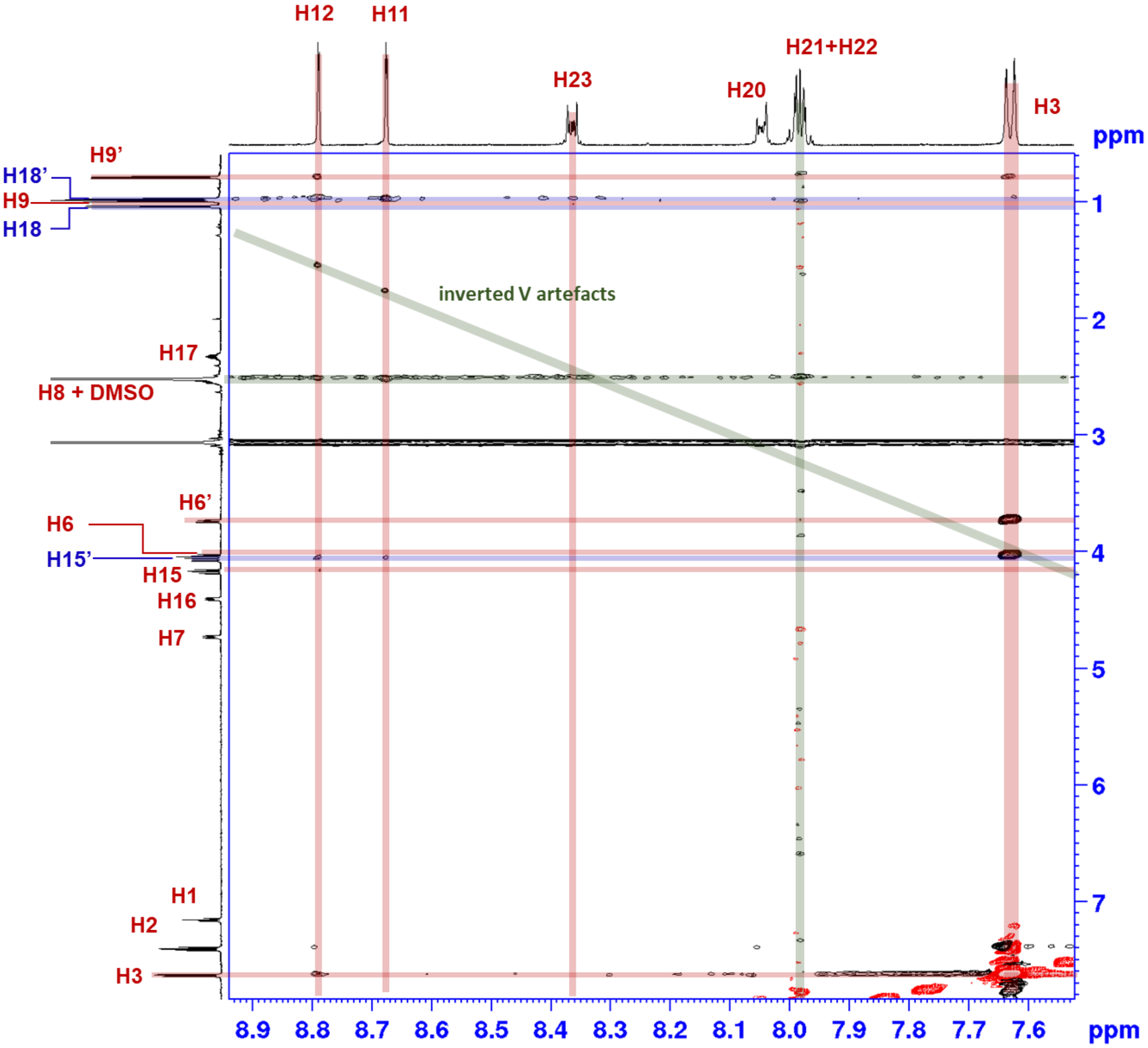

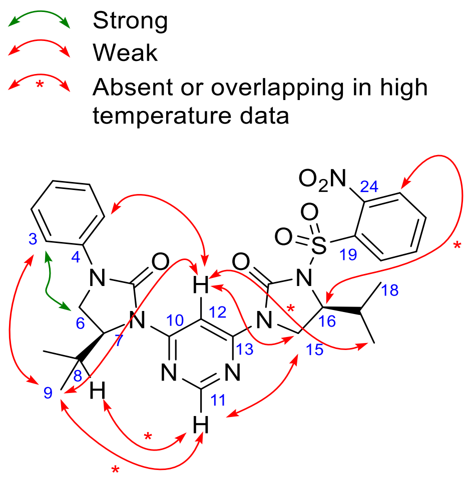

Table S2. Selected ranked nOe cross-peak intensities for dimer **10** in d_6-DMSO.

Peak	Normalized Intensity	
	298 K	355 K
H3 - H6'	1.000	1.000
H3 - H6'	0.888	0.992
H16 - H23	0.084	-[a]
H8 - H11	0.079	-[b]
H3 - H9'	0.079	0.175
H9' - H12	0.062	0.165
H11 - H15'	0.043	0.111
H12 - H18'	0.040	-[b]
H9' - H11	0.039	-[a]
H3 - H12	0.036	0.185
H12 - H15	0.023	0.112
H12 - H15'	0.023	0.070

[a] Not observed; [b] value not given due to coincidence of peak with a strong band of noise, preventing accurate integration.

As with the $CDCl_3$ data, the RT d_6-DMSO are largely consistent with the dipole-opposed conformation about the *N*-$C_{pyrimidine}$ bonds, in particular due to the H8↔H11, H3↔H12 and H11↔H15 resonances, all of which would not be expected to occur in the alternative, dipole-aligned conformers. The absence of H7↔H11 and H7↔H12 resonances is unexpected, but inspection of the data shows that there are no signals to H7 across the entire F2 spectral range, suggesting the reason for this is experimental, rather than conformational. When the temperature was increased to 355 K, all observable signals were strengthened relative to H3↔H6, consistent with an overall thermal increase in conformational flexibility. However, the fact that H3↔H12 is now the third most intense signal in this region of the spectrum strongly suggests that the molecule remains biased towards the dipole-opposed conformation at this temperature. As noted in the main text, the combined intensities of the H3↔H6 and H3↔H6' resonances would be expected to have an intensity ratio with the combined H12↔H15 and H12↔H15 resonances of 2:1 in an unbiased system. In RT DMSO, this ratio is 41:1, indicating the presence of a strong conformational biasing effect. At 355 K this value dropped to 11:1, indicating increased rotation about the N-C13 bond, but the retention of some of the dipolar bias even at this temperature.

Pyridazine Dimer 11

ROESY, $CDCl_3$, 600 MHz, t_{mix} = 0.2 s

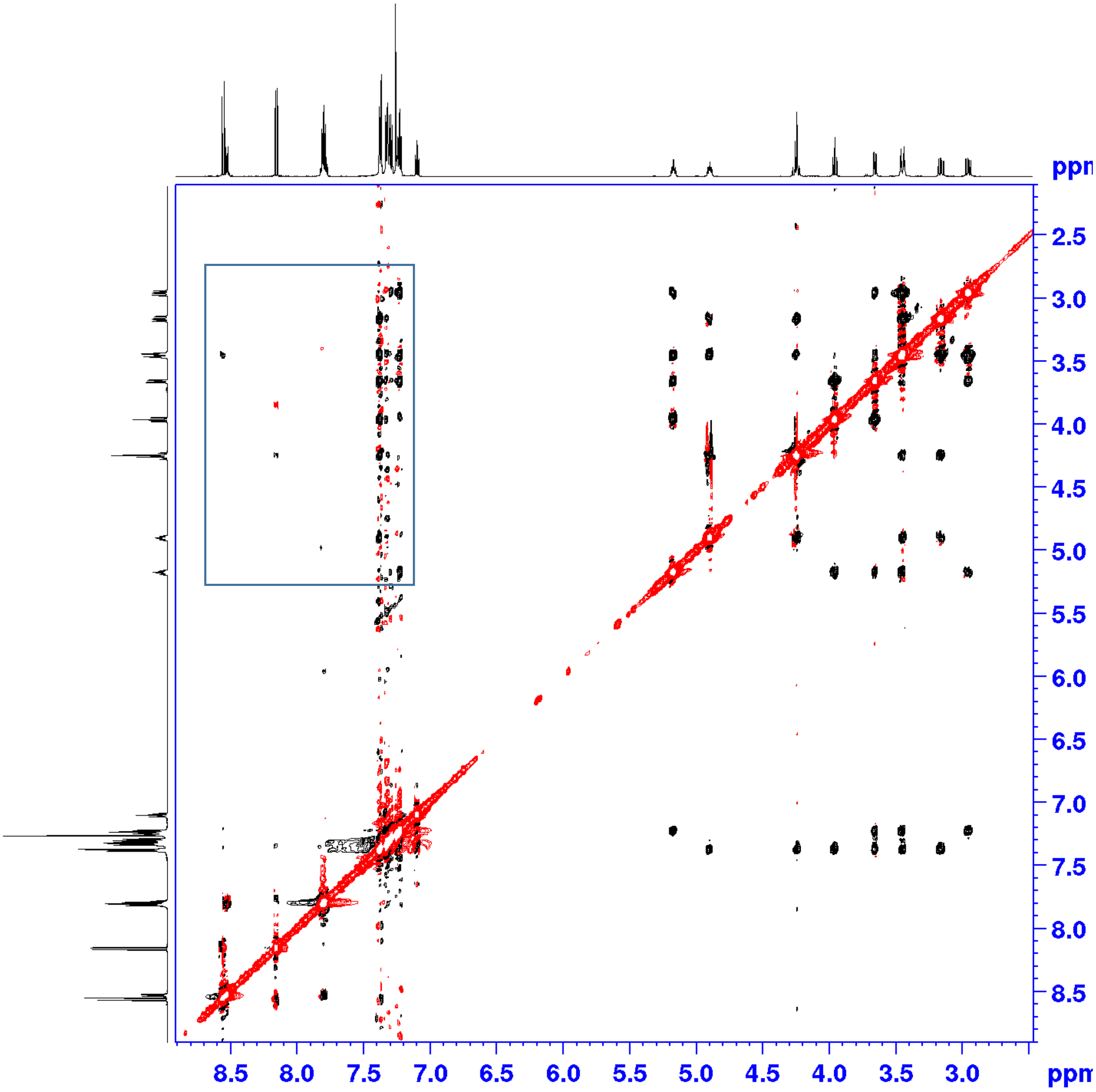

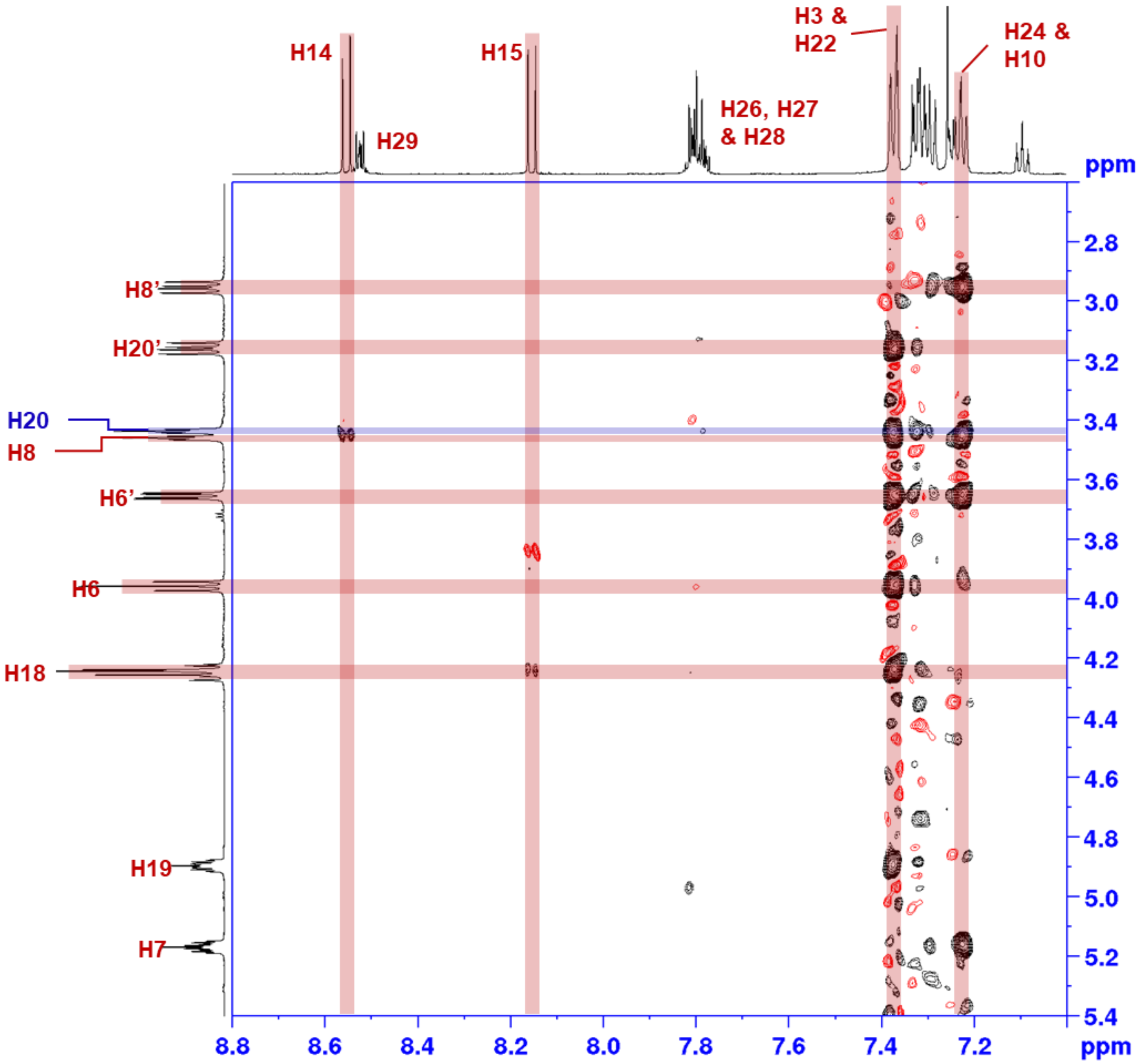

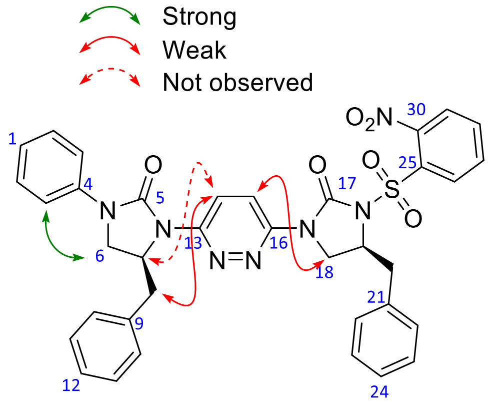

Table S3. Selected ranked nOe cross-peak intensities for dimer **11**

Peak	Normalized Intensity
H3 - H6	1.000
H3 - H6'	0.899
H8 - H14	0.070
H15 – [H18+H18'][a]	0.066

[a] Peaks isochronous

The strong nOe between H3 and H6 was used as an internal standard for integration of peaks. In addition to H3↔H6', the only other relevant cross-peaks observed were H8↔H14 and H15↔[H18+H18'], both of which were observed at ~7% the intensity of H3↔H6. The weakness of these resonances, and the fact that H7↔H14 is not observed at all, is in agreement with the proposed, dipole-opposed conformation. If the dipole mediated conformational biasing were entirely absent, it would be expected that the ratio of H15↔[H18+H18'] to (H3↔H6 + H3↔H6') would be 1:2 – the observed value is 1:29.

Pyrazine Dimer 12

ROESY, $CDCl_3$, 600 MHz, t_{mix} = 0.2 s

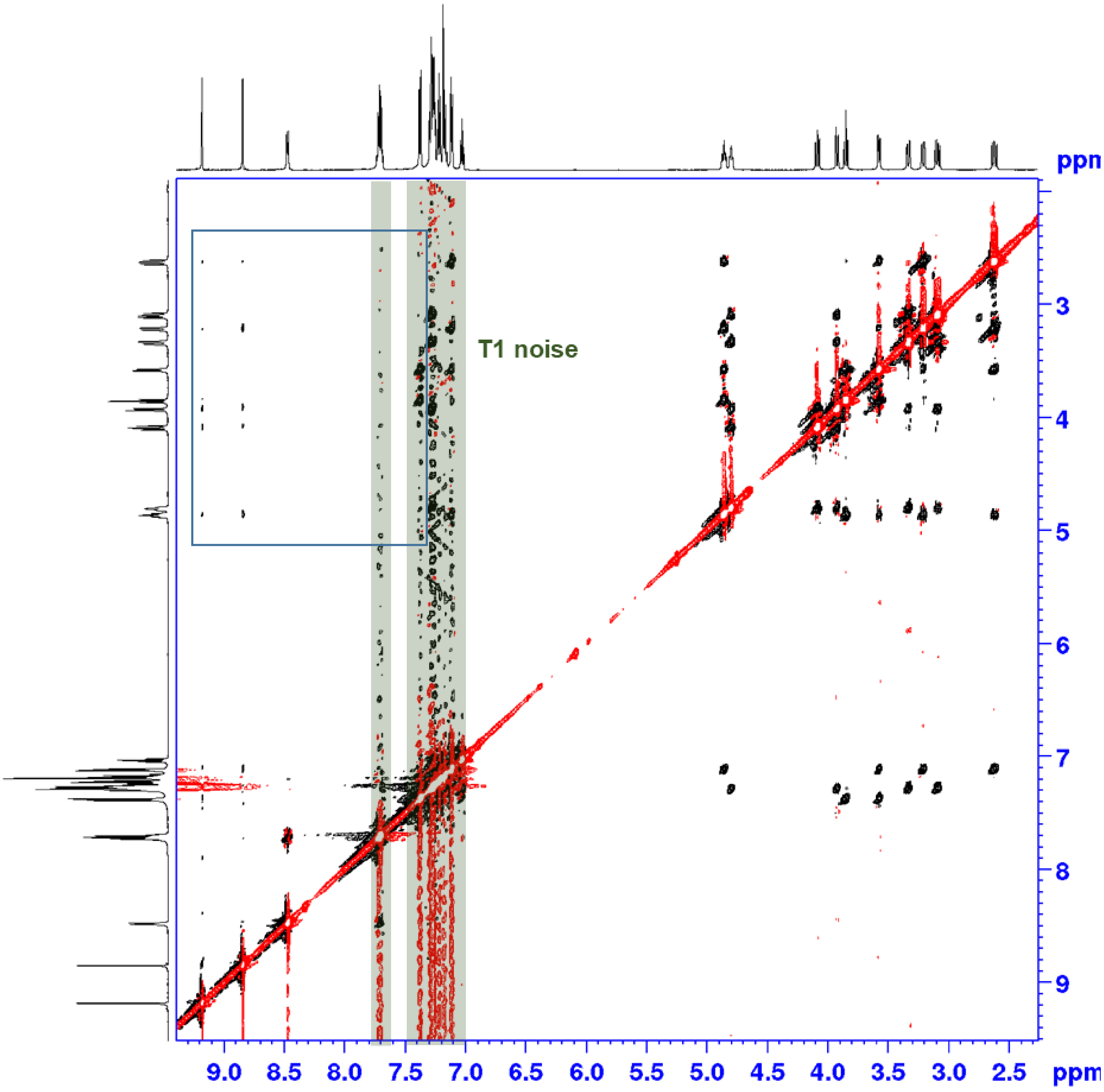

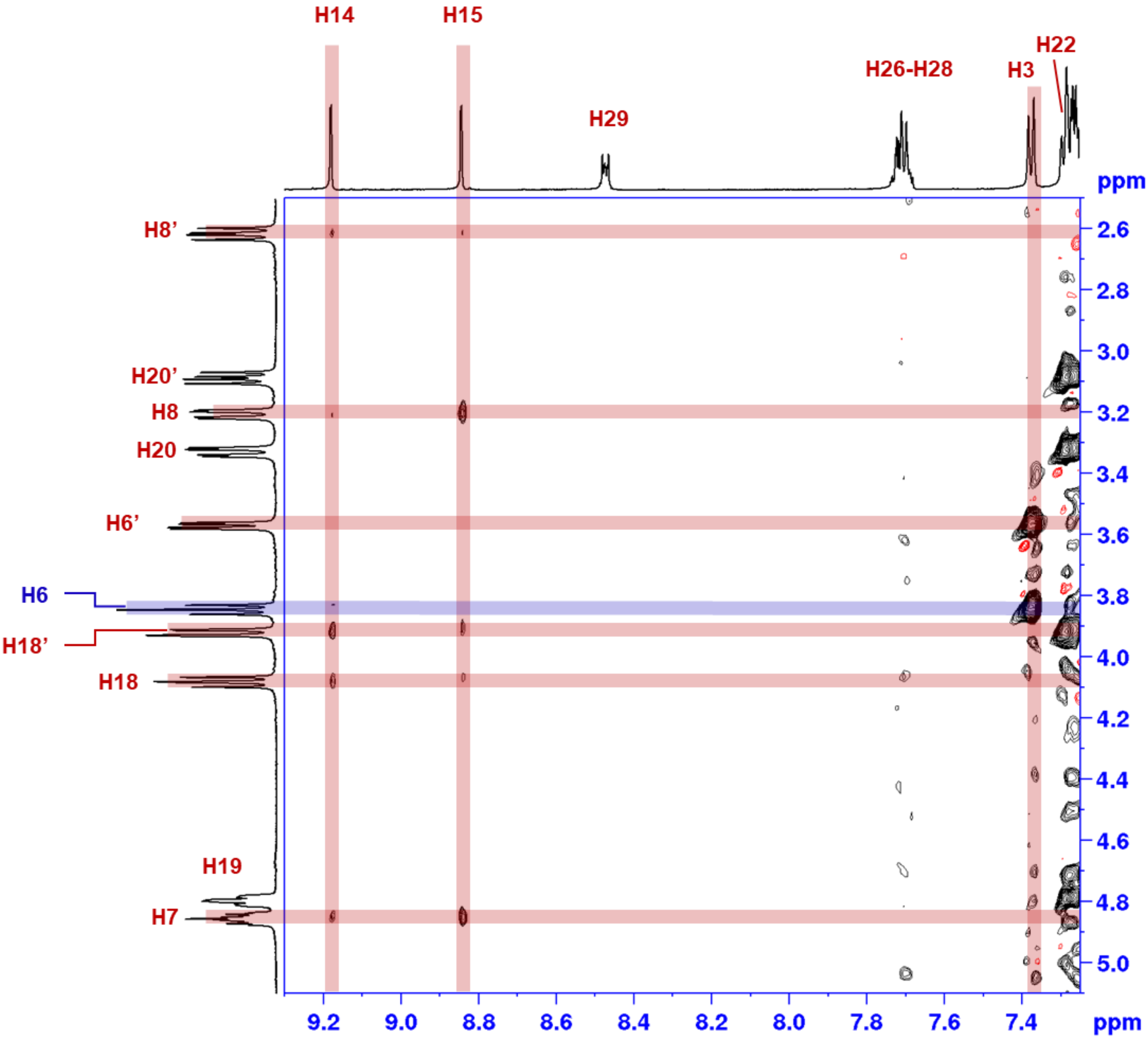

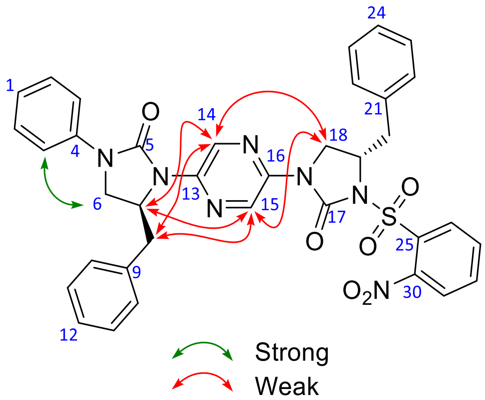

Table S4. Selected ranked nOe cross-peak intensities for dimer **12**

Peak	Normalized Intensity
H3 - H6	1.000
H3 - H6'	0.936
H8 - H15	0.055
H7 - H14	0.052
H14 - H18'	0.037
H14 - H18	0.031
H15 - H18'	0.026
H7 - H15	0.023
H15 - H18	0.022
H8' - H14	0.019
H8' - H15	0.018

The strong nOe between H3 and H6 was used as an internal standard for integration of peaks. In the dipole opposed conformation, the distances between imidazolidine-2-one CH's (e.g. H7) and the two peaks on the arene (H14, H15)

are close in value; as a result, weak nOes are generally observed between the imidazolidin-2-one and arene CH's with similar intensity (e.g. H14↔H18' = 0.037; H15↔H18' = 0.026). If the alternative conformation were adopted (with *ortho*-arene nitrogens *syn* with the adjacent carbonyl) we would expect e.g. H15↔H18' >> H14↔H18'. The fact that H7↔H14 is stronger than H7↔H15 does not fully agree with the corresponding distances from the X-ray structure (4.6 Å and 4.1 Å respectively), suggesting there may be a small degree of rotation around the *N*-C13 bond placing H7 in occasional close proximity to H14. However, the overall weakness of H7↔H14 compared to H3↔H6 is still consistent with the depicted, dipole opposed conformation being predominant in solution. If the dipole mediated conformational biasing were absent, it would be expected that the ratio of (H15↔H18 + H15↔H18') to (H3↔H6 + H3↔H6') would be 1:2 – the observed value is 1:40. This is in good agreement with the ratios obtained for the pyrimidine (1:25) and pyridazine (1:29) dimers, suggesting the conformational biasing is of similar strength across all three heterocycles.

Pyrimidine Trimer 13

ROESY, $CDCl_3$, 600 MHz, t_{mix} = 0.2 s.

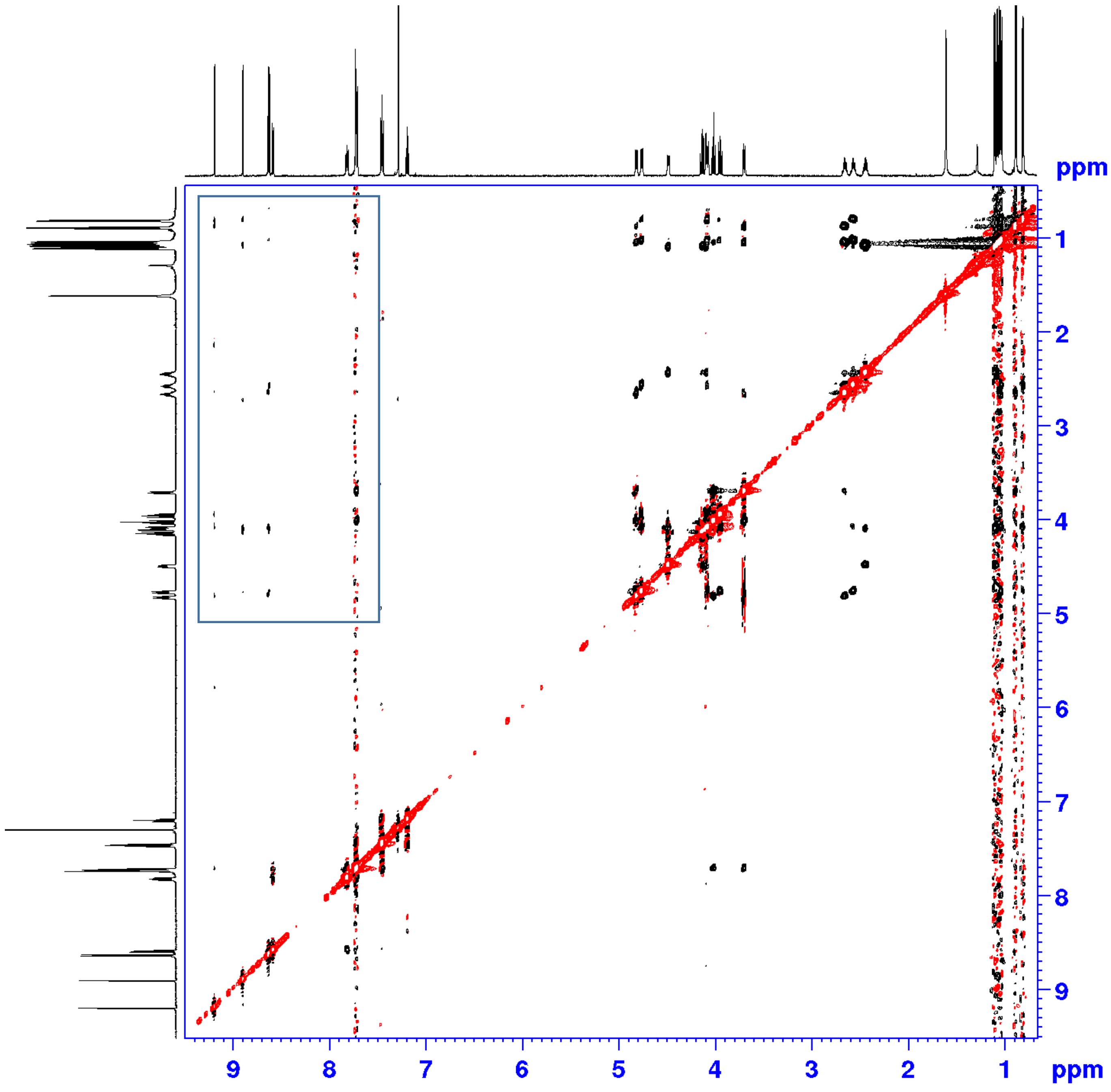

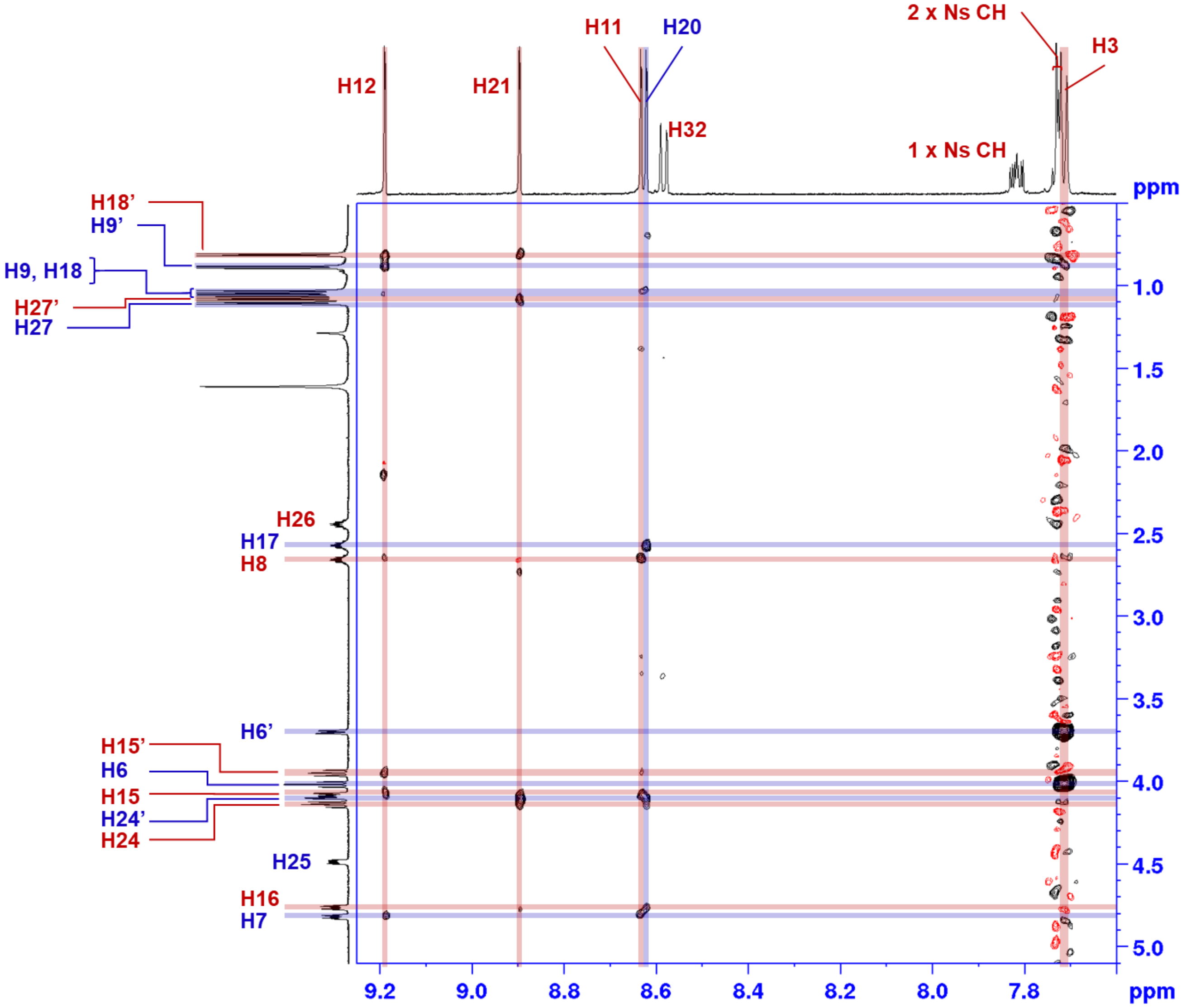

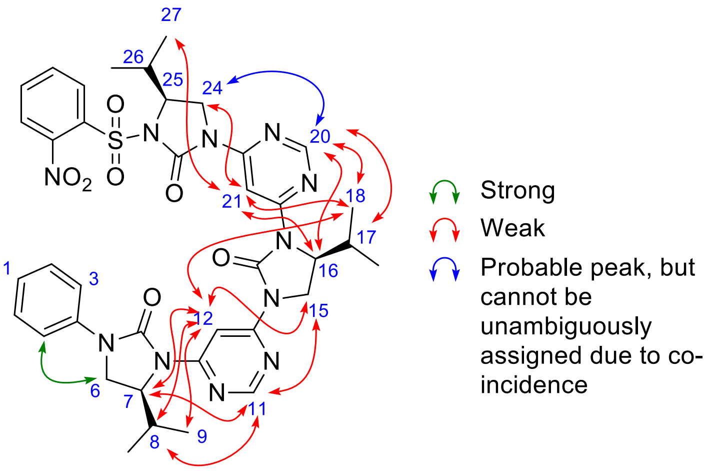

Table S5. Selected ranked nOe cross-peak intensities for trimer **13**

Peak	Normalized Intensity
H3 - H6	1.000
H3 - H6'	0.929
H21 - [H24+H24'][a]	0.137
[H15+H24+H24'] - [H11+H20][b]	0.124
H17 - H20	0.078
H9' - H12	0.070
H8 - H11	0.062
H21 - [H27+H27']	0.061
H12 - H15	0.052
H7 - H11	0.051
H12 - H15'	0.051
H18' - H21	0.050
H12 - H18'	0.042
H18 - H20	0.038
H7 - H12	0.037
H8 - H12	0.030
H11 - H15'	0.026
H16 - H21	0.023
H16 - H20	0.022
H9 - H12	0.017

[a] Peaks isochronous at the resolution of the ROESY experiment; [b] coalesced cross-peaks could not be unambiguously integrated.

The strong nOe between H3 and H6 was used as an internal standard for integration of peaks. Relative to this and H3↔H6', the H21↔[H24+H24'] and [H15+H24+H24']↔[H11+H20] cross-peaks were the next most intense. This is likely largely due to the isochronous nature of the peaks involved. H17↔H20 and H8↔H11 are both ranked relatively highly, and their presence strongly indicates that the dipole opposed conformation is adopted about the *N*-C10 and *N*-C19 bonds. The presence of an H11↔H15' cross-peak also suggests the dipole opposed conformation about the C13-*N* bond, since in the alternative dipole-aligned conformation these would be too far apart to experience an nOe. The analogous H20↔[H24+H24'] is likely also present, but due to overlap with H11↔H15 cannot be unambiguously interpreted.

More generally, the weakness of all imidazolidine-2-one cross peaks with adjacent *ortho*-aryl CHs is consistent with the proposed conformation. For example, the nOe intensity ratio between H12↔H15' and H3↔H6 is 1:20, where if there were no conformational bias around C13-*N* this ratio would be ~1:2.

Pyridazine Trimer 14

ROESY, $CDCl_3$, 600 MHz, t_{mix} = 0.2 s.

The fully expanded spectrum is presented at two different levels of intensity scaling.

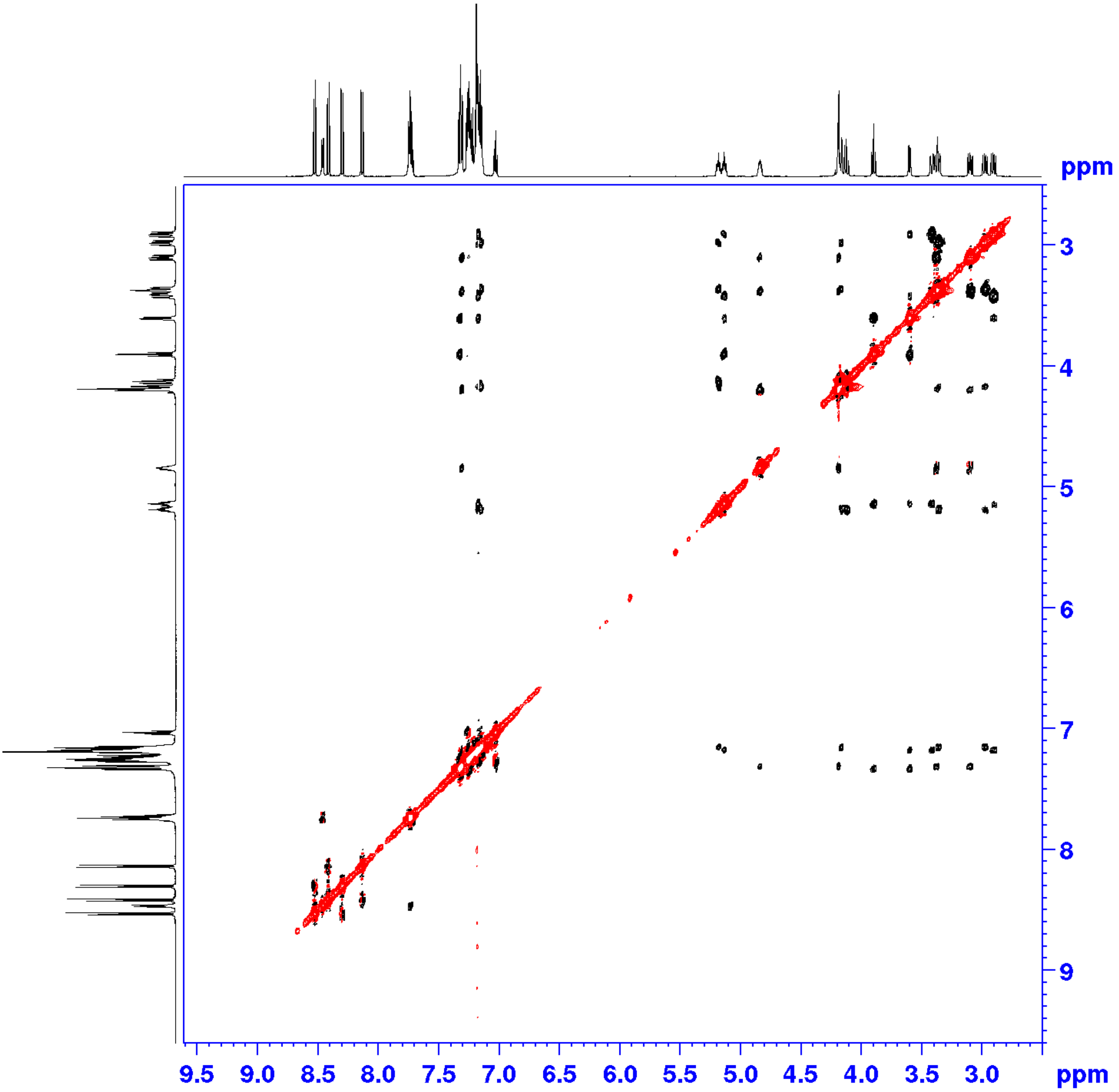

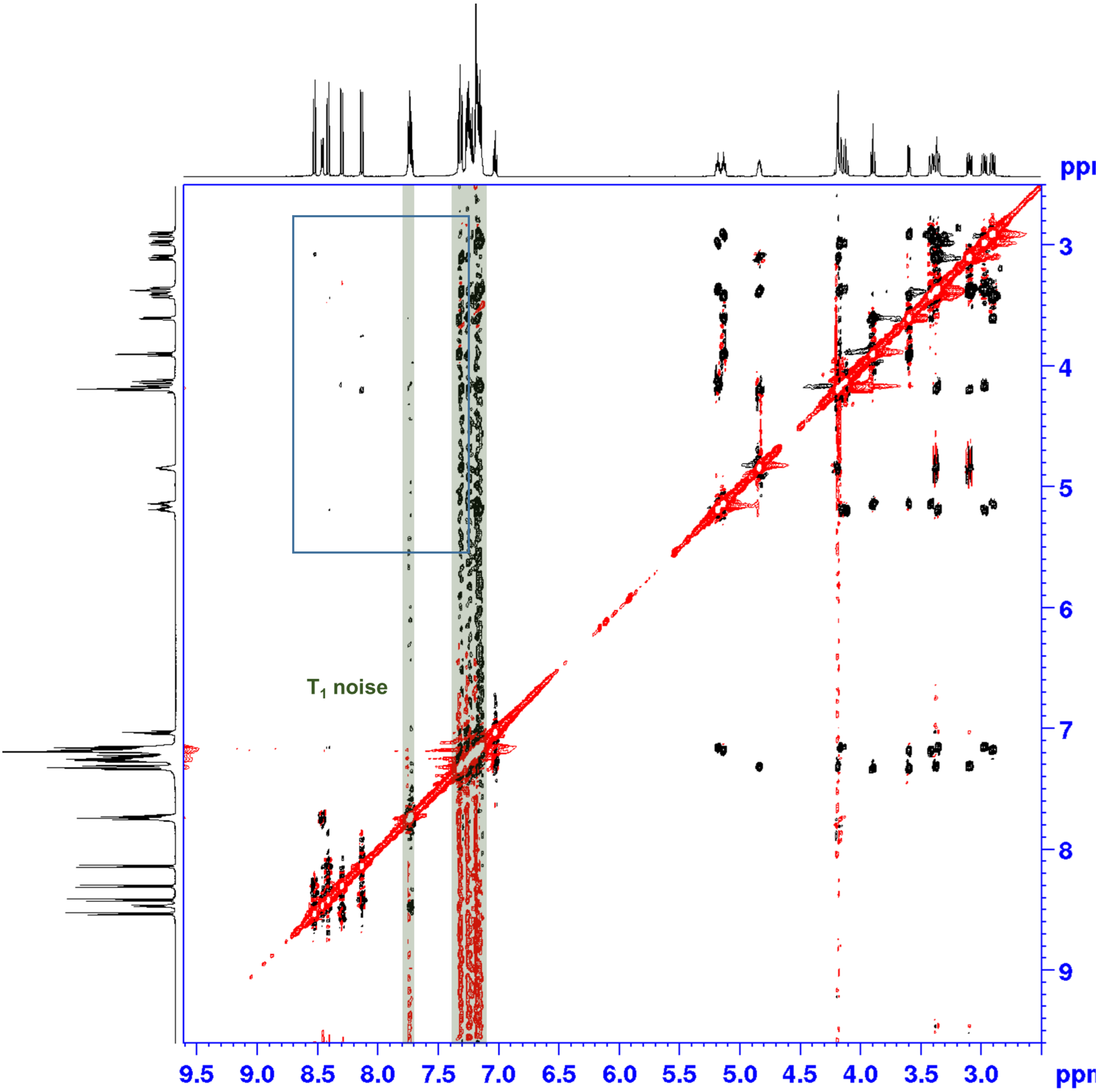
T1 noise
ppm
9.5
9.0
8.5
8.0
7.5
7.0
6.5
6.0
5.5
5.0
4.5
4.0
3.5
3.0
3
4
5
6
7
8
9

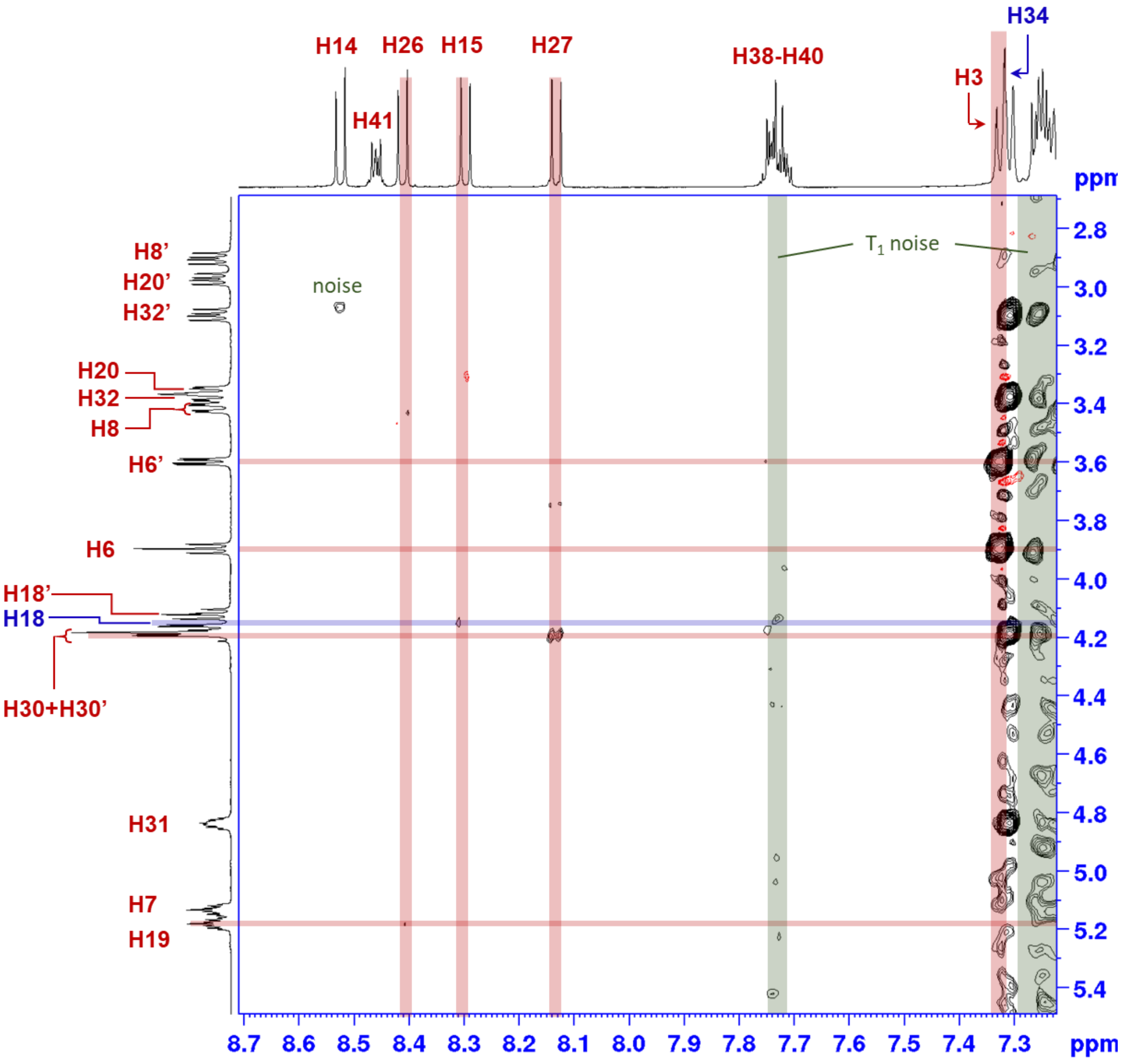

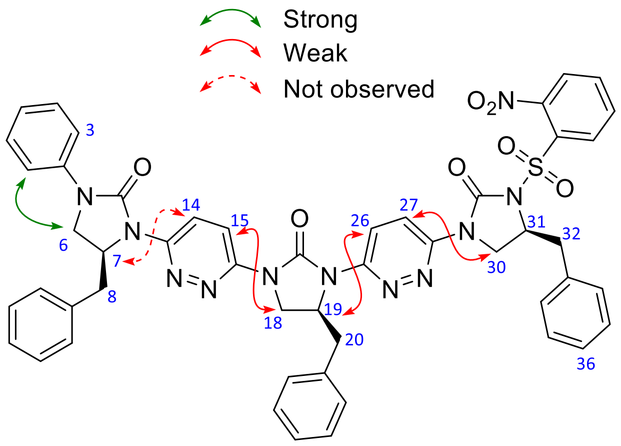

Table S6. Selected ranked nOe cross-peak intensities for trimer **14**

Peak	Normalized Intensity
H3 – H6	1.000
H3 – H6’	0.916
H27 – [H30+H30’][a]	0.057
H15 – H18	0.051
H19 – H26	0.035

[a] Peaks isochronous.

The strong nOe between H3 and H6 was used as an internal standard for integration of peaks. Relative to this and H3↔H6’, the H27↔H30 resonance was the next strongest peak, with an intensity ~6% of H3↔H6. Due to H30 and H30’ being isochronous, the true integral per hydrogen atom is likely closer to 0.03. The remaining cross-peaks of H15↔H18 and H19↔H26 were extremely weak, and H7↔H14 was not observed at an intensity that could be

distinguished from background noise. These observations are consistent with the dipole-opposed conformation depicted.

Pyrazine Trimer 15

ROESY, $CDCl_3$, 600 MHz, t_{mix} = 0.2 s.

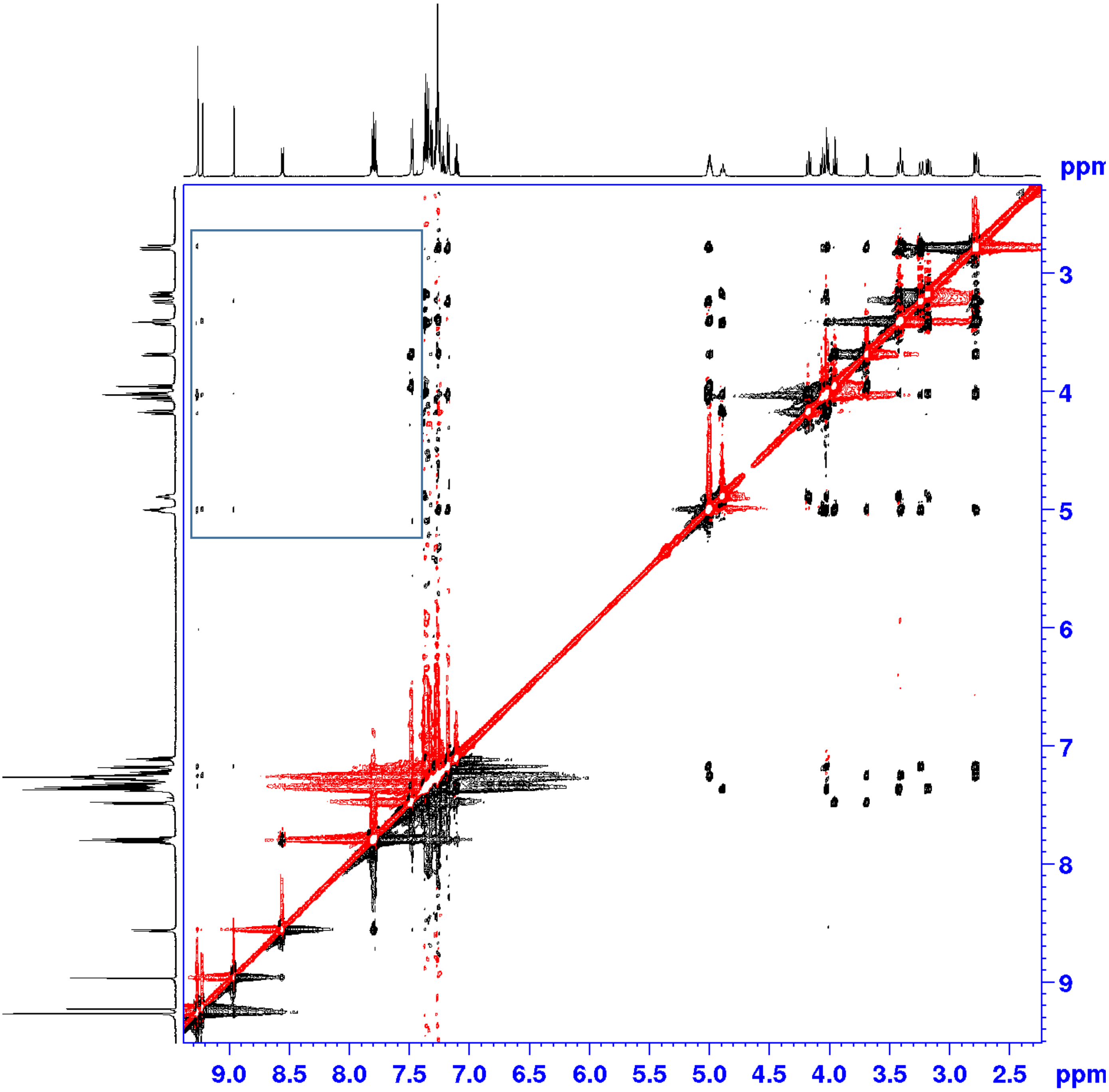

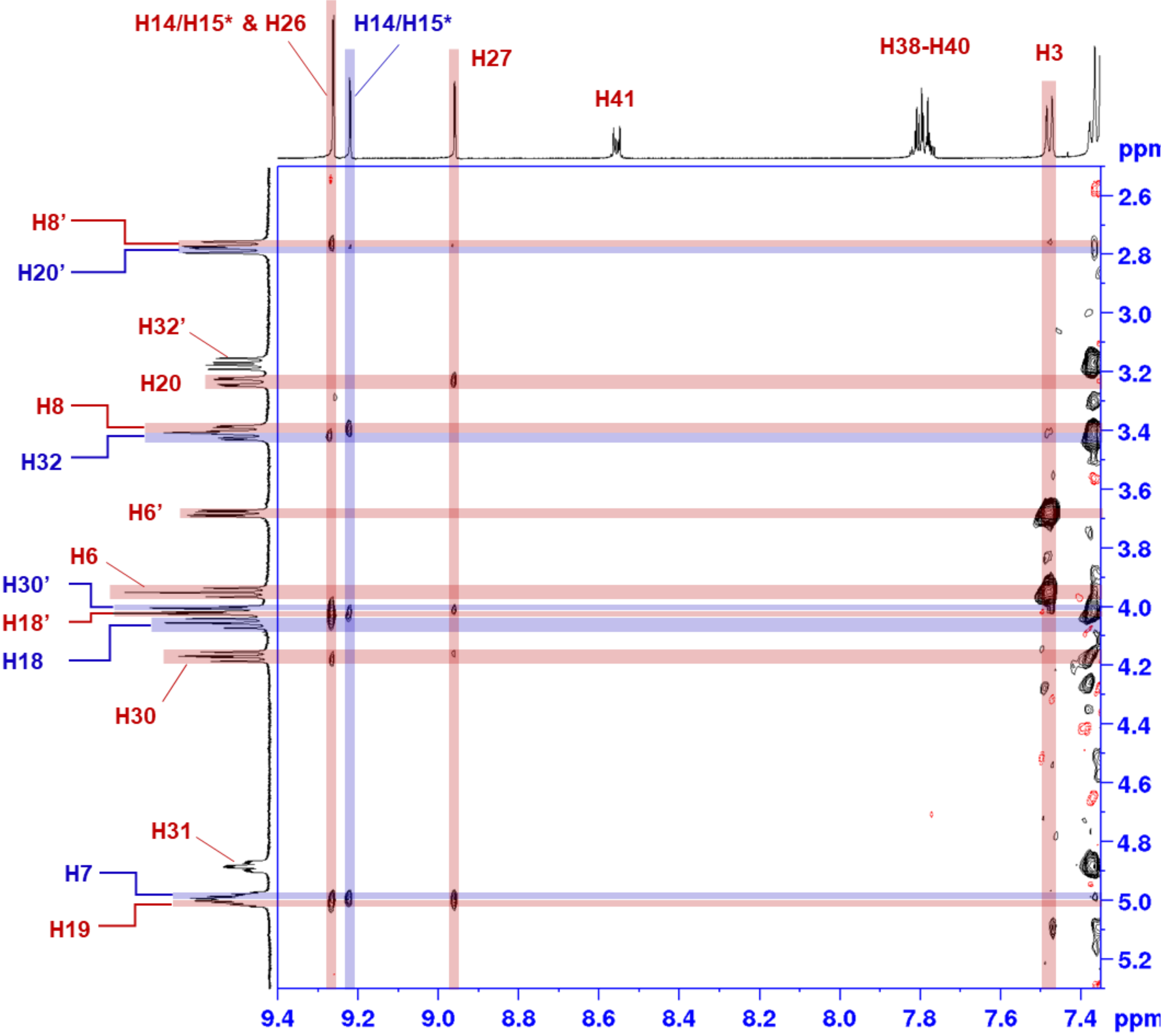

Table S7. Selected ranked nOe cross-peak intensities for trimer **15**

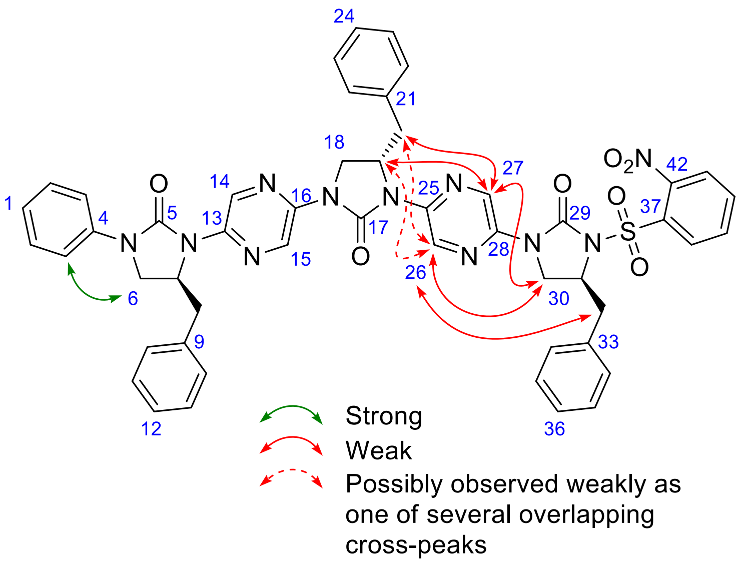

Peak	Normalized Intensity
H3 - H6'	1.000
H3 - H6	0.991
[H18+H18'+H30'] - [H14/15+H26][a]	0.155
[H7+H19] - [H14/15+H26][a]	0.065
H14/15 - H18'	0.064
H7 - H14/H15	0.057
H20 - H27	0.055
H19 - H27	0.051
H8 - H14/15	0.047
H26 - H32	0.041
[H8'+H20] - [H14/15+H26][a]	0.040
H26 - H30	0.033
H27 - H30'	0.030

[a] Coalesced cross-peaks could not be unambiguously integrated.

Due to the absence of HMBC cross-peaks corresponding to either H7↔C13 or H18↔C16, it was not possible to unambiguously assign protons H14 or H15 on the ^{1}H NMR spectrum of pyrazine trimer **15**. H26 is also isochronous with one of these two proton signals, which causes several conformationally relevant cross-peaks to overlap on the ROESY spectrum. Despite this, several clear conclusions regarding the conformational preferences of the molecule can be drawn from its ROESY spectrum.

The presence of weak cross-peaks corresponding to H26↔H30 and H26↔H32 indicates that the depicted conformation is preferred around the C28-*N* bond. Similarly, the presence of H19↔H27 and H20↔H27 peaks indicate the depicted conformation about the *N*-C25 bond, since these hydrogens would be too distant for an observable nOe in the alternative, dipoles-*syn* conformation. The weakness of the H19↔H26 and H27↔H30 peaks also supports the proposed conformation around the *N*-C25 and *N*-C28 bonds, since these would be expected to have ~1/2 the intensity of the H3↔H6 resonances if freely-rotating between *syn*- and *anti*-conformers.

As before, the strong nOe between H3 and H6' was used as an internal standard for the integration of peaks. Relative to this and H3 and H6, the next strongest conformationally relevant peak is ~16% the intensity of H3↔H6, which is relatively high when compared to the pyrazine dimer **12**; however, the fact that this cross-peak likely corresponds to three overlapping resonances (H14/15↔H18, H14/15↔H18' and H26↔H30') accounts for the observed intensity being slightly higher than expected. By analogy to the spectral features implicating the dipole-opposed conformation about the *N*-C25 and *N*-C28 bonds, the weakness of all other cross-peaks involving H14 and H15 (not indicated on structure due to the ambiguous NMR assignment of these protons) is consistent with the depicted conformation around the *N*-C13 and C16-*N* bonds.

Pyrimidine Tetramer 16

ROESY, $CDCl_3$, 600 MHz, t_{mix} = 0.2 s.

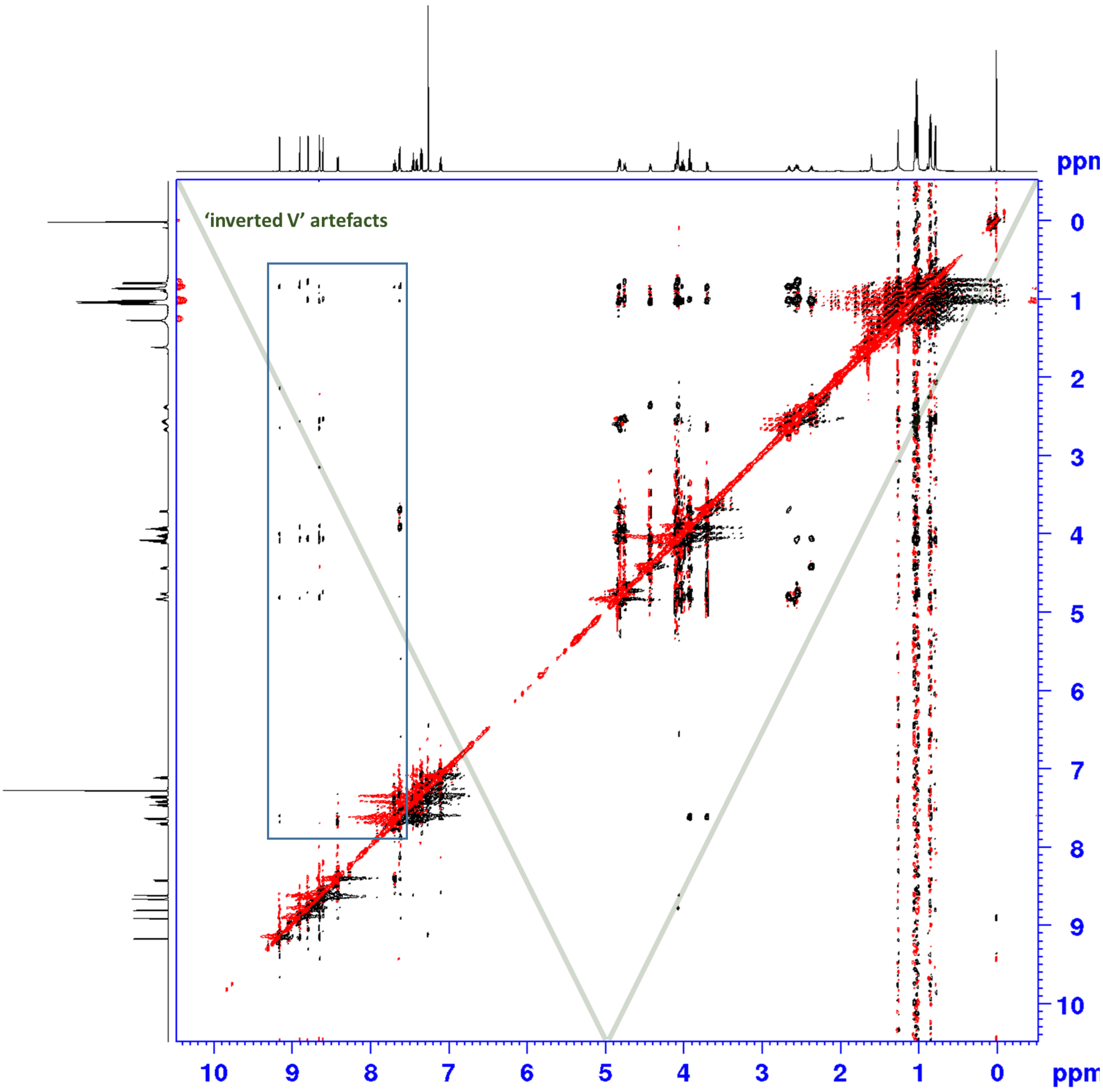

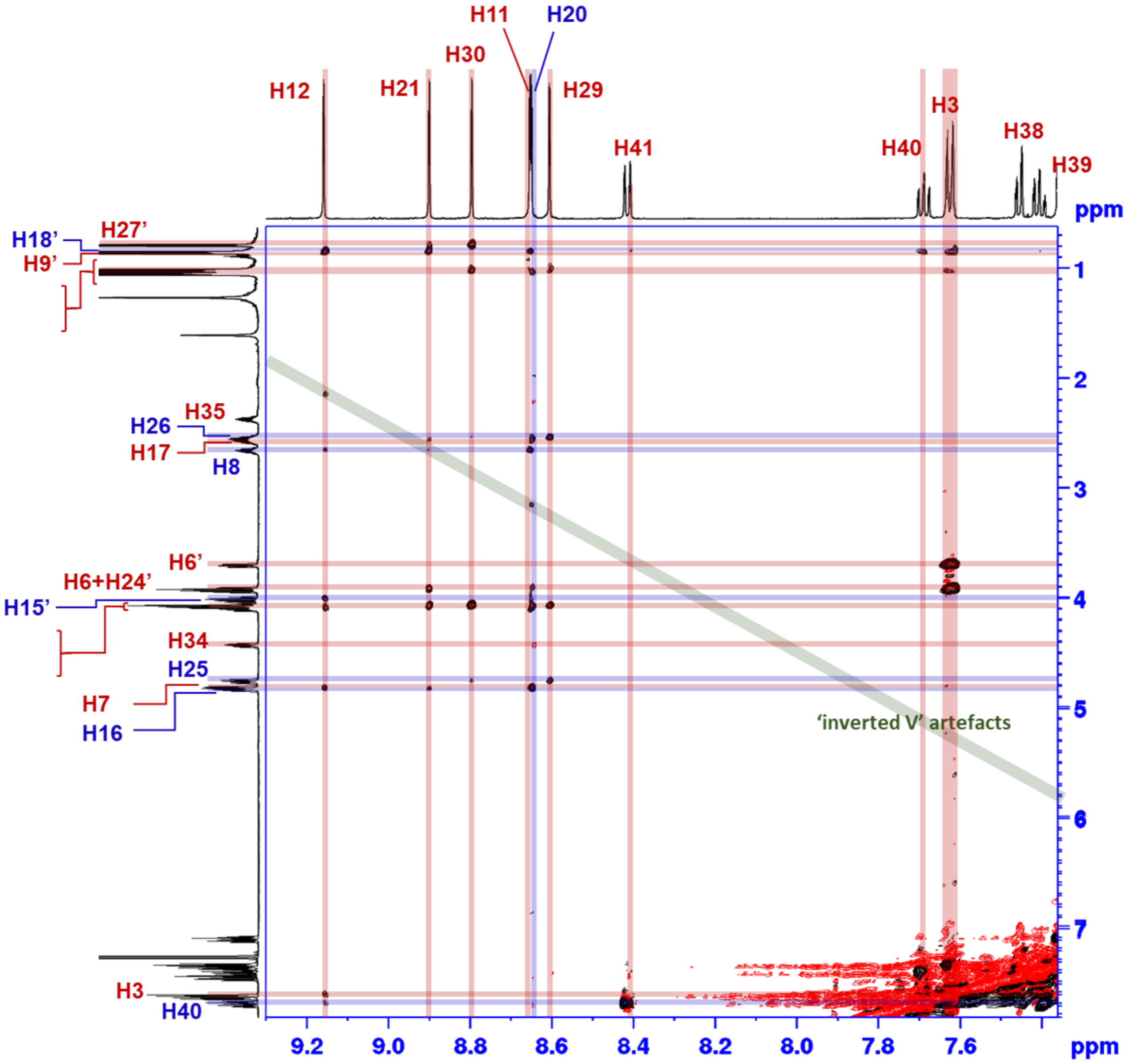

H11
H20
H30
H12
H21
H29
H41
H3
H40
H38
H39
ppm
H18'
H27'
H9'
H35
H26
H17
H8
H6'
H6+H24'
H15'
H34
H25
H7
H16
H3
H40
'inverted V' artefacts
1
2
3
4
5
6
7
9.2
9.0
8.8
8.6
8.4
8.2
8.0
7.8
7.6
ppm

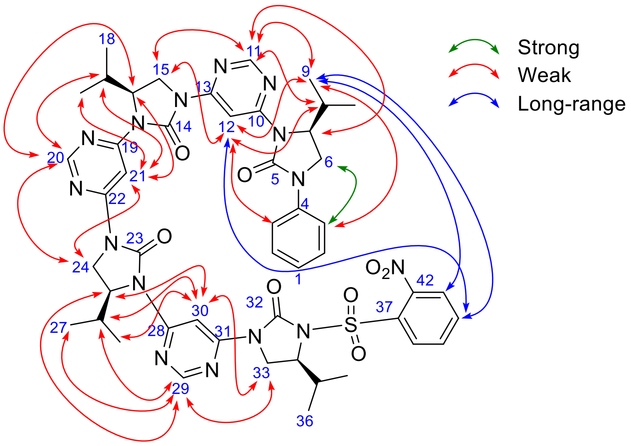

Table S10. Selected ranked nOe cross-peak intensities for tetramer **16**

Peak	Normalized Intensity
H3 - H6'	1.000
H3 - H6	0.848
H30 - [H33+H33'][a],[b]	0.159
H11 - H15[b]	0.108
H18' - H21	0.104
H9' - H12	0.102
H3 - H9'	0.092
H27' - H30	0.084
H21 - H24[b]	0.082
[H7 – H11 + H16 – H20]	0.066
H29 - [H33+H33'][a],[b]	0.065
**H9' - H40[c]**	**0.060**
H21 - H24'[b]	0.058
H8 - H11	0.054
H26 - H29	0.053
H12 - H15[b]	0.052
H27 - H30[b]	0.050
H25 - H29	0.050
H12 - H15'[b]	0.049
H27 - H29[b]	0.048
H18 - H20[b]	0.046
H17 - H20	0.045
H3 - H9[b]	0.042
H9' - H11	0.040
H16 - H21	0.036
H7 - H12	0.036
**H12 - H40[c]**	**0.035**
H20 - H24'[b]	0.034
H3 - H12	0.031
H25 - H30	0.025
**H9' - H41[c]**	**0.025**
H26 - H30	0.021
H17 - H21	0.020
H3 - H6'	1.000

[a] Peaks isochronous; [b] the identity of this peak is inferred, since one of the corresponding peaks in the 1D spectrum lies within an unresolved multiplet; [c] lines in bold indicate long-range nOe correlations.

The strong nOe between H3 and H6' was used as an internal standard for integration of peaks. Relative to this and H3↔H6, the H30↔[H33+H33'] resonance was the next strongest peak, with an intensity ~16% of H3↔H6'. Its unusually high intensity is likely due to the anisochronous nature of the H33 and H33' resonances. In line with the shorter oligomers in this series, the presence of resonances H7↔H11, H11↔H15, H16↔H20, H20↔H24', H25↔H29 and H29↔[H33+H33'] indicates the depicted conformation is adopted, since in the alternative conformation (with pyrimidine and urea dipoles aligned) these nOes would be negligible in magnitude. In addition, long-range nOes were observed between H9'↔H40, H9'↔H41 and H12↔H40. These provide strong evidence that the foldamer does indeed adopt the fully curved conformation depicted, bringing its termini into close contact. This is supported by NMR chemical shift analysis of the ^{1}H resonances corresponding to the nosyl protecting group. When compared with all other *N*-nosyl foldamers prepared in this study, there is a clear dispersion and upfield shift of the four peaks (Figure S1). In all other foldamers examined, the *H ortho*-to the nitro group appears above 8.45 ppm, and the remaining three resonances

appear as a multiplet between 7.70-7.85 ppm, whereas in tetramer **16** the former appears at 8.41 ppm, and the latter are resolved and appear between 7.69 and 7.40 ppm.

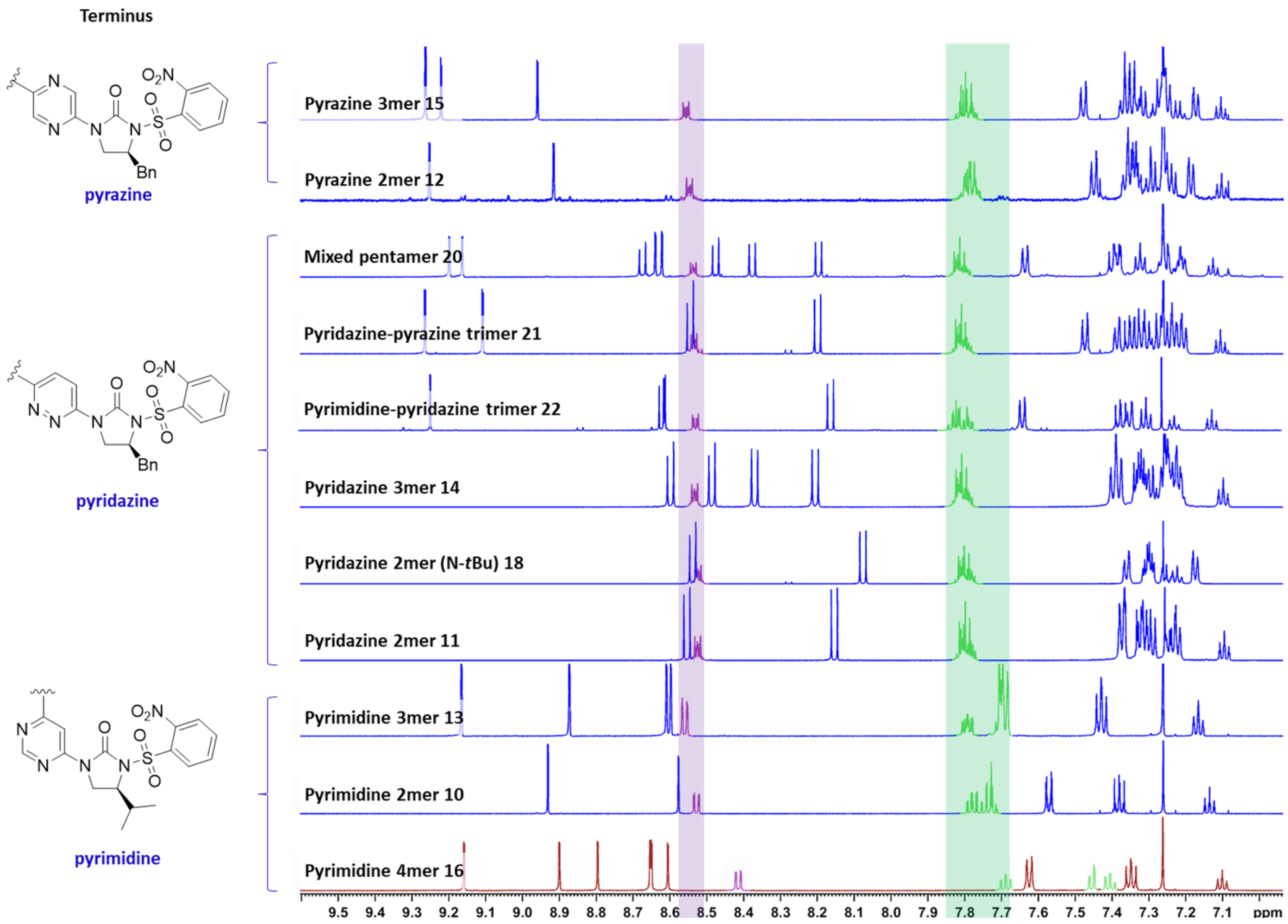

Figure S1. ^{1}H NMR spectra of tetramer **16** (bottom, maroon) and all other N-Ns foldamers prepared in this study (blue), highlighting the appearance of the nosyl protecting group. The CH *ortho*-to the nitro group appears in purple, and the remaining three peaks appear in green.

Mixed Pentamer 20

ROESY, $CDCl_3$, 600 MHz, t_{mix} = 0.2 s.

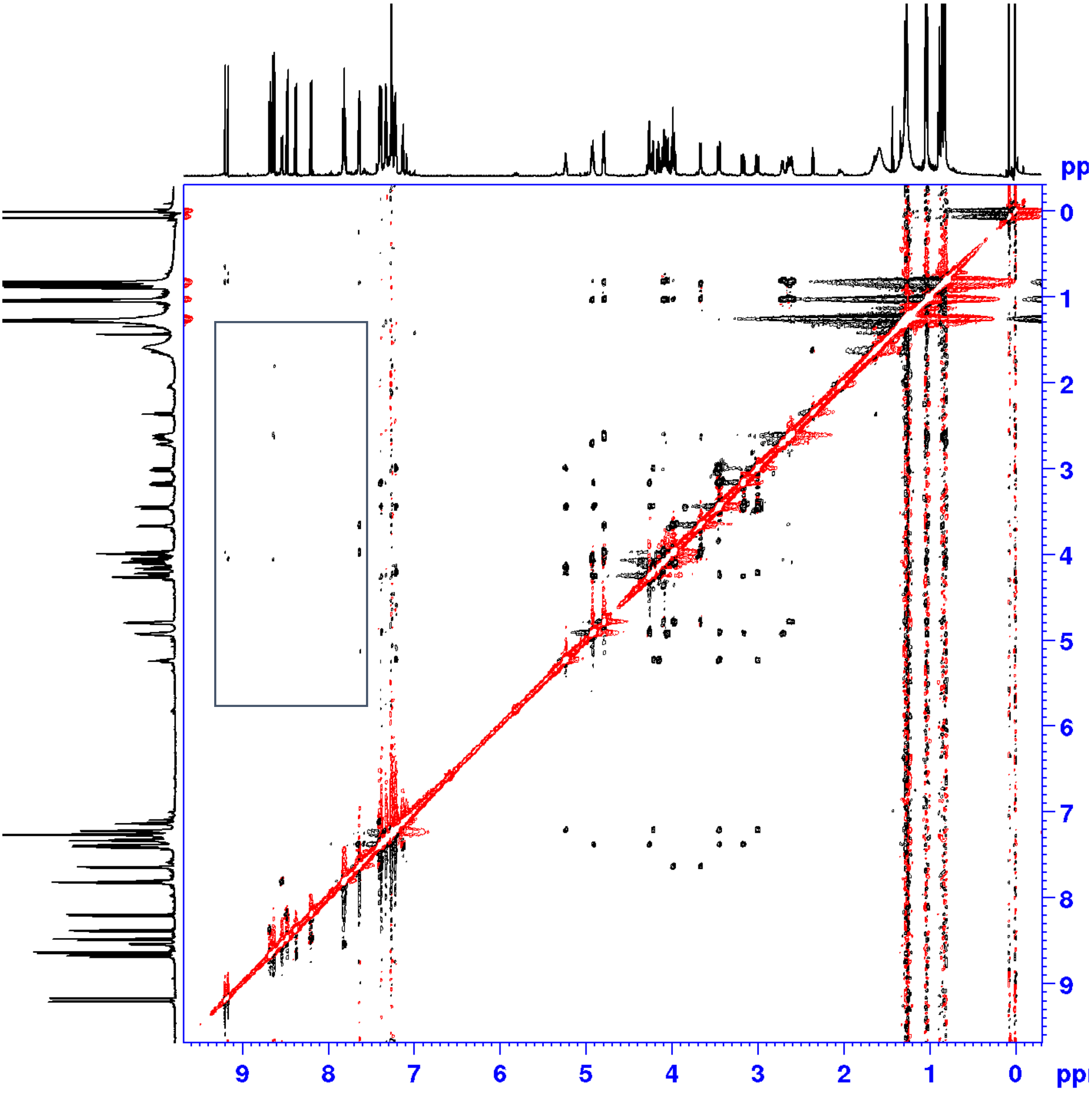

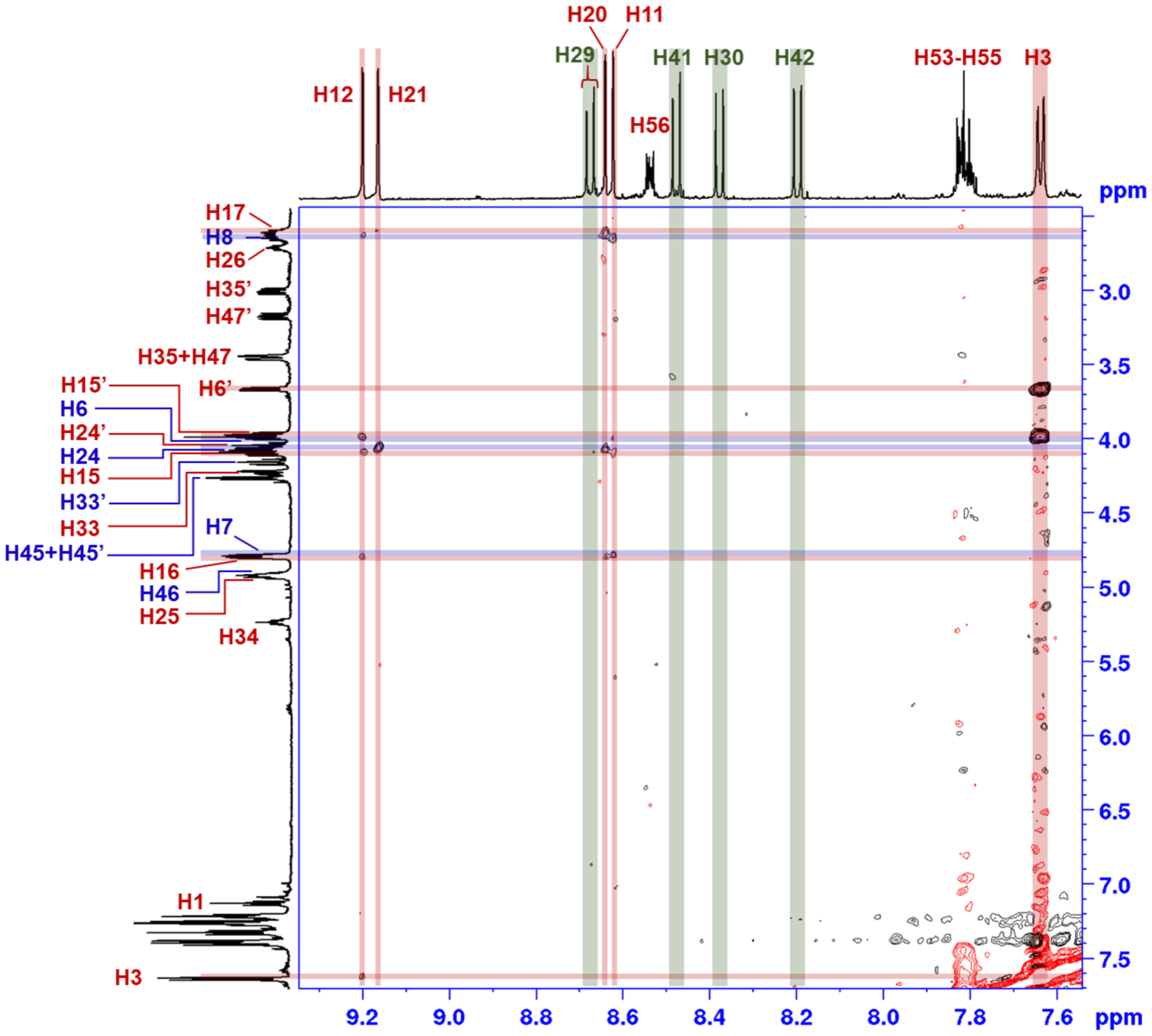

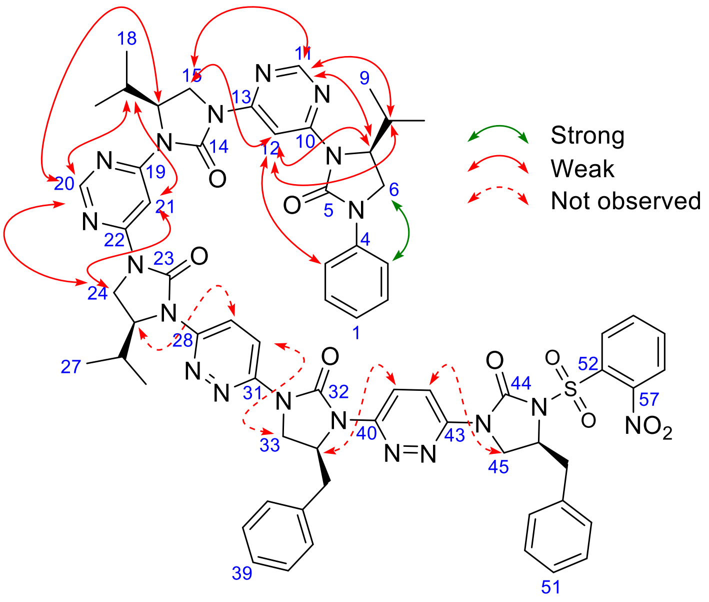

Table S11. Selected ranked nOe cross-peak intensities for pentamer **20**

Peak	Normalized Intensity
H3 - H6'	1.000
H3 - H6	0.970
H21 – [H24+H24'][a]	0.122
H20 – [H24+H24'][a]	0.096
H17 - H20	0.085
H8 - H11	0.061
H12 - H15'	0.044
H11 - H15	0.041
H12 - H15	0.040
H17 - H21	0.036
H7 - H12	0.035
H7 - H11	0.028
H3 - H12	0.026
H16 - H20	0.022
H8 - H12	0.016

[a] Peaks isochronous

The strong nOe between H3 and H6' was used as an internal standard for integration of peaks. Relative to this and H3↔H6, the next strongest resonance was H21↔[H24+H24']. This was the only peak for which two hydrogens were isochronous – it can be inferred that each diastereotopic H24 peak contributes ~0.06 to this normalised integral of 0.122, so the strength of this nOe relative to those below it is inconsequential. The H17↔H20 and H8↔H11 peaks are relatively strong, indicating the depicted dipole-opposed conformation is favoured about the C19-*N* and C10-*N* bonds. H20↔[H24+H24'] and H21↔[H24+H24'] are similar in intensity, as are H11↔H15 and H12↔H15, again indicating the depicted conformation around the C22-*N* and C13-*N* bonds, since in the alternative (dipole aligned) conformation, H21↔H24 and H15↔H12 would be much stronger than H20↔H24 and H15↔H11 respectively. Regarding the conformation around the *N*-$C_{pyridazine}$ bonds, no nOes were observed that could be discerned from the background noise (see green bands on zoomed spectrum. This is consistent with the depicted conformation, since if no control were in effect the observed intensity should be ~1/2 the H3↔H6 intensity.

Pyridazine-Pyrazine Trimer 21

ROESY, $CDCl_3$, 600 MHz, t_{mix} = 0.2 s

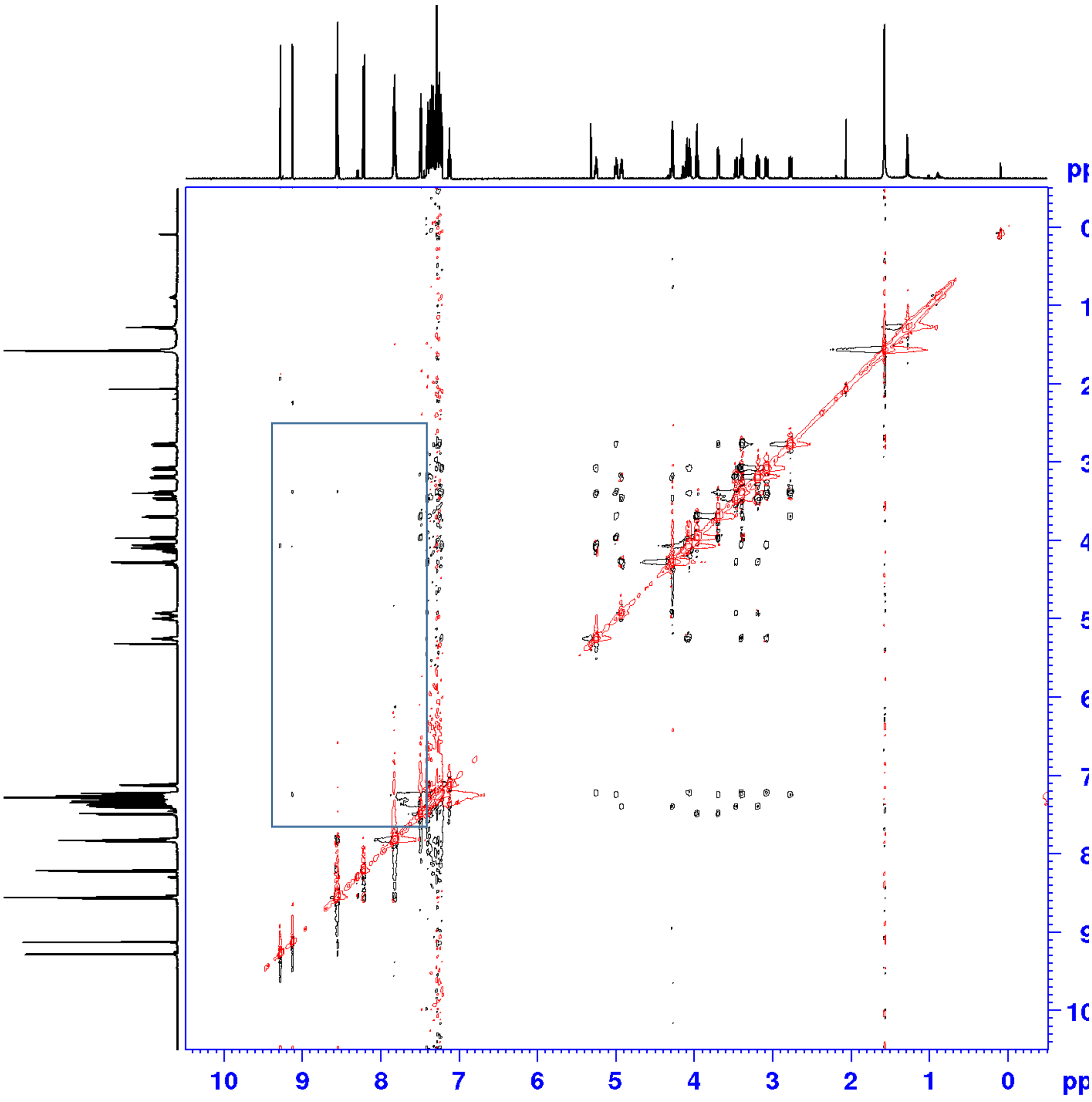

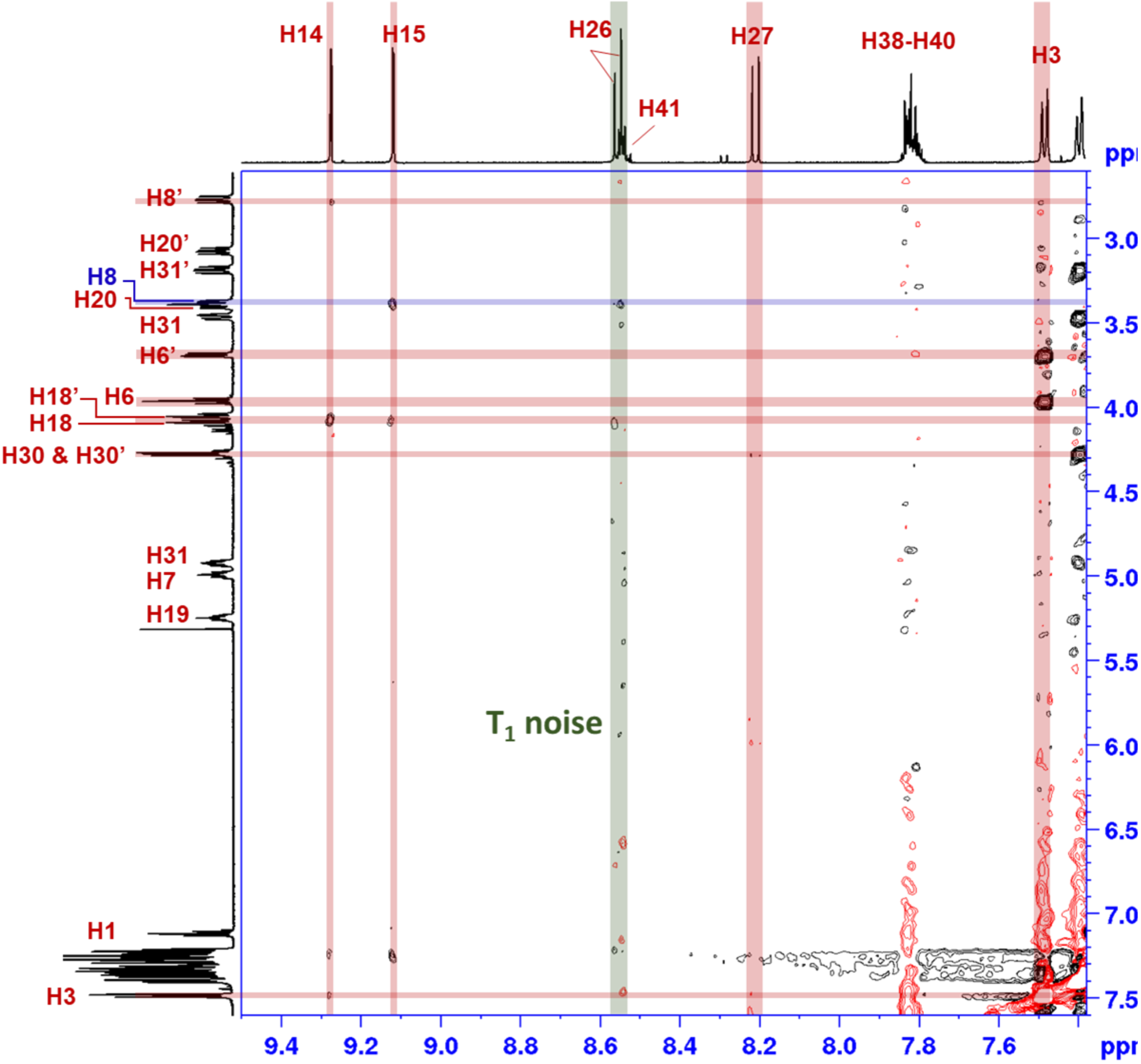

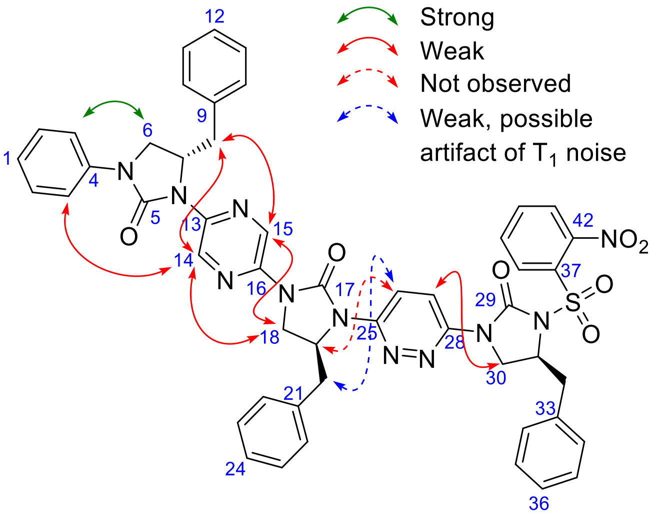

Table S8. Selected ranked nOe cross-peak intensities for trimer **21**

Peak	Normalized Intensity
H3 - H6'	1.000
H3 - H6'	0.961
[H18+H18'][a] - H14	0.091
H8 - H15	0.064
H27 - [H30+H30'][a]	0.062
H20 - H26[b]	0.049
H15 - [H18+H18'][a]	0.040
H3 - H14	0.027
H8' - H14	0.024

[a] Peaks isochronous; [b] corresponds to a band of T_1 noise, so the presence and intensity of this peak should be regarded with caution.

The strong nOe between H3 and H6’ was used as an internal standard for integration of peaks. Relative to this and H3↔H6, the H18↔H14 resonance was the next strongest peak, with an intensity ~9% of H3↔H6’. Due to H18 and H18’ being isochronous, the true integral per hydrogen atom is likely closer to 0.045. However, the presence of this peak, and of H8↔H15 (the next most intense), indicate the depicted conformation, since if the *N*-$C_{pyrazine}$ bonds were rotated 180°, the H15↔H18 and H8↔H14 nOe would be much stronger than these. Similarly, the H3↔H14 nOe strongly indicated the conformation given about the *N*-C13 bond. A peak of relative intensity 0.049 was observed between H20 and H26, however this peak corresponds with a strong band of T_1 noise (see blue vertical band on spectrum) so no major conclusions can be drawn from its presence. A weak H27↔H30 nOe and the absence of an H19↔H26 nOe indicate the predicted, dipole-opposed conformation is adopted about the *N*-$C_{pyridazine}$ bonds.

Pyrimidine-Pyrazine Trimer 22

ROESY, $CDCl_3$, 600 MHz, t_{mix} = 0.2 s.

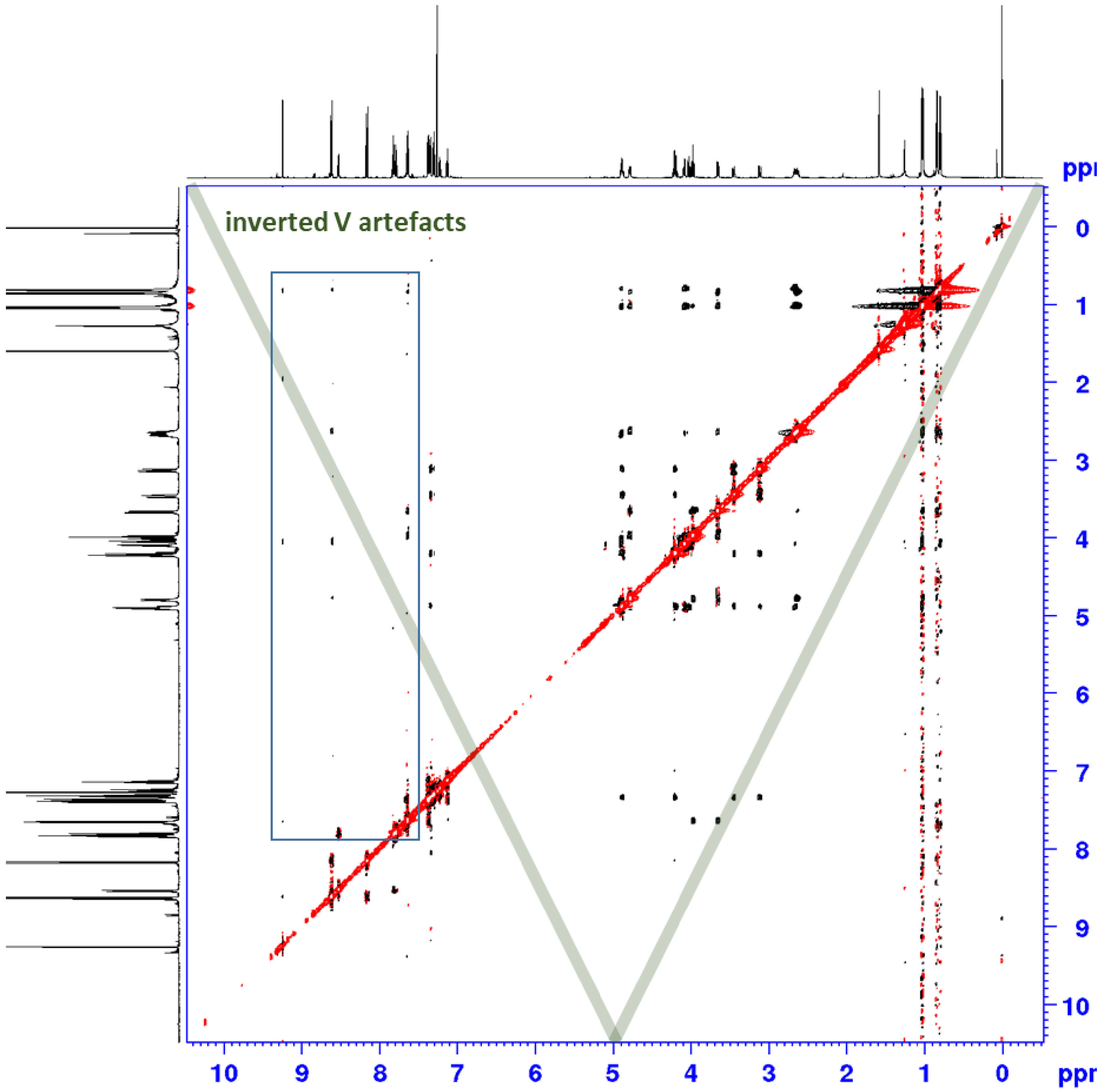

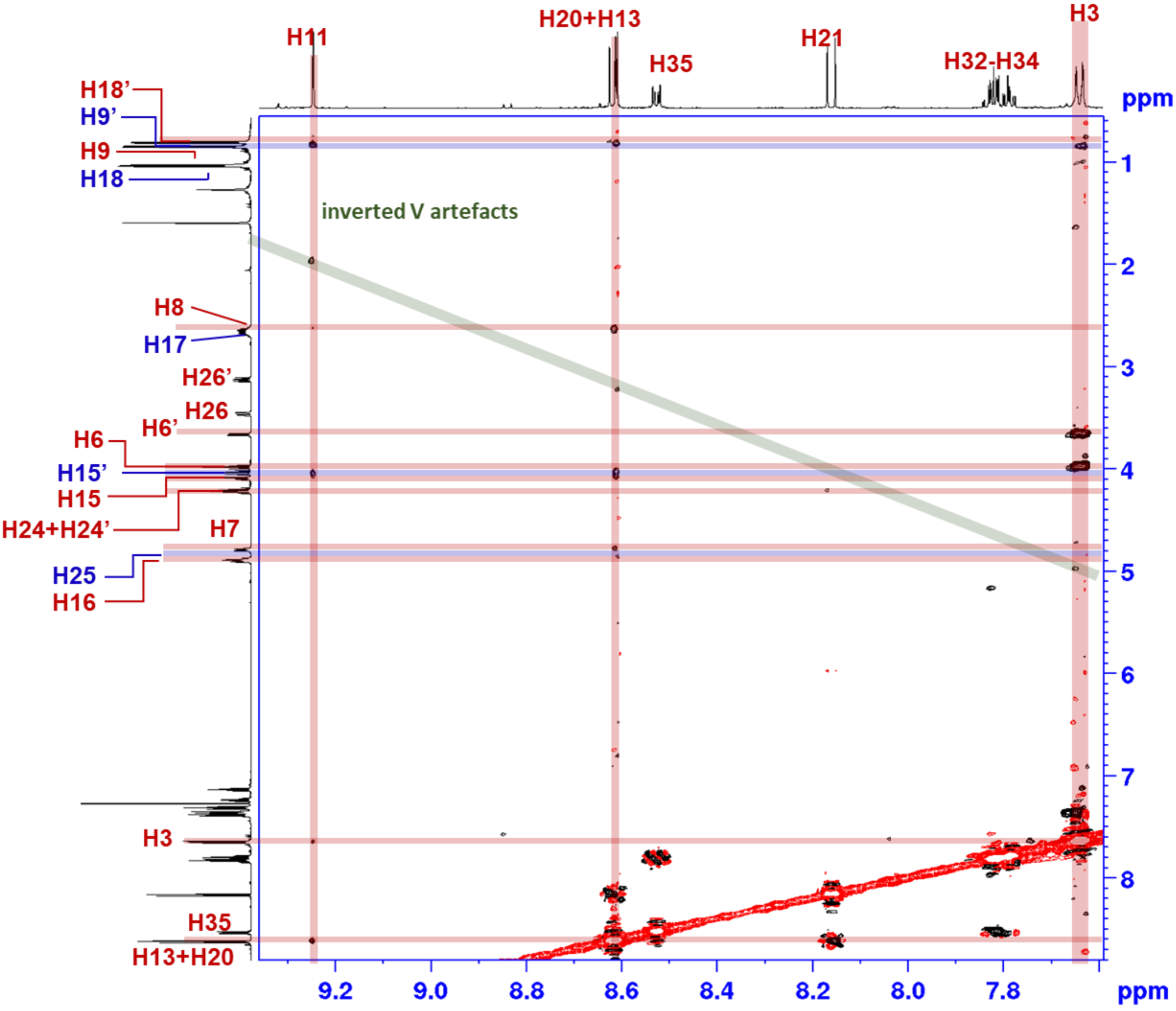

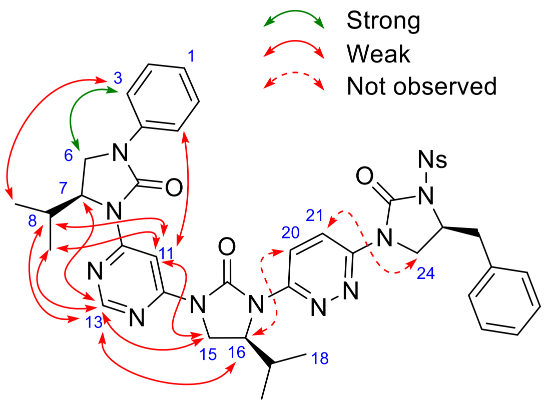

Table S9. Selected ranked nOe cross-peak intensities for trimer **22**

Peak	Normalized Intensity
H3 - H6'	1.000
H3 - H6	0.909
H3 - H9'	0.109
H9' - H13	0.086
H9' - H11	0.086
H11 - H15'	0.080
H8 - H13	0.069
H13 - H15	0.054
H13 - H15'	0.040
H13 - H16	0.034
H8 - H11	0.023
H3 - H11	0.021
H7 - H13	0.013

The strong nOe between H3 and H6' was used as an internal standard for integration of peaks. Relative to this, the next strongest conformationally relevant peak was H9'↔H13. The presence of this, and H7↔H13, indicate that the depicted conformation is preferred around the *N*-C10 bond. Similarly, the presence of H13↔H15 and H13↔H16 peaks indicate the depicted conformation about the *N*-C14 bond, since these hydrogens would be too distant for an observable nOe in the alternative, dipoles-*syn* conformation. The absence of H16↔H20 and H21↔H24 peaks supports the proposed conformation around the *N*-$C_{pyrazine}$ bonds, since these would be expected to have ~1/2 the intensity of H3↔H6 resonances if freely-rotating between *syn*- and *anti*-conformers.

X-Ray Crystallography

Data for compounds **3**, **4**, **5**, **10**, **11** and **12** were collected as described in the General Experimental section.

Pyrimidine Monomer 3

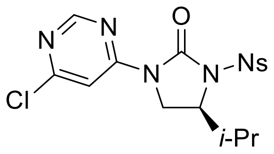

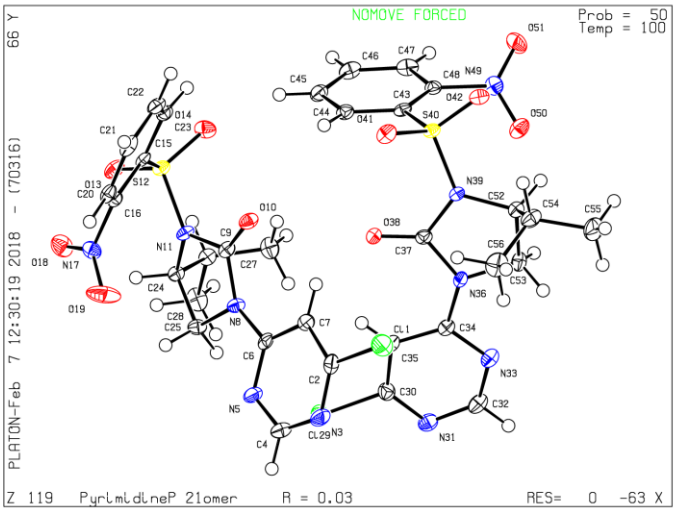

Crystal Data

a = 8.02450(10) Å α = 90°

b = 12.37800(10) Å β = 99.3938(10)°

c = 18.9641(2) Å γ = 90°

Volume	1858.39(3) $Å^3$	Crystal Class	monoclinic
Space group	$P\ 2_1$	Z =	1
Formula	$C_{64}H_{64}Cl_4N_{20}O_{20}S_4$	M_r	1703.41
Cell determined from	8288 reflections	Cell θ range =	6 - 72°
Temperature	100K		
Pressure	100 kPa	Shape	block
Colour	clear_pale_colourless	Size	0.14 × 0.16 × 0.20 mm
D_x	1.52 Mg m^{-3}	F000	880.000
μ	3.238 mm^{-1}		
Absorption correction	multi-scan		
T_{min}	0.54	T_{max}	0.64

Data Collection

Diffractometer	multi-scan
Scan type	ω scans
Reflections measured	10980
Independent reflections	7076
Rint	0.0228
$θ_{max}$	72.4100
h =	-7 → 9
k =	-15 → 15

l =	-23 → 18

Refinement

$\Delta\rho_{min}$ =	-0.24 e Å^{-3}
$\Delta\rho_{max}$ =	0.19 e Å^{-3}
Reflections used	7049
Cutoff: I >	-3.00σ(I)
Parameters refined	506
S =	1.14
R-factor	0.026
weighted R-factor	0.043
Δ/σ_{max}	0.0005
Flack parameter	0.012(7)
Refinement on	F^2
w =	$w' \times [1 - (\Delta F_{obs} / 6 \times \Delta F_{est})^2]^2$
w′ =	$[P_0T_0'(x) + P_1T_1'(x) + ...P_{n-1}T_{n-1}'(x)]^{-1}$, where P_i are the coefficients of a Chebychev series in $t_i(x)$, and $x = F_{calc}^2/F_{calc}^2{}_{max}$.
P_0 - P_{n-1} =	673. 859. 410.

Pyridazine Monomer 4

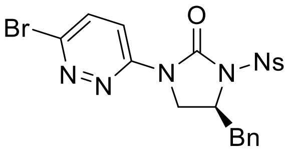

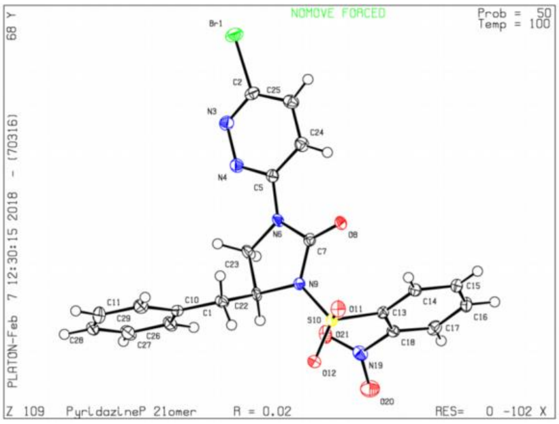

Crystal Data

a = 10.11450(10) Å α = 90°
b = 9.86380(10) Å β = 102.2561(10)°
c = 10.66220(10) Å γ = 90°

Volume	1039.496(18) Å^3	Crystal Class	monoclinic
Space group	P 2_1	Z =	2
Formula	C_{20} H_{16} Br_1 N_5 O_5 S_1	M_r	518.35
Cell determined from	10793 reflections	Cell θ range =	4 - 72°
Temperature	100K		
Shape	plate		
Colour	clear_pale_colourless	Size	0.04 × 0.10 × 0.16 mm
D_x	1.66 Mg m^{-3}	F000	524.000
μ	4.033 mm^{-1}		
Absorption correction	multi-scan		
T_{min}	0.47	T_{max}	0.85

Data Collection

Diffractometer	multi-scan
Scan type	ω scans
Reflections measured	19414
Independent reflections	4018
Rint	0.0328
$θ_{max}$	72.4313
h =	-12 → 12
k =	-12 → 12
l =	-13 → 13

Refinement

$Δρ_{min}$ =	-0.37 e Å^{-3}
$Δρ_{max}$ =	0.29 e Å^{-3}
Reflections used	4004
Cutoff: I >	-3.00σ(I)
Parameters refined	290

S =	0.88
R-factor	0.023
weighted R-factor	0.055
Δ/σ_{max}	0.0014
Flack parameter	-0.014(10)
Refinement on	F^2
w =	$w' \times [1 - (\Delta F_{obs} / 6 \times \Delta F_{est})^2]^2$
w′ =	$[P_0T_0'(x) + P_1T_1'(x) + ...P_{n-1}T_{n-1}'(x)]^{-1}$, where P_i are the coefficients of a Chebychev series in $t_i(x)$, and $x = F_{calc}^2/F_{calc}^2{}_{max}$.
$P_0 - P_{n-1}$ =	0.216E+04 0.356E+04 0.237E+04 0.110E+04 354.

Pyrazine Monomer 5

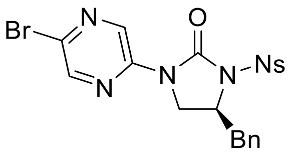

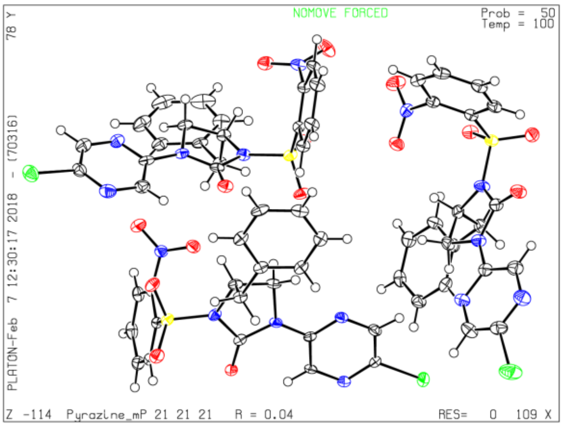

Crystal Data

a = 8.02780(10) Å α = 90°
b = 24.1538(3) Å β = 90°
c = 32.2777(3) Å γ = 90°

Volume	6258.71(12) $Å^3$	Crystal Class	orthorhombic
Space group	$P\ 2_1\ 2_1\ 2_1$	Z =	1
Formula	$C_{240}\ H_{192}\ Br_{12}\ N_{60}\ O_{60}\ S_{12}$	M_r	6220.17
Cell determined from	37987 reflections	Cell θ range =	4 - 72°
Temperature	100K		
Shape	plates		
Colour	clear_pale_colourless	Size	0.10 × 0.18 × 0.26 mm
D_x	1.65 Mg m^{-3}	F000	3144.000
μ	4.019 mm^{-1}		
Absorption correction	multi-scan		
T_{min}	0.36	T_{max}	0.67

Data Collection

Diffractometer	multi-scan
Scan type	ω scans
Reflections measured	59389
Independent reflections	12235
Rint	0.0604
$θ_{max}$	72.6945
h =	-9 → 9
k =	-25 → 29
l =	-39 → 39

Refinement

$Δρ_{min}$ =	-1.12 e $Å^{-3}$
$Δρ_{max}$ =	1.11 e $Å^{-3}$
Reflections used	12184
Cutoff: I >	-3.00σ(I)

Parameters refined	866
S =	0.90
R-factor	0.040
weighted R-factor	0.092
Δ/σ_{max}	0.0183
Flack parameter	-0.011(11)
Refinement on	F^2
w =	$w' \times [1 - (\Delta F_{obs} / 6 \times \Delta F_{est})^2]^2$
w′ =	$[P_0T_0'(x) + P_1T_1'(x) + ...P_{n-1}T_{n-1}'(x)]^{-1}$, where P_i are the coefficients of a Chebychev series in $t_i(x)$, and $x = F_{calc}^2/F_{calc}^2{}_{max}$.
P_0 - P_{n-1} =	0.184E+04 0.297E+04 0.193E+04 849. 243.

Pyrimidine Dimer 10

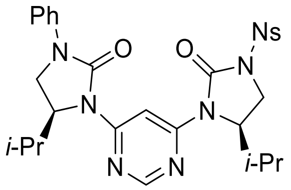

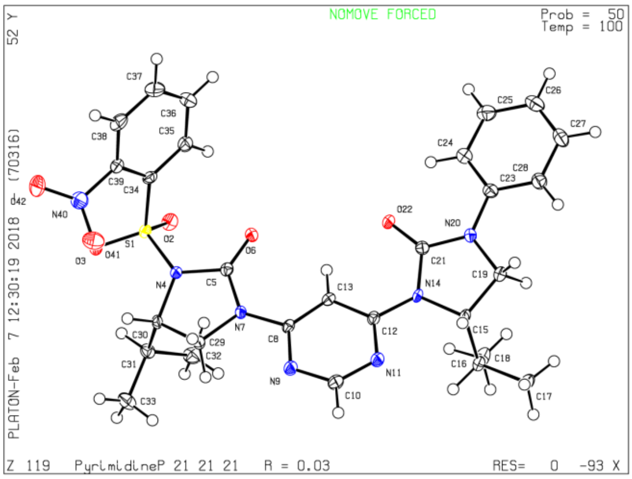

Crystal Data

a = 6.25710(10) Å α = 90°
b = 16.3319(2) Å β = 90°
c = 27.6898(3) Å γ = 90°

Volume	2829.63(6) $Å^3$	Crystal Class	orthorhombic
Space group	P 2_1 2_1 2_1	Z =	1
Formula	C_{112} H_{124} N_{28} O_{24} S_4	M_r	2374.65
Cell determined from	15554 reflections	Cell θ range =	4 - 72°
Temperature	100K		
Pressure	100 kPa	Shape	needles
Colour	clear_pale_colourless	Size	0.04 × 0.06 × 0.14 mm
D_x	1.39 Mg m^{-3}	F000	1248.000
μ	1.490 mm^{-1}		
Absorption correction	multi-scan		
T_{min}	0.81	T_{max}	0.94

Data Collection

Diffractometer	multi-scan
Scan type	ω scans
Reflections measured	26805
Independent reflections	5544
Rint	0.0361
$θ_{max}$	72.5542
h =	-7 → 7
k =	-19 → 19
l =	-33 → 33

Refinement

$Δρ_{min}$ =	-0.23 e $Å^{-3}$
$Δρ_{max}$ =	0.19 e $Å^{-3}$

Reflections used	5524
Cutoff: I >	-3.00σ(I)
Parameters refined	380
S =	1.15
R-factor	0.027
weighted R-factor	0.040
Δ/σ_{max}	0.0012
Flack parameter	0.005(11)
Refinement on	F^2
w =	$w' \times [1 - (\Delta F_{obs} / 6 \times \Delta F_{est})^2]^2$
w′ =	$[P_0T_0'(x) + P_1T_1'(x) + ...P_{n-1}T_{n-1}'(x)]^{-1}$, where P_i are the coefficients of a Chebychev series in $t_i(x)$, and $x = F_{calc}^2/F_{calc}^2{}_{max}$.
$P_0 - P_{n-1}$ =	509. 570. 278.

Pyridazine Dimer 11

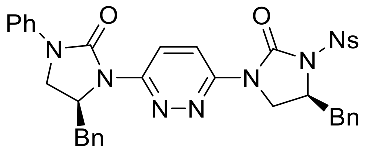

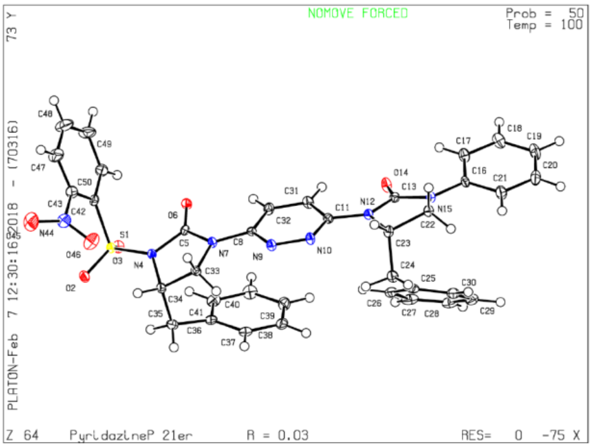

Crystal Data

a = 6.40670(10) Å α = 90°
b = 13.47240(10) Å β = 96.0676(10)°
c = 18.9255(2) Å γ = 90°

Volume	1624.38(3) $Å^3$	Crystal Class	monoclinic
Space group	P 2_1	Z =	1
Formula	C_{72} H_{62} N_{14} O_{12} S_2	M_r	1379.50
Cell determined from	6589 reflections	Cell θ range =	6 - 72°
Temperature	100K		
Pressure	100 kPa	Shape	plates
Colour	clear_pale_colourless	Size	0.04 × 0.12 × 0.16 mm
D_x	1.41 Mg m^{-3}	F000	720.000
μ	1.387 mm^{-1}		
Absorption correction	multi-scan		
T_{min}	0.86	T_{max}	0.95

Data Collection

Diffractometer	multi-scan
Scan type	ω scans
Reflections measured	9459
Independent reflections	6188
Rint	0.0270
$θ_{max}$	72.4365
h =	-7 → 5
k =	-16 → 16
l =	-23 → 21

Refinement

$Δρ_{min}$ =	-0.27 e $Å^{-3}$
$Δρ_{max}$ =	0.24 e $Å^{-3}$
Reflections used	6162
Cutoff: I >	-3.00σ(I)
Parameters refined	452

S =	1.12
R-factor	0.031
weighted R-factor	0.049
Δ/σ_{max}	0.0005
Flack parameter	0.012(13)
Refinement on	F^2
w =	$w' \times [1 - (\Delta F_{obs} / 6 \times \Delta F_{est})^2]^2$
w′ =	$[P_0T_0'(x) + P_1T_1'(x) + ...P_{n-1}T_{n-1}'(x)]^{-1}$, where P_i are the coefficients of a Chebychev series in $t_i(x)$, and $x = F_{calc}^2/F_{calc}^2{}_{max}$.
$P_0 - P_{n-1}$ =	301. 409. 141.

Pyrazine Dimer 12

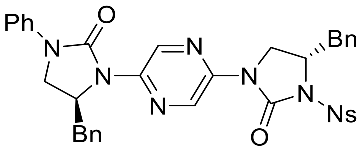

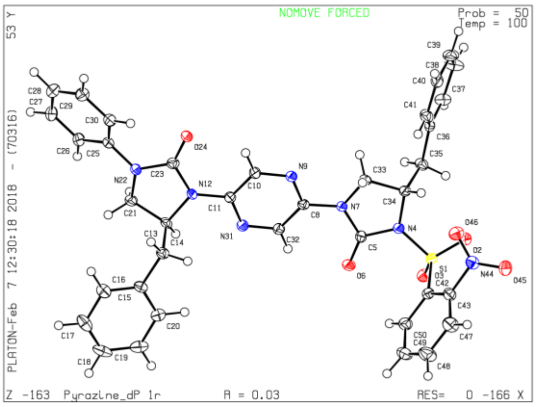

Crystal Data

a = 5.5579(2) Å   α = 68.460(3)°
b = 11.6199(4) Å β = 86.988(3)°
c = 13.7205(5) Å γ = 76.575(3)°

Volume	801.17(5) Å^3	Crystal Class	triclinic
Space group	P 1	Z =	1
Formula	$C_{36}H_{31}N_7O_6S_1$	M_r	689.75
Cell determined from	7505 reflections	Cell θ range =	6 - 72°
Temperature	100K		
Shape	blocks		
Colour	clear_pale_colourless	Size	0.10 × 0.14 × 0.18 mm
D_x	1.43 Mg m^{-3}	F000	360.000
μ	1.406 mm^{-1}		
Absorption correction	multi-scan		
T_{min}	0.76	T_{max}	0.87

Data Collection

Diffractometer	multi-scan
Scan type	ω scans
Reflections measured	10556
Independent reflections	5352
Rint	0.0242
θ_{max}	72.5887
h =	-6 → 6
k =	-14 → 14
l =	-16 → 16

Refinement

$\Delta\rho_{min}$ =	-0.18 e Å^{-3}
$\Delta\rho_{max}$ =	0.22 e Å^{-3}
Reflections used	5327
Cutoff: I >	-2.50σ(I)
Parameters refined	452

S =	0.89
R-factor	0.029
weighted R-factor	0.066
Δ/σ_{max}	0.0003
Flack parameter	0.004(11)
Refinement on	F^2
w =	$w' \times [1 - (\Delta F_{obs} / 6 \times \Delta F_{est})^2]^2$
w′ =	$[P_0T_0'(x) + P_1T_1'(x) + ...P_{n-1}T_{n-1}'(x)]^{-1}$, where P_i are the coefficients of a Chebychev series in $t_i(x)$, and $x = F_{calc}^2/F_{calc}^2{}_{max}$.
$P_0 - P_{n-1}$ =	0.174E+04 0.286E+04 0.185E+04 823. 245.

Circular Dichroism

Samples were prepared at a concentration of 100 μM in the solvent indicated, and 300 μL of the resulting solution was placed in a 1 mm path length quartz cuvette. Data were acquired between 240 and 400 nm, since below 240 nm $CHCl_3$ absorbs significantly, and preliminary analysis indicated no peaks occurred above 400 nm. A data pitch of 1 nm and continuous scanning speed of 25 nm·min^{-1} were employed. Each spectrum was acquired twice, and the data averaged. The resulting raw data was smoothed using the Savitsky-Golay method, with a window size of 25 nm.

Homo-Pyrimidine Series

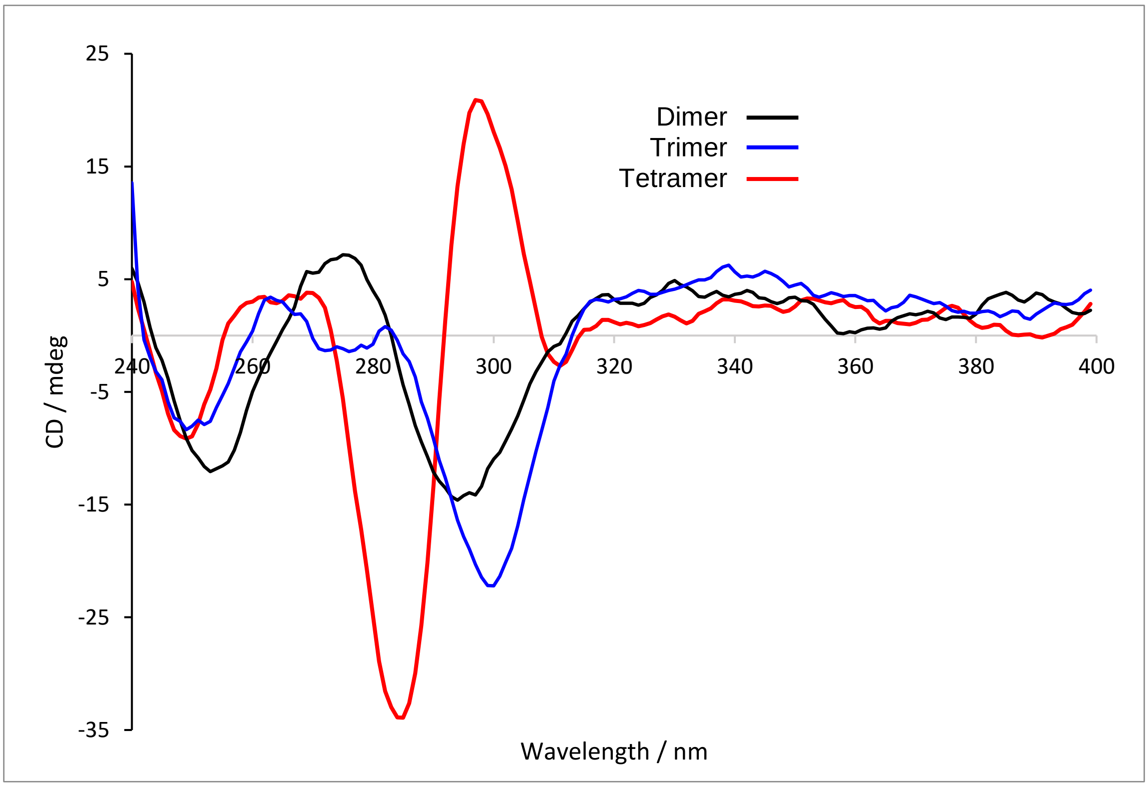

Figure S2. CD spectra of pyrimidine dimer **10** (black) and trimer **13** (blue) and tetramer **16** (red); $CHCl_3$, 100 μM.

Dimer **10** and trimer **13** display qualitatively similar spectra, with strong minima for each at ~250 nm and ~300 nm. Tetramer **16** retains the minimum at 250 nm observed for dimer and trimer **10** and **13**, but gains a strong new minimum at ~280 nm and maximum at ~300 nm. Considered alongside the computation and ROESY data outlined in the main text (**Figure 4**), this may suggest that the minimum at ~280 nm and maximum at ~300 nm observed for the tetramer are diagnostic of the formation of a (*P*)-helix in the pyrimidine-(*S*)-imidazolidin-2-one oligomers.

Homo-Pyridazine Series

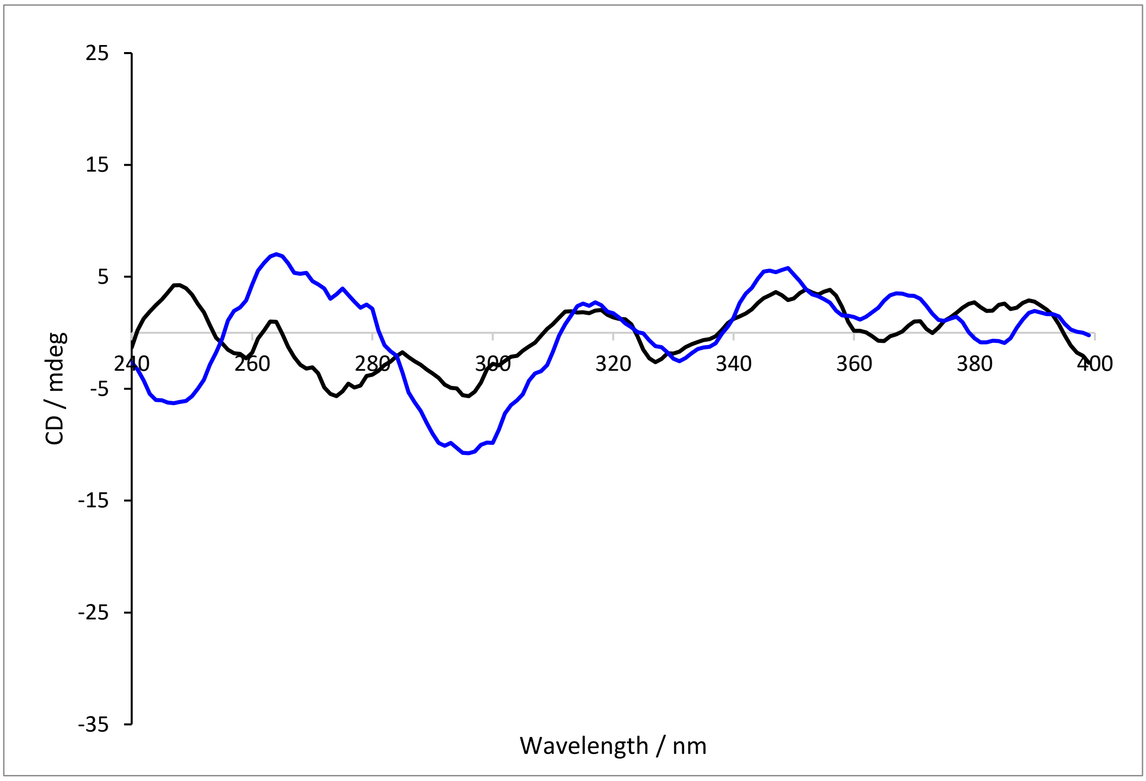

Figure S3. CD spectra of pyridazine dimer **11** (black) and trimer **14** (blue); $CHCl_3$, 100 μM.

The intensity of CD observed for both the pyridazine dimer **11** and trimer **14** was significantly less than that of the equivalent pyrimidines, making them challenging to interpret. Trimer **14** exhibits a minimum at ~295 nm and a maximum at ~265 nm, but dimer **11** shows no clear peaks.

Homo-Pyrazine Series

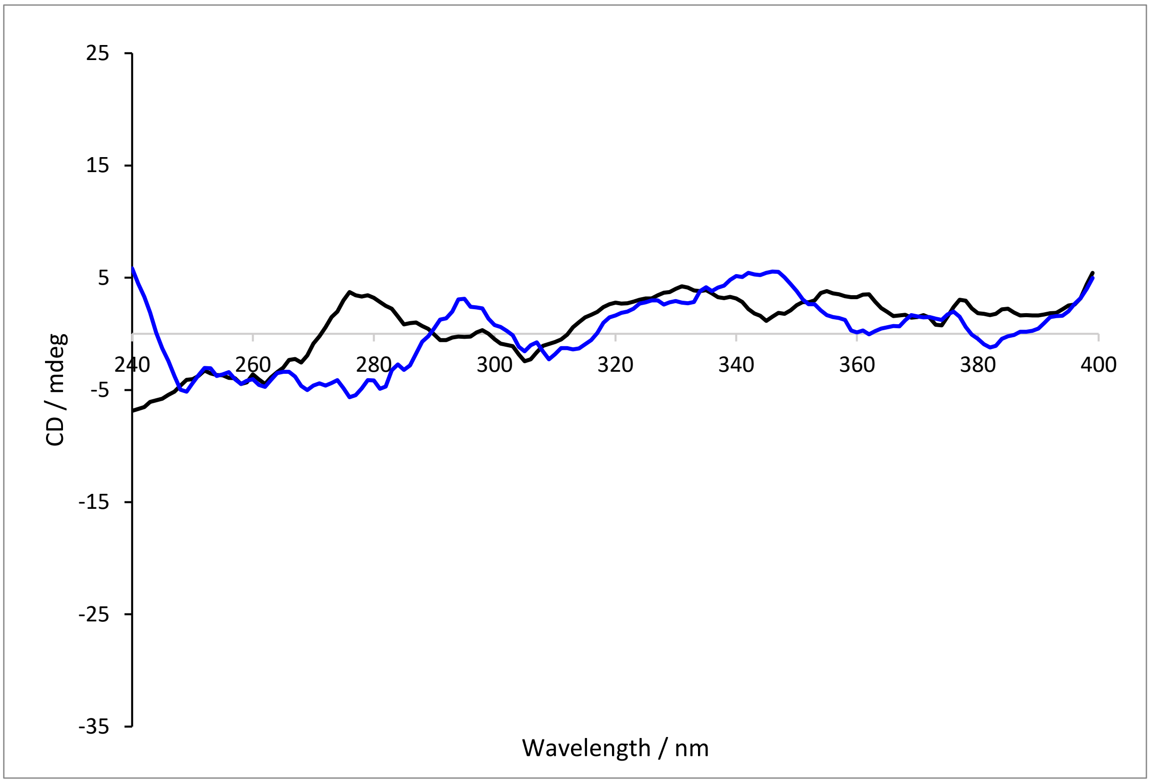

Figure S4. CD spectra of pyrazine dimer **12** (black) and trimer **15** (blue); $CHCl_3$, 100 μM.

The CD spectra of both pyrazine dimer **12** and trimer **15** display no strong or diagnostic features common to both. There may be a maximum below 240 nm for trimer **15**, but due to the absorption characteristics of the solvent this could not be fully observed.

Mixed Pyridazine-Pyrazine Trimer 21

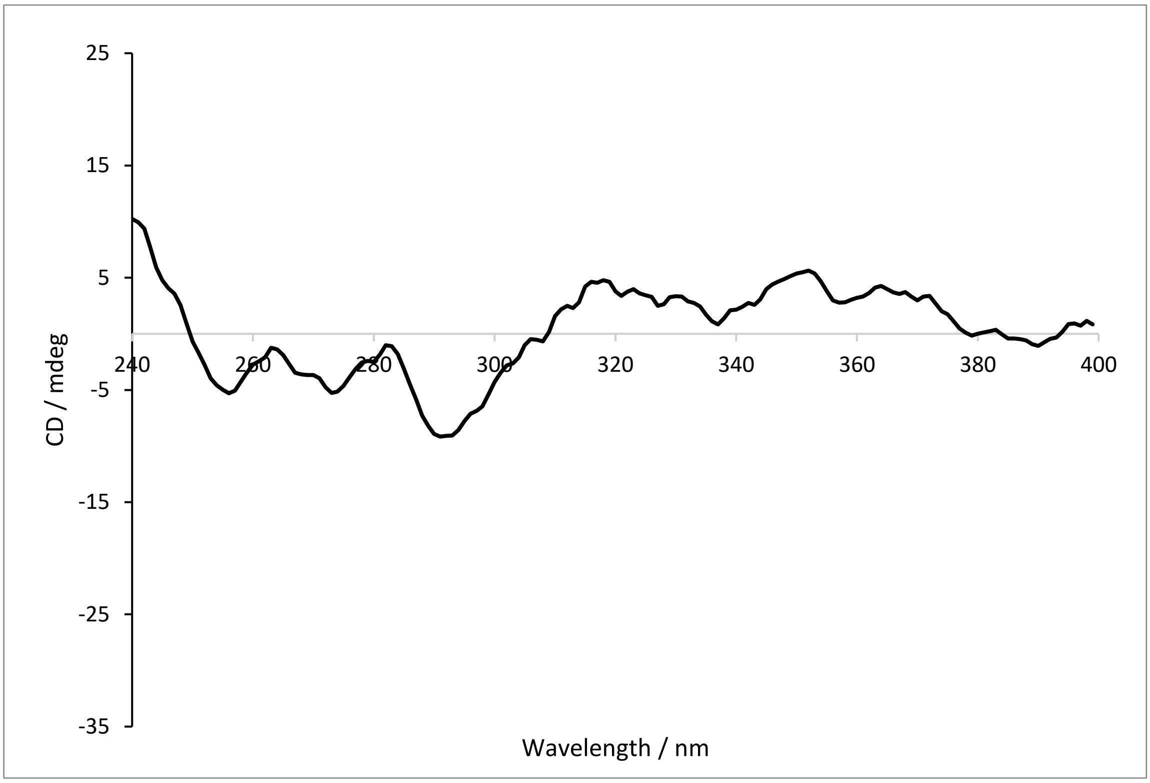

Figure S5. CD spectrum of pyridazine-pyrazine trimer **21**; $CHCl_3$, 100 μM.

Trimer **21** gave only weak CD peaks, consistent with the homo-oligomers in the pyridazine and pyrazine series. The only major features are a minimum at ~290 nm, which is in agreement with the putative minimum observed for the pyridazine series at ~295 nm, and a potential maximum below 240 nm, consistent with that suggested for pyrazine homo-trimer **15**.

Mixed Pyrimidine-Pyridazine Trimer 22

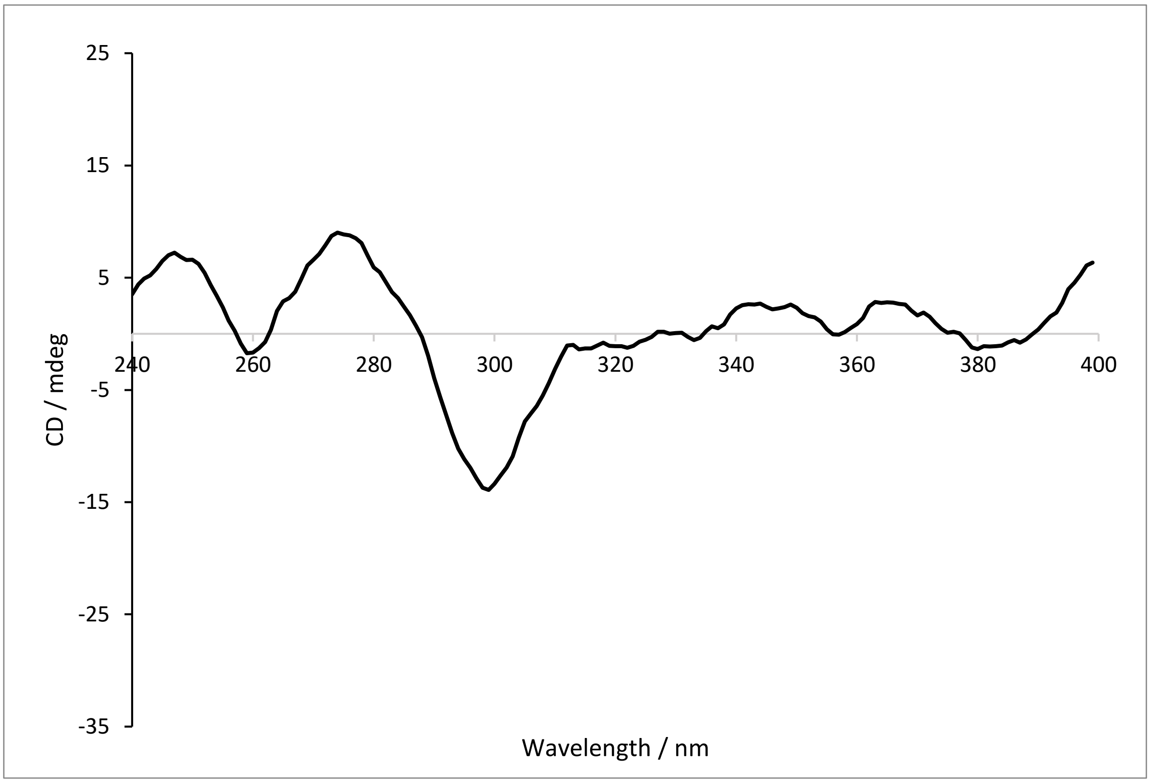

Figure S6. CD spectrum of pyrimidine-pyridazine trimer **22**; $CHCl_3$, 100 *μ*M.

The CD spectrum of pyrimidine-pyridazine trimer **22** displays a strong minimum at ~300 nm, consistent with those observed for the pyrimidine homo-dimer and trimer. Its overall appearance resembles an overlay of the pyrimidine and pyridazine dimers **10** and **11**. A maximum was also observed at ~275 nm.

Mixed Pyrimidine-Pyridazine Pentamer 20

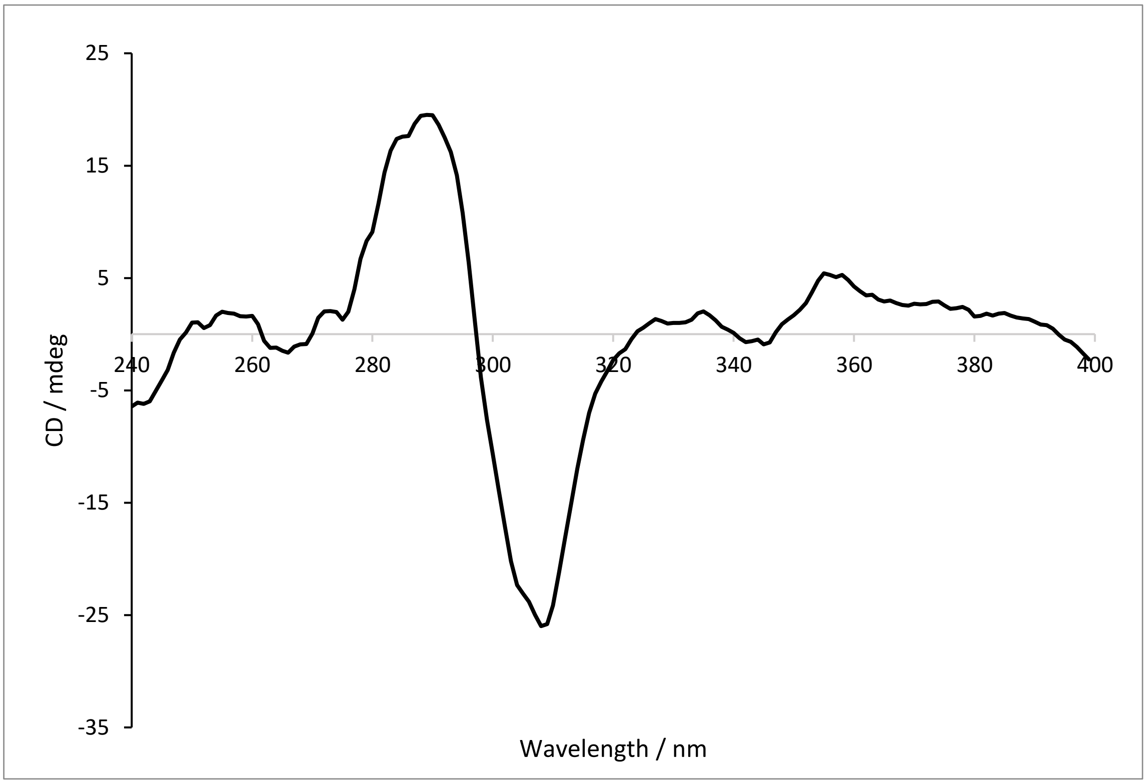

Figure S6. CD spectrum of pyrimidine-pyridazine pentamer **20**; $CHCl_3$, 100 μM.

The CD spectrum of pyrimidine-pyridazine pentamer **20** displays a strong minimum at ~310 nm, consistent with those observed in this region for the pyrimidine homo-dimer and trimer, and the pyrimidine-pyridazine trimer **22**. A strong maximum was also observed at ~290 nm, such that the spectrum of **20** resembles that of **22**, slightly red-shifted.

Computation

Tetramer 16

The lowest energy conformation of tetramer **16** was determined computationally to allow comparison with the long-range nOe data obtained (Figure S7). This was conducted using the open-source VegaZZ software package.[8] Unconstrained conformational searching was conducted using molecular mechanics: the AMMP algorithm under Boltzmann jump conditions (*T* =800 K, 50000 steps, RMSD = 60, dielectric constant = 1) with the SP4 force field and standard bonding, angle, and non-bonding interaction potentials. This generated an ensemble of conformers, the lowest energy of which possessed (*P*)-helicity. The lowest energy (*M*)-conformer was located with ΔH_f ~ 32 kJ·mol^{-1} higher energy than the (*P*)-helical conformer. The lowest energy (*P*)- and (*M*)-structures thus obtained were minimised using semi-empirical methods (MOPAC 2016,[9] PM7 method[10]). This indicated that the (*P*)-helix lies 39 kJ·mol^{-1} lower in energy than the (*M*)-helix.

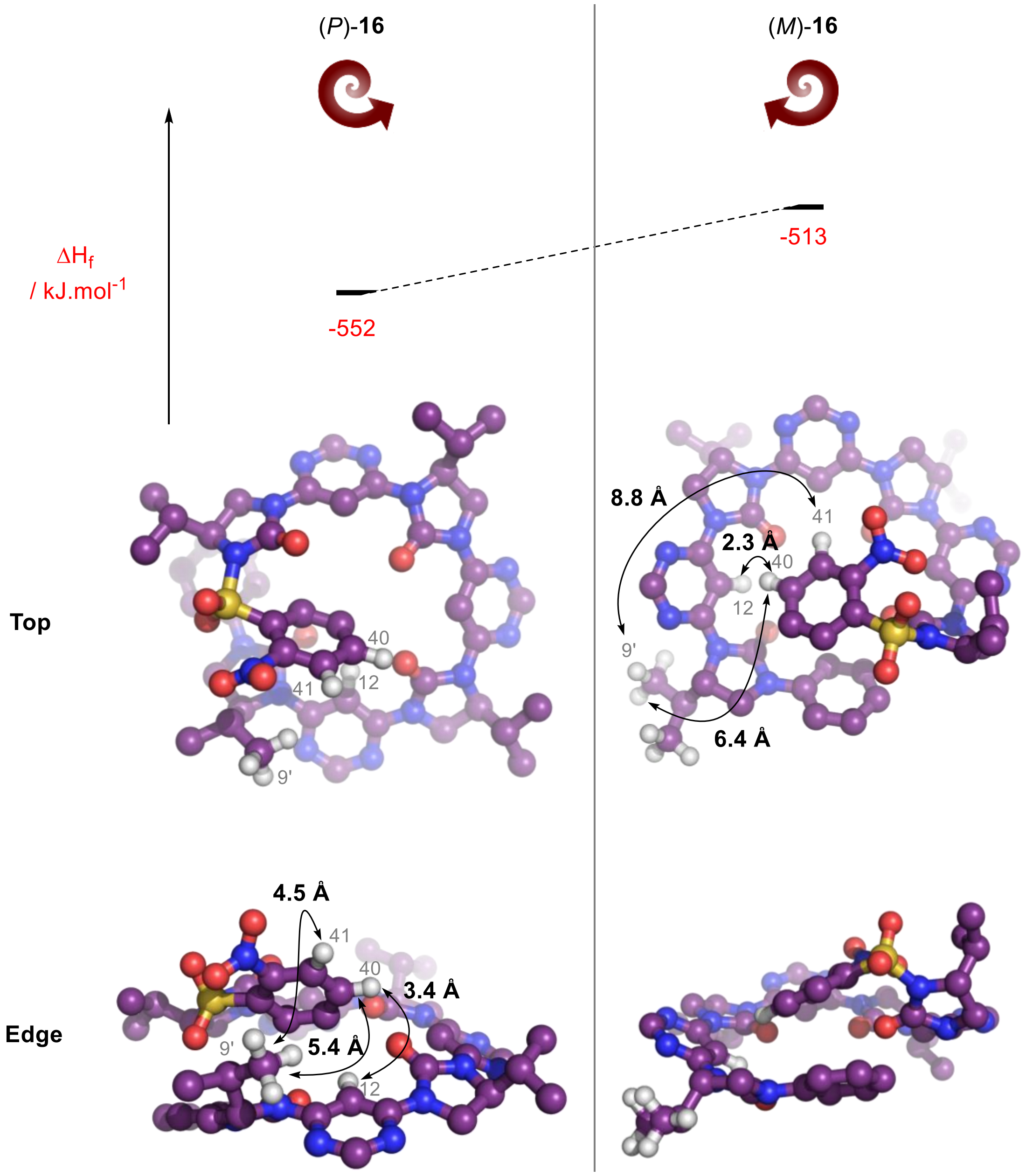

Figure S7. Computed lowest energy structures of (*P*)- (left) and (*M*)- (right) conformations of tetramer **16**. Top – relative energies of the two conformations; middle – top-oriented view; bottom – edge-oriented view. Atoms are labelled in grey. Distances are marked between H atoms which exhibited cross-peaks in the ROESY spectrum of **16**. Distances relating to the side-chain methyl group are the average of the three hydrogen atoms.

Both helical structures are consistent with the dipole repulsion hypothesis, and the (*P*)-conformer has H9'↔H41 and H12↔H40 distances that would be expected to produce nOe correlations (Figure S7, bottom left). The fact that these nOes are observed (along with H9'↔H40) is consistent with **16** adopting an ensemble of conformers heavily biased towards those where the termini are in close contact and the helix adopted is of (*P*)-stereochemistry. The alternative (*M*)-helix is expected to give H9'↔H40 and H9'↔H41 distances much greater than the detection limit of the nOe experiment (Figure S7, middle right).

Atomic Coordinates of (*P*)-helical energy minimum of 16

1	C	2.541814	-3.33046	-5.86904	45	C	1.472411	-0.56596	-8.43803
2	C	1.590112	-2.42747	-6.7187	46	S	-2.89743	3.042116	-0.89756
3	N	0.972819	-1.52592	-5.70905	47	C	-4.3213	1.294529	-4.31901
4	C	1.533471	-1.70774	-4.42078	48	C	-4.5908	2.152393	-3.03319
5	N	2.477828	-2.75763	-4.50754	49	N	-3.22931	2.510077	-2.55846
6	O	1.27802	-1.05925	-3.44205	50	C	-2.24109	2.0602	-3.43393
7	C	2.695299	-4.14206	2.441349	51	N	-2.8507	1.238342	-4.41891
8	C	3.816034	-4.30578	1.355846	52	O	-1.06315	2.309947	-3.37228
9	N	3.372241	-3.40118	0.258364	53	C	-0.35066	-1.07652	-5.82895
10	C	2.311748	-2.55977	0.692382	54	N	-1.12403	-1.69336	-6.77185
11	N	1.855171	-3.0443	1.927083	55	C	-2.40908	-1.29654	-6.87008
12	O	1.914045	-1.57326	0.131188	56	N	-2.99442	-0.3567	-6.10339
13	C	0.755828	-2.53067	2.605818	57	C	-2.20872	0.285739	-5.182
14	C	3.483876	-3.73872	-1.09154	58	C	-0.84736	-0.04514	-5.02188
15	N	4.155154	-4.89344	-1.382	59	C	-5.46272	3.383468	-3.38267
16	C	4.274022	-5.22914	-2.68351	60	C	-5.75179	4.250141	-2.15622
17	N	3.740943	-4.54975	-3.71725	61	C	-6.79478	2.88877	-3.96608
18	C	3.053054	-3.40124	-3.42391	62	O	-2.73708	4.463072	-0.94394
19	C	2.938833	-2.93164	-2.10507	63	O	-3.96556	2.422255	-0.14822
20	C	-0.21595	-1.75864	1.950144	64	C	-1.37035	2.271527	-0.4946
21	C	-1.30282	-1.31441	2.721151	65	C	-1.10035	0.992774	-0.98027
22	N	-1.39362	-1.60165	4.053759	66	C	0.168237	0.424322	-0.83749
23	C	-0.41325	-2.35299	4.594249	67	C	1.170859	1.116482	-0.16683
24	N	0.654765	-2.84513	3.930124	68	C	0.893208	2.358925	0.403649
25	N	-2.30891	-0.53943	2.142071	69	C	-0.37445	2.916508	0.245233
26	C	-2.8556	-0.92985	0.883327	70	N	-0.60727	4.186243	0.942709
27	N	-4.19644	-0.54736	0.844583	71	O	-1.6426	4.301435	1.576017
28	C	-4.54405	0.291815	2.013473	72	O	0.280051	5.019914	0.920735
29	C	-3.29491	0.219941	2.95438	73	H	2.217137	-4.39289	-5.8689
30	O	-2.23141	-1.5101	0.027624	74	H	3.591566	-3.31206	-6.23248
31	C	2.379026	-1.59583	-7.76117	75	H	0.807632	-3.04183	-7.2323
32	C	2.960146	-2.54426	-8.81519	76	H	2.090713	-5.06525	2.558217
33	C	5.19116	-3.84713	1.902726	77	H	3.100982	-3.88458	3.443596
34	C	5.648835	-4.83211	2.983665	78	H	3.879123	-5.36194	0.994203
35	C	6.23292	-3.7869	0.78456	79	H	4.83704	-6.14842	-2.91802
36	C	-4.99519	-0.56597	-0.31572	80	H	2.443582	-1.97992	-1.88383
37	C	-4.62978	-1.35403	-1.421	81	H	-0.15318	-1.52006	0.883344
38	C	-5.48249	-1.44584	-2.5143	82	H	-0.49365	-2.59377	5.66789
39	C	-6.69541	-0.75904	-2.53145	83	H	-5.4478	-0.1112	2.507841
40	C	-7.04881	0.036473	-1.44365	84	H	-4.74551	1.344871	1.691468
41	C	-6.20734	0.146136	-0.34175	85	H	-3.52918	-0.32516	3.901003
42	C	-2.76146	1.639278	3.270677	86	H	3.209098	-1.0573	-7.24174
43	C	-3.78776	2.365283	4.146986	87	H	3.536737	-1.98703	-9.56347
44	C	-1.41279	1.587432	3.989644	88	H	3.634085	-3.28968	-8.38165

89	H	2.169302	-3.07984	-9.35364	109	H	1.148359	0.209817	-7.73224
90	H	5.082775	-2.82767	2.347247	110	H	1.988149	-0.05913	-9.26014
91	H	6.617958	-4.52994	3.399002	111	H	0.567167	-1.03031	-8.8487
92	H	4.946429	-4.89107	3.821166	112	H	-4.76021	0.278184	-4.23468
93	H	5.775381	-5.84312	2.578581	113	H	-4.73546	1.753339	-5.24123
94	H	7.231897	-3.5759	1.17862	114	H	-5.10683	1.534057	-2.25015
95	H	5.995456	-3.00184	0.055888	115	H	-3.03641	-1.79423	-7.62832
96	H	6.284559	-4.7326	0.228898	116	H	-0.20888	0.474604	-4.30032
97	H	-3.69114	-1.91402	-1.42933	117	H	-4.92806	4.002086	-4.14375
98	H	-5.19982	-2.06946	-3.36362	118	H	-5.98757	3.649258	-1.26862
99	H	-7.36177	-0.84736	-3.38463	119	H	-6.60274	4.917267	-2.32809
100	H	-7.99282	0.579353	-1.45222	120	H	-4.90245	4.90383	-1.90581
101	H	-6.49074	0.796932	0.481454	121	H	-7.46902	3.729616	-4.16642
102	H	-2.63466	2.20185	2.310026	122	H	-6.66878	2.355025	-4.91301
103	H	-4.76229	2.451589	3.656313	123	H	-7.31084	2.217856	-3.26935
104	H	-3.93082	1.861457	5.108174	124	H	-1.86432	0.395453	-1.49243
105	H	-3.4517	3.38884	4.360764	125	H	0.370268	-0.56251	-1.27388
106	H	-1.09555	2.589493	4.301174	126	H	2.172347	0.687311	-0.08234
107	H	-0.62577	1.18472	3.342472	127	H	1.666151	2.892426	0.960053
108	H	-1.45533	0.958017	4.886542					

MOPAC 2016 Output for (*P*)-helical energy minimum of 16

PM7 CALCULATION
MOPAC2016 (Version: 16.230W)
Tue Feb 13 12:44:24 2018
No. of days remaining = 0

```
FINAL HEAT OF FORMATION =       -131.87209 KCAL/MOL =    -551.75284 KJ/MOL

TOTAL ENERGY            =    -11968.78766 EV
ELECTRONIC ENERGY       =   -184729.14420 EV  POINT GROUP:     C1
CORE-CORE REPULSION     =    172760.35654 EV
COSMO AREA              =        739.56 SQUARE ANGSTROMS
COSMO VOLUME            =       1127.92 CUBIC ANGSTROMS

GRADIENT NORM           =          0.19529
IONIZATION POTENTIAL    =          8.785680 EV
HOMO LUMO ENERGIES (EV) =         -8.786 -0.870
NO. OF FILLED LEVELS    =        188
MOLECULAR WEIGHT        =       1002.1182

MOLECULAR DIMENSIONS (Angstroms)

  Atom       Atom       Distance
  H   121    H    91    17.93143
  H    98    O    72    12.57902
  H    93    H   121     0.63718

SCF CALCULATIONS        =       1847

WALL-CLOCK TIME         =  7 MINUTES AND 48.777 SECONDS
COMPUTATION TIME        = 30 MINUTES AND 33.359 SECONDS
```

Atomic Coordinates of lowest energy (*M*)-helical conformer of 16

1	C	0.75700	5.30300	-2.26200	52	O	2.40500	-1.16600	-1.15000
2	C	1.39900	4.61600	-3.50900	53	C	2.53100	2.36800	-3.41100
3	N	1.43800	3.18000	-3.10800	54	N	3.49900	2.93500	-4.19600
4	C	0.59100	2.93000	-1.99800	55	C	4.56800	2.17900	-4.50800
5	N	0.10900	4.18100	-1.55000	56	N	4.80100	0.93400	-4.04500
6	O	0.30500	1.85900	-1.53600	57	C	3.85100	0.37900	-3.23700
7	C	-4.60400	1.96700	3.45400	58	C	2.64600	1.04800	-2.95200
8	C	-4.48100	3.22500	2.53000	59	C	7.03800	-0.67800	-0.86000
9	N	-3.40800	2.82400	1.57800	60	C	7.80300	-1.14400	0.38200
10	C	-3.14900	1.43700	1.63300	61	C	6.55800	0.76100	-0.69100
11	N	-3.94900	0.90400	2.66700	62	O	4.64000	-2.99900	1.54900
12	O	-2.38000	0.81700	0.94600	63	O	5.12500	-0.57700	1.84500
13	C	-3.98400	-0.43600	3.02100	64	C	2.65700	-1.29700	1.46500
14	C	-2.52600	3.73200	0.99600	65	C	1.90200	-2.35100	1.97500
15	N	-2.64000	5.02700	1.42200	66	C	0.53400	-2.19700	2.21500
16	C	-1.85700	5.94700	0.82200	67	C	-0.10000	-1.00200	1.90200
17	N	-0.96600	5.69300	-0.15500	68	C	0.63700	0.06700	1.39000
18	C	-0.82400	4.38800	-0.55200	69	C	2.01200	-0.07900	1.21100
19	C	-1.60200	3.35800	0.00700	70	N	2.71300	1.11700	0.73200
20	C	-3.55900	-1.44700	2.14700	71	O	3.60000	0.99200	-0.09900
21	C	-3.51500	-2.75700	2.66100	72	O	2.35600	2.19300	1.17400
22	N	-3.92900	-3.02800	3.94000	73	H	1.51600	5.78400	-1.60900
23	C	-4.40700	-2.00100	4.67000	74	H	0.01500	6.08400	-2.53000
24	N	-4.44800	-0.71200	4.27900	75	H	2.43100	5.00300	-3.70000
25	N	-3.03500	-3.82500	1.92600	76	H	-4.08800	2.10800	4.42700
26	C	-2.32000	-3.65400	0.69200	77	H	-5.65500	1.69300	3.67900
27	N	-1.34600	-4.66200	0.61500	78	H	-4.17000	4.13200	3.10500
28	C	-1.40000	-5.53800	1.80700	79	H	-1.95400	6.99200	1.16100
29	C	-2.73700	-5.14800	2.52500	80	H	-1.50200	2.32100	-0.33300
30	O	-2.59800	-2.83400	-0.13900	81	H	-3.29200	-1.22500	1.10800
31	C	0.51700	4.80100	-4.77000	82	H	-4.77600	-2.23300	5.68300
32	C	0.57000	6.27100	-5.20200	83	H	-0.52900	-5.38400	2.47400
33	C	-5.81600	3.48800	1.78600	84	H	-1.40100	-6.59900	1.48000
34	C	-6.85200	3.99200	2.79600	85	H	-2.60200	-5.06200	3.63100
35	C	-5.64600	4.51800	0.66900	86	H	-0.53500	4.52300	-4.52000
36	C	-0.15300	-4.52700	-0.13700	87	H	-0.05000	6.43700	-6.09100
37	C	-0.05300	-3.56000	-1.14800	88	H	0.20200	6.94800	-4.42500
38	C	1.12400	-3.45000	-1.87900	89	H	1.59000	6.57700	-5.46000
39	C	2.20200	-4.29600	-1.62000	90	H	-6.17100	2.52800	1.33400
40	C	2.09700	-5.25900	-0.62200	91	H	-7.81500	4.17800	2.30500
41	C	0.92500	-5.38800	0.12100	92	H	-6.53900	4.93700	3.25500
42	C	-3.86000	-6.16800	2.20600	93	H	-7.03700	3.27300	3.60000
43	C	-3.55000	-7.49000	2.91700	94	H	-6.60900	4.78200	0.22000
44	C	-5.22100	-5.64300	2.66500	95	H	-5.00600	4.13900	-0.13600
45	C	1.00100	3.91000	-5.91500	96	H	-5.18800	5.44400	1.04100
46	S	4.37100	-1.62400	1.21400	97	H	-0.87600	-2.87500	-1.36800
47	C	5.50100	-1.43500	-2.75000	98	H	1.20600	-2.67900	-2.65000
48	C	5.90200	-1.66000	-1.24300	99	H	3.12200	-4.19600	-2.19000
49	N	4.60700	-1.53100	-0.53100	100	H	2.94100	-5.91400	-0.40800
50	C	3.58300	-1.16600	-1.40300	101	H	0.86800	-6.15600	0.88300
51	N	4.12300	-0.89500	-2.71600	102	H	-3.89400	-6.33500	1.10200

103	H	-2.59400	-7.92000	2.60400	116	H	1.84600	0.56400	-2.38000
104	H	-3.52400	-7.36400	4.00600	117	H	7.76200	-0.70100	-1.72000
105	H	-4.32300	-8.23700	2.70000	118	H	7.95800	-2.22600	0.40000
106	H	-6.00400	-6.39700	2.54300	119	H	7.28700	-0.86400	1.31400
107	H	-5.52300	-4.75800	2.09100	120	H	8.78900	-0.66800	0.43200
108	H	-5.20300	-5.34400	3.72200	121	H	5.91400	1.08300	-1.51600
109	H	0.86800	2.84700	-5.68100	122	H	7.40100	1.45700	-0.63900
110	H	0.45300	4.11300	-6.84000	123	H	5.98200	0.89900	0.23900
111	H	2.07000	4.06100	-6.11900	124	H	2.36100	-3.32600	2.18700
112	H	6.21100	-0.76700	-3.28100	125	H	-0.03400	-3.02400	2.63400
113	H	5.47500	-2.39300	-3.30800	126	H	-1.17800	-0.88900	2.04200
114	H	6.26200	-2.71200	-1.10100	127	H	0.12800	1.00100	1.11600
115	H	5.32900	2.62400	-5.17100					

MOPAC 2016 Output for lowest energy (*M*)-helical conformer of 16

PM7 CALCULATION
MOPAC2016 (Version: 16.230W)
Thu Apr 12 09:23:13 2018
No. of days remaining = 0

FINAL HEAT OF FORMATION = -122.50936 KCAL/MOL = -512.57916 KJ/MOL

TOTAL ENERGY = -11968.45499 EV
ELECTRONIC ENERGY = -183414.43997 EV POINT GROUP: C1
CORE-CORE REPULSION = 171445.98499 EV
COSMO AREA = 745.76 SQUARE ANGSTROMS
COSMO VOLUME = 1575.01 CUBIC ANGSTROMS

GRADIENT NORM = 0.19235
IONIZATION POTENTIAL = 8.688262 EV
HOMO LUMO ENERGIES (EV) = -8.688 -0.938
NO. OF FILLED LEVELS = 188
MOLECULAR WEIGHT = 1002.1182

MOLECULAR DIMENSIONS (Angstroms)

Atom		Atom		Distance
H	105	H	89	17.75605
H	105	H	79	16.16939
H	110	H	82	9.49890

SCF CALCULATIONS = 2015

WALL-CLOCK TIME = 8 MINUTES AND 23.090 SECONDS
COMPUTATION TIME = 33 MINUTES AND 3.141 SECONDS

References

[1] J. Cosier, A. M. Glazer, *J. Appl. Crystallogr.* **1986**, *19*, 105–107.

[2] *CrysAlisPRO*, Oxford Diffraction/Agilent Technologies UK Ltd, Yarnton, England, **n.d.**

[3] L. Palatinus, G. Chapuis, *J. Appl. Crystallogr.* **2007**, *40*, 786–790.

[4] P. W. Betteridge, J. R. Carruthers, R. I. Cooper, K. Prout, D. J. Watkin, *J. Appl. Crystallogr.* **2003**, *36*, 1487.

[5] R. I. Cooper, A. L. Thompson, D. J. Watkin, *J. Appl. Crystallogr.* **2008**, *43*, 1100–1107.

[6] P. C. Knipe, S. Thompson, A. D. Hamilton, *Chem. Commun.* **2016**, *52*, 6521–6524.

[7] J.-I. Yamaguchi, E. Shibuta, Y. Oishi, *Int. J. Org. Chem.* **2014**, *4*, 286–291.

[8] A. Pedretti, L. Villa, G. Vistoli, *J. Comput. Aided. Mol. Des.* **2004**, *18*, 167–173.

[9] J. J. P. Stewart, *MOPAC 2016*, Stewart Computational Chemistry, Colorado Springs, CO (USA), **2016**.

[10] J. J. P. Stewart, *J. Mol. Model.* **2013**, *19*, 1–32.

NMR Spectra

S1

^{1}H NMR

400 MHz

$CDCl_3$

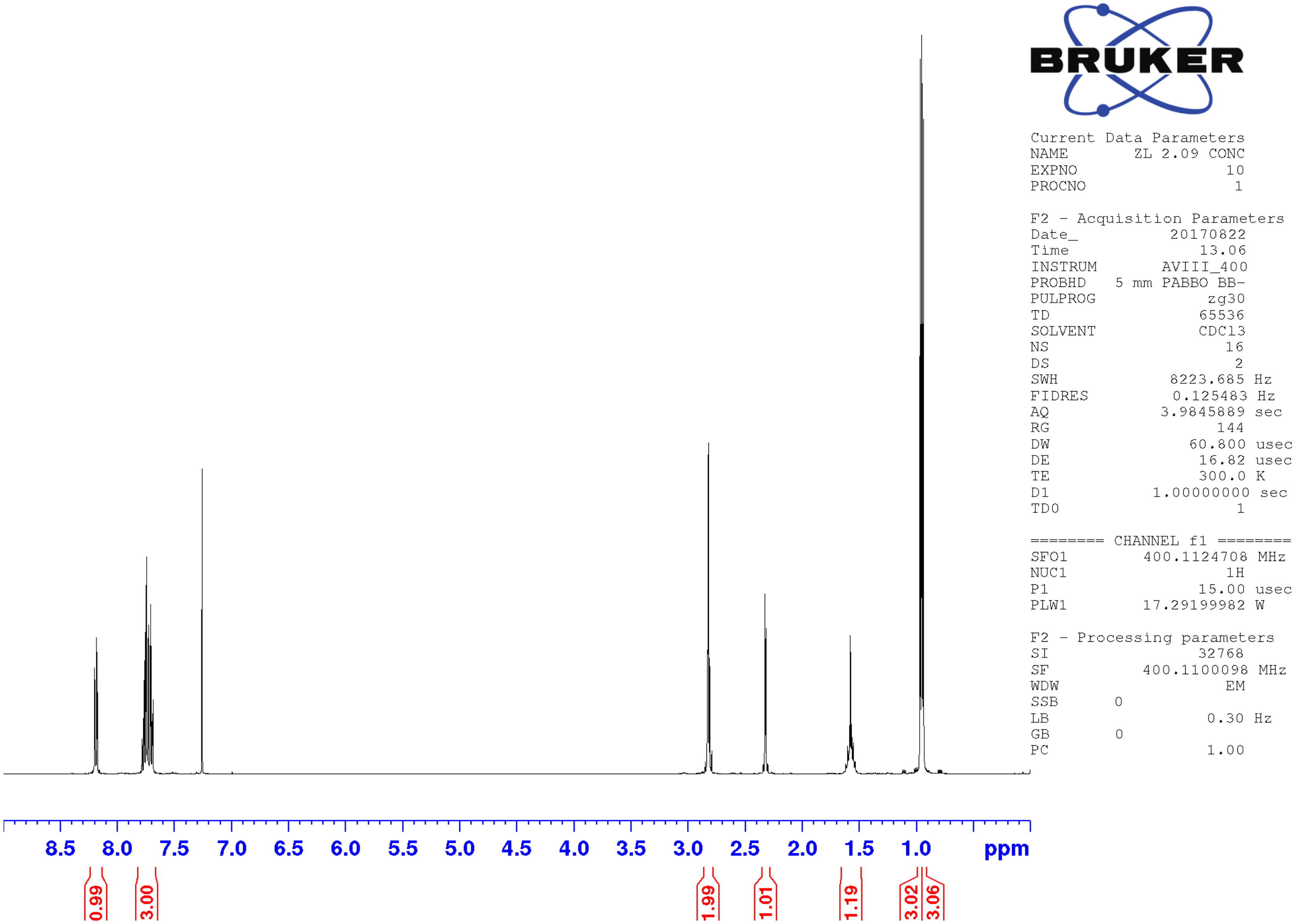

S1

^{13}C NMR

75 MHz

$CDCl_3$

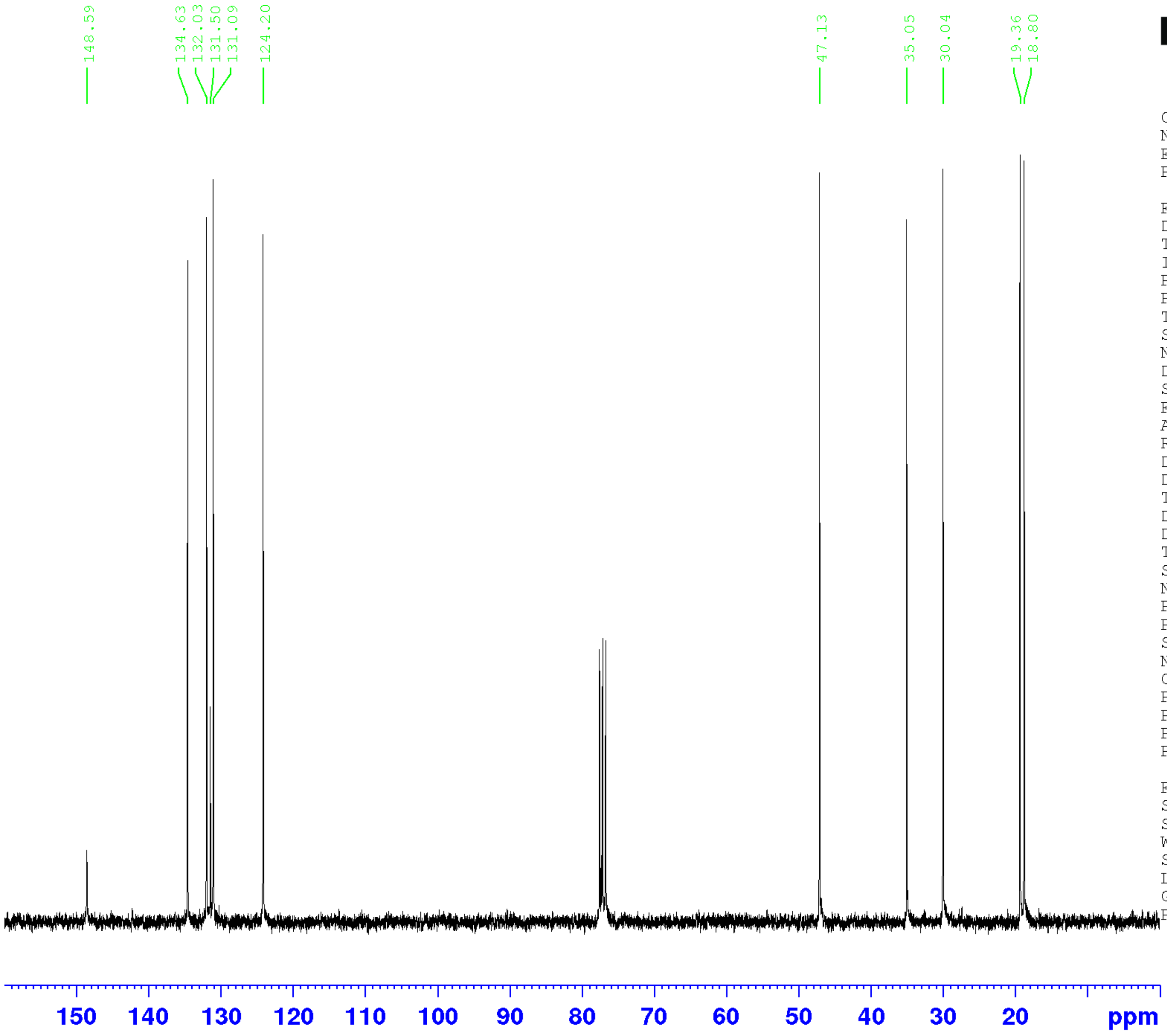

Current Data Parameters
NAME PCK ZL 2.09
EXPNO 11
PROCNO 1

F2 - Acquisition Parameters
Date_ 20180302
Time 8.48 h
INSTRUM spect
PROBHD Z104275_0386 (
PULPROG zgpg30
TD 65536
SOLVENT CDCl3
NS 256
DS 4
SWH 18115.941 Hz
FIDRES 0.552855 Hz
AQ 1.8087935 sec
RG 11.22
DW 27.600 usec
DE 6.50 usec
TE 296.8 K
D1 2.00000000 sec
D11 0.03000000 sec
TD0 1
SFO1 75.4752953 MHz
NUC1 13C
P1 10.00 usec
PLW1 43.88999939 W
SFO2 300.1312005 MHz
NUC2 1H
CPDPRG[2 waltz16
PCPD2 90.00 usec
PLW2 7.33230019 W
PLW12 0.17742001 W
PLW13 0.08924300 W

F2 - Processing parameters
SI 32768
SF 75.4677484 MHz
WDW EM
SSB 0
LB 1.00 Hz
GB 0
PC 1.40

S2

^{1}H NMR

400 MHz

$CDCl_3$

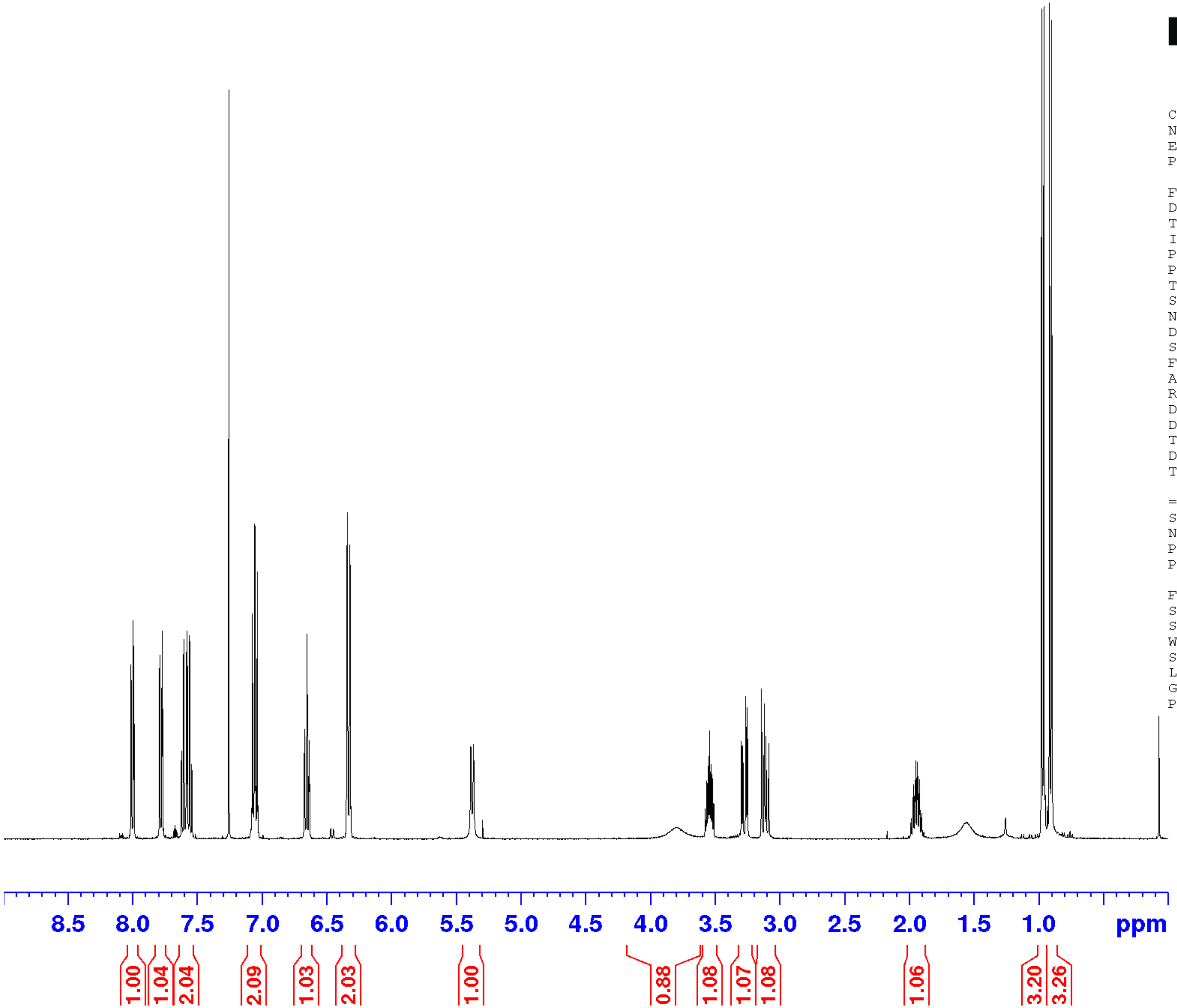


```
Current Data Parameters
NAME      ZL-1-006-Final_Product_2
EXPNO                   10
PROCNO                   1

F2 - Acquisition Parameters
Date_             20161212
Time                 23.14
INSTRUM          AVIII_400
PROBHD    5 mm PABBO BB-
PULPROG               zg30
TD                   65536
SOLVENT              CDCl3
NS                      16
DS                       2
SWH               8223.685 Hz
FIDRES            0.125483 Hz
AQ               3.9845889 sec
RG                     256
DW                  60.800 usec
DE                   16.82 usec
TE                   300.0 K
D1              1.00000000 sec
TD0                      1

======== CHANNEL f1 ========
SFO1           400.1124708 MHz
NUC1                    1H
P1                   15.00 usec
PLW1           17.29199982 W

F2 - Processing parameters
SI                   32768
SF             400.1100098 MHz
WDW                     EM
SSB       0
LB                    0.30 Hz
GB        0
PC                    1.00
```

S2

^{13}C NMR

101 MHz

$CDCl_3$

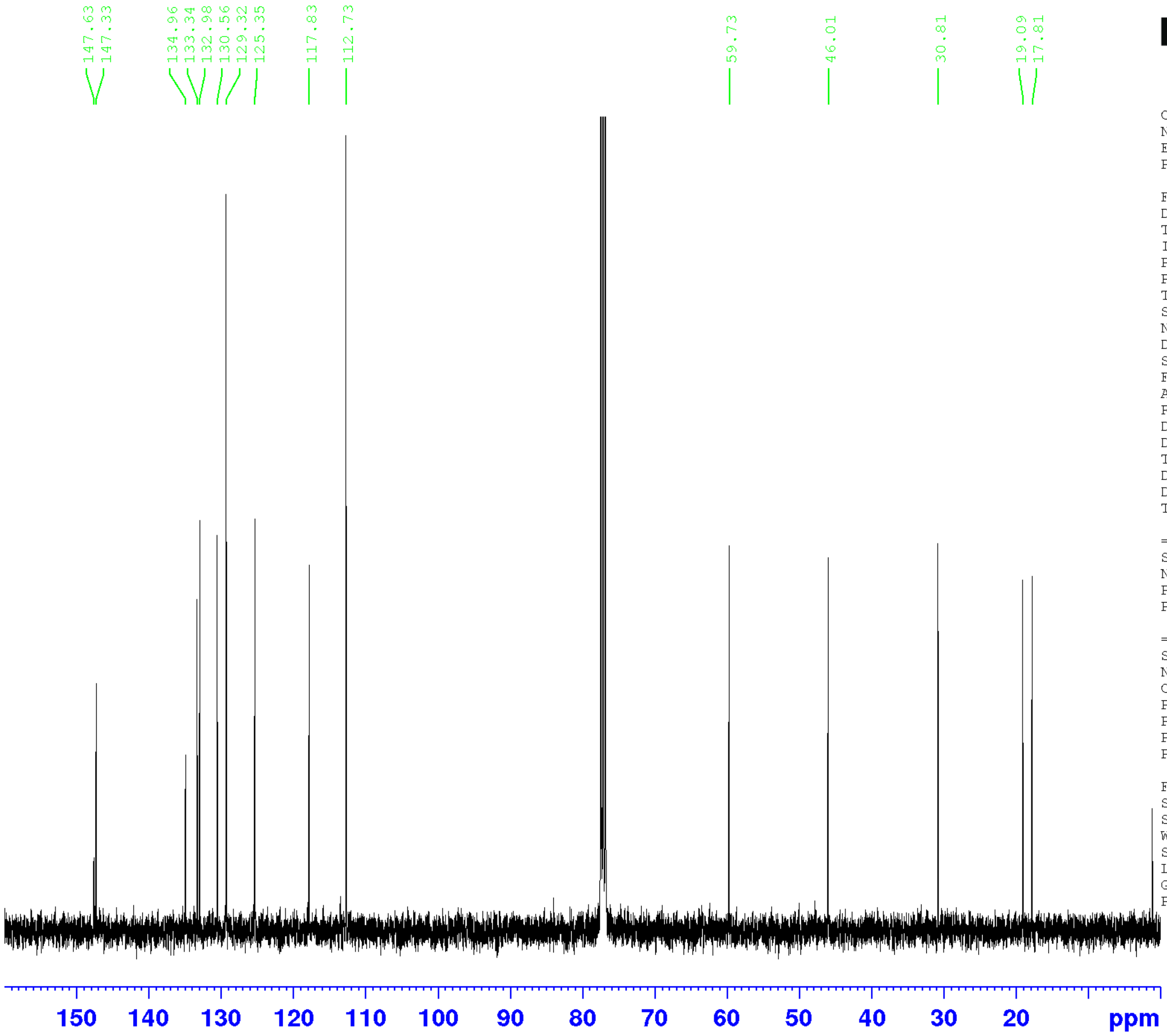


```
Current Data Parameters
NAME       ZL-1-006-Final_Product
EXPNO                        11
PROCNO                        1

F2 - Acquisition Parameters
Date_              20161114
Time                  18.11
INSTRUM           AVIII_400
PROBHD   5 mm PABBO BB-
PULPROG             zgpg30
TD                   65536
SOLVENT              CDCl3
NS                    1024
DS                       4
SWH              24038.461 Hz
FIDRES            0.366798 Hz
AQ               1.3631488 sec
RG                    2050
DW                  20.800 usec
DE                    6.50 usec
TE                   300.0 K
D1              2.00000000 sec
D11             0.03000000 sec
TD0                      1

======== CHANNEL f1 ========
SFO1          100.6177998 MHz
NUC1                  13C
P1                   9.00 usec
PLW1          96.68000031 W

======== CHANNEL f2 ========
SFO2          400.1116004 MHz
NUC2                   1H
CPDPRG[2           waltz16
PCPD2               90.00 usec
PLW2          17.29199982 W
PLW12          0.48032999 W
PLW13          0.24160001 W

F2 - Processing parameters
SI                   32768
SF            100.6077266 MHz
WDW                     EM
SSB        0
LB                    1.00 Hz
GB         0
PC                    1.40
```

S3

^{1}H NMR

400 MHz

$CDCl_3$

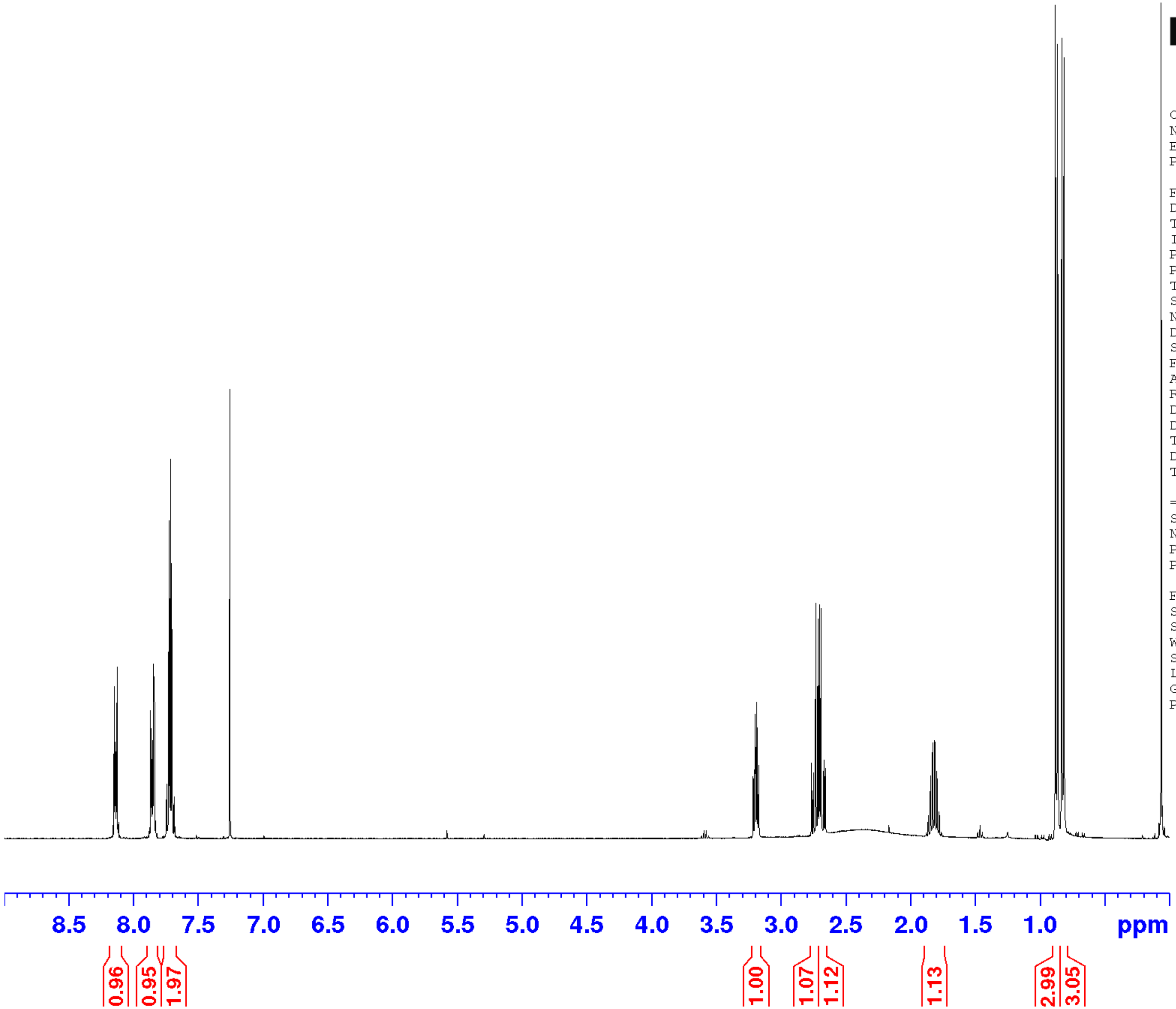


```
Current Data Parameters
NAME      ZL-1-012-Final_Product_2
EXPNO                  10
PROCNO                  1

F2 - Acquisition Parameters
Date_            20161213
Time                 0.50
INSTRUM         AVIII_400
PROBHD    5 mm PABBO BB-
PULPROG              zg30
TD                  65536
SOLVENT             CDCl3
NS                     16
DS                      2
SWH              8223.685 Hz
FIDRES           0.125483 Hz
AQ              3.9845889 sec
RG                    256
DW                 60.800 usec
DE                  16.82 usec
TE                  300.0 K
D1             1.00000000 sec
TD0                     1

======== CHANNEL f1 ========
SFO1          400.1124708 MHz
NUC1                   1H
P1                  15.00 usec
PLW1          17.29199982 W

F2 - Processing parameters
SI                  32768
SF            400.1100098 MHz
WDW                    EM
SSB      0
LB                   0.30 Hz
GB       0
PC                   1.00
```

S3

^{13}C NMR

101 MHz

$CDCl_3$

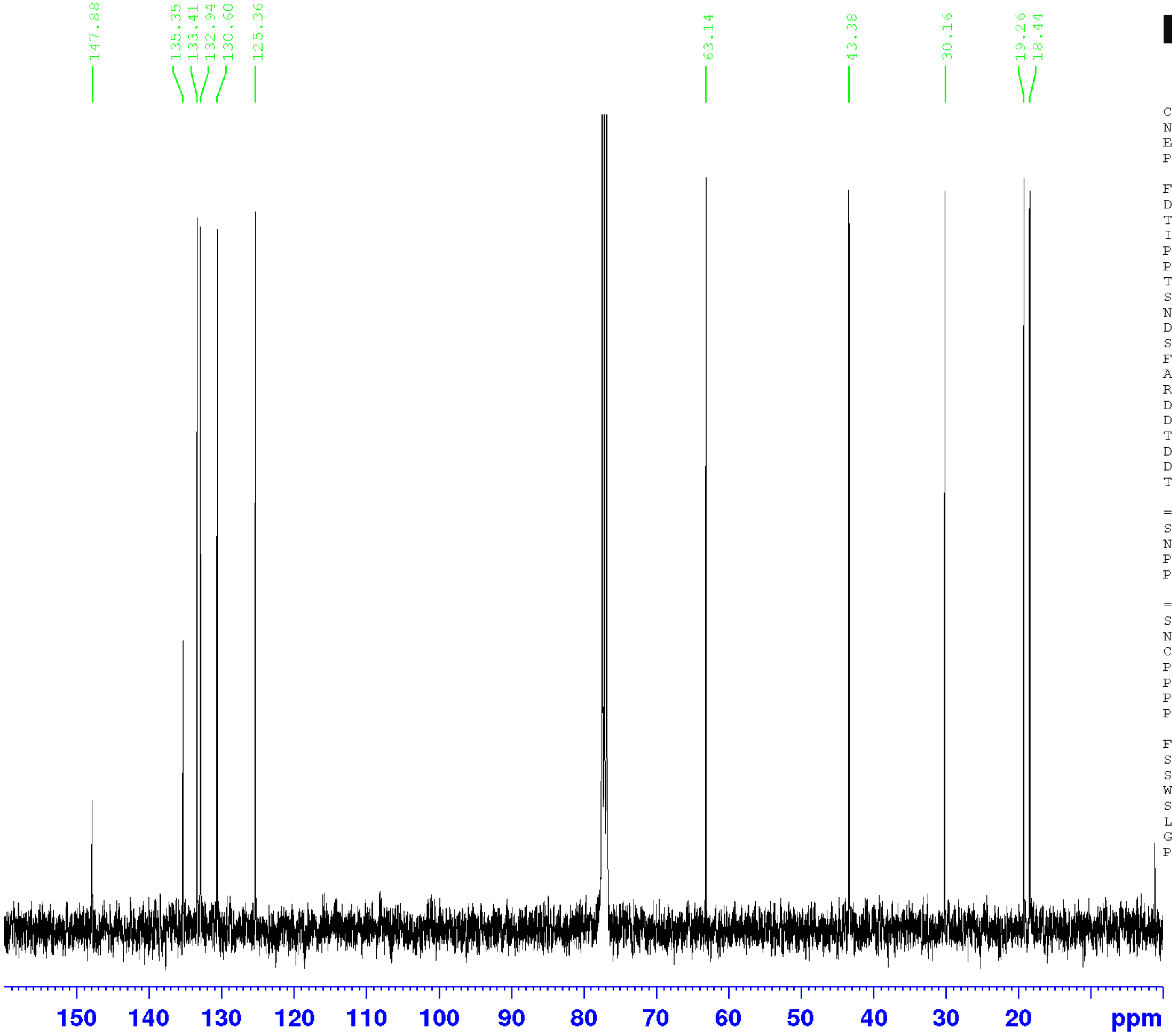


```
Current Data Parameters
NAME      ZL-1-012-Final_Product_3
EXPNO                   10
PROCNO                   1

F2 - Acquisition Parameters
Date_             20161213
Time                  3.17
INSTRUM          AVIII_400
PROBHD    5 mm PABBO BB-
PULPROG            zgpg30
TD                   96150
SOLVENT              CDCl3
NS                    1024
DS                       4
SWH              24038.461 Hz
FIDRES            0.250010 Hz
AQ               1.9999200 sec
RG                    2050
DW                  20.800 usec
DE                    6.50 usec
TE                   300.0 K
D1              1.00000000 sec
D11             0.03000000 sec
TD0                      1

======== CHANNEL f1 ========
SFO1           100.6178003 MHz
NUC1                   13C
P1                    9.00 usec
PLW1           96.68000031 W

======== CHANNEL f2 ========
SFO2           400.1116004 MHz
NUC2                    1H
CPDPRG[2            waltz64
PCPD2                90.00 usec
PLW2           17.29199982 W
PLW12           0.48032999 W
PLW13           0.24160001 W

F2 - Processing parameters
SI                  131072
SF             100.6077274 MHz
WDW                     EM
SSB       0
LB                    2.00 Hz
GB        0
PC                    1.40
```

2

^{1}H NMR

400 MHz

d_6-DMSO

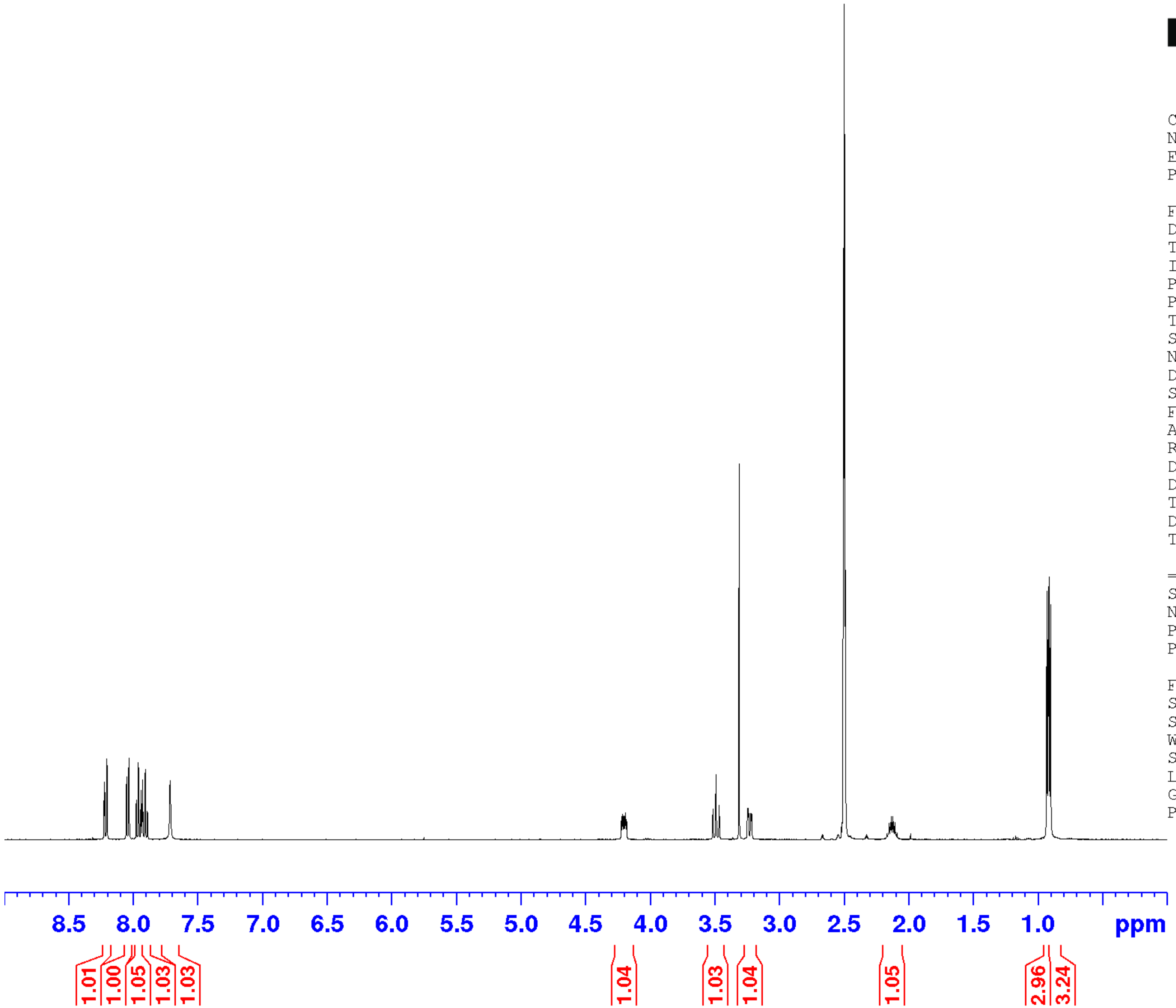


```
Current Data Parameters
NAME      ZL-1-019-Final
EXPNO                 10
PROCNO                 1

F2 - Acquisition Parameters
Date_           20170323
Time               18.05
INSTRUM        AVIII_400
PROBHD    5 mm PABBO BB-
PULPROG             zg30
TD                 65536
SOLVENT             DMSO
NS                    16
DS                     2
SWH             8223.685 Hz
FIDRES          0.125483 Hz
AQ             3.9845889 sec
RG                   228
DW                60.800 usec
DE                 16.82 usec
TE                 300.0 K
D1            1.00000000 sec
TD0                    1

======== CHANNEL f1 ========
SFO1         400.1124708 MHz
NUC1                  1H
P1                 15.00 usec
PLW1         17.29199982 W

F2 - Processing parameters
SI                 32768
SF           400.1100029 MHz
WDW                   EM
SSB       0
LB                  0.30 Hz
GB        0
PC                  1.00
```

2

^{13}C NMR

101 MHz

d_6-DMSO

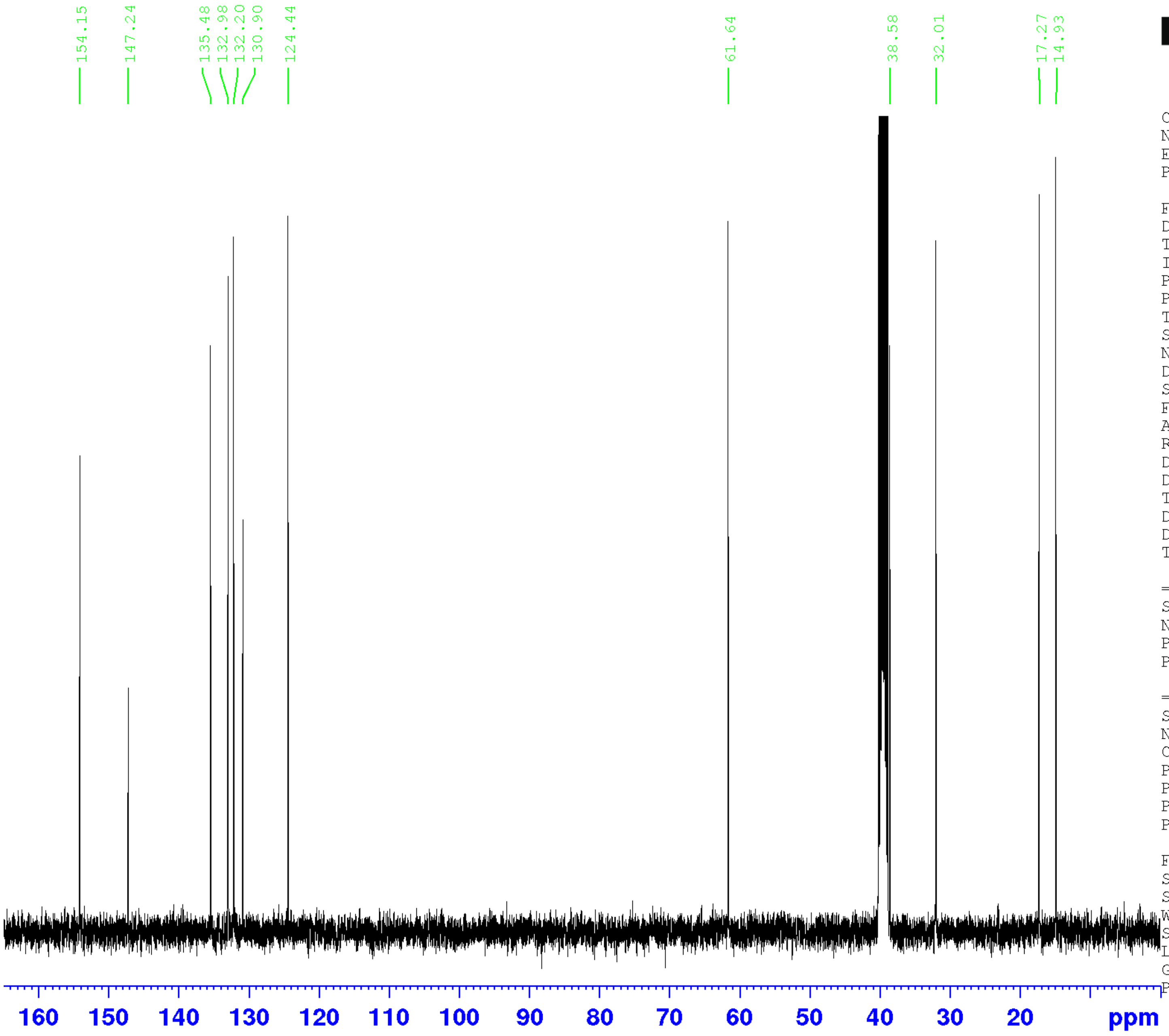


```
Current Data Parameters
NAME       ZL-1-019-FinalConc
EXPNO                      11
PROCNO                      1

F2 - Acquisition Parameters
Date_              20170324
Time                   4.02
INSTRUM               spect
PROBHD     5 mm PABBO BB/
PULPROG             zgpg30
TD                   65536
SOLVENT               DMSO
NS                    1024
DS                       4
SWH              24038.461 Hz
FIDRES            0.366798 Hz
AQ               1.3631488 sec
RG                     512
DW                  20.800 usec
DE                    6.50 usec
TE                   300.0 K
D1              2.00000000 sec
D11             0.03000000 sec
TD0                      1

======== CHANNEL f1 ========
SFO1          100.5649900 MHz
NUC1                  13C
P1                  10.00 usec
PLW1          55.00000000 W

======== CHANNEL f2 ========
SFO2          399.9015996 MHz
NUC2                   1H
CPDPRG[2            waltz16
PCPD2               90.00 usec
PLW2          19.00000000 W
PLW12          0.29951999 W
PLW13          0.24260999 W

F2 - Processing parameters
SI                   32768
SF            100.5549848 MHz
WDW                     EM
SSB        0
LB                    1.00 Hz
GB         0
PC                    1.40
```

3

^{1}H NMR

600 MHz

$CDCl_3$

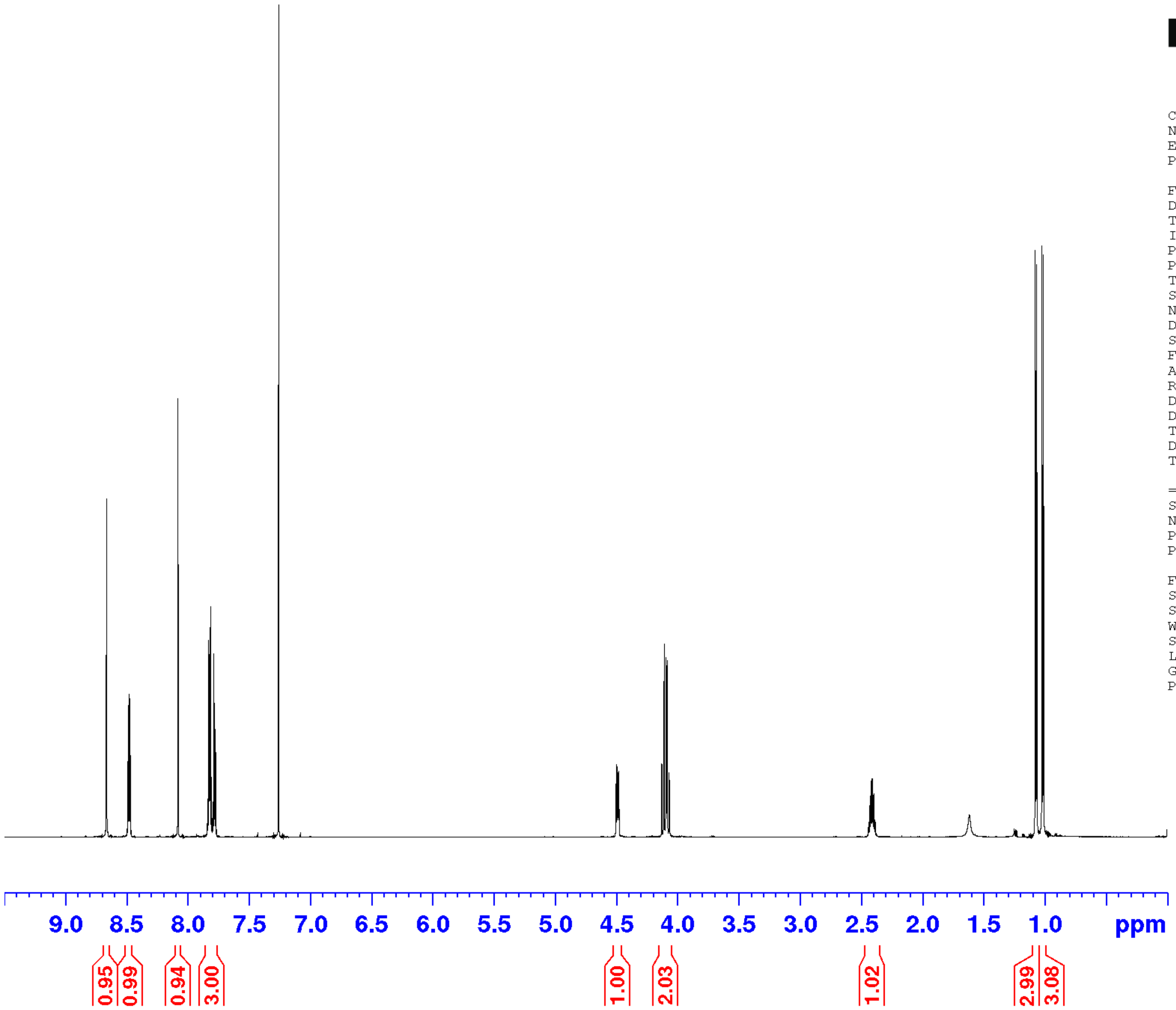


```
Current Data Parameters
NAME      ZL 2.18 FINAL 13C 600 MHz
EXPNO                    10
PROCNO                    1

F2 - Acquisition Parameters
Date_              20170823
Time                  20.32
INSTRUM               spect
PROBHD    5 mm PABBO BB/
PULPROG                zg30
TD                   180286
SOLVENT               CDCl3
NS                       16
DS                        0
SWH              18028.846 Hz
FIDRES            0.100001 Hz
AQ               4.9999318 sec
RG                     97.5
DW                   27.733 usec
DE                     7.60 usec
TE                    298.1 K
D1              0.10000000 sec
TD0                       1

======== CHANNEL f1 ========
SFO1          600.1337060 MHz
NUC1                     1H
P1                    10.00 usec
PLW1            26.60000038 W

F2 - Processing parameters
SI                   262144
SF            600.1300143 MHz
WDW                      EM
SSB        0
LB                     0.10 Hz
GB         0
PC                     1.00
```

3

^{13}C NMR

151 MHz

$CDCl_3$

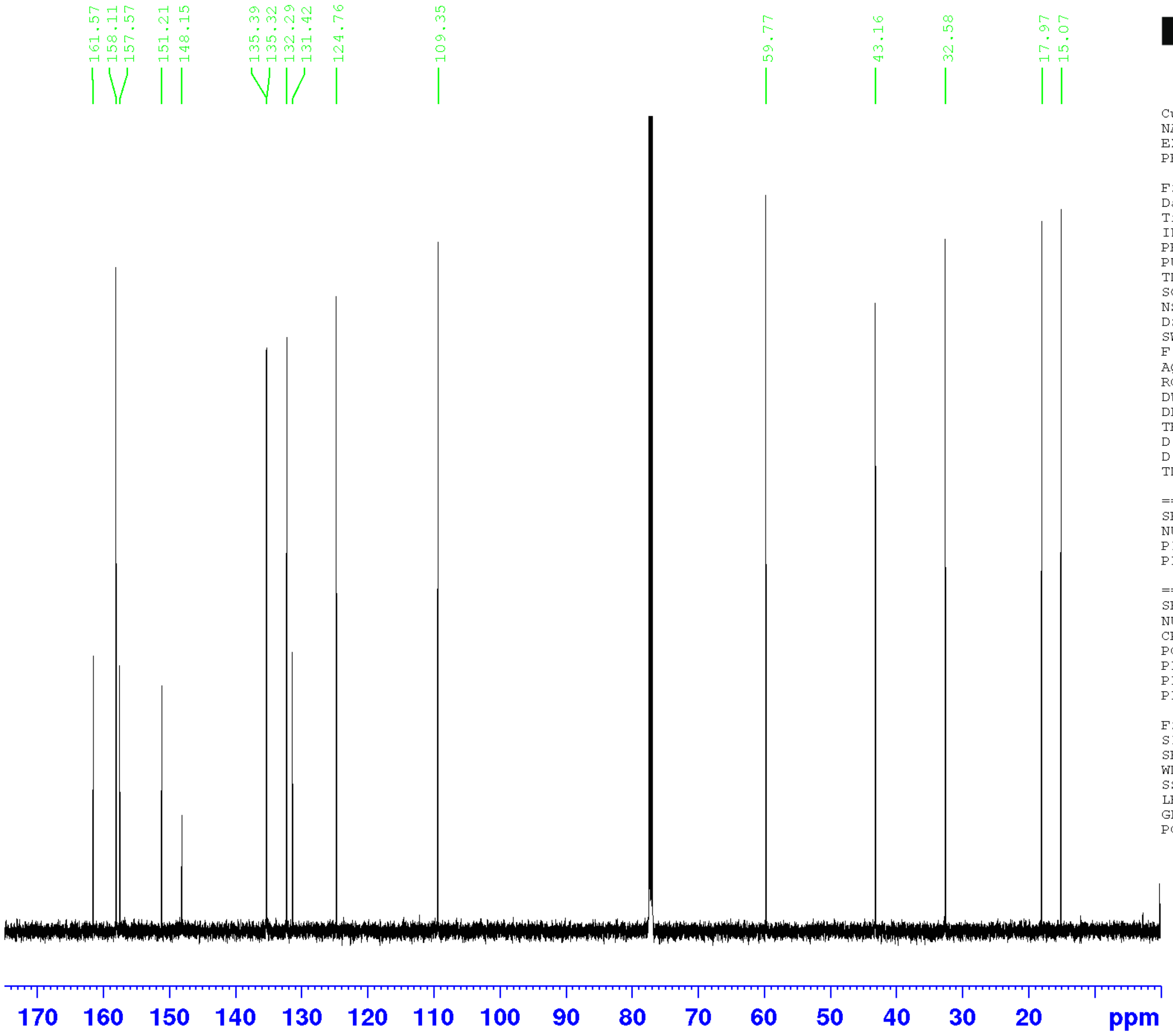


```
Current Data Parameters
NAME       ZL 2.18 FINAL 13C 600 MHz
EXPNO                     11
PROCNO                     1

F2 - Acquisition Parameters
Date_               20170823
Time                   21.18
INSTRUM                spect
PROBHD     5 mm PABBO BB/
PULPROG               zgpg30
TD                    119044
SOLVENT                CDCl3
NS                      1024
DS                         4
SWH               37500.000 Hz
FIDRES             0.315010 Hz
AQ                1.5872533 sec
RG                    186.92
DW                    13.333 usec
DE                      7.73 usec
TE                     298.1 K
D1               1.00000000 sec
D11              0.03000000 sec
TD0                        1

======== CHANNEL f1 ========
SFO1            150.9194058 MHz
NUC1                    13C
P1                     11.80 usec
PLW1            85.00000000 W

======== CHANNEL f2 ========
SFO2            600.1324005 MHz
NUC2                      1H
CPDPRG[2             waltz64
PCPD2                  80.00 usec
PLW2            27.00000000 W
PLW12            0.43891999 W
PLW13            0.28090999 W

F2 - Processing parameters
SI                    131072
SF              150.9027897 MHz
WDW                       EM
SSB      0
LB                      1.00 Hz
GB       0
PC                      1.40
```

S4

^{1}H NMR

400 MHz

$CDCl_3$

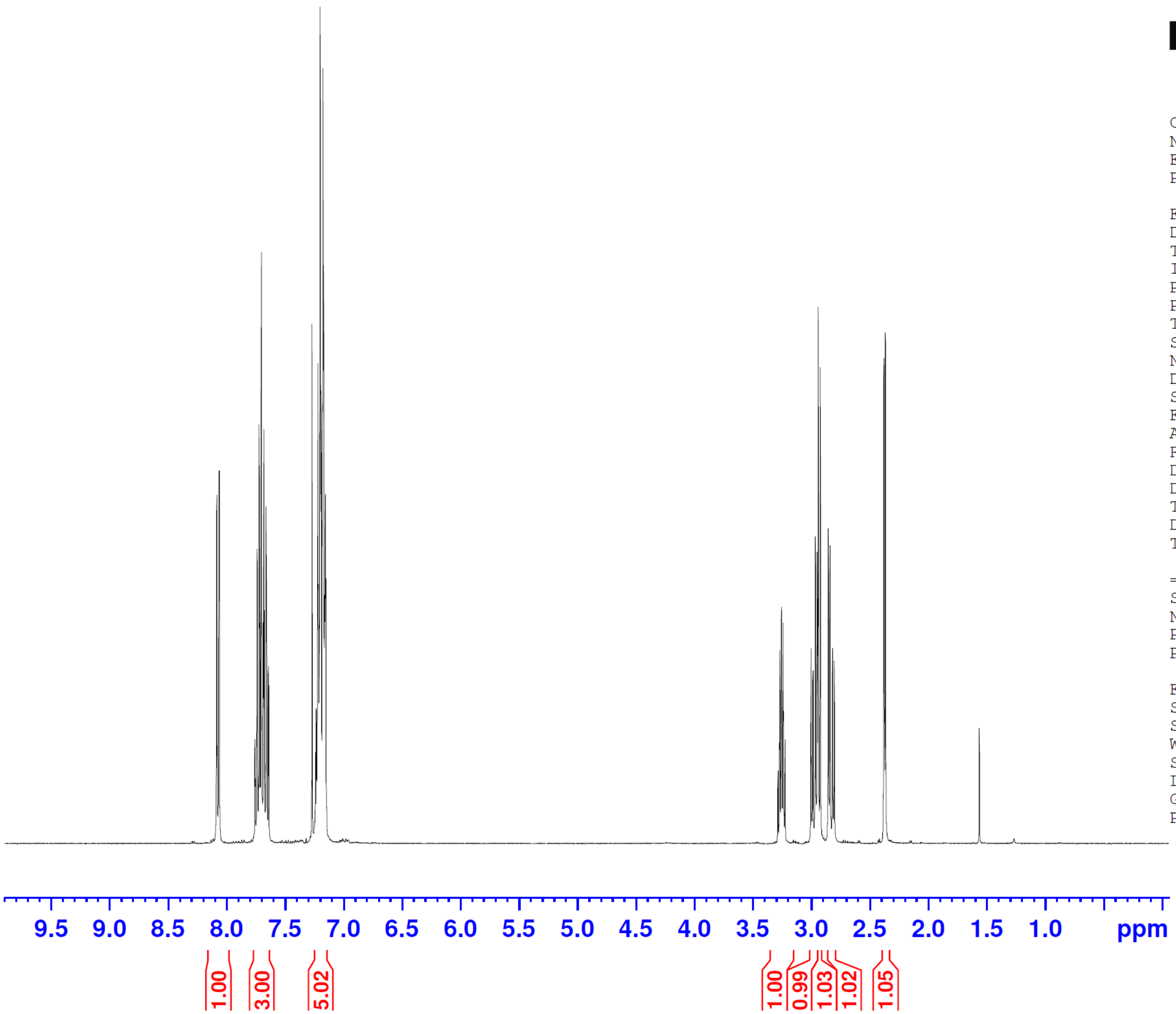


```
Current Data Parameters
NAME      pck 1.82 recryst
EXPNO                   10
PROCNO                   1

F2 - Acquisition Parameters
Date_             20170417
Time                 15.16
INSTRUM          AVIII_400
PROBHD    5 mm PABBO BB-
PULPROG               zg30
TD                   65536
SOLVENT              CDCl3
NS                      16
DS                       2
SWH               8223.685 Hz
FIDRES            0.125483 Hz
AQ               3.9845889 sec
RG                     203
DW                  60.800 usec
DE                   16.82 usec
TE                   300.0 K
D1              1.00000000 sec
TD0                      1

======== CHANNEL f1 ========
SFO1         400.1124708 MHz
NUC1                    1H
P1                   15.00 usec
PLW1           17.29199982 W

F2 - Processing parameters
SI                   32768
SF           400.1100052 MHz
WDW                     EM
SSB       0
LB                    0.30 Hz
GB        0
PC                    1.00
```

S4

^{13}C NMR
(DEPT-Q)

101 MHz

$CDCl_3$

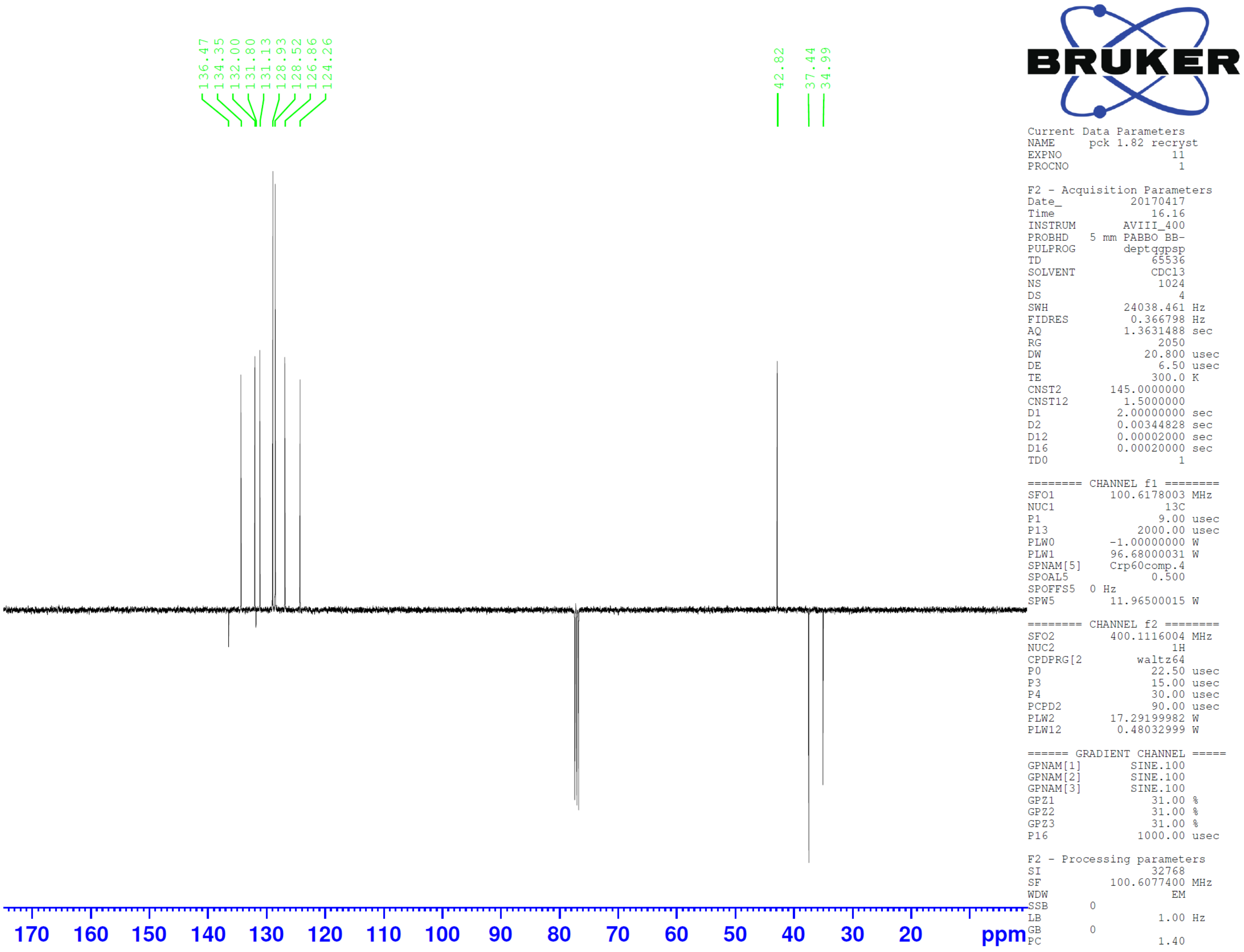

4

^1H NMR

600 MHz

$CDCl_3$

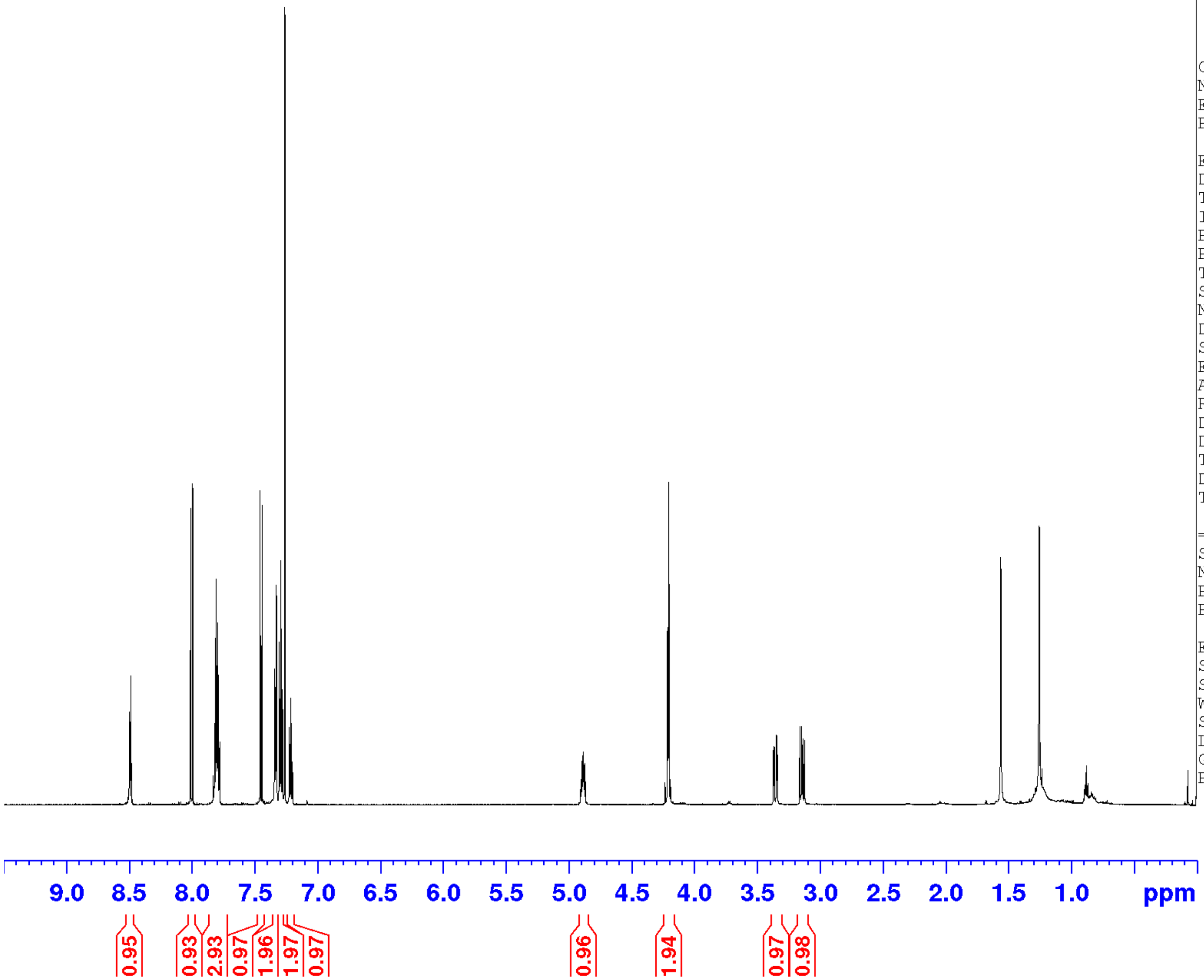


```
Current Data Parameters
NAME          PCK ZL 2.08
EXPNO                  10
PROCNO                  1

F2 - Acquisition Parameters
Date_            20171220
Time                 1.01
INSTRUM             spect
PROBHD   5 mm PABBO BB/
PULPROG              zg30
TD                 180286
SOLVENT             CDCl3
NS                     16
DS                      0
SWH            18028.846 Hz
FIDRES          0.100001 Hz
AQ             4.9999318 sec
RG                   97.5
DW                 27.733 usec
DE                   7.60 usec
TE                  298.2 K
D1             0.10000000 sec
TD0                     1

======== CHANNEL f1 ========
SFO1         600.1337060 MHz
NUC1                   1H
P1                  10.00 usec
PLW1          26.60000038 W

F2 - Processing parameters
SI                 262144
SF           600.1300142 MHz
WDW                    EM
SSB       0
LB                   0.10 Hz
GB        0
PC                   1.00
```

4

^{13}C NMR

151 MHz

$CDCl_3$

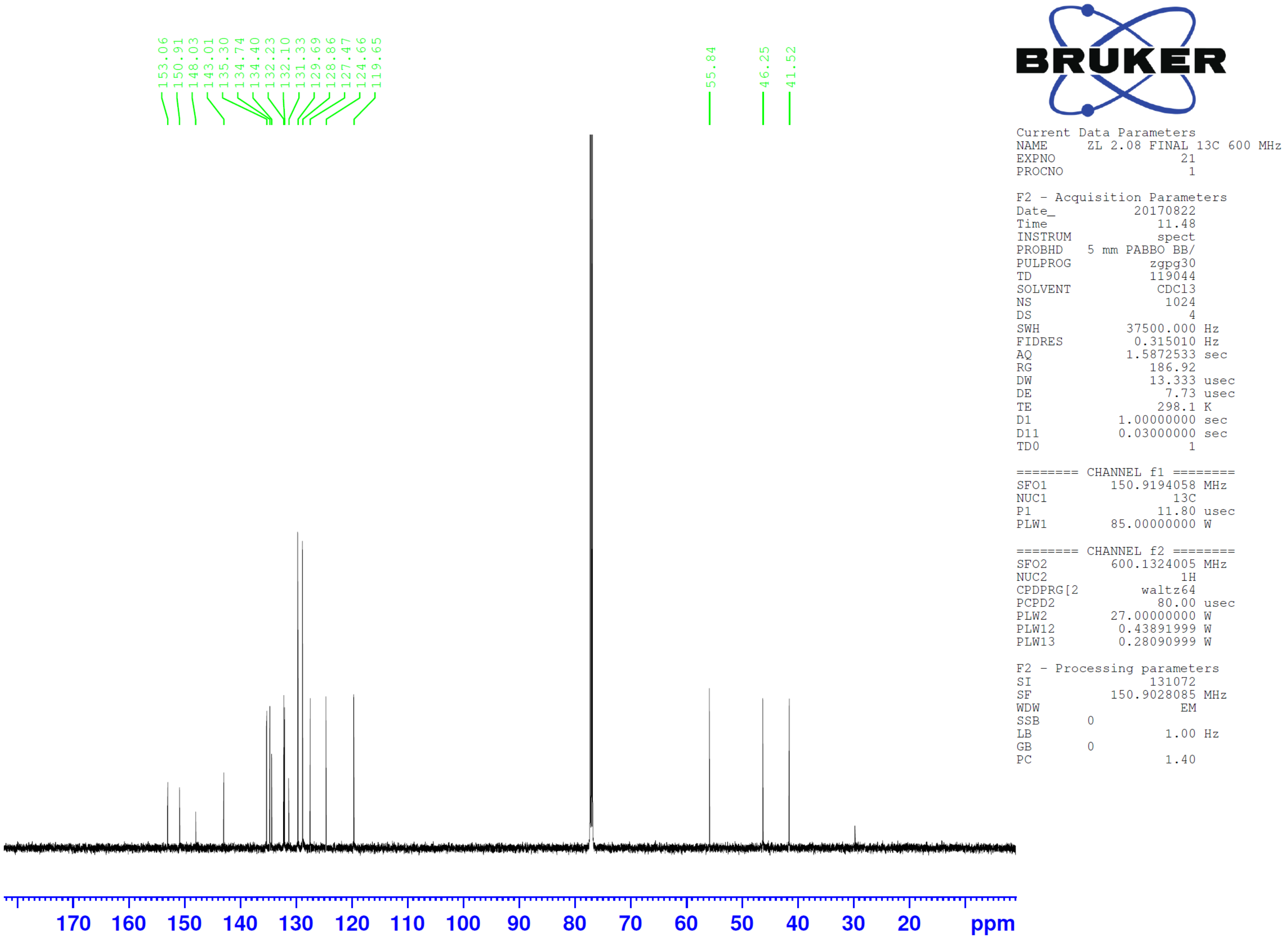

5

^{1}H NMR

400 MHz

$CDCl_3$

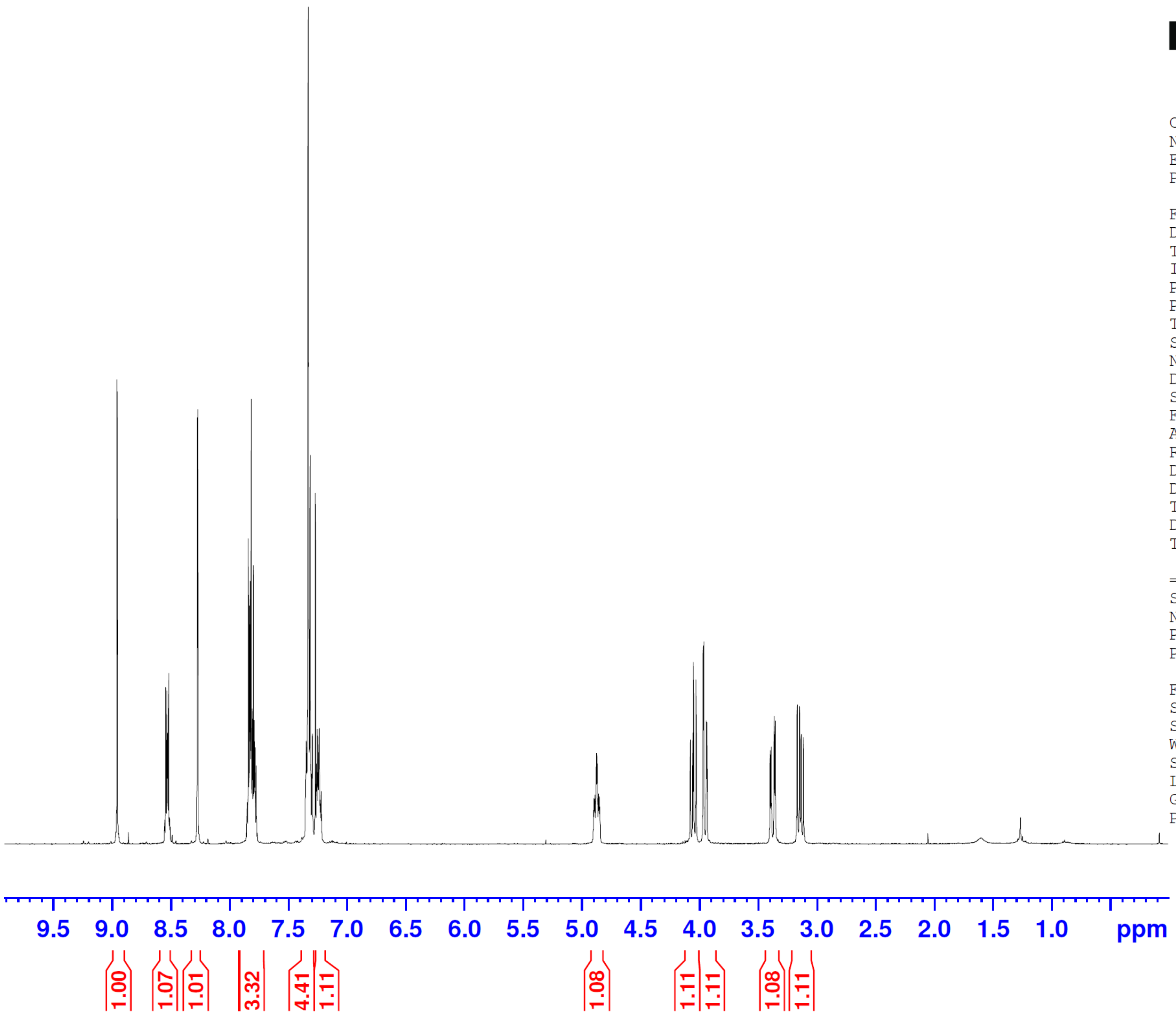


```
Current Data Parameters
NAME       pck 2.01 repeat
EXPNO                   10
PROCNO                   1

F2 - Acquisition Parameters
Date_             20170506
Time                  1.17
INSTRUM          AVIII_400
PROBHD     5 mm PABBO BB-
PULPROG               zg30
TD                   65536
SOLVENT              CDCl3
NS                      16
DS                       2
SWH               8223.685 Hz
FIDRES            0.125483 Hz
AQ               3.9845889 sec
RG                     228
DW                  60.800 usec
DE                   16.82 usec
TE                   300.0 K
D1              1.00000000 sec
TD0                      1

======== CHANNEL f1 ========
SFO1           400.1124708 MHz
NUC1                    1H
P1                   15.00 usec
PLW1           17.29199982 W

F2 - Processing parameters
SI                   32768
SF             400.1100058 MHz
WDW                     EM
SSB       0
LB                    0.30 Hz
GB        0
PC                    1.00
```

5

^{13}C NMR
(DEPT-Q)

101 MHz

$CDCl_3$

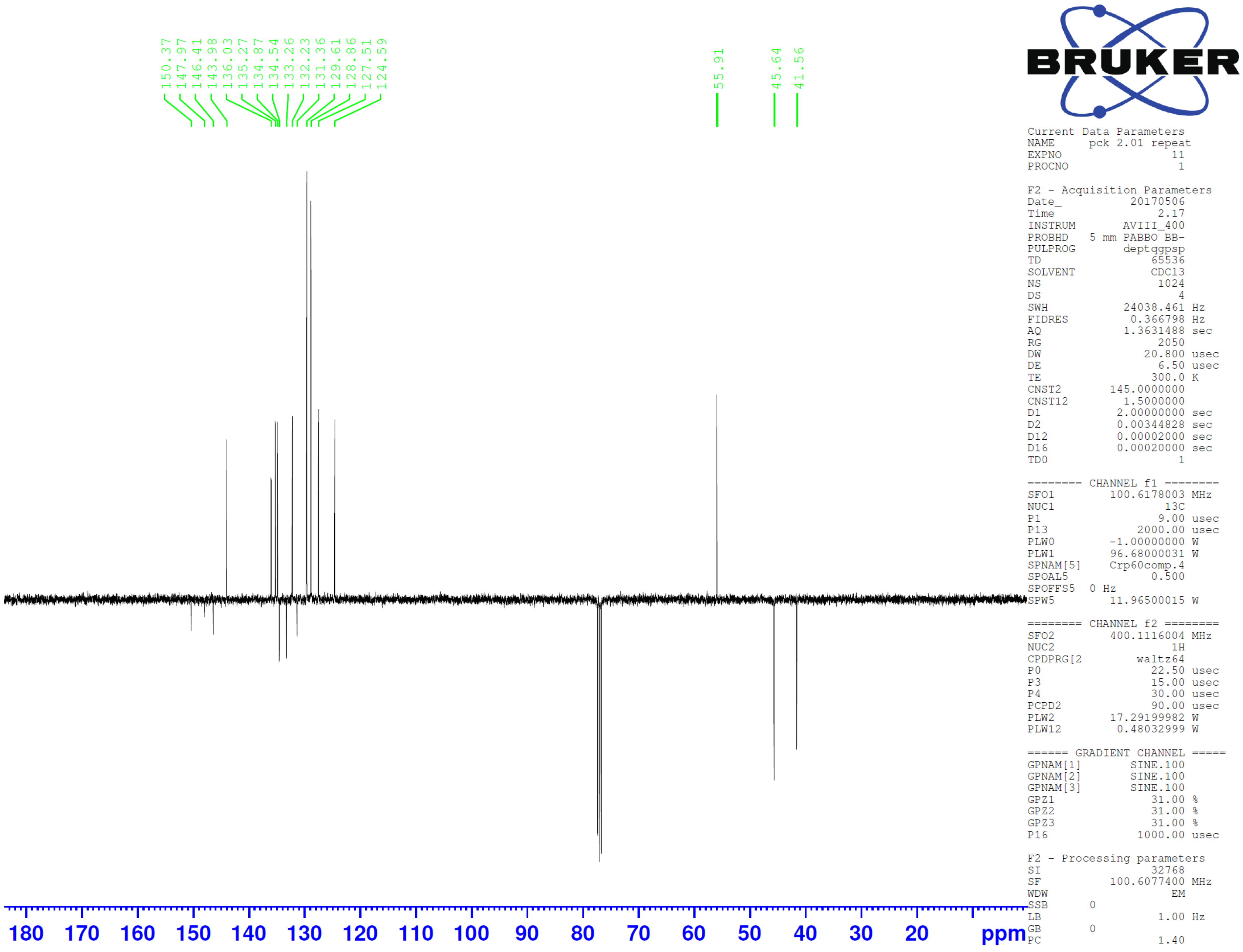

6

^1H NMR

400 MHz

$CDCl_3$

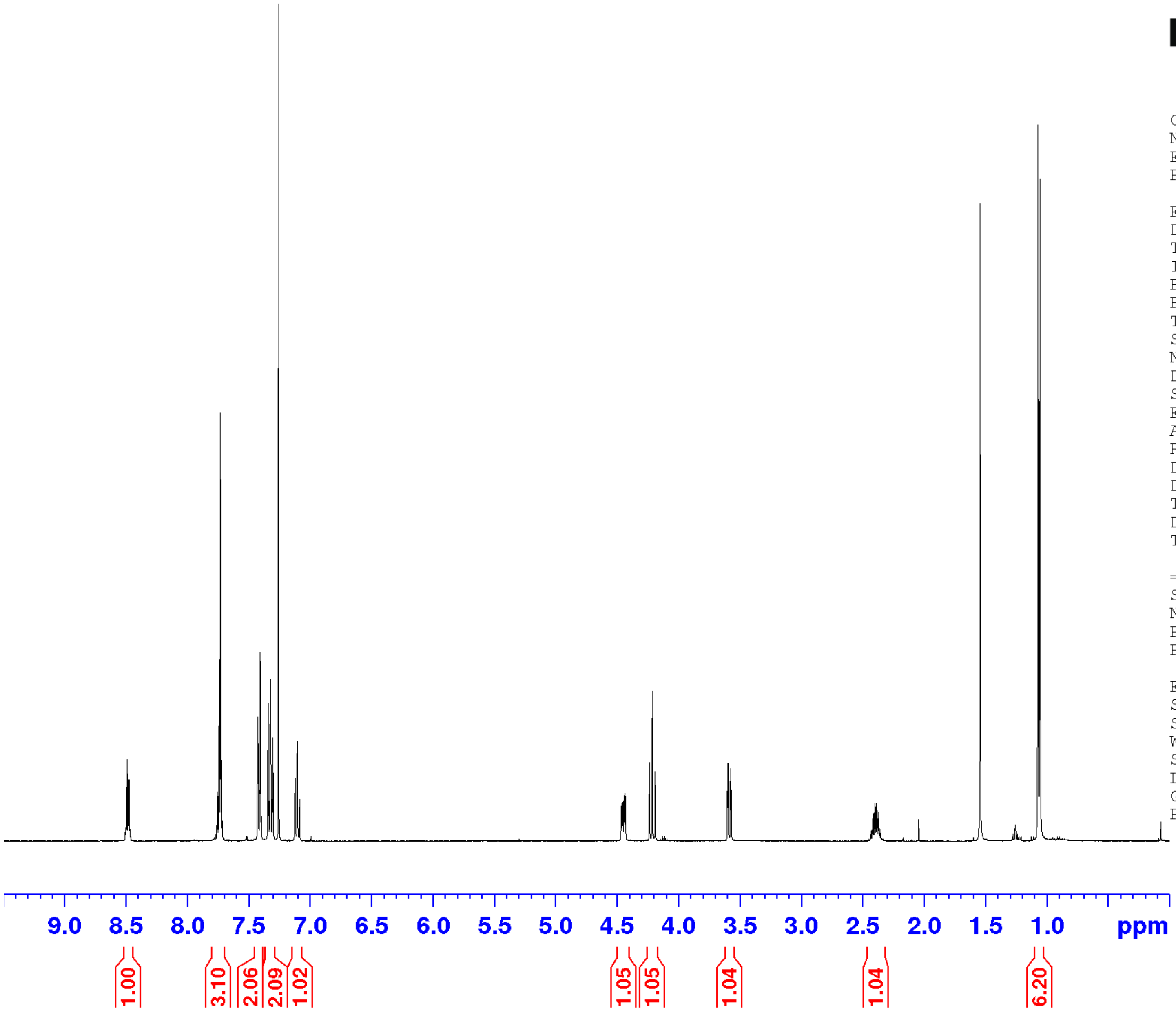


```
Current Data Parameters
NAME       ZL-1-020-Final
EXPNO                  11
PROCNO                  1

F2 - Acquisition Parameters
Date_            20170323
Time                18.50
INSTRUM         AVIII_400
PROBHD     5 mm PABBO BB-
PULPROG              zg30
TD                  65536
SOLVENT             CDCl3
NS                    100
DS                      2
SWH           8223.685 Hz
FIDRES        0.125483 Hz
AQ          3.9845889 sec
RG                    406
DW            60.800 usec
DE             16.82 usec
TE                300.0 K
D1         1.00000000 sec
TD0                     1

======== CHANNEL f1 ========
SFO1       400.1124708 MHz
NUC1                   1H
P1             15.00 usec
PLW1       17.29199982 W

F2 - Processing parameters
SI                  32768
SF         400.1100095 MHz
WDW                    EM
SSB      0
LB                0.30 Hz
GB       0
PC                   1.00
```

6

^{13}C NMR

101 MHz

$CDCl_3$

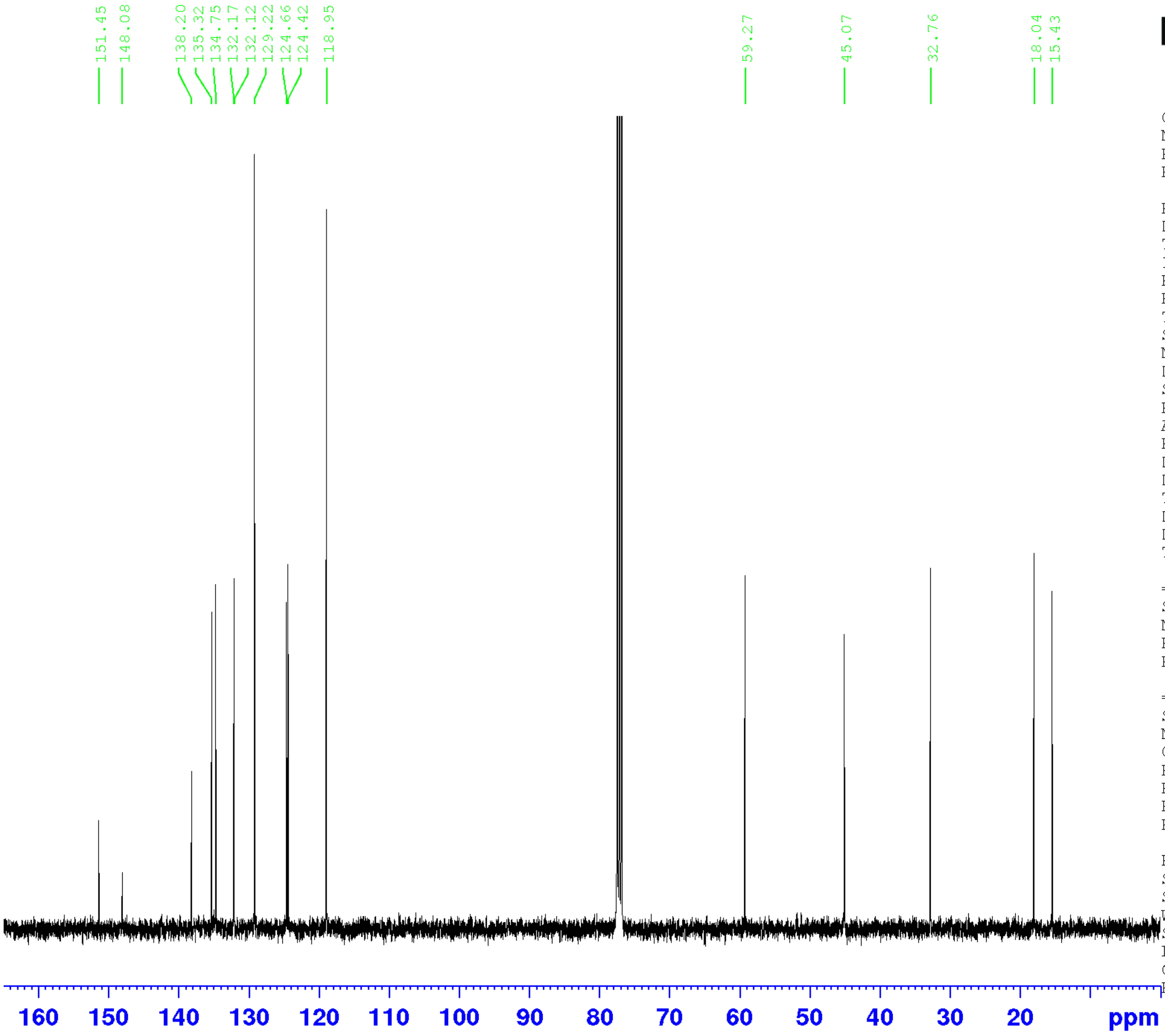


```
Current Data Parameters
NAME       ZL-1-020-FinalConc
EXPNO                      21
PROCNO                      1

F2 - Acquisition Parameters
Date_              20170324
Time                   5.10
INSTRUM               spect
PROBHD   5 mm PABBO BB/
PULPROG             zgpg30
TD                   65536
SOLVENT              CDCl3
NS                    1024
DS                       4
SWH              24038.461 Hz
FIDRES            0.366798 Hz
AQ               1.3631488 sec
RG                     228
DW                  20.800 usec
DE                    6.50 usec
TE                   300.0 K
D1              2.00000000 sec
D11             0.03000000 sec
TD0                      1

======== CHANNEL f1 ========
SFO1           100.5649900 MHz
NUC1                   13C
P1                   10.00 usec
PLW1           55.00000000 W

======== CHANNEL f2 ========
SFO2           399.9015996 MHz
NUC2                    1H
CPDPRG[2             waltz16
PCPD2                90.00 usec
PLW2           19.00000000 W
PLW12           0.29951999 W
PLW13           0.24260999 W

F2 - Processing parameters
SI                   32768
SF             100.5549219 MHz
WDW                     EM
SSB      0
LB                    1.00 Hz
GB       0
PC                    1.40
```

7

^{1}H NMR

400 MHz

$CDCl_3$

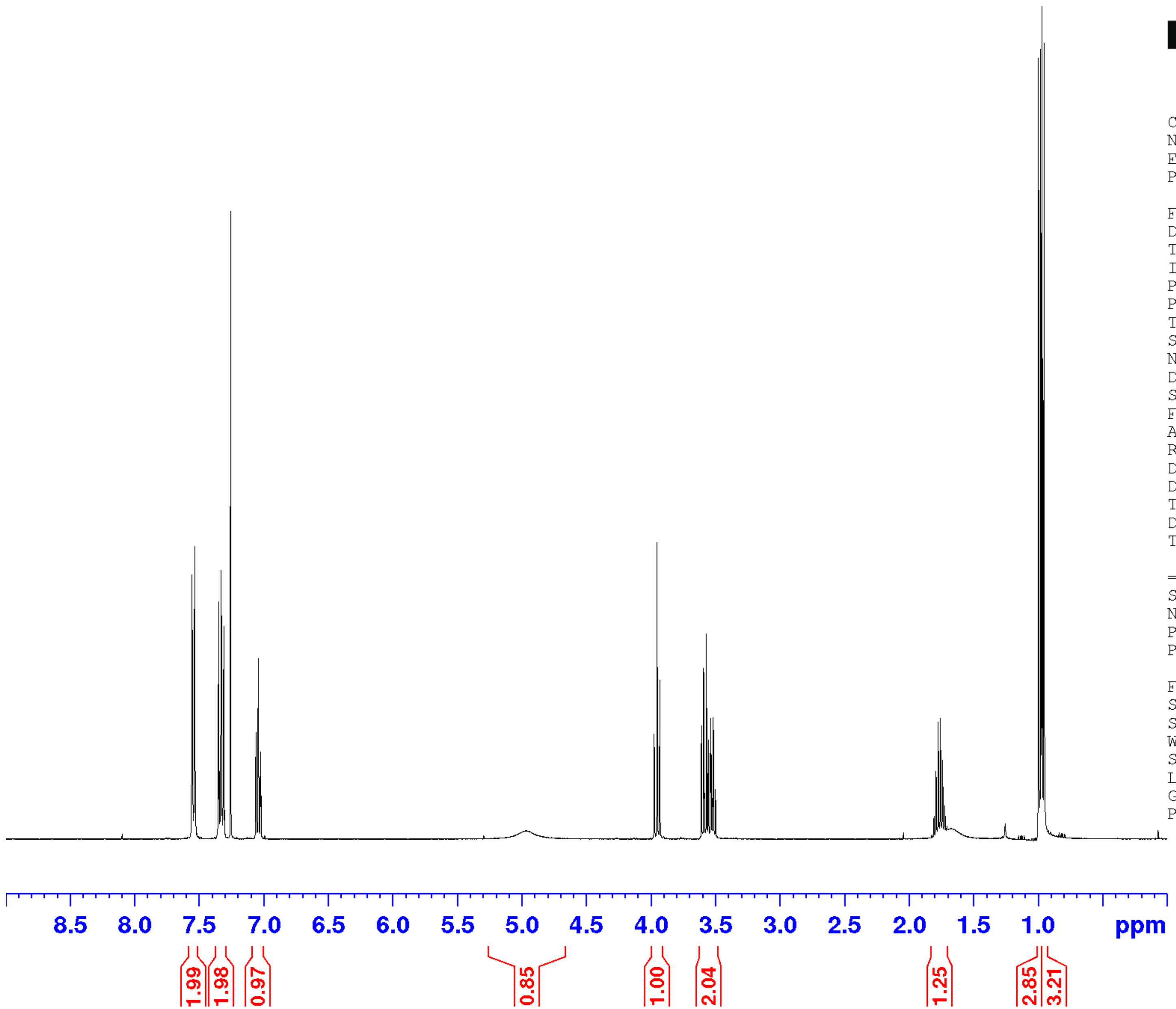


```
Current Data Parameters
NAME        ZL-1-028-Final
EXPNO                   10
PROCNO                   1

F2 - Acquisition Parameters
Date_             20170323
Time                 19.51
INSTRUM          AVIII_400
PROBHD      5 mm PABBO BB-
PULPROG               zg30
TD                   65536
SOLVENT              CDCl3
NS                      16
DS                       2
SWH            8223.685 Hz
FIDRES         0.125483 Hz
AQ           3.9845889 sec
RG                     287
DW              60.800 usec
DE               16.82 usec
TE                 300.0 K
D1          1.00000000 sec
TD0                      1

======== CHANNEL f1 ========
SFO1        400.1124708 MHz
NUC1                    1H
P1               15.00 usec
PLW1        17.29199982 W

F2 - Processing parameters
SI                   32768
SF          400.1100095 MHz
WDW                     EM
SSB      0
LB                 0.30 Hz
GB       0
PC                    1.00
```

7

^{13}C NMR

101 MHz

$CDCl_3$

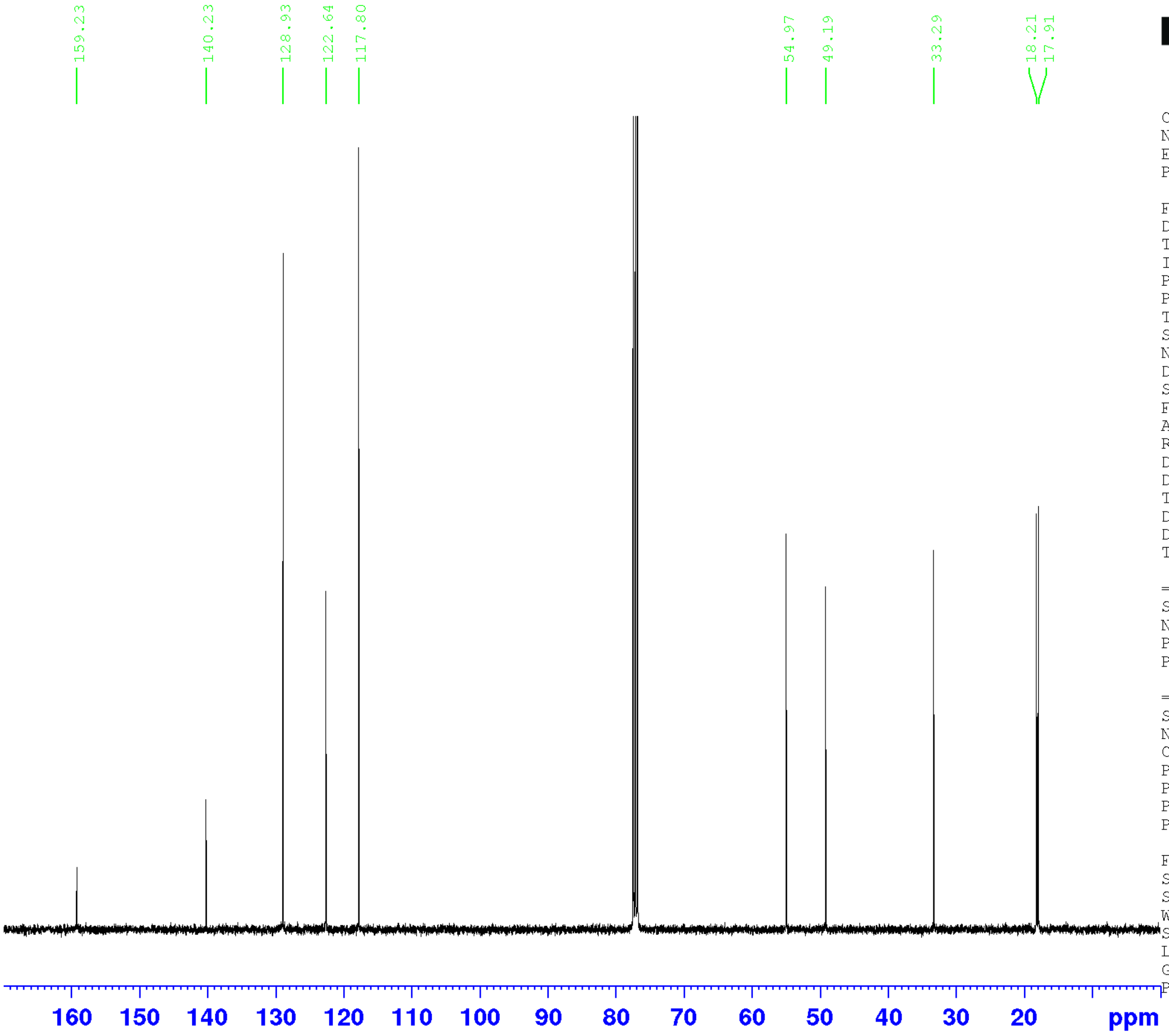

Current Data Parameters
NAME ZL-1-028-FinalConc
EXPNO 41
PROCNO 1

F2 - Acquisition Parameters
Date_ 20170324
Time 8.44
INSTRUM spect
PROBHD 5 mm PABBO BB/
PULPROG zgpg30
TD 65536
SOLVENT CDCl3
NS 1024
DS 4
SWH 24038.461 Hz
FIDRES 0.366798 Hz
AQ 1.3631488 sec
RG 406
DW 20.800 usec
DE 6.50 usec
TE 300.0 K
D1 2.00000000 sec
D11 0.03000000 sec
TD0 1

======== CHANNEL f1 ========
SFO1 100.5649900 MHz
NUC1 13C
P1 10.00 usec
PLW1 55.00000000 W

======== CHANNEL f2 ========
SFO2 399.9015996 MHz
NUC2 1H
CPDPRG[2 waltz16
PCPD2 90.00 usec
PLW2 19.00000000 W
PLW12 0.29951999 W
PLW13 0.24260999 W

F2 - Processing parameters
SI 32768
SF 100.5549235 MHz
WDW EM
SSB 0
LB 1.00 Hz
GB 0
PC 1.40

10

^{1}H NMR

600 MHz

$CDCl_3$

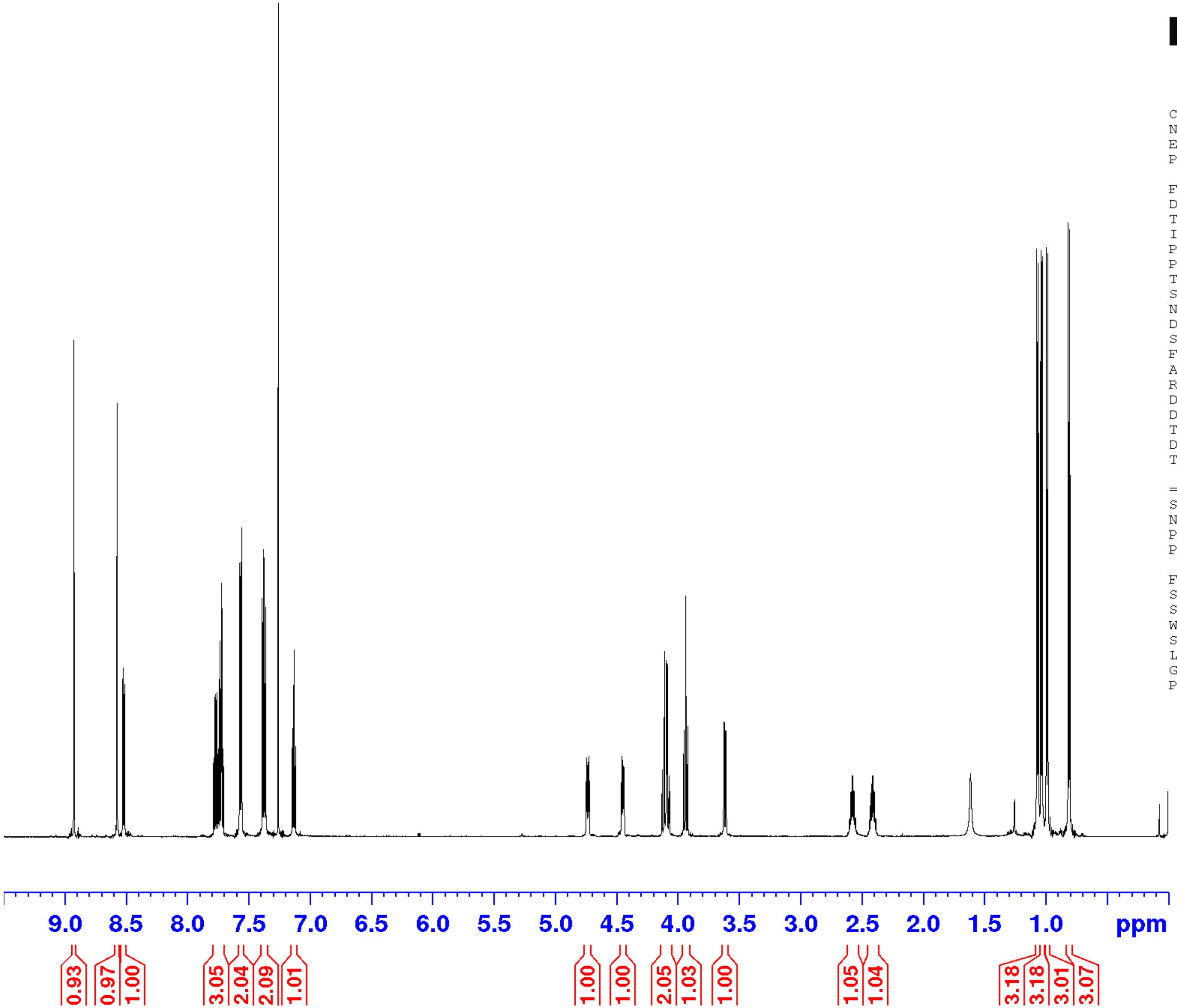


```
Current Data Parameters
NAME       ZL 2.19 FINAL 13C 600 MHz
EXPNO                     20
PROCNO                     1

F2 - Acquisition Parameters
Date_               20170823
Time                   22.40
INSTRUM                spect
PROBHD     5 mm PABBO BB/
PULPROG                 zg30
TD                    180286
SOLVENT                CDCl3
NS                        16
DS                         0
SWH               18028.846 Hz
FIDRES             0.100001 Hz
AQ                4.9999318 sec
RG                      97.5
DW                    27.733 usec
DE                      7.60 usec
TE                     298.1 K
D1                0.10000000 sec
TD0                        1

======== CHANNEL f1 ========
SFO1            600.1337060 MHz
NUC1                     1H
P1                     10.00 usec
PLW1             26.60000038 W

F2 - Processing parameters
SI                    262144
SF               600.1300146 MHz
WDW                       EM
SSB         0
LB                      0.10 Hz
GB          0
PC                      1.00
```

10

^{13}C NMR

151 MHz

$CDCl_3$

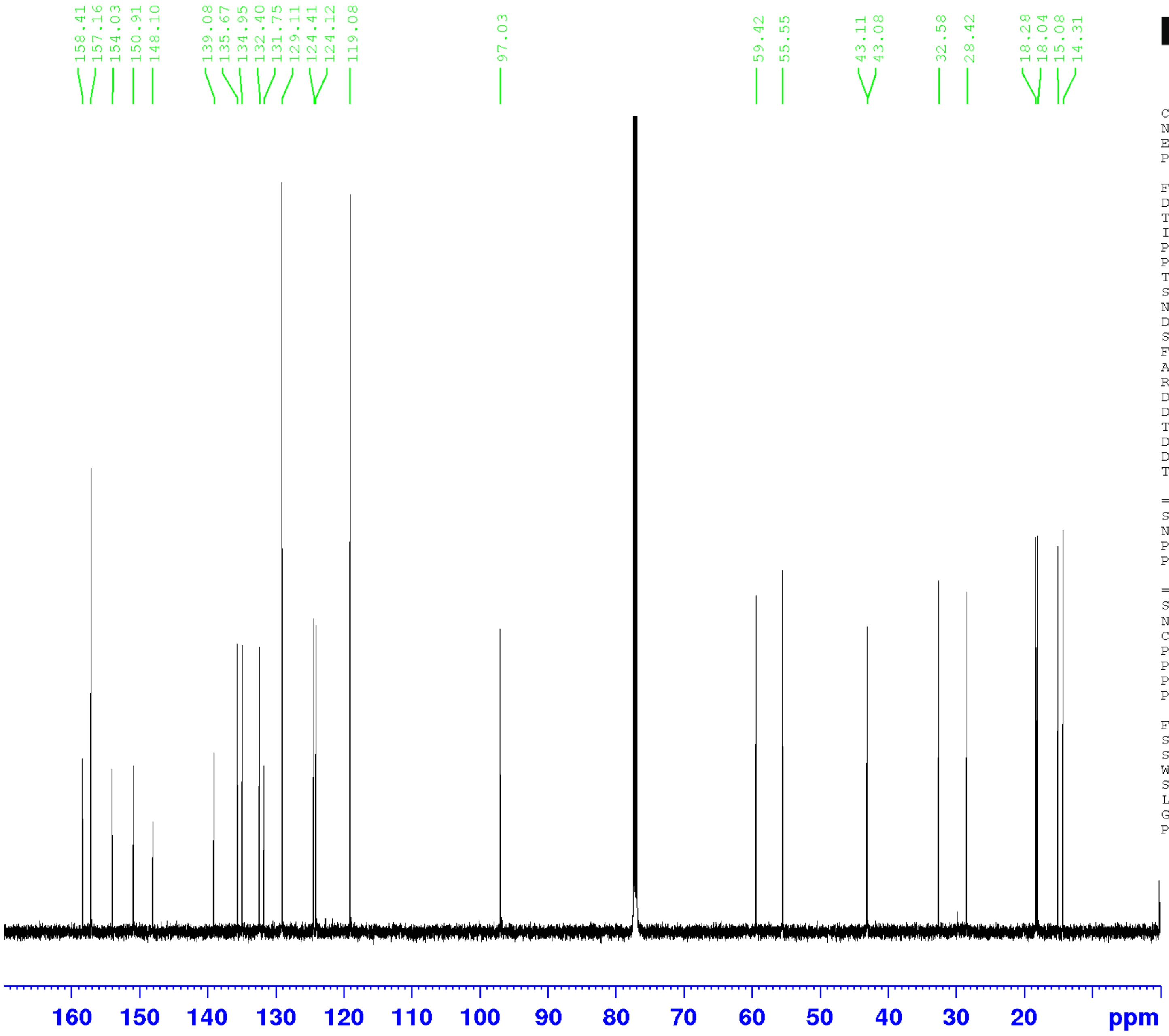

BRUKER

```
Current Data Parameters
NAME       ZL 2.19 FINAL 13C 600 MHz
EXPNO                     21
PROCNO                     1

F2 - Acquisition Parameters
Date_               20170824
Time                    0.12
INSTRUM                spect
PROBHD       5 mm PABBO BB/
PULPROG              zgpg30
TD                    119044
SOLVENT                CDCl3
NS                      2048
DS                         4
SWH               37500.000 Hz
FIDRES             0.315010 Hz
AQ                1.5872533 sec
RG                    186.92
DW                    13.333 usec
DE                      7.73 usec
TE                     298.1 K
D1                1.00000000 sec
D11               0.03000000 sec
TD0                        1

======== CHANNEL f1 ========
SFO1            150.9194058 MHz
NUC1                     13C
P1                     11.80 usec
PLW1             85.00000000 W

======== CHANNEL f2 ========
SFO2            600.1324005 MHz
NUC2                      1H
CPDPRG[2             waltz64
PCPD2                  80.00 usec
PLW2             27.00000000 W
PLW12             0.43891999 W
PLW13             0.28090999 W

F2 - Processing parameters
SI                    131072
SF              150.9027898 MHz
WDW                       EM
SSB       0
LB                      1.00 Hz
GB        0
PC                      1.40
```

10

^1H NMR

151 MHz

d_6-DMSO

298 K

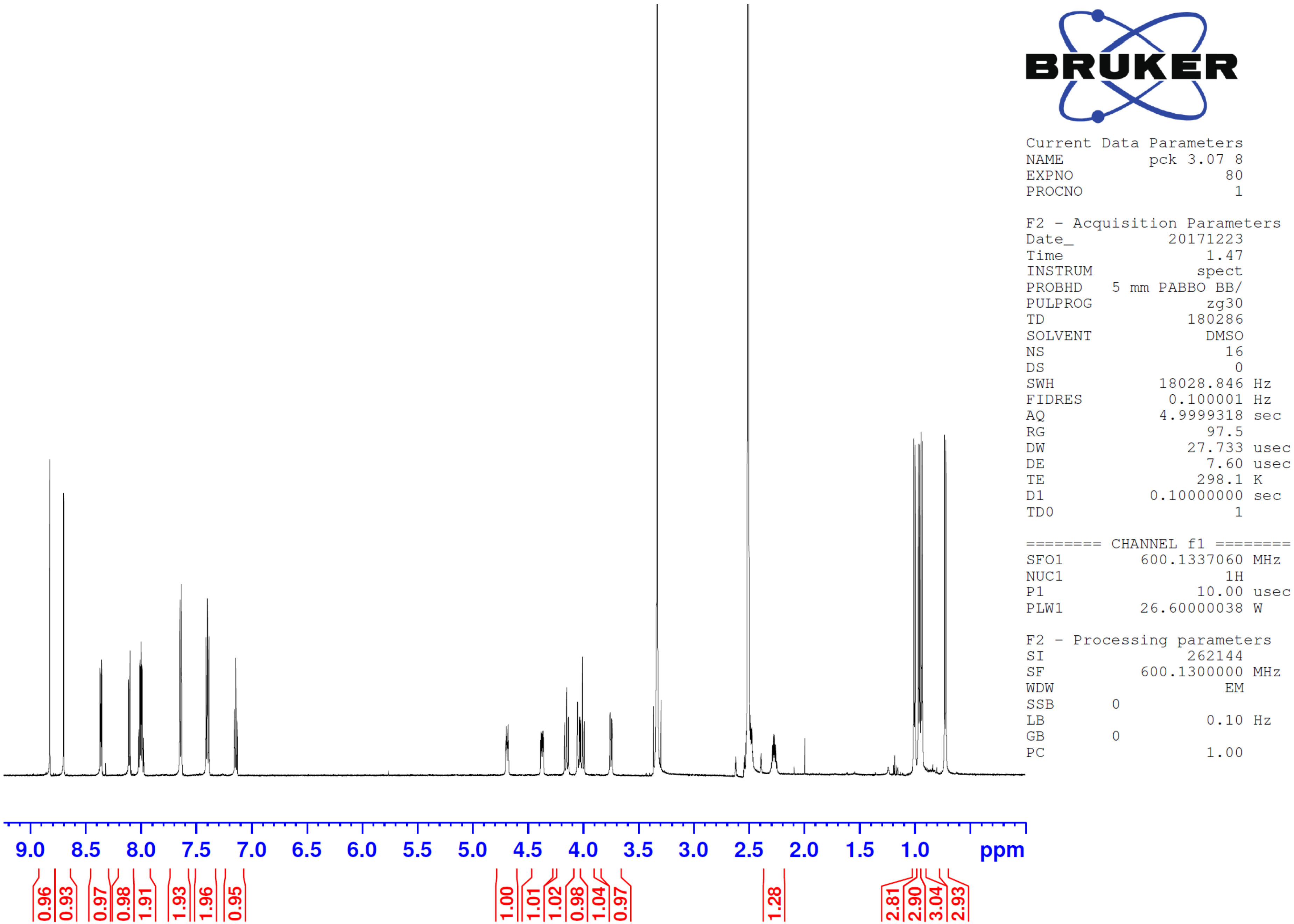

10

^{1}H NMR

151 MHz

d_6-DMSO

355 K

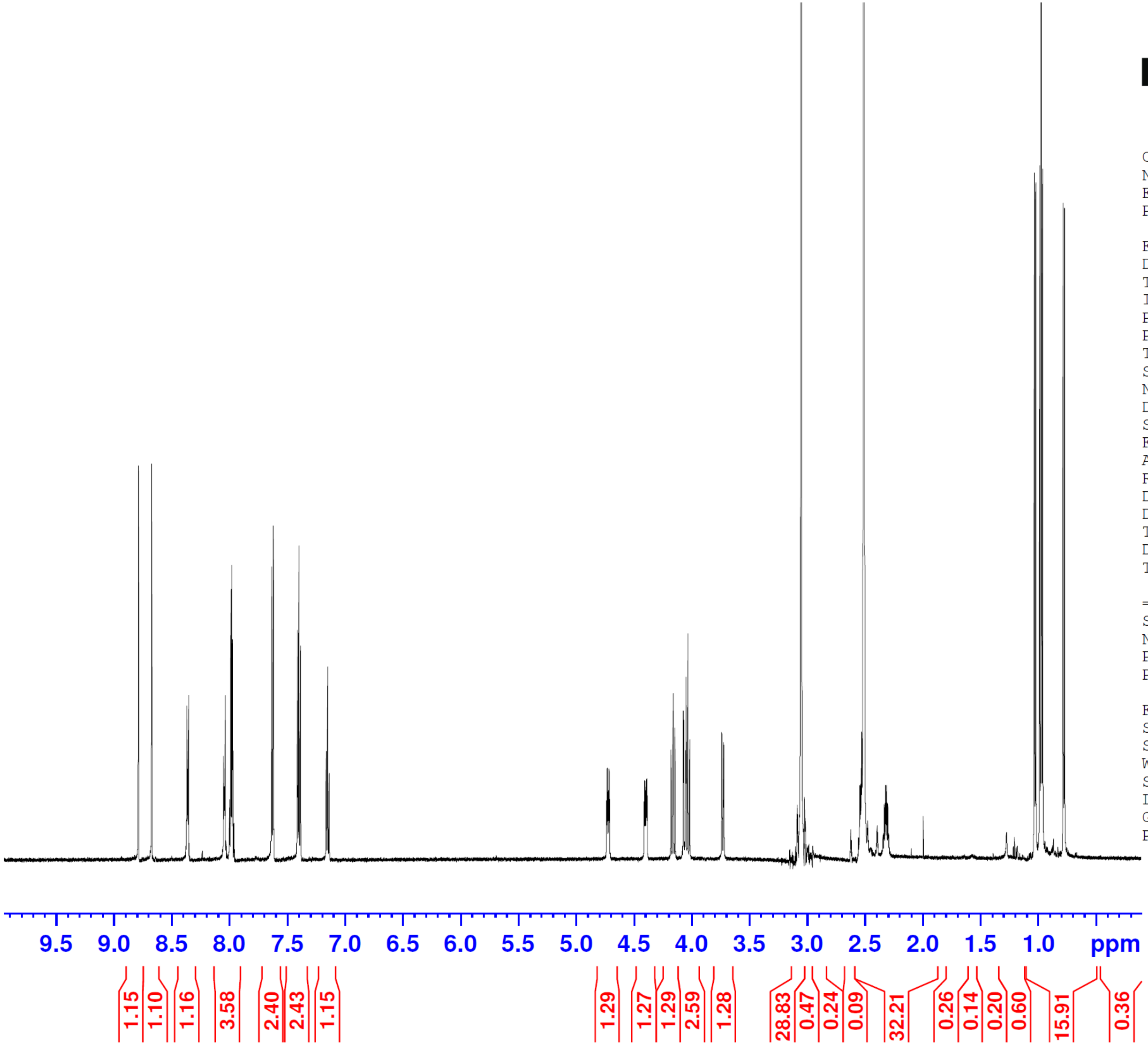


```
Current Data Parameters
NAME      pck 3.07 8 VT 80C
EXPNO                    10
PROCNO                    1

F2 - Acquisition Parameters
Date_              20171227
Time                  14.39
INSTRUM               spect
PROBHD    5 mm PABBO BB/
PULPROG                zg30
TD                   180286
SOLVENT                DMSO
NS                       16
DS                        0
SWH              18028.846 Hz
FIDRES            0.100001 Hz
AQ               4.9999318 sec
RG                     97.5
DW                   27.733 usec
DE                     7.60 usec
TE                    354.9 K
D1               0.10000000 sec
TD0                       1

======== CHANNEL f1 ========
SFO1          600.1337060 MHz
NUC1                     1H
P1                    10.00 usec
PLW1            26.60000038 W

F2 - Processing parameters
SI                   262144
SF            600.1300000 MHz
WDW                      EM
SSB       0
LB                     0.10 Hz
GB        0
PC                     1.00
```

11

^{1}H NMR

600 MHz

$CDCl_3$

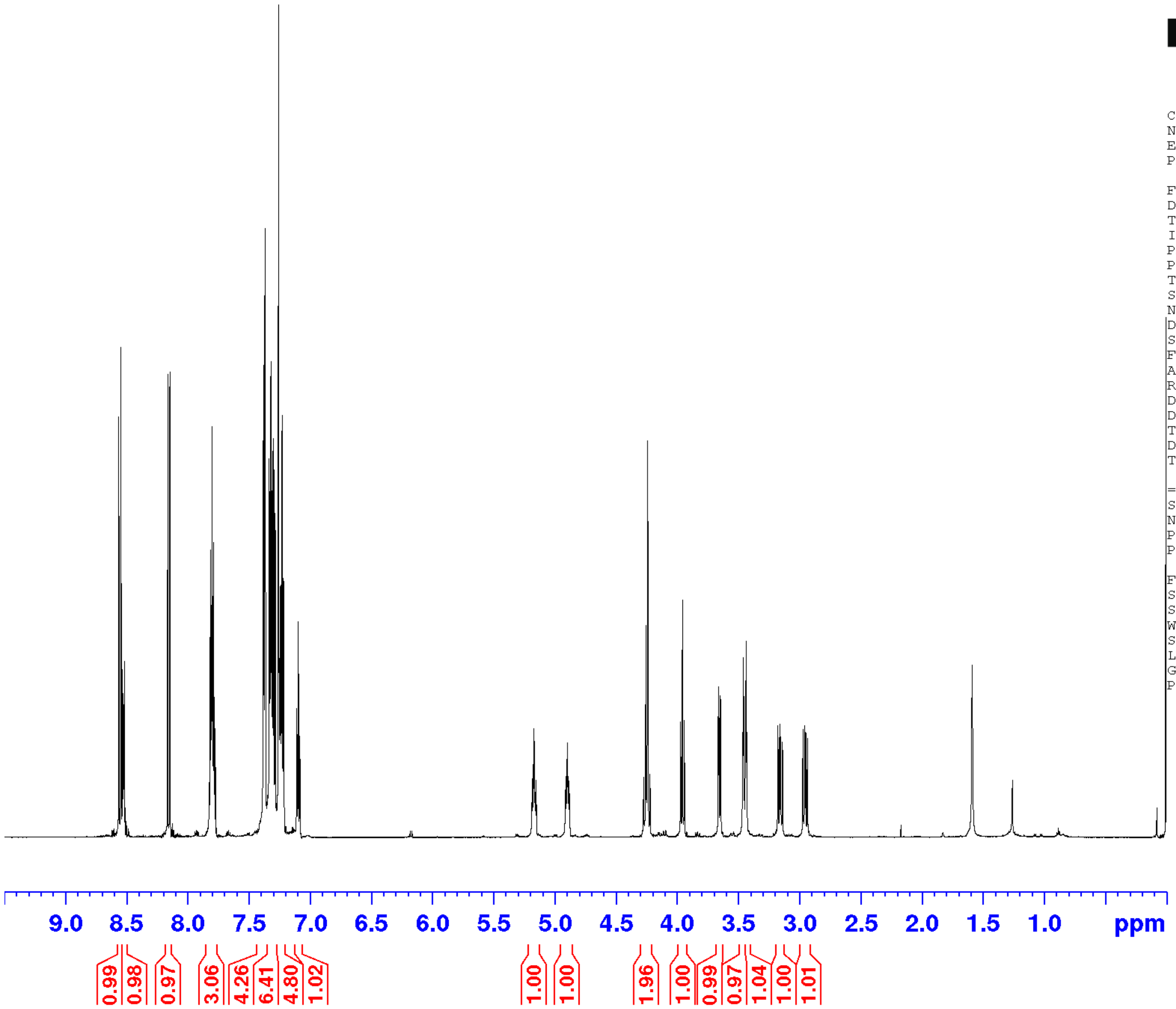


```
Current Data Parameters
NAME      ZL 2.10 FINAL 13C 600 MHz
EXPNO                    10
PROCNO                    1

F2 - Acquisition Parameters
Date_              20170822
Time                  13.06
INSTRUM               spect
PROBHD      5 mm PABBO BB/
PULPROG                zg30
TD                   180286
SOLVENT               CDCl3
NS                       16
DS                        0
SWH              18028.846 Hz
FIDRES            0.100001 Hz
AQ               4.9999318 sec
RG                     97.5
DW                   27.733 usec
DE                     7.60 usec
TE                    298.1 K
D1               0.10000000 sec
TD0                       1

======== CHANNEL f1 ========
SFO1           600.1337060 MHz
NUC1                     1H
P1                    10.00 usec
PLW1            26.60000038 W

F2 - Processing parameters
SI                   262144
SF             600.1300143 MHz
WDW                      EM
SSB         0
LB                     0.10 Hz
GB          0
PC                     1.00
```

11

^{13}C NMR

151 MHz

$CDCl_3$

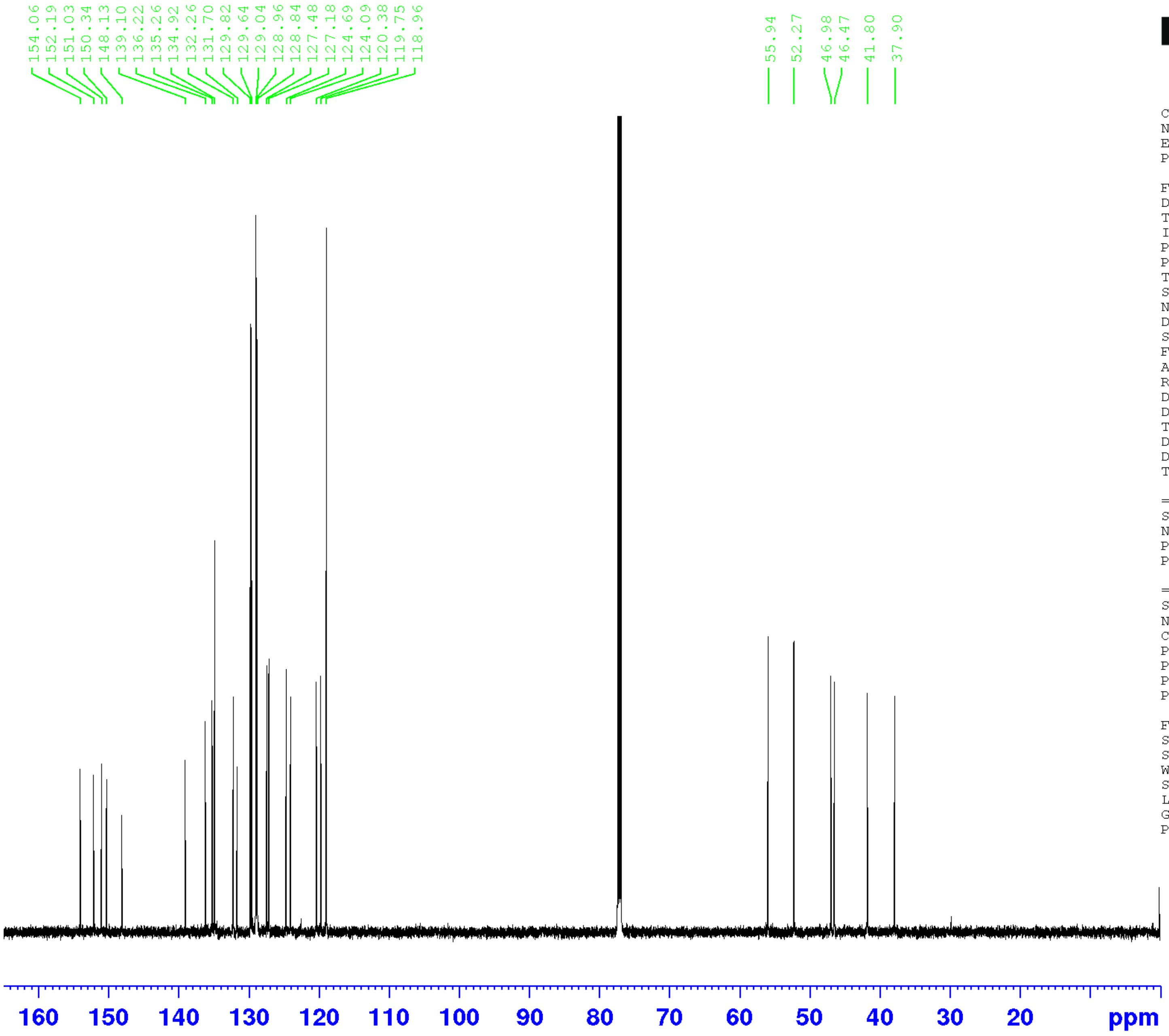


```
Current Data Parameters
NAME      ZL 2.10 FINAL 13C 600 MHz
EXPNO                    11
PROCNO                    1

F2 - Acquisition Parameters
Date_              20170822
Time                  14.37
INSTRUM               spect
PROBHD       5 mm PABBO BB/
PULPROG             zgpg30
TD                  119044
SOLVENT              CDCl3
NS                    2048
DS                       4
SWH             37500.000 Hz
FIDRES           0.315010 Hz
AQ              1.5872533 sec
RG                 186.92
DW                 13.333 usec
DE                   7.73 usec
TE                  298.1 K
D1             1.00000000 sec
D11            0.03000000 sec
TD0                      1

======== CHANNEL f1 ========
SFO1          150.9194058 MHz
NUC1                  13C
P1                  11.80 usec
PLW1          85.00000000 W

======== CHANNEL f2 ========
SFO2          600.1324005 MHz
NUC2                   1H
CPDPRG[2           waltz64
PCPD2               80.00 usec
PLW2          27.00000000 W
PLW12          0.43891999 W
PLW13          0.28090999 W

F2 - Processing parameters
SI                  131072
SF            150.9027919 MHz
WDW                     EM
SSB      0
LB                    1.00 Hz
GB       0
PC                    1.40
```

12

^{1}H NMR

600 MHz

$CDCl_3$

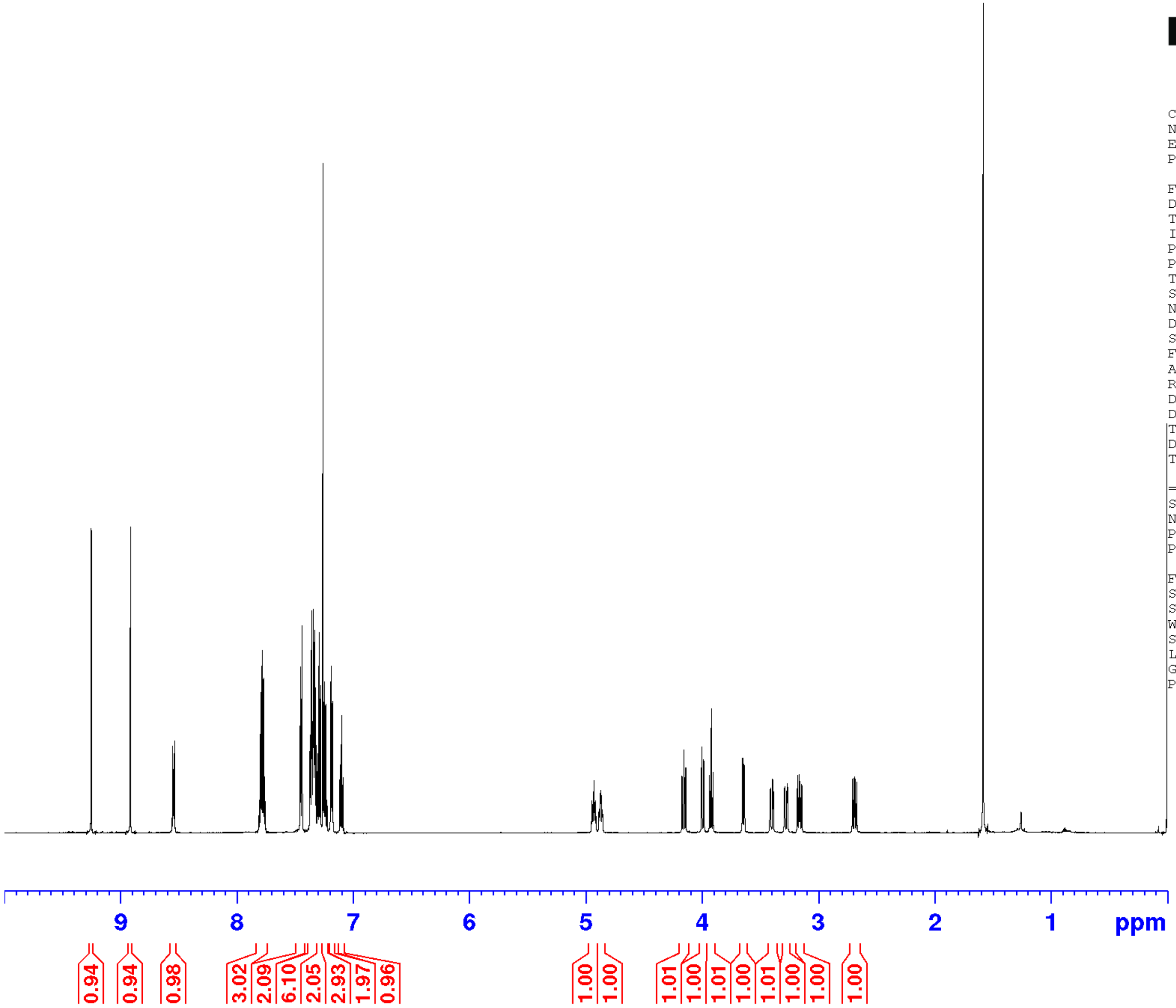

BRUKER

Current Data Parameters
NAME ZL 2.32 FINAL 13C 600 MHz
EXPNO 10
PROCNO 1

F2 - Acquisition Parameters
Date_ 20170825
Time 5.39
INSTRUM spect
PROBHD 5 mm PABBO BB/
PULPROG zg30
TD 180286
SOLVENT CDCl3
NS 16
DS 0
SWH 18028.846 Hz
FIDRES 0.100001 Hz
AQ 4.9999318 sec
RG 97.5
DW 27.733 usec
DE 7.60 usec
TE 298.1 K
D1 0.10000000 sec
TD0 1

======== CHANNEL f1 ========
SFO1 600.1337060 MHz
NUC1 1H
P1 10.00 usec
PLW1 26.60000038 W

F2 - Processing parameters
SI 262144
SF 600.1300147 MHz
WDW EM
SSB 0
LB 0.10 Hz
GB 0
PC 1.00

12

^{13}C NMR

151 MHz

$CDCl_3$

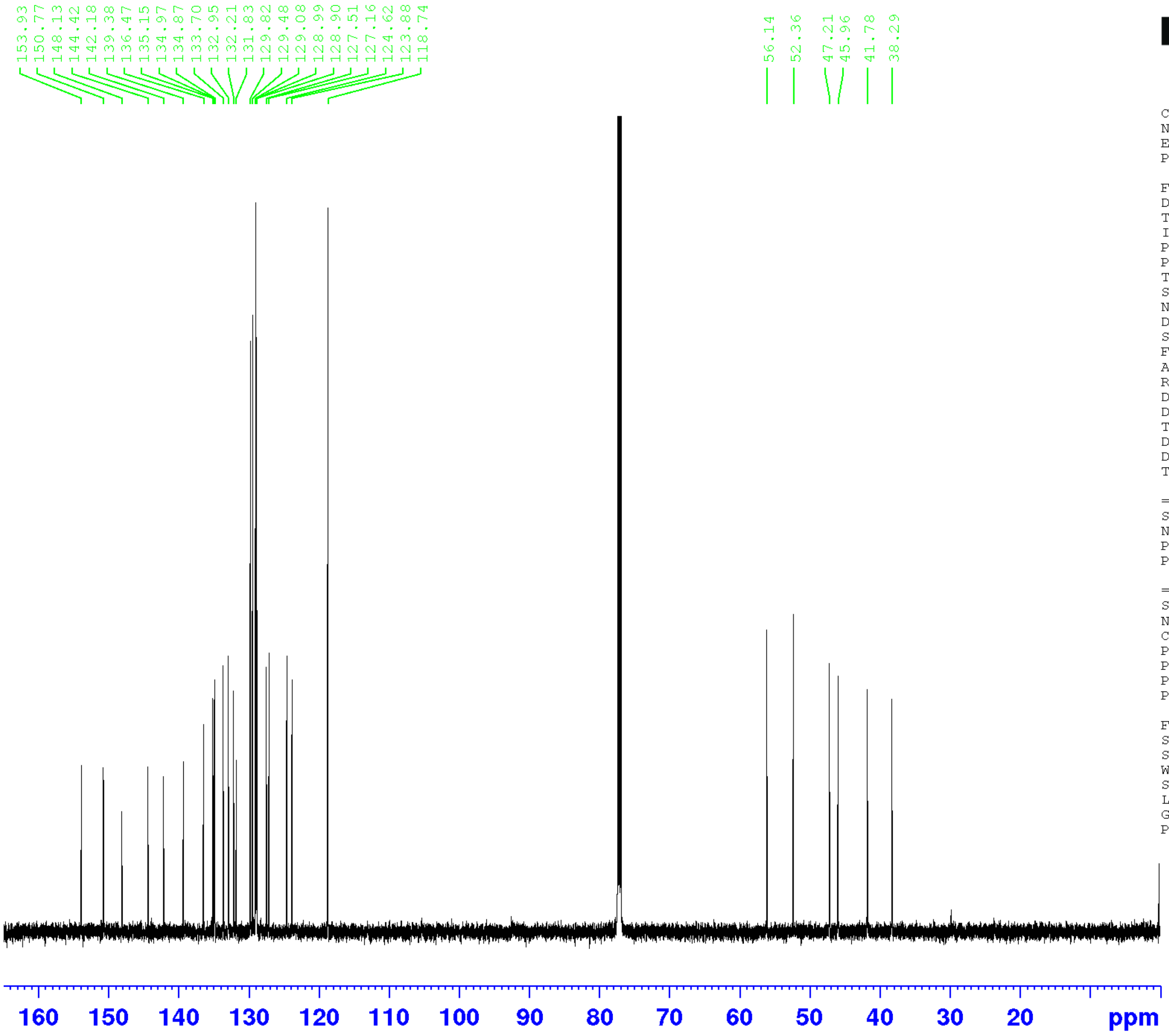

Current Data Parameters
NAME ZL 2.32 FINAL 13C 600 MHz
EXPNO 11
PROCNO 1

F2 - Acquisition Parameters
Date_ 20170825
Time 7.10
INSTRUM spect
PROBHD 5 mm PABBO BB/
PULPROG zgpg30
TD 119044
SOLVENT CDCl3
NS 2048
DS 4
SWH 37500.000 Hz
FIDRES 0.315010 Hz
AQ 1.5872533 sec
RG 186.92
DW 13.333 usec
DE 7.73 usec
TE 298.1 K
D1 1.00000000 sec
D11 0.03000000 sec
TD0 1

======== CHANNEL f1 ========
SFO1 150.9194058 MHz
NUC1 13C
P1 11.80 usec
PLW1 85.00000000 W

======== CHANNEL f2 ========
SFO2 600.1324005 MHz
NUC2 1H
CPDPRG[2 waltz64
PCPD2 80.00 usec
PLW2 27.00000000 W
PLW12 0.43891999 W
PLW13 0.28090999 W

F2 - Processing parameters
SI 131072
SF 150.9027899 MHz
WDW EM
SSB 0
LB 1.00 Hz
GB 0
PC 1.40

S5

^{1}H NMR

600 MHz

$CDCl_3$

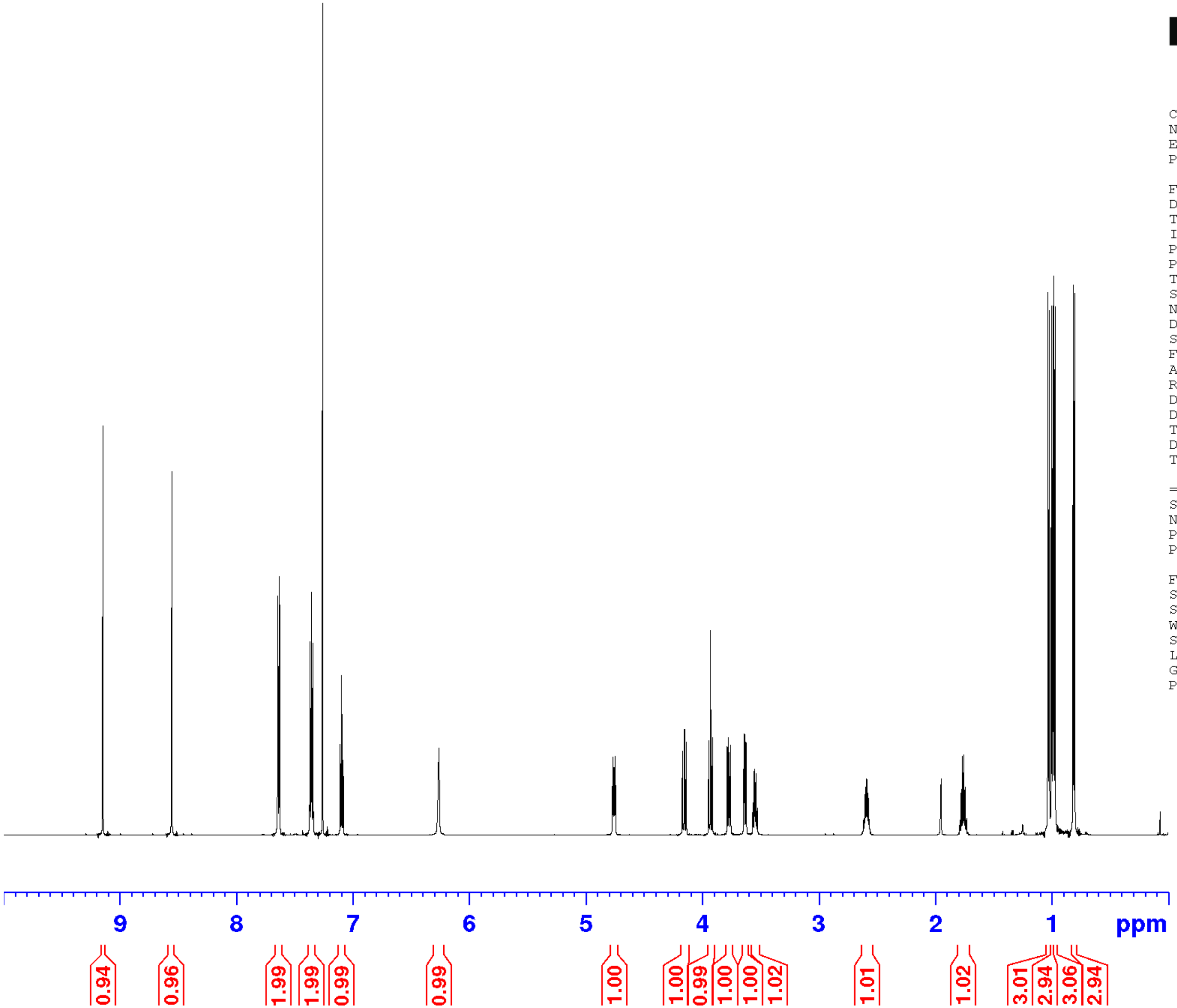

Current Data Parameters
NAME ZL 2.20 FINAL 13C 600 MHz
EXPNO 10
PROCNO 1

F2 - Acquisition Parameters
Date_ 20170824
Time 1.57
INSTRUM spect
PROBHD 5 mm PABBO BB/
PULPROG zg30
TD 180286
SOLVENT CDCl3
NS 16
DS 0
SWH 18028.846 Hz
FIDRES 0.100001 Hz
AQ 4.9999318 sec
RG 49.63
DW 27.733 usec
DE 7.60 usec
TE 298.1 K
D1 0.10000000 sec
TD0 1

======== CHANNEL f1 ========
SFO1 600.1337060 MHz
NUC1 1H
P1 10.00 usec
PLW1 26.60000038 W

F2 - Processing parameters
SI 262144
SF 600.1300146 MHz
WDW EM
SSB 0
LB 0.10 Hz
GB 0
PC 1.00

S5

^{13}C NMR

151 MHz

$CDCl_3$

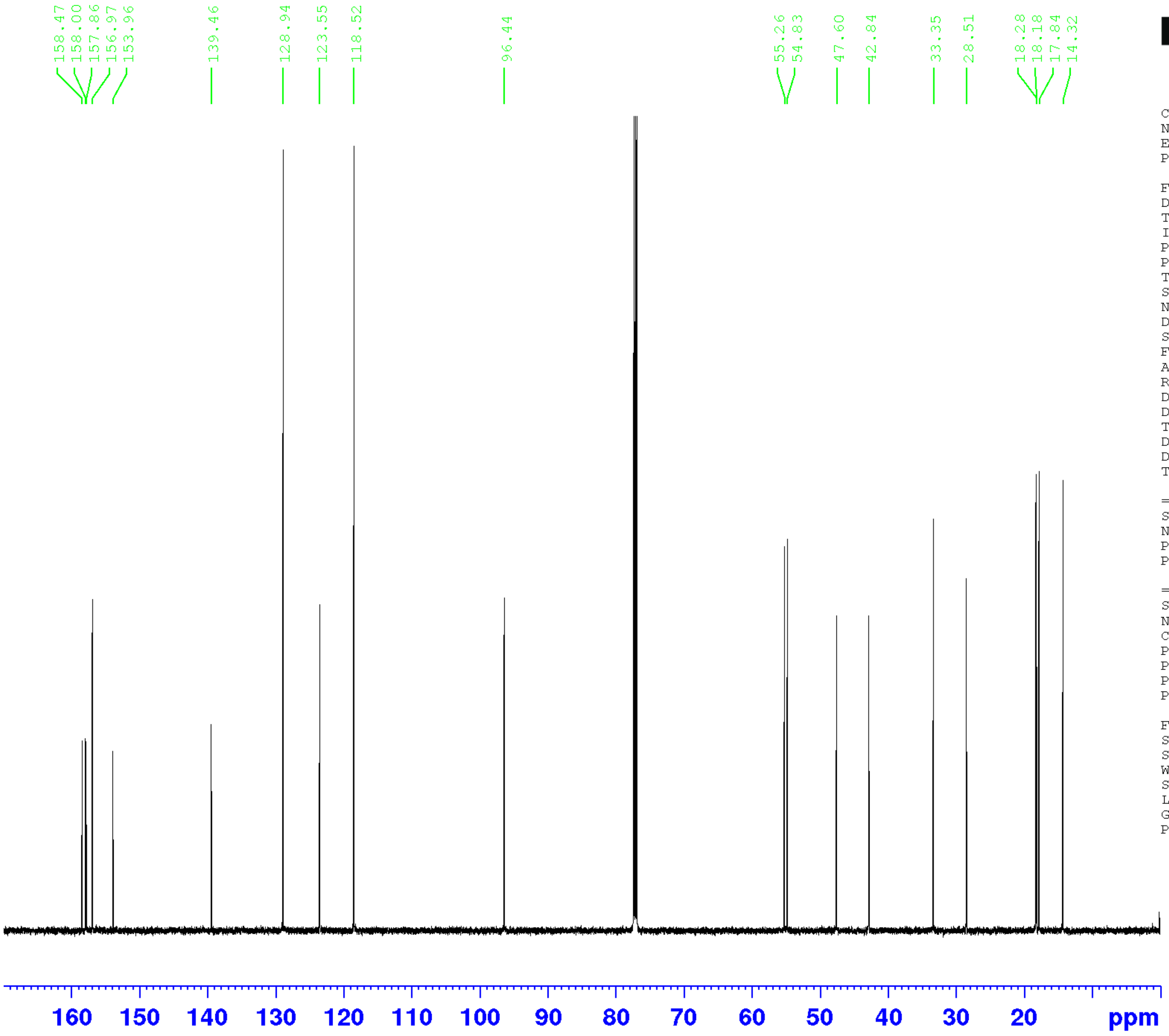


```
Current Data Parameters
NAME      ZL 2.20 FINAL 13C 600 MHz
EXPNO                    11
PROCNO                   1

F2 - Acquisition Parameters
Date_              20170824
Time                   2.42
INSTRUM               spect
PROBHD      5 mm PABBO BB/
PULPROG             zgpg30
TD                   119044
SOLVENT               CDCl3
NS                     1024
DS                        4
SWH             37500.000 Hz
FIDRES           0.315010 Hz
AQ              1.5872533 sec
RG                   186.92
DW                   13.333 usec
DE                     7.73 usec
TE                    298.1 K
D1               1.00000000 sec
D11              0.03000000 sec
TD0                       1

======== CHANNEL f1 ========
SFO1          150.9194058 MHz
NUC1                    13C
P1                    11.80 usec
PLW1            85.00000000 W

======== CHANNEL f2 ========
SFO2          600.1324005 MHz
NUC2                     1H
CPDPRG[2            waltz64
PCPD2                 80.00 usec
PLW2            27.00000000 W
PLW12            0.43891999 W
PLW13            0.28090999 W

F2 - Processing parameters
SI                   131072
SF            150.9027937 MHz
WDW                      EM
SSB        0
LB                     1.00 Hz
GB         0
PC                     1.40
```

S6

^{1}H NMR

600 MHz

$CDCl_3$

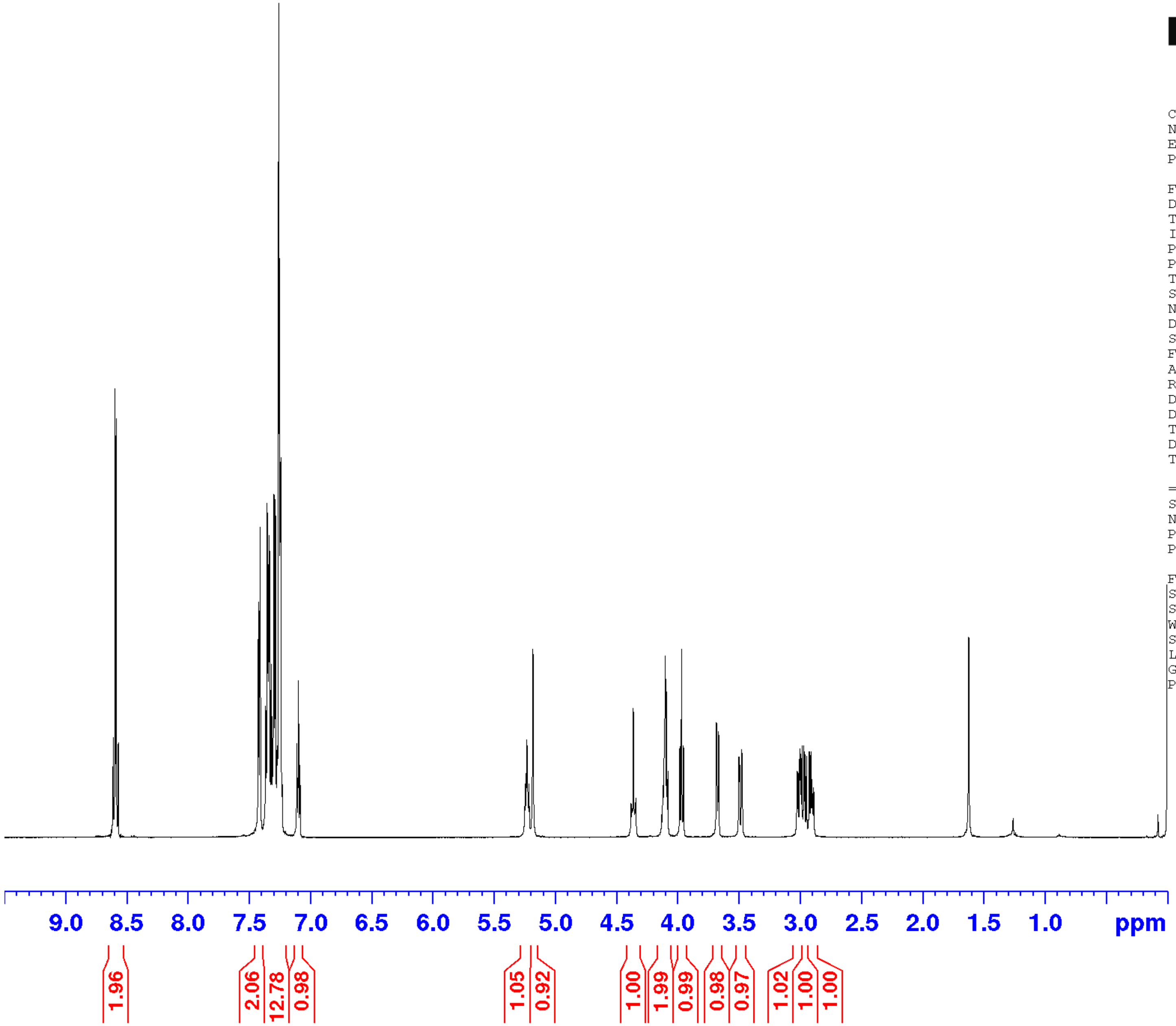


```
Current Data Parameters
NAME      ZL 2.11 FINAL 13C 600 MHz
EXPNO                    10
PROCNO                    1

F2 - Acquisition Parameters
Date_              20170823
Time                   3.14
INSTRUM               spect
PROBHD      5 mm PABBO BB/
PULPROG                zg30
TD                   180286
SOLVENT               CDCl3
NS                       16
DS                        0
SWH              18028.846 Hz
FIDRES            0.100001 Hz
AQ               4.9999318 sec
RG                     97.5
DW                   27.733 usec
DE                     7.60 usec
TE                    300.0 K
D1               0.10000000 sec
TD0                       1

======== CHANNEL f1 ========
SFO1           600.1337060 MHz
NUC1                     1H
P1                    10.00 usec
PLW1            26.60000038 W

F2 - Processing parameters
SI                   262144
SF              600.1300146 MHz
WDW                      EM
SSB        0
LB                     0.10 Hz
GB         0
PC                     1.00
```

S6

^{13}C NMR

151 MHz

$CDCl_3$

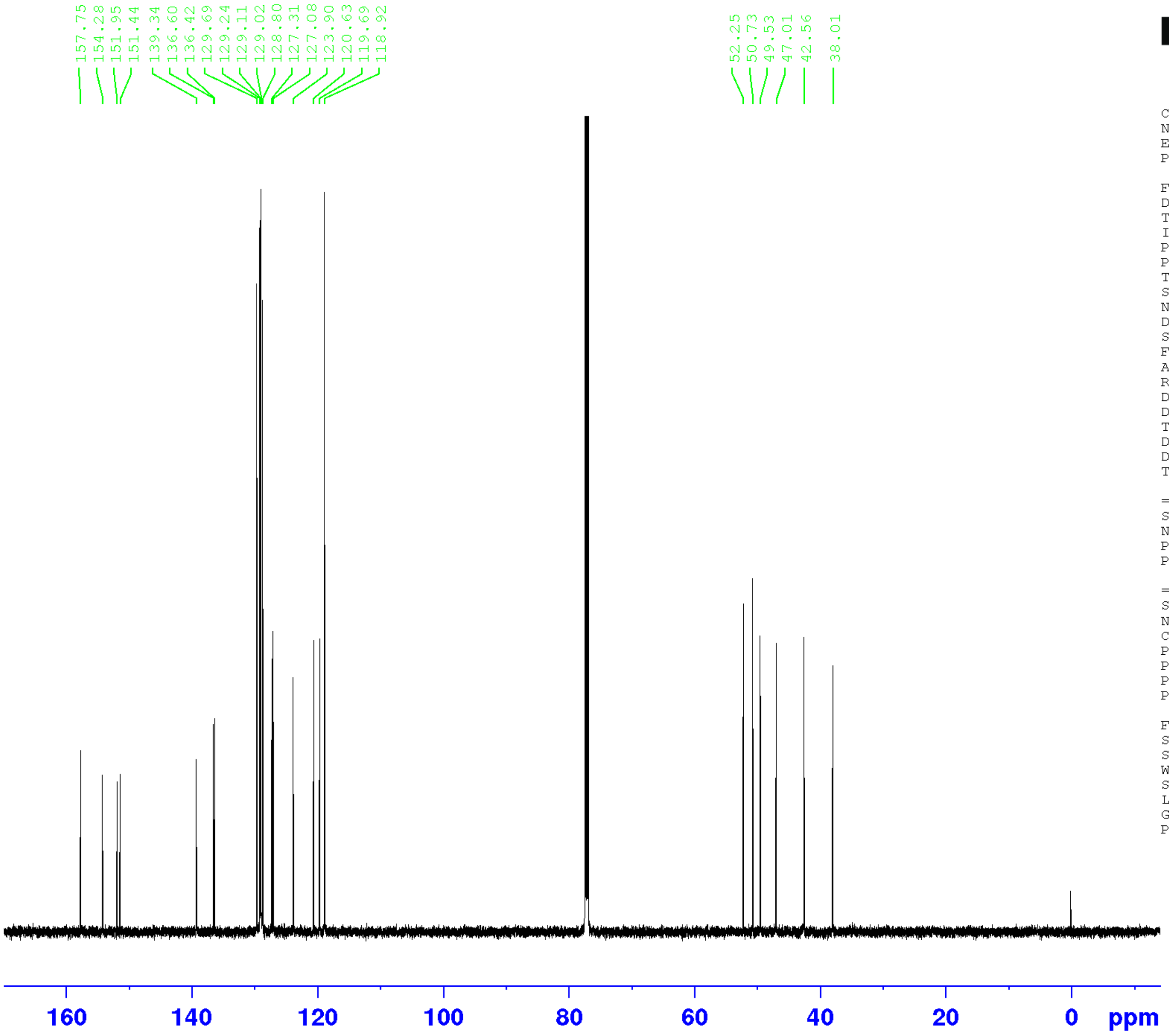


```
Current Data Parameters
NAME       ZL 2.11 FINAL 13C 600 MHz
EXPNO                    11
PROCNO                    1

F2 - Acquisition Parameters
Date_              20170823
Time                   4.45
INSTRUM               spect
PROBHD     5 mm PABBO BB/
PULPROG              zgpg30
TD                   119044
SOLVENT               CDCl3
NS                     2048
DS                        4
SWH               37500.000 Hz
FIDRES             0.315010 Hz
AQ                1.5872533 sec
RG                   186.92
DW                   13.333 usec
DE                     7.73 usec
TE                    300.0 K
D1               1.00000000 sec
D11              0.03000000 sec
TD0                       1

======== CHANNEL f1 ========
SFO1            150.9194058 MHz
NUC1                    13C
P1                    11.80 usec
PLW1            85.00000000 W

======== CHANNEL f2 ========
SFO2            600.1324005 MHz
NUC2                     1H
CPDPRG[2            waltz64
PCPD2                 80.00 usec
PLW2            27.00000000 W
PLW12            0.43891999 W
PLW13            0.28090999 W

F2 - Processing parameters
SI                   131072
SF              150.9027908 MHz
WDW                      EM
SSB        0
LB                     1.00 Hz
GB         0
PC                     1.40
```

S7

^{1}H NMR

600 MHz

$CDCl_3$

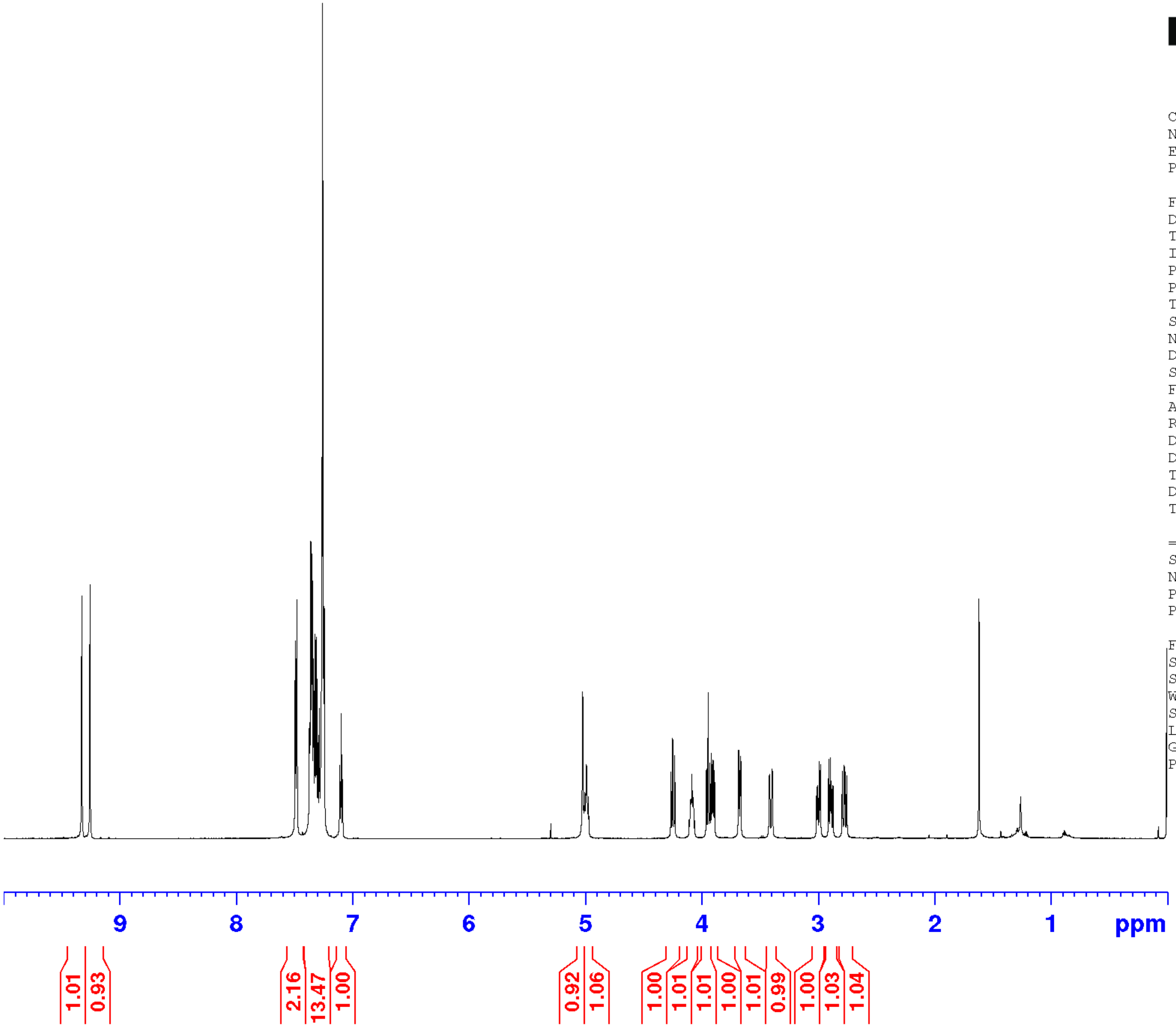


```
Current Data Parameters
NAME      ZL 2.33 FINAL 600 MHz
EXPNO                    10
PROCNO                    1

F2 - Acquisition Parameters
Date_              20170828
Time                  14.40
INSTRUM               spect
PROBHD    5 mm PABBO BB/
PULPROG                zg30
TD                   180286
SOLVENT               CDCl3
NS                       16
DS                        0
SWH              18028.846 Hz
FIDRES            0.100001 Hz
AQ               4.9999318 sec
RG                    97.5
DW                  27.733 usec
DE                    7.60 usec
TE                   298.1 K
D1              0.10000000 sec
TD0                       1

======== CHANNEL f1 ========
SFO1          600.1337060 MHz
NUC1                   1H
P1                   10.00 usec
PLW1           26.60000038 W

F2 - Processing parameters
SI                   262144
SF            600.1300145 MHz
WDW                      EM
SSB       0
LB                    0.10 Hz
GB        0
PC                     1.00
```

S7

^{13}C NMR

151 MHz

$CDCl_3$

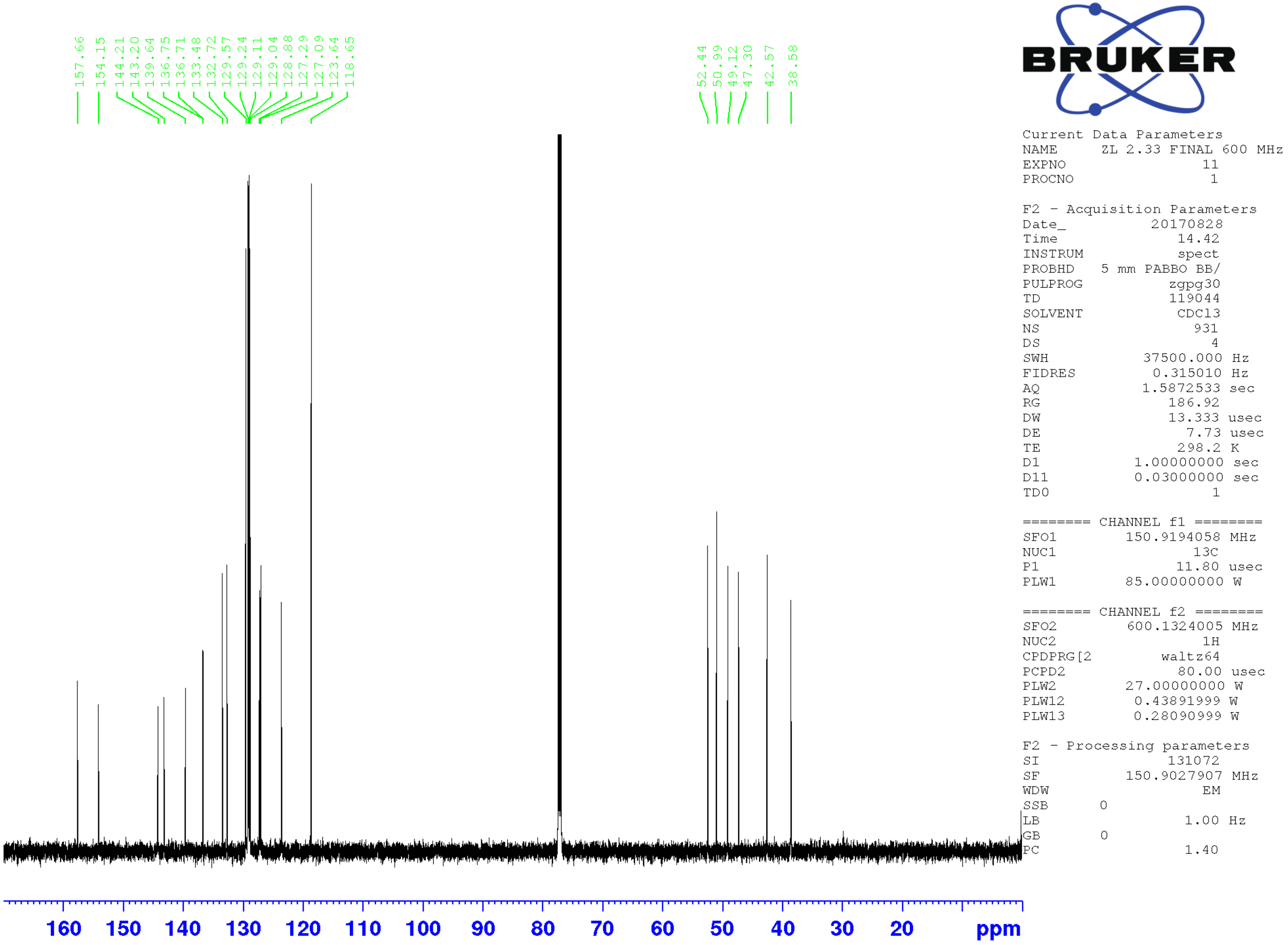

13

^{1}H NMR

600 MHz

$CDCl_3$

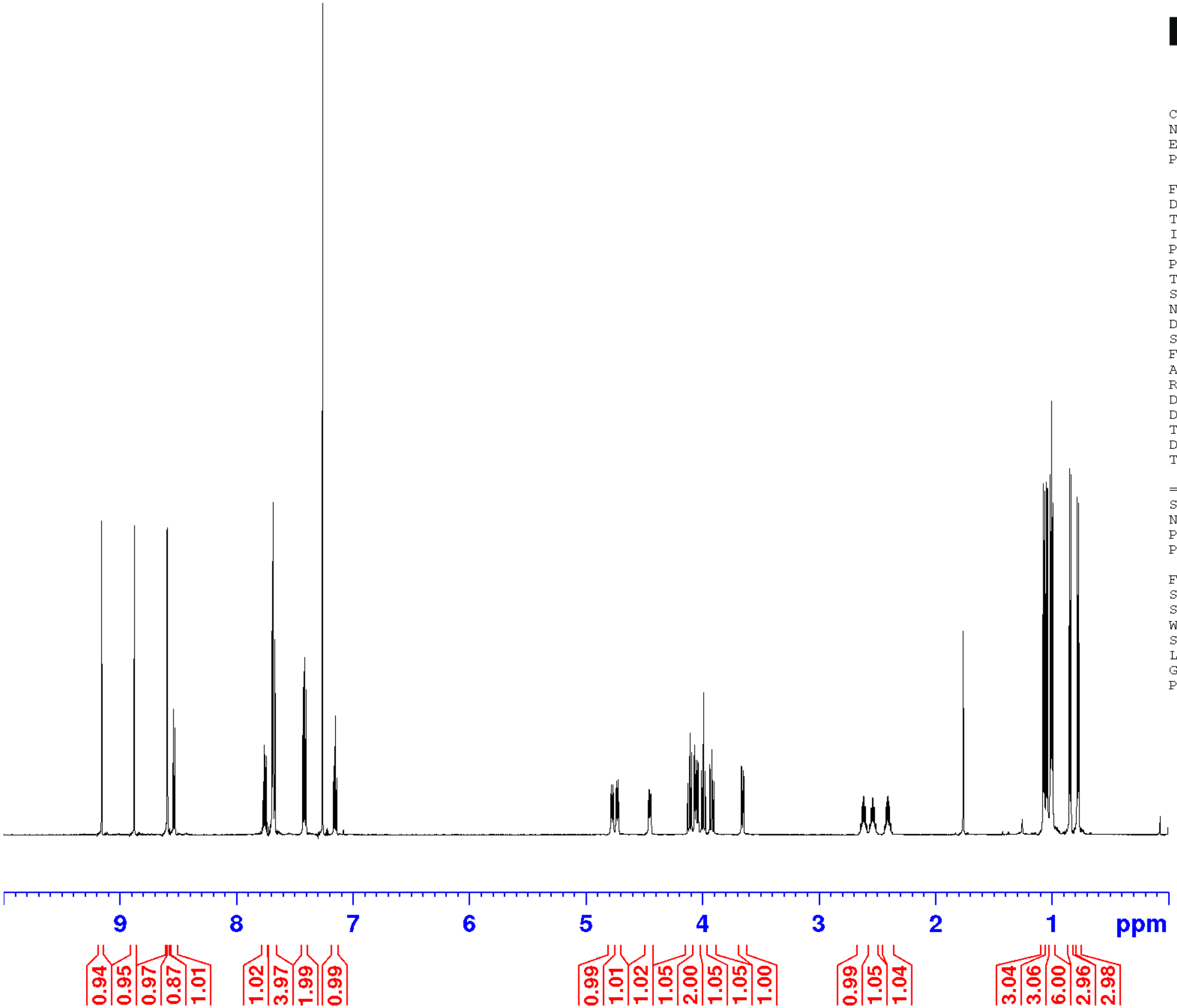


```
Current Data Parameters
NAME      ZL 2.22 FINAL 13C 600 MHz
EXPNO                   10
PROCNO                   1

F2 - Acquisition Parameters
Date_            20170825
Time                 3.08
INSTRUM             spect
PROBHD     5 mm PABBO BB/
PULPROG              zg30
TD                 180286
SOLVENT             CDCl3
NS                     16
DS                      0
SWH             18028.846 Hz
FIDRES           0.100001 Hz
AQ              4.9999318 sec
RG                  55.43
DW                 27.733 usec
DE                   7.60 usec
TE                  298.1 K
D1             0.10000000 sec
TD0                     1

======== CHANNEL f1 ========
SFO1          600.1337060 MHz
NUC1                   1H
P1                  10.00 usec
PLW1          26.60000038 W

F2 - Processing parameters
SI                 262144
SF            600.1300147 MHz
WDW                    EM
SSB       0
LB                   0.10 Hz
GB        0
PC                   1.00
```

13

^{13}C NMR

151 MHz

$CDCl_3$

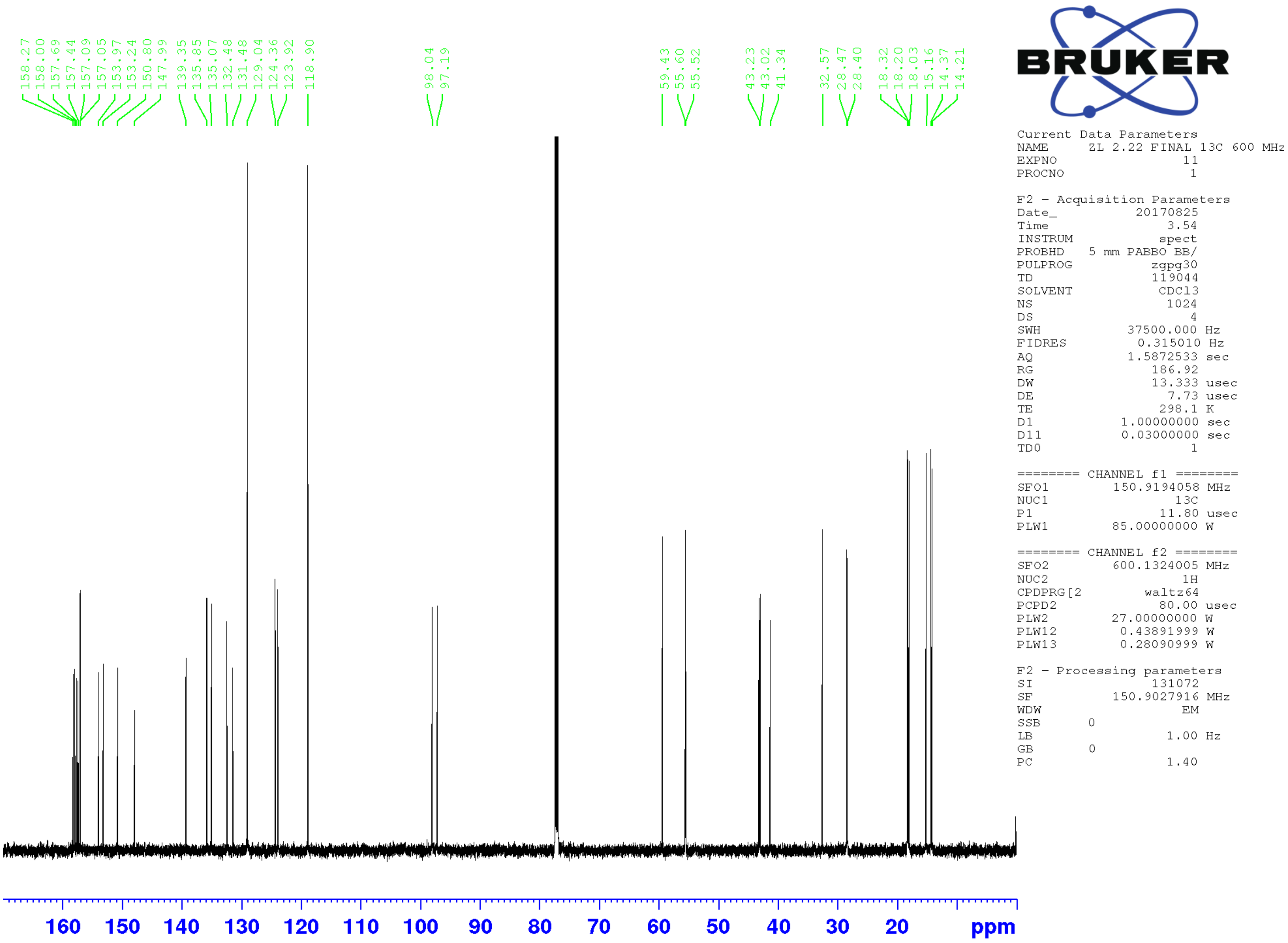

14

^1H NMR

600 MHz

$CDCl_3$

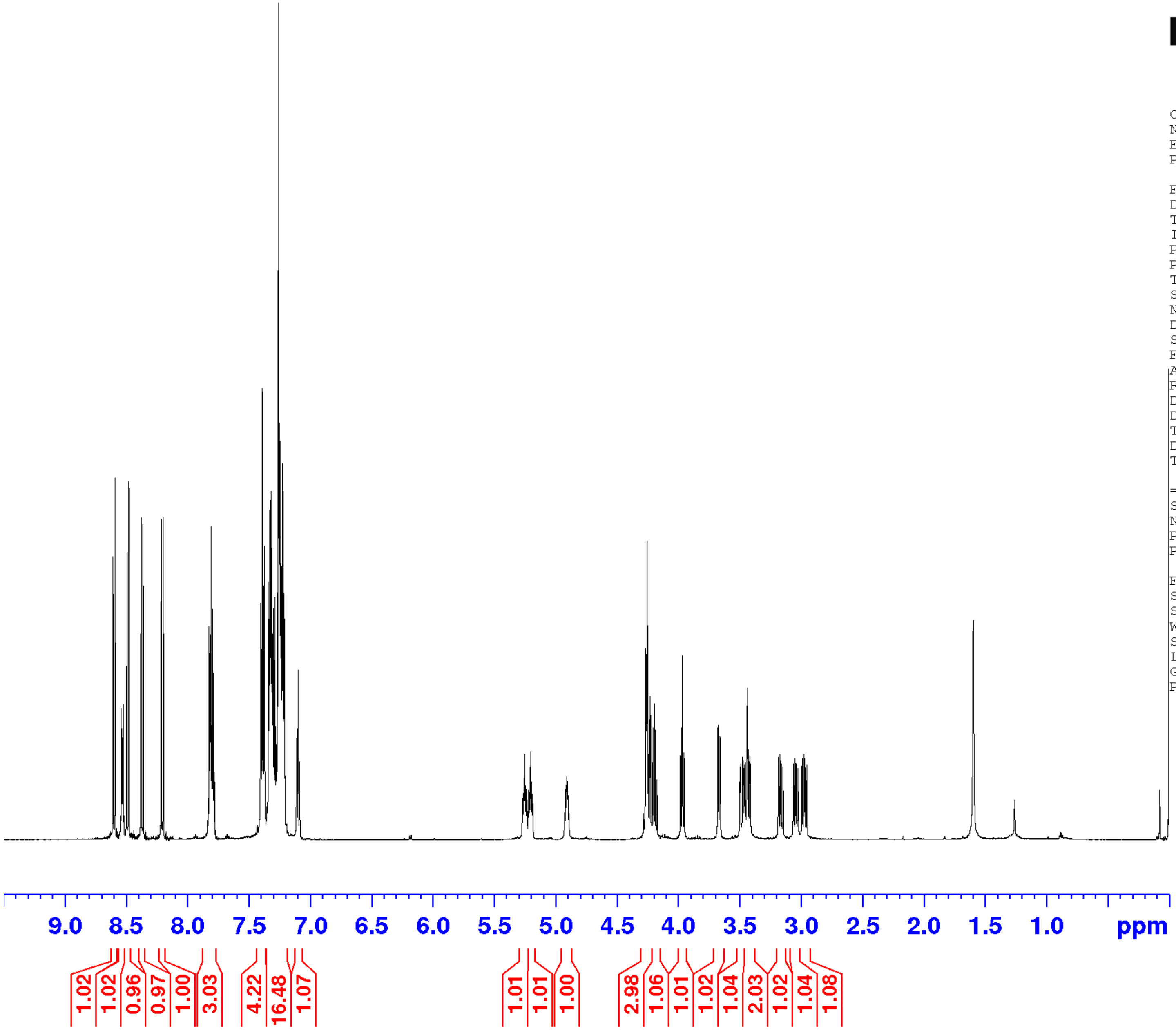


```
Current Data Parameters
NAME       ZL 2.12 FINAL 13C 600 MHz
EXPNO                    10
PROCNO                    1

F2 - Acquisition Parameters
Date_              20170823
Time                  17.00
INSTRUM               spect
PROBHD    5 mm PABBO BB/
PULPROG                zg30
TD                   180286
SOLVENT               CDCl3
NS                       16
DS                        0
SWH              18028.846 Hz
FIDRES            0.100001 Hz
AQ               4.9999318 sec
RG                     97.5
DW                   27.733 usec
DE                     7.60 usec
TE                    298.1 K
D1              0.10000000 sec
TD0                       1

======== CHANNEL f1 ========
SFO1           600.1337060 MHz
NUC1                     1H
P1                    10.00 usec
PLW1            26.60000038 W

F2 - Processing parameters
SI                   262144
SF              600.1300144 MHz
WDW                      EM
SSB      0
LB                     0.10 Hz
GB       0
PC                     1.00
```

14

^{13}C NMR

151 MHz

$CDCl_3$

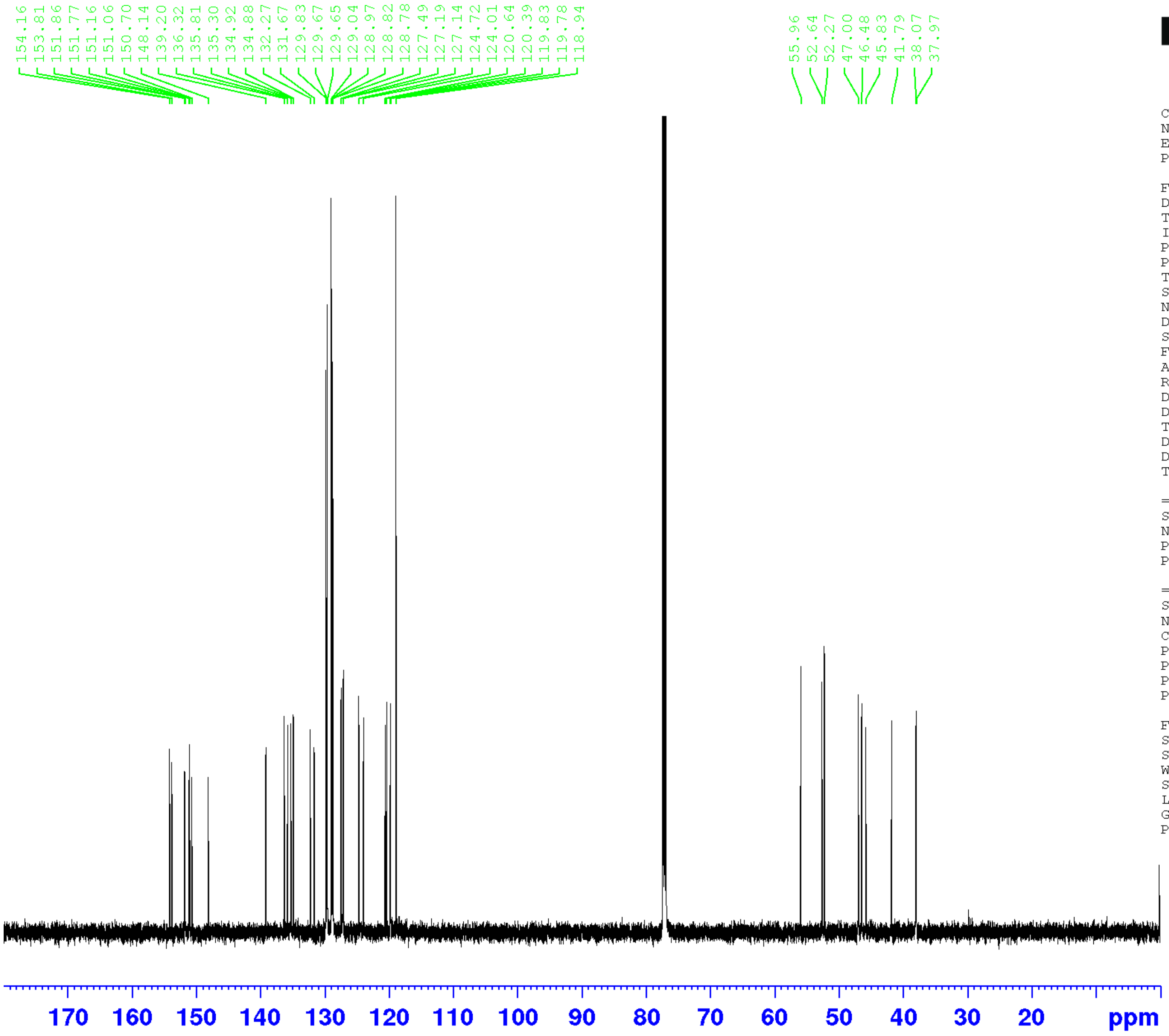

BRUKER

```
Current Data Parameters
NAME      ZL 2.12 FINAL 13C 600 MHz
EXPNO                   11
PROCNO                   1

F2 - Acquisition Parameters
Date_             20170823
Time                 18.32
INSTRUM              spect
PROBHD     5 mm PABBO BB/
PULPROG            zgpg30
TD                  119044
SOLVENT              CDCl3
NS                    2048
DS                       4
SWH             37500.000 Hz
FIDRES           0.315010 Hz
AQ              1.5872533 sec
RG                  186.92
DW                  13.333 usec
DE                    7.73 usec
TE                   298.1 K
D1              1.00000000 sec
D11             0.03000000 sec
TD0                      1

======== CHANNEL f1 ========
SFO1          150.9194058 MHz
NUC1                   13C
P1                   11.80 usec
PLW1           85.00000000 W

======== CHANNEL f2 ========
SFO2          600.1324005 MHz
NUC2                    1H
CPDPRG[2           waltz64
PCPD2                80.00 usec
PLW2           27.00000000 W
PLW12           0.43891999 W
PLW13           0.28090999 W

F2 - Processing parameters
SI                  131072
SF            150.9027917 MHz
WDW                     EM
SSB       0
LB                    1.00 Hz
GB        0
PC                    1.40
```

15

^{1}H NMR

600 MHz

$CDCl_3$

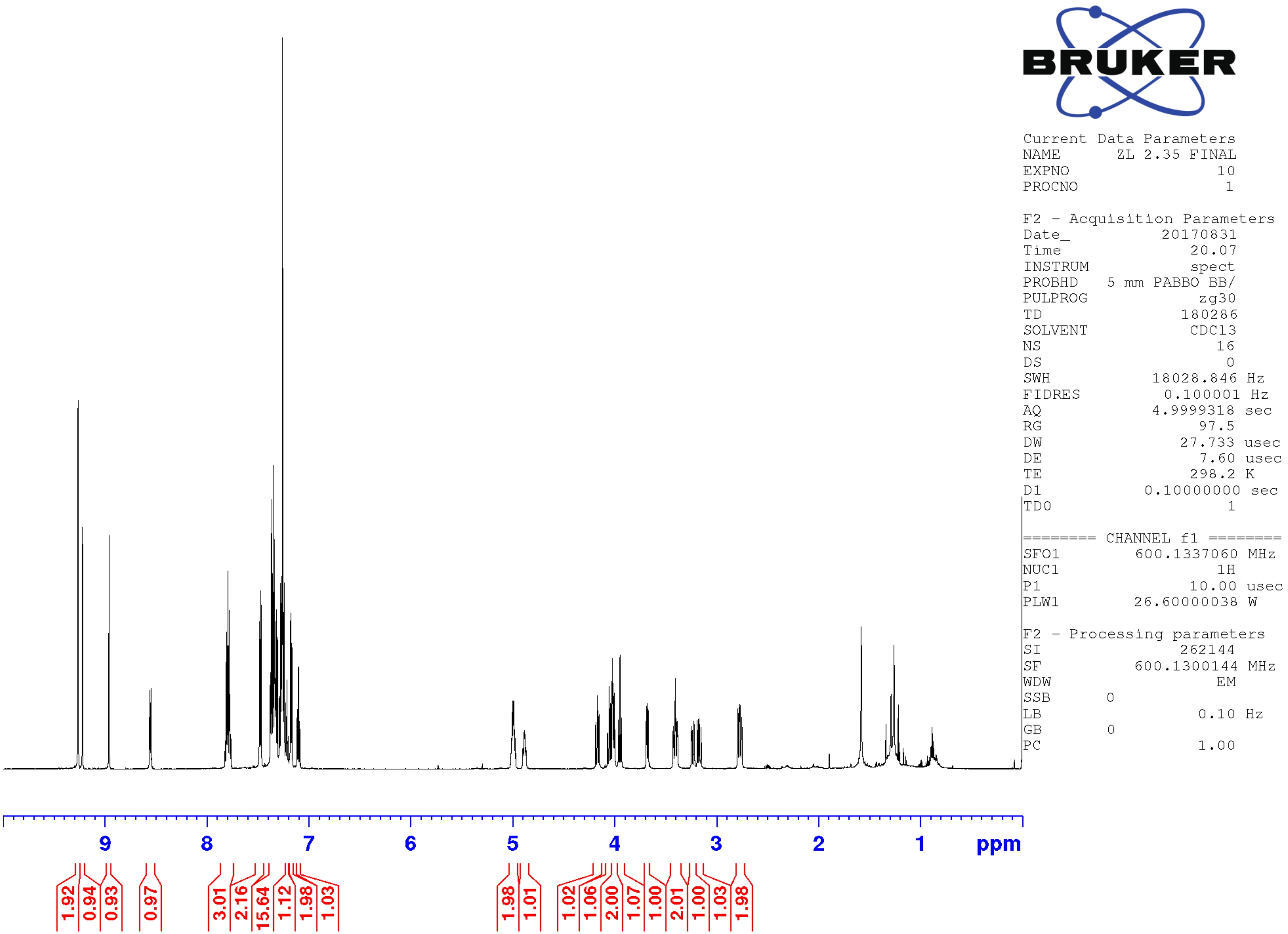

15

^{13}C NMR

151 MHz

$CDCl_3$

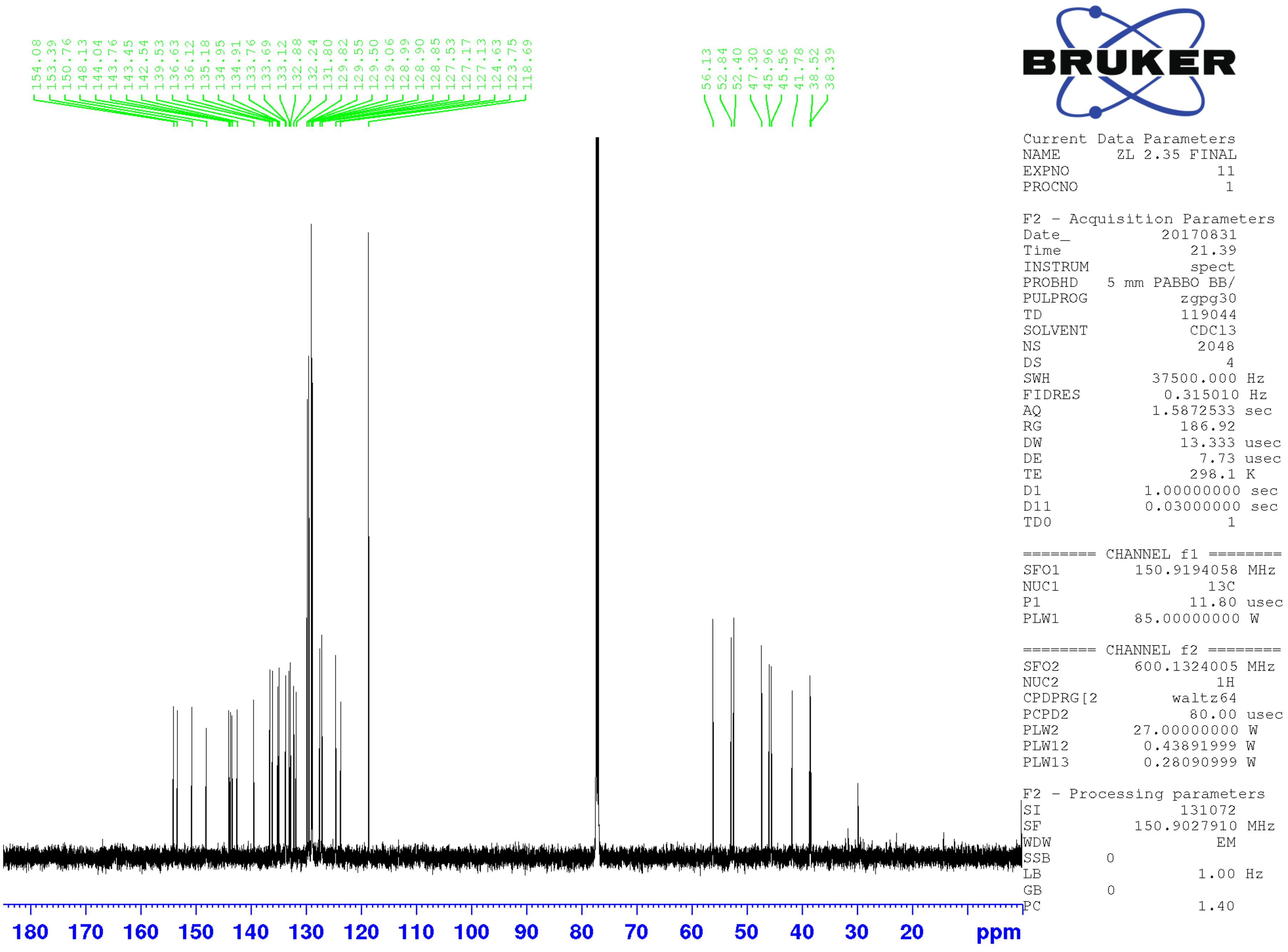

S8

^{1}H NMR

600 MHz

$CDCl_3$

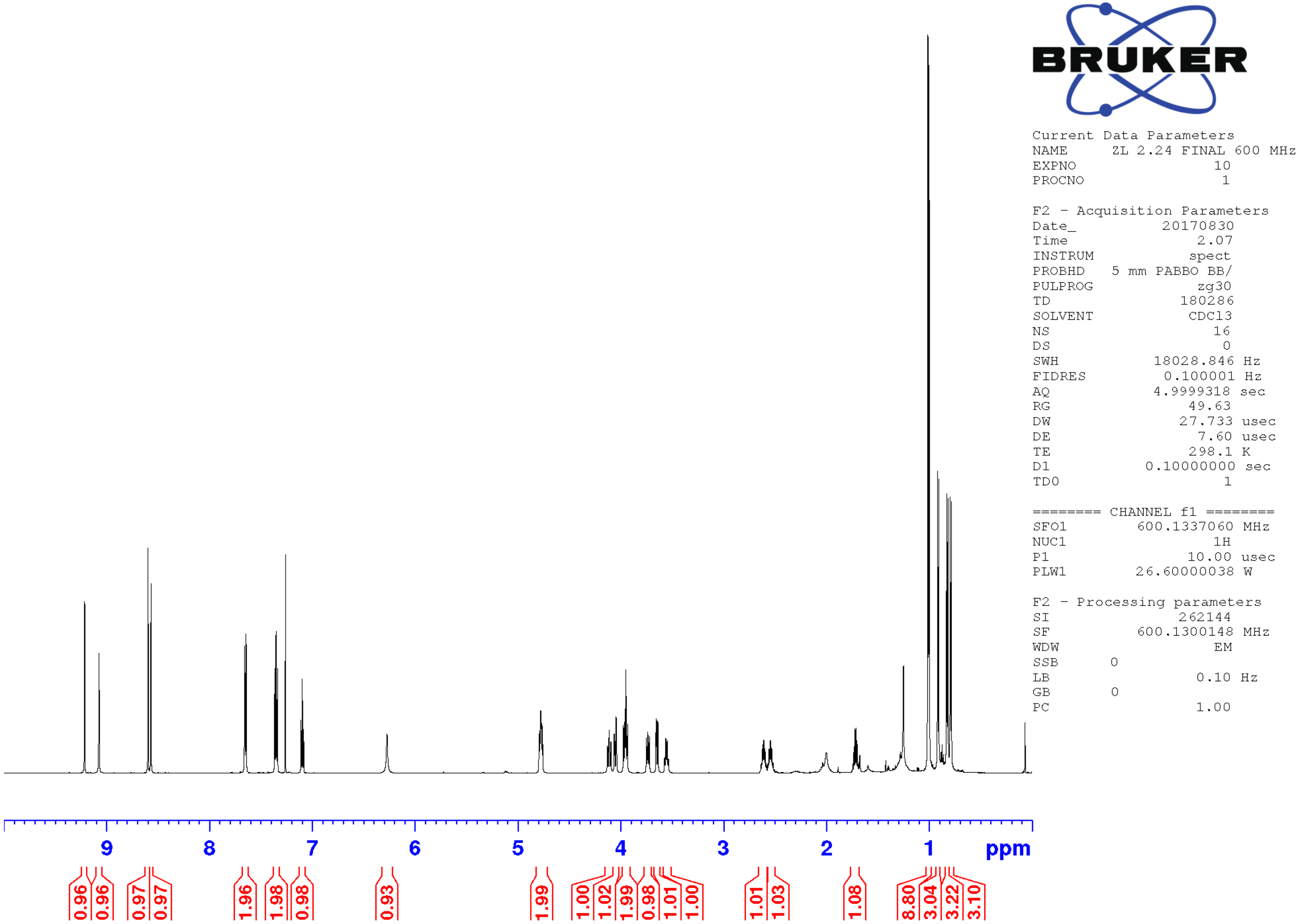

S8

^{13}C NMR

151 MHz

$CDCl_3$

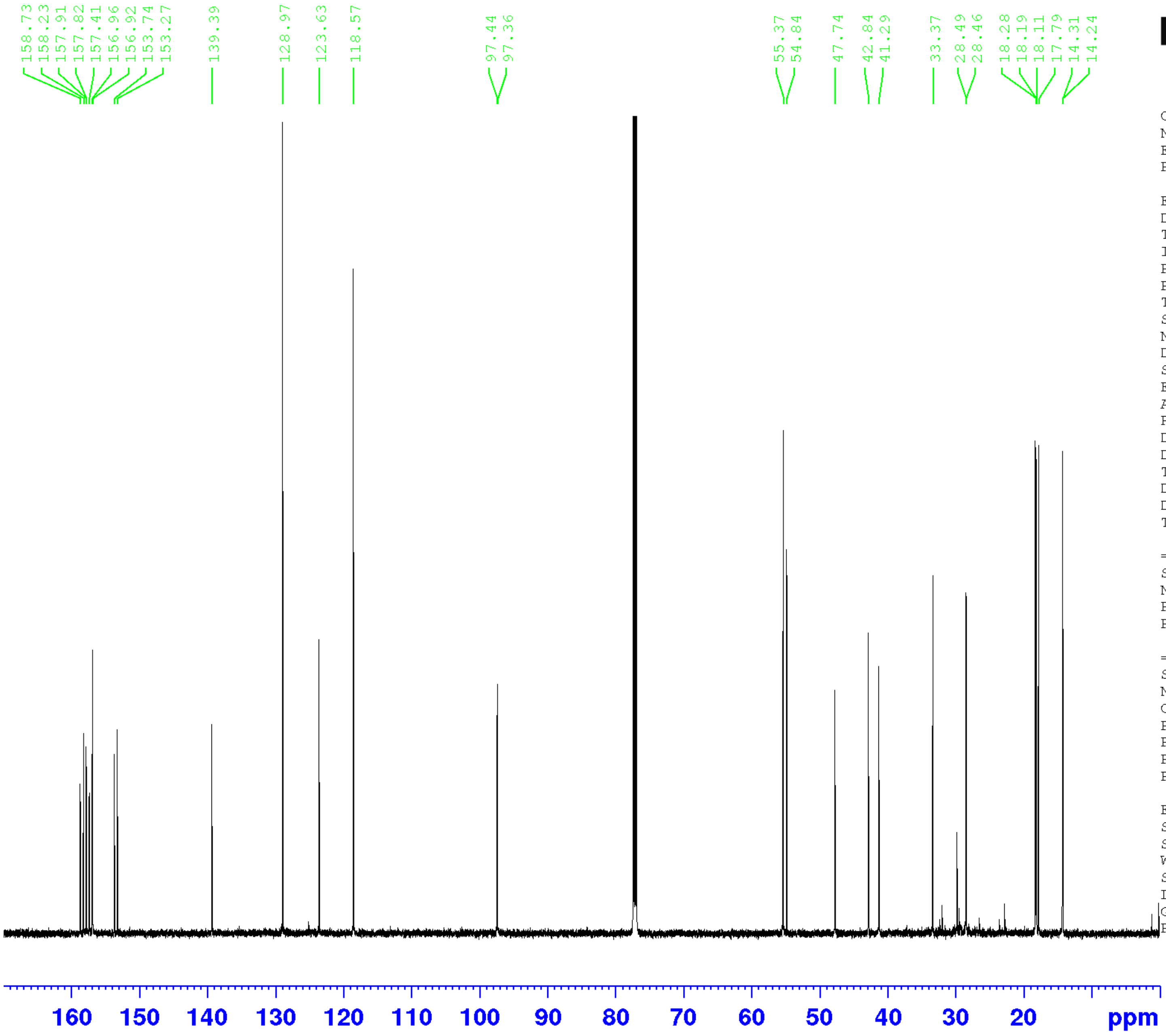


```
Current Data Parameters
NAME      ZL 2.24 FINAL 600 MHz
EXPNO                  11
PROCNO                 1

F2 - Acquisition Parameters
Date_            20170830
Time                 5.08
INSTRUM             spect
PROBHD     5 mm PABBO BB/
PULPROG           zgpg30
TD                119044
SOLVENT            CDCl3
NS                  4096
DS                     4
SWH           37500.000 Hz
FIDRES         0.315010 Hz
AQ            1.5872533 sec
RG               186.92
DW               13.333 usec
DE                 7.73 usec
TE                298.2 K
D1           1.00000000 sec
D11          0.03000000 sec
TD0                    1

======== CHANNEL f1 ========
SFO1        150.9194058 MHz
NUC1                13C
P1                11.80 usec
PLW1        85.00000000 W

======== CHANNEL f2 ========
SFO2        600.1324005 MHz
NUC2                 1H
CPDPRG[2          waltz64
PCPD2             80.00 usec
PLW2        27.00000000 W
PLW12        0.43891999 W
PLW13        0.28090999 W

F2 - Processing parameters
SI                131072
SF          150.9027921 MHz
WDW                   EM
SSB      0
LB                 1.00 Hz
GB       0
PC                 1.40
```

16

^1H NMR

600 MHz

$CDCl_3$

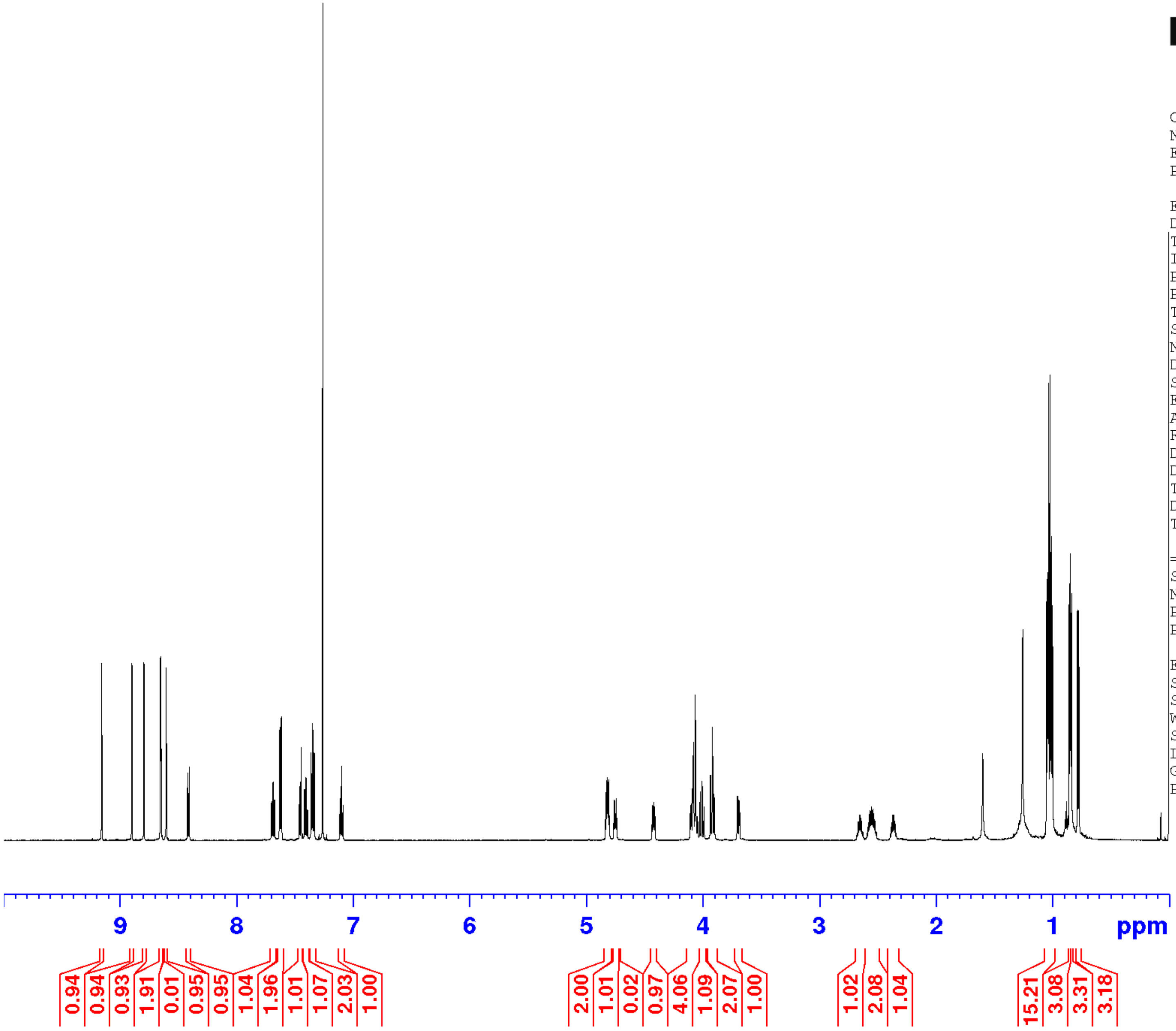

BRUKER

```
Current Data Parameters
NAME      ZL 2.34 new nmr data
EXPNO                       10
PROCNO                       1

F2 - Acquisition Parameters
Date_                 20171020
Time                     18.08
INSTRUM                  spect
PROBHD    5 mm PABBO BB/
PULPROG                   zg30
TD                      180286
SOLVENT                  CDCl3
NS                          16
DS                           0
SWH                  18028.846 Hz
FIDRES                0.100001 Hz
AQ                   4.9999318 sec
RG                        97.5
DW                      27.733 usec
DE                        7.60 usec
TE                       298.1 K
D1                  0.10000000 sec
TD0                          1

======== CHANNEL f1 ========
SFO1               600.1337060 MHz
NUC1                        1H
P1                       10.00 usec
PLW1               26.60000038 W

F2 - Processing parameters
SI                      262144
SF                 600.1300140 MHz
WDW                         EM
SSB       0
LB                        0.10 Hz
GB        0
PC                        1.00
```

16

^{13}C NMR

151 MHz

$CDCl_3$

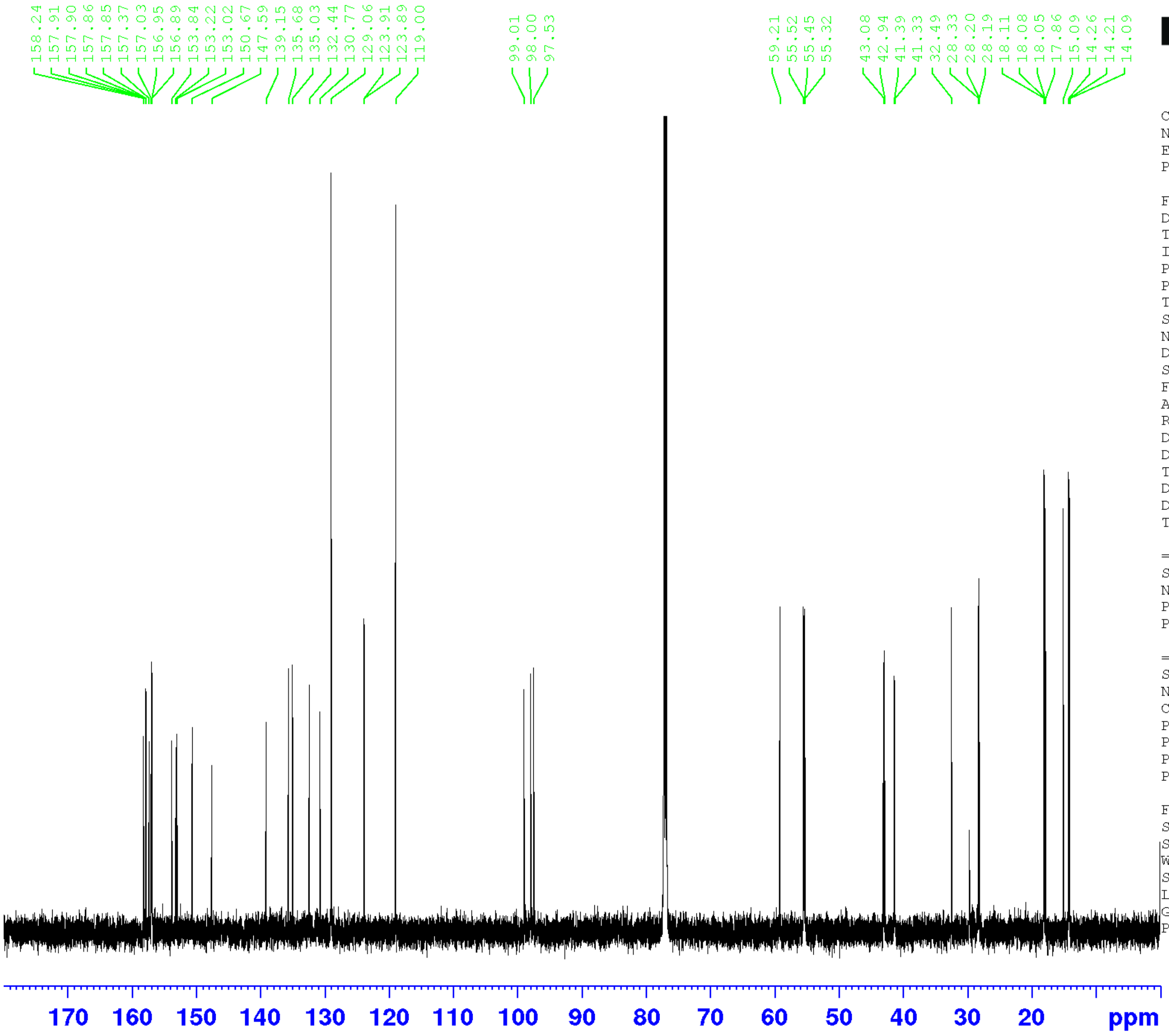

Current Data Parameters
NAME ZL 2.34 FINAL 600 MHz
EXPNO 11
PROCNO 1

F2 - Acquisition Parameters
Date_ 20170828
Time 18.16
INSTRUM spect
PROBHD 5 mm PABBO BB/
PULPROG zgpg30
TD 119044
SOLVENT CDCl3
NS 2048
DS 4
SWH 37500.000 Hz
FIDRES 0.315010 Hz
AQ 1.5872533 sec
RG 186.92
DW 13.333 usec
DE 7.73 usec
TE 298.1 K
D1 1.00000000 sec
D11 0.03000000 sec
TD0 1

======== CHANNEL f1 ========
SFO1 150.9194058 MHz
NUC1 13C
P1 11.80 usec
PLW1 85.00000000 W

======== CHANNEL f2 ========
SFO2 600.1324005 MHz
NUC2 1H
CPDPRG[2 waltz64
PCPD2 80.00 usec
PLW2 27.00000000 W
PLW12 0.43891999 W
PLW13 0.28090999 W

F2 - Processing parameters
SI 131072
SF 150.9027892 MHz
WDW EM
SSB 0
LB 1.00 Hz
GB 0
PC 1.40

S9

^{1}H NMR

600 MHz

$CDCl_3$

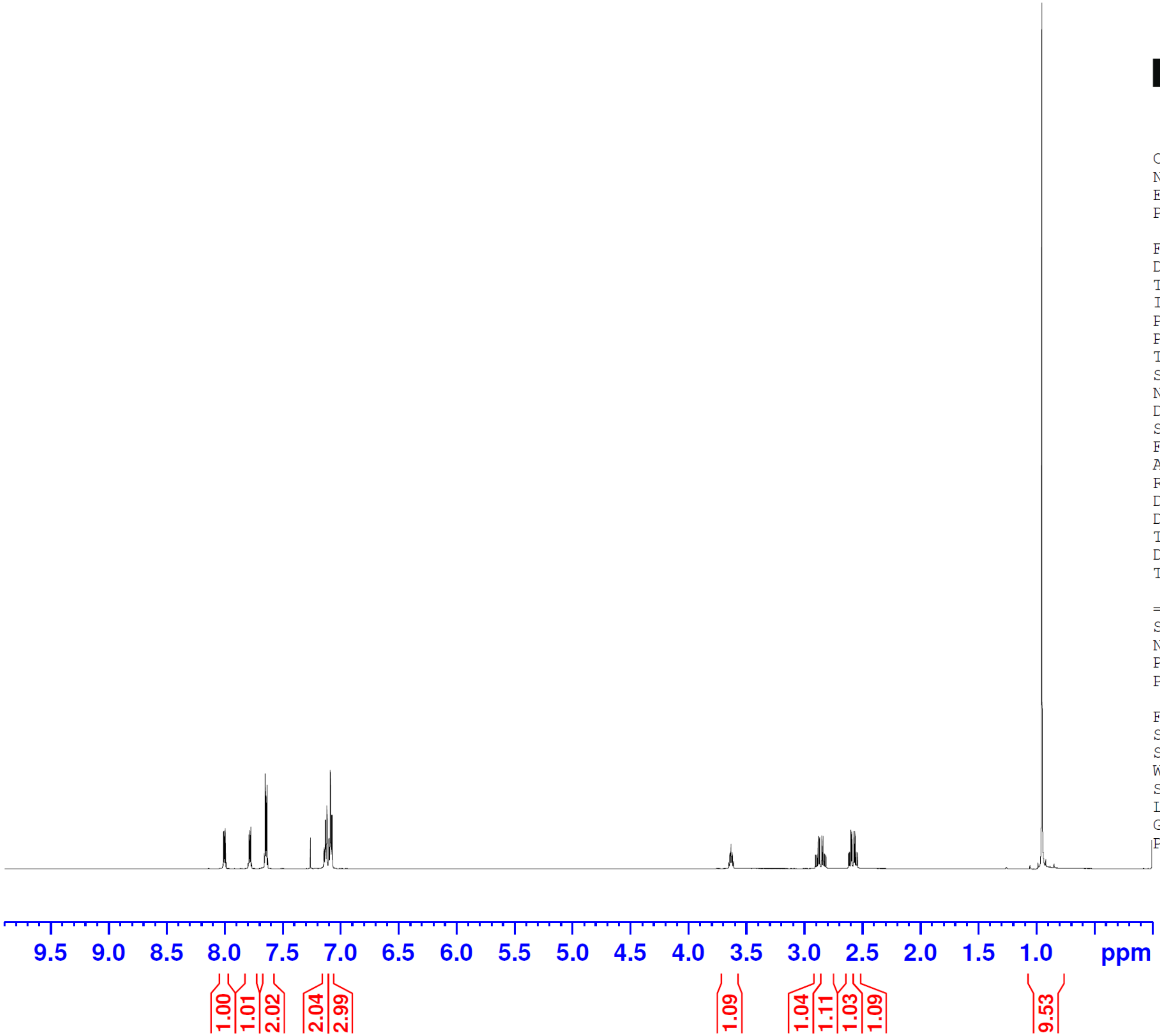

Current Data Parameters

NAME	pck 3.10
EXPNO	10
PROCNO	1

F2 - Acquisition Parameters

Date_	20180101
Time	14.57
INSTRUM	spect
PROBHD	5 mm PABBO BB/
PULPROG	zg30
TD	180286
SOLVENT	CDCl3
NS	16
DS	0
SWH	18028.846 Hz
FIDRES	0.100001 Hz
AQ	4.9999318 sec
RG	43.25
DW	27.733 usec
DE	7.60 usec
TE	298.1 K
D1	0.10000000 sec
TD0	1

======== CHANNEL f1 ========

SFO1	600.1337060 MHz
NUC1	1H
P1	10.00 usec
PLW1	26.60000038 W

F2 - Processing parameters

SI	262144
SF	600.1300142 MHz
WDW	EM
SSB	0
LB	0.10 Hz
GB	0
PC	1.00

S9

^{13}C NMR
(DEPT-Q)

151 MHz

$CDCl_3$

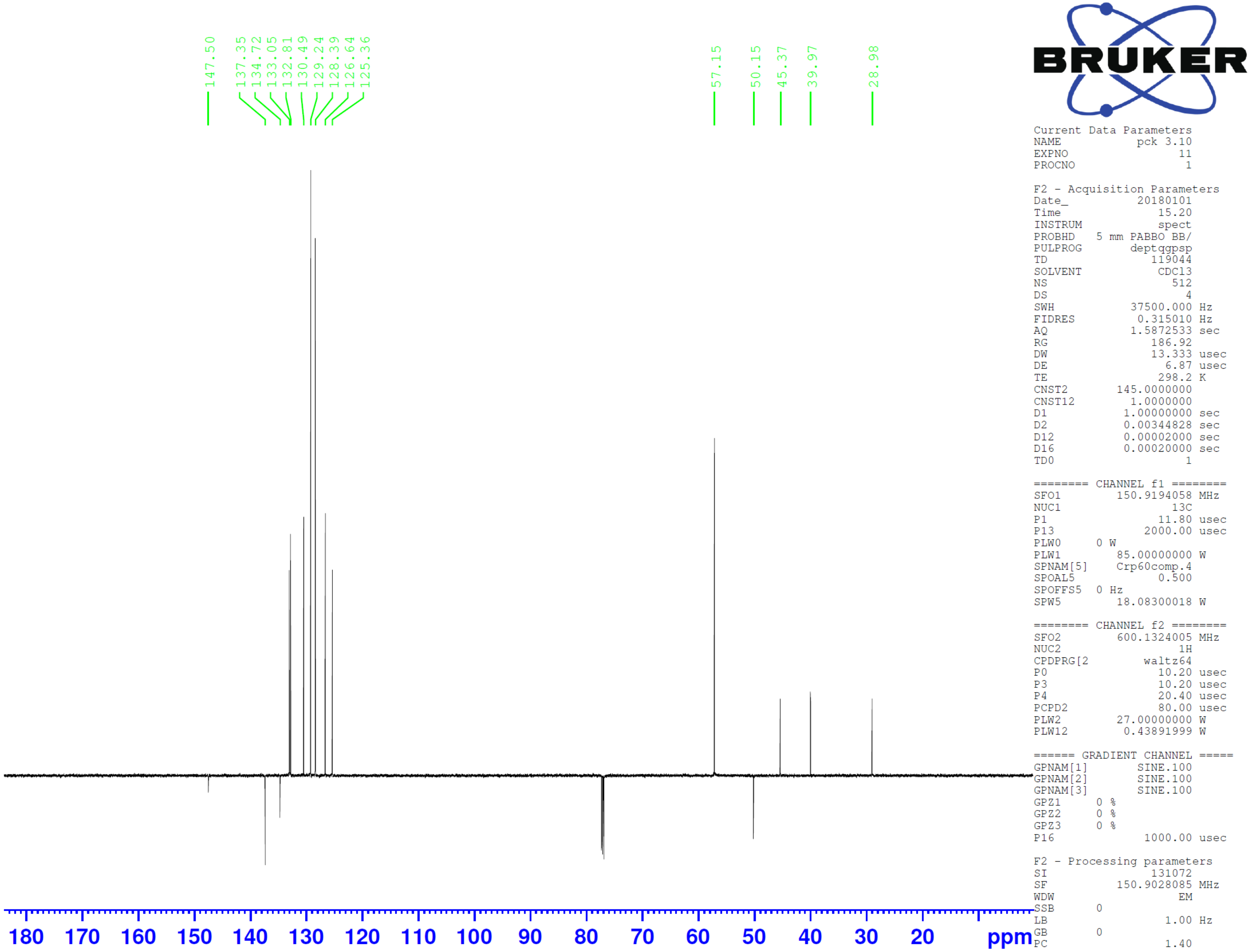

S10

^{1}H NMR

600 MHz

$CDCl_3$

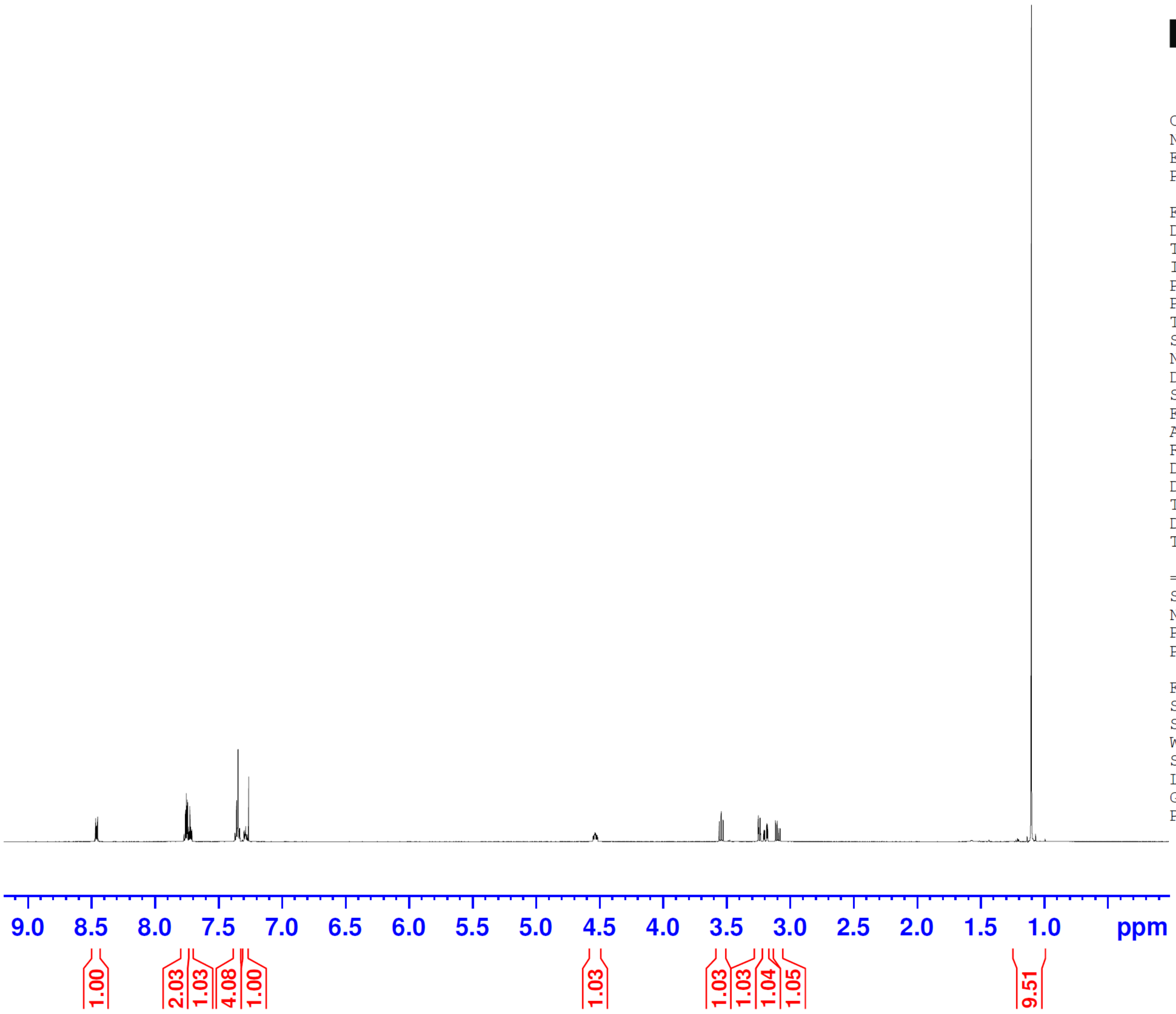


```
Current Data Parameters
NAME              pck 3.11
EXPNO                   20
PROCNO                   1

F2 - Acquisition Parameters
Date_             20180101
Time                 15.31
INSTRUM              spect
PROBHD     5 mm PABBO BB/
PULPROG               zg30
TD                  180286
SOLVENT              CDCl3
NS                      16
DS                       0
SWH             18028.846 Hz
FIDRES           0.100001 Hz
AQ              4.9999318 sec
RG                    97.5
DW                  27.733 usec
DE                    7.60 usec
TE                   298.1 K
D1             0.10000000 sec
TD0                      1

======== CHANNEL f1 ========
SFO1          600.1337060 MHz
NUC1                    1H
P1                   10.00 usec
PLW1          26.60000038 W

F2 - Processing parameters
SI                  262144
SF            600.1300142 MHz
WDW                     EM
SSB         0
LB                    0.10 Hz
GB          0
PC                    1.00
```

S10

^{13}C NMR
(DEPT-Q)

151 MHz

$CDCl_3$

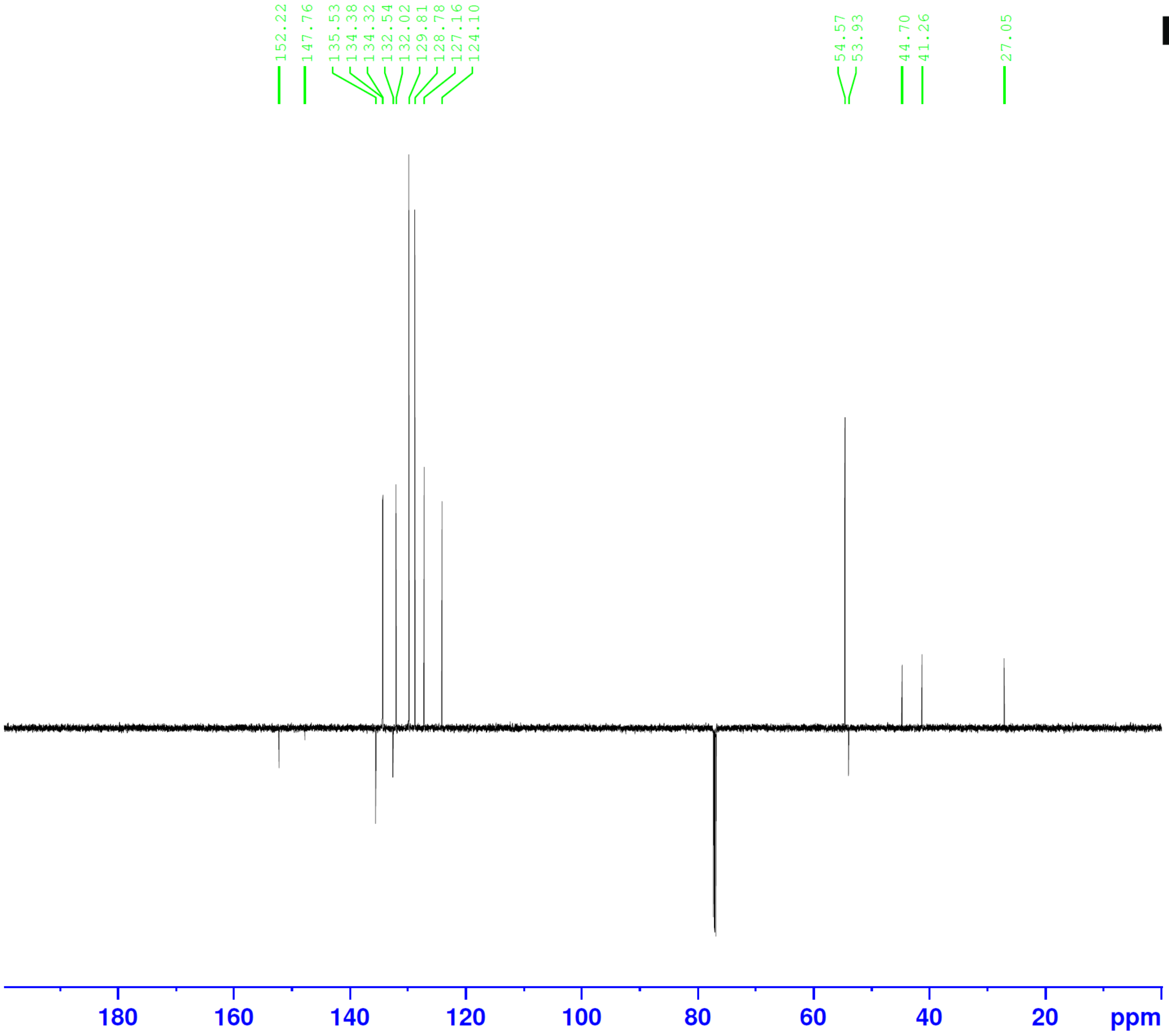

Current Data Parameters
NAME pck 3.11
EXPNO 22
PROCNO 1

F2 - Acquisition Parameters
Date_ 20180101
Time 15.54
INSTRUM spect
PROBHD 5 mm PABBO BB/
PULPROG deptqgpsp
TD 119044
SOLVENT CDCl3
NS 512
DS 4
SWH 37500.000 Hz
FIDRES 0.315010 Hz
AQ 1.5872533 sec
RG 186.92
DW 13.333 usec
DE 6.87 usec
TE 298.1 K
CNST2 145.0000000
CNST12 1.0000000
D1 1.00000000 sec
D2 0.00344828 sec
D12 0.00002000 sec
D16 0.00020000 sec
TD0 1

======== CHANNEL f1 ========
SFO1 150.9194058 MHz
NUC1 13C
P1 11.80 usec
P13 2000.00 usec
PLW0 0 W
PLW1 85.00000000 W
SPNAM[5] Crp60comp.4
SPOAL5 0.500
SPOFFS5 0 Hz
SPW5 18.08300018 W

======== CHANNEL f2 ========
SFO2 600.1324005 MHz
NUC2 1H
CPDPRG[2 waltz64
P0 10.20 usec
P3 10.20 usec
P4 20.40 usec
PCPD2 80.00 usec
PLW2 27.00000000 W
PLW12 0.43891999 W

====== GRADIENT CHANNEL =====
GPNAM[1] SINE.100
GPNAM[2] SINE.100
GPNAM[3] SINE.100
GPZ1 0 %
GPZ2 0 %
GPZ3 0 %
P16 1000.00 usec

F2 - Processing parameters
SI 131072
SF 150.9028085 MHz
WDW EM
SSB 0
LB 1.00 Hz
GB 0
PC 1.40

S11

^{1}H NMR

600 MHz

$CDCl_3$

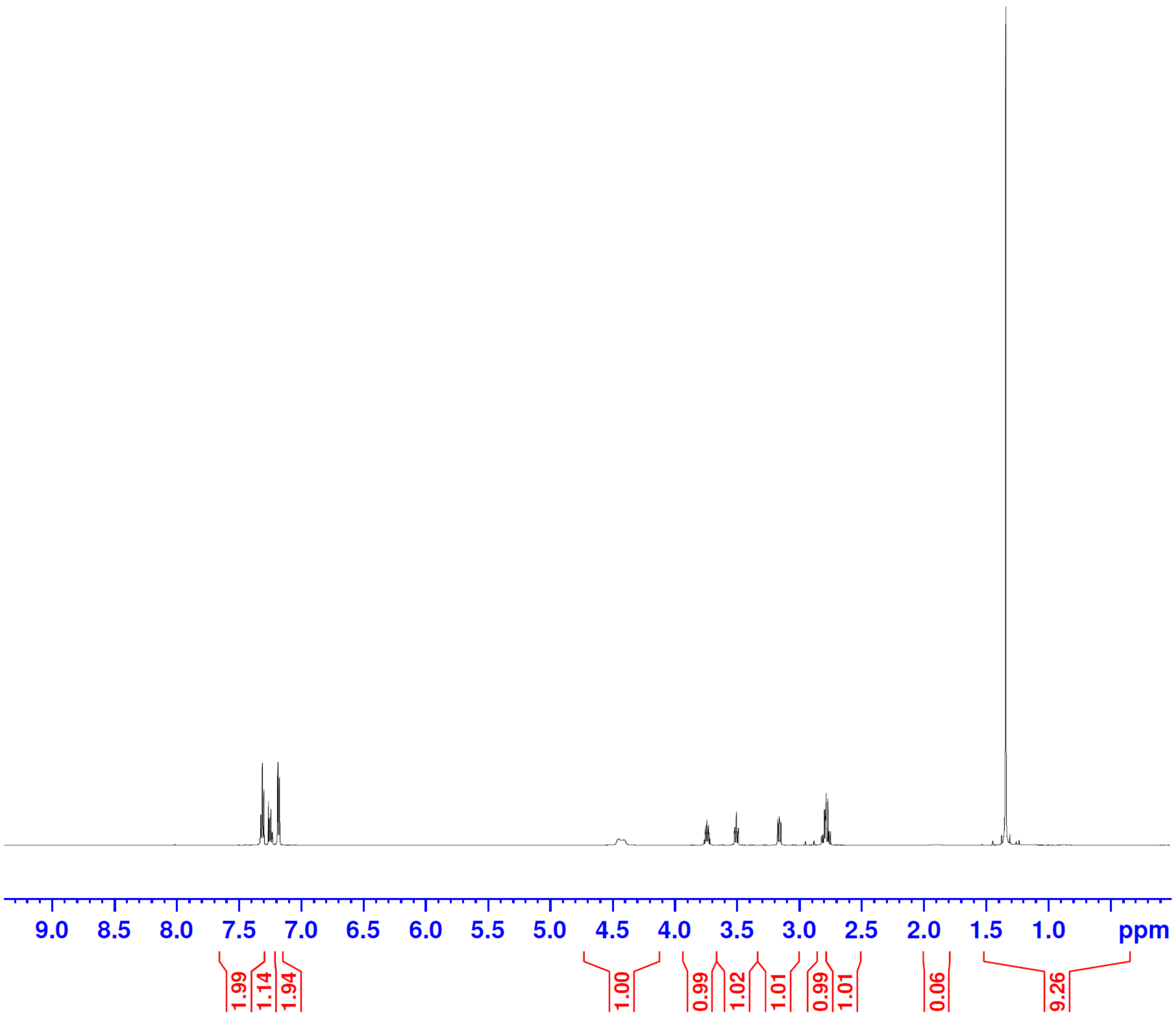


```
Current Data Parameters
NAME                pck 3.13
EXPNO                     40
PROCNO                     1

F2 - Acquisition Parameters
Date_               20180101
Time                   16.38
INSTRUM                spect
PROBHD      5 mm PABBO BB/
PULPROG                 zg30
TD                    180286
SOLVENT                CDCl3
NS                        16
DS                         0
SWH             18028.846 Hz
FIDRES           0.100001 Hz
AQ              4.9999318 sec
RG                     43.25
DW                27.733 usec
DE                  7.60 usec
TE                   298.2 K
D1            0.10000000 sec
TD0                        1

======== CHANNEL f1 ========
SFO1         600.1337060 MHz
NUC1                      1H
P1                 10.00 usec
PLW1          26.60000038 W

F2 - Processing parameters
SI                    262144
SF           600.1300120 MHz
WDW                       EM
SSB       0
LB                   0.10 Hz
GB        0
PC                      1.00
```

S11

^{13}C NMR
(DEPT-Q)

151 MHz

$CDCl_3$

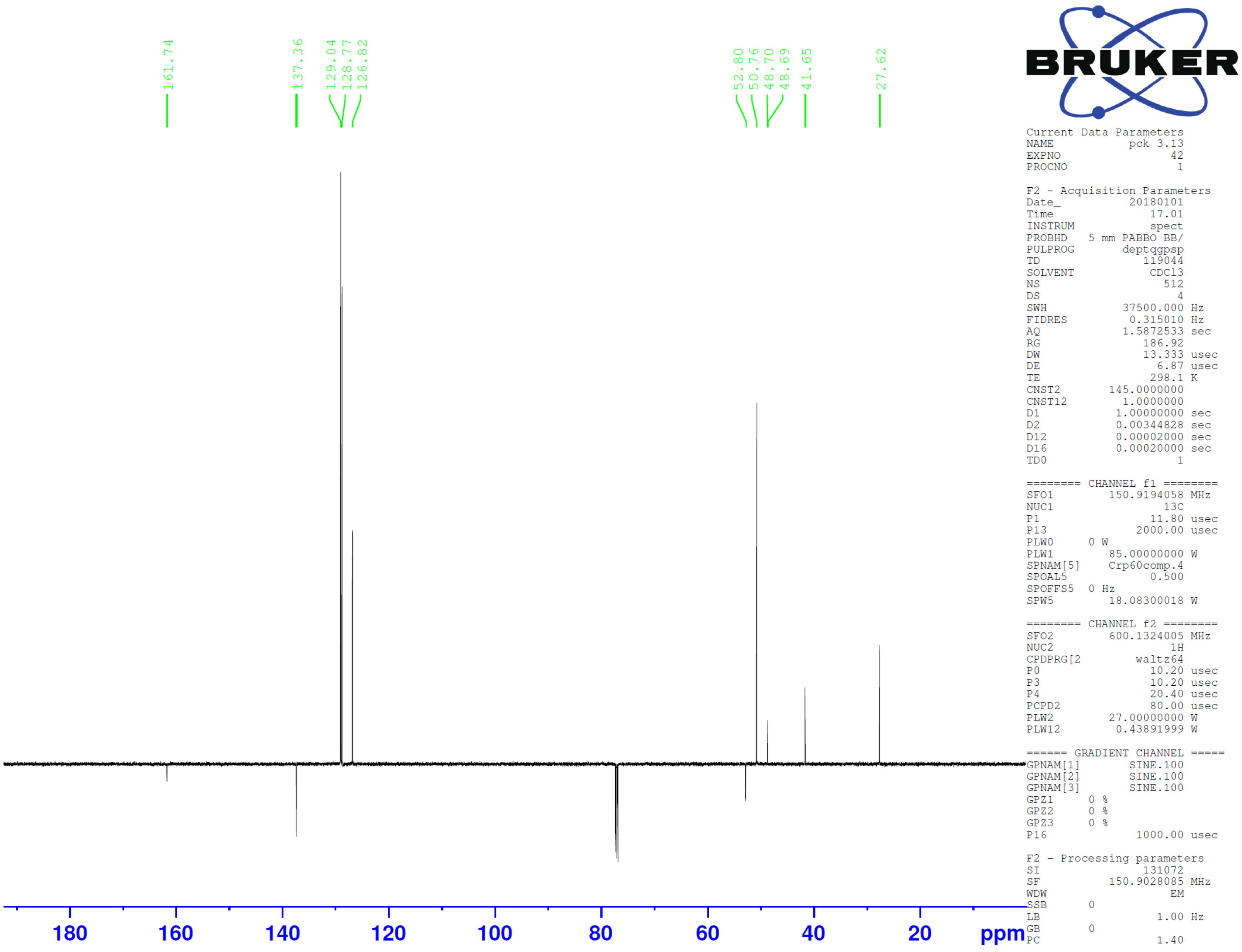

17

^{1}H NMR

600 MHz

$CDCl_3$

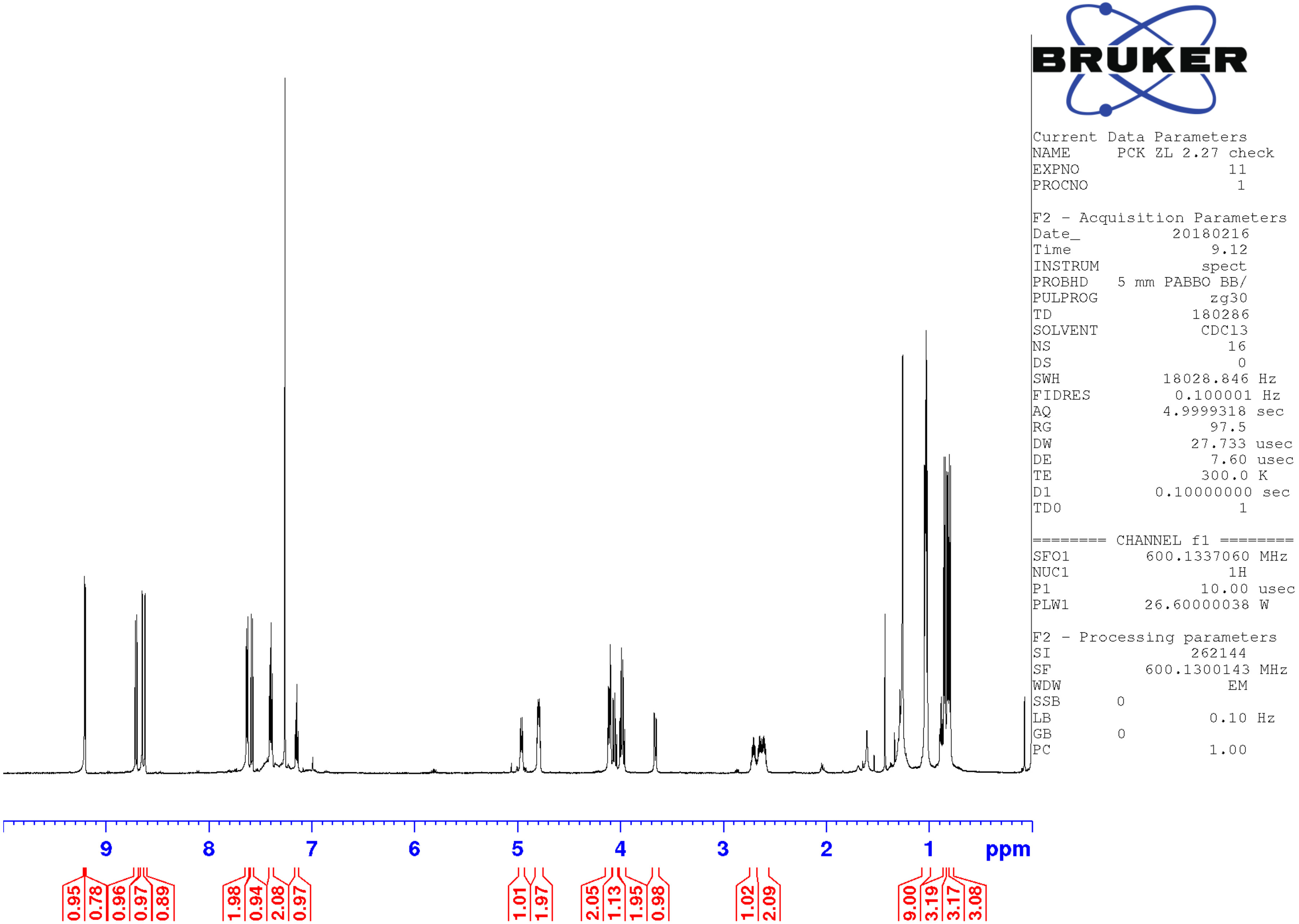

17

^{13}C NMR

151 MHz

$CDCl_3$

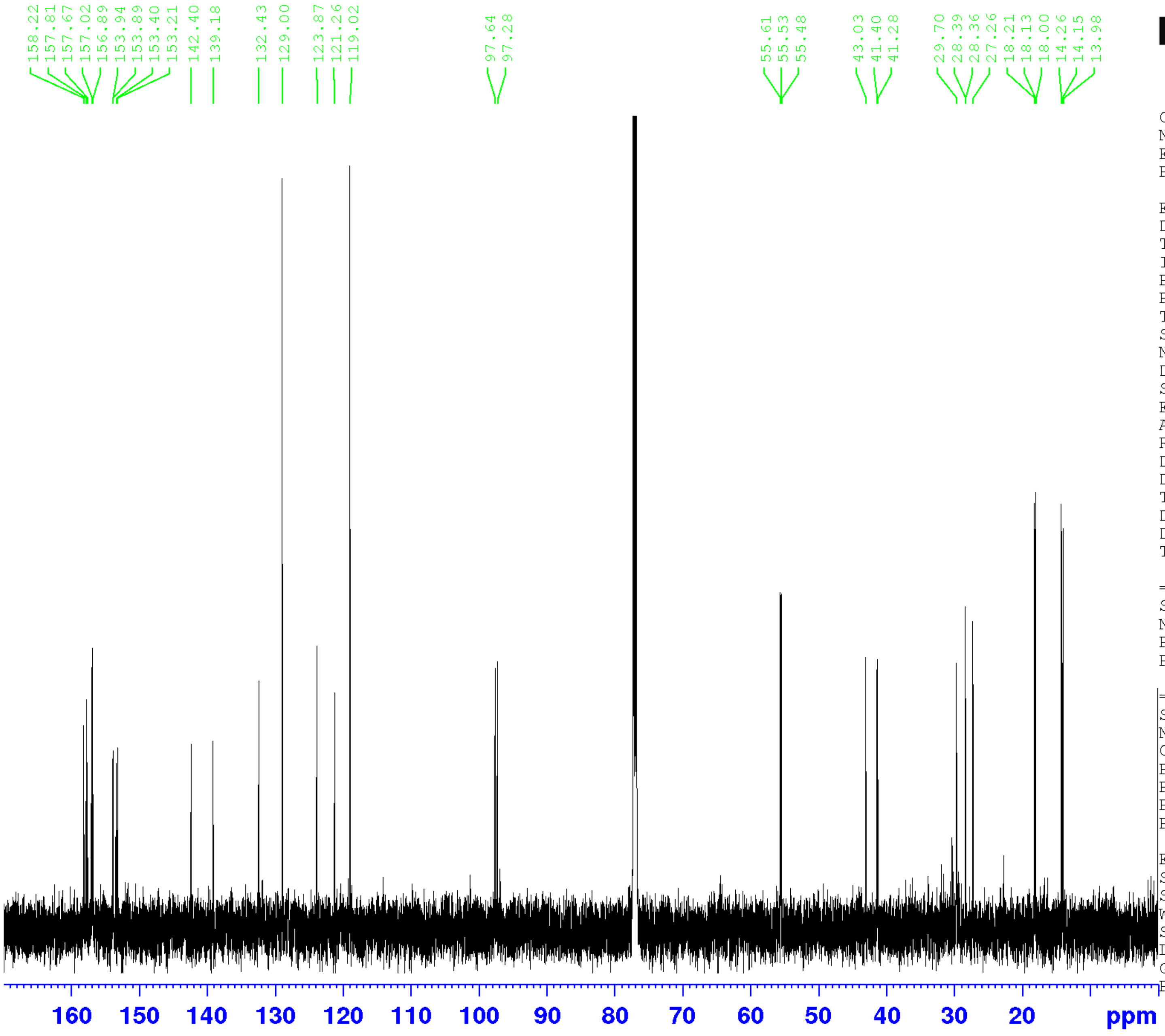


```
Current Data Parameters
NAME      PCK ZL 2.27 check
EXPNO                    12
PROCNO                    1

F2 - Acquisition Parameters
Date_              20180216
Time                  10.06
INSTRUM               spect
PROBHD     5 mm PABBO BB/
PULPROG             zgpg30
TD                   119044
SOLVENT               CDCl3
NS                     1024
DS                        4
SWH              37500.000 Hz
FIDRES            0.315010 Hz
AQ               1.5872533 sec
RG                   186.92
DW                   13.333 usec
DE                     7.73 usec
TE                    300.0 K
D1              1.00000000 sec
D11             0.03000000 sec
TD0                       1

======== CHANNEL f1 ========
SFO1          150.9194058 MHz
NUC1                    13C
P1                    11.80 usec
PLW1          85.00000000 W

======== CHANNEL f2 ========
SFO2          600.1324005 MHz
NUC2                     1H
CPDPRG[2            waltz64
PCPD2                 80.00 usec
PLW2          27.00000000 W
PLW12          0.43891999 W
PLW13          0.28090999 W

F2 - Processing parameters
SI                   131072
SF            150.9028089 MHz
WDW                      EM
SSB        0
LB                     1.00 Hz
GB         0
PC                     1.40
```

18

^{1}H NMR

600 MHz

$CDCl_3$

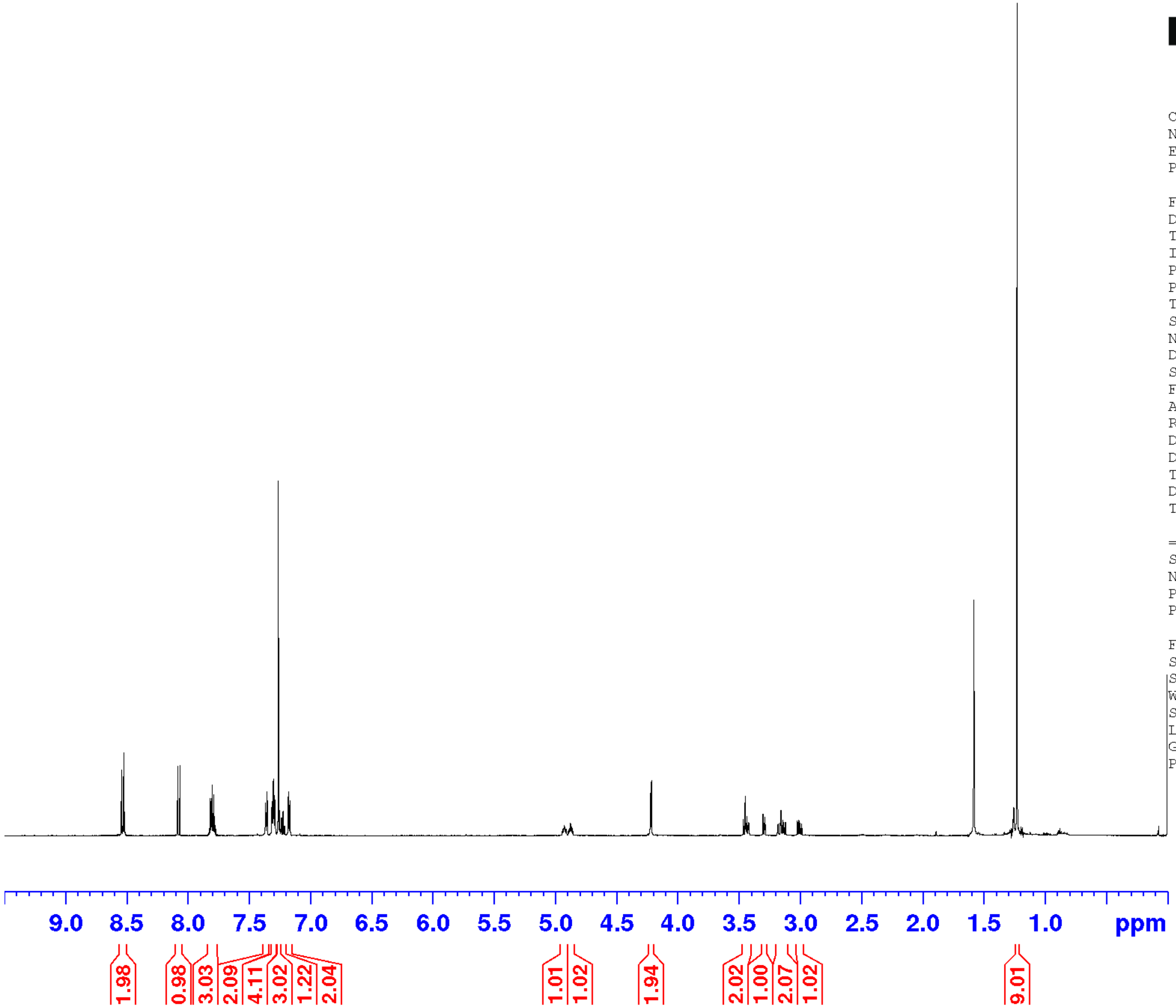


```
Current Data Parameters
NAME      ZL 2.23 FINAL 600 MHz
EXPNO                     10
PROCNO                     1

F2 - Acquisition Parameters
Date_               20170824
Time                   14.09
INSTRUM                spect
PROBHD     5 mm PABBO BB/
PULPROG                 zg30
TD                    180286
SOLVENT                CDCl3
NS                        16
DS                         0
SWH               18028.846 Hz
FIDRES             0.100001 Hz
AQ                4.9999318 sec
RG                      97.5
DW                    27.733 usec
DE                      7.60 usec
TE                     298.1 K
D1               0.10000000 sec
TD0                        1

======== CHANNEL f1 ========
SFO1            600.1337060 MHz
NUC1                      1H
P1                     10.00 usec
PLW1            26.60000038 W

F2 - Processing parameters
SI                    262144
SF              600.1300136 MHz
WDW                       EM
SSB      0
LB                      0.10 Hz
GB       0
PC                      1.00
```

18

^{13}C NMR

151 MHz

$CDCl_3$

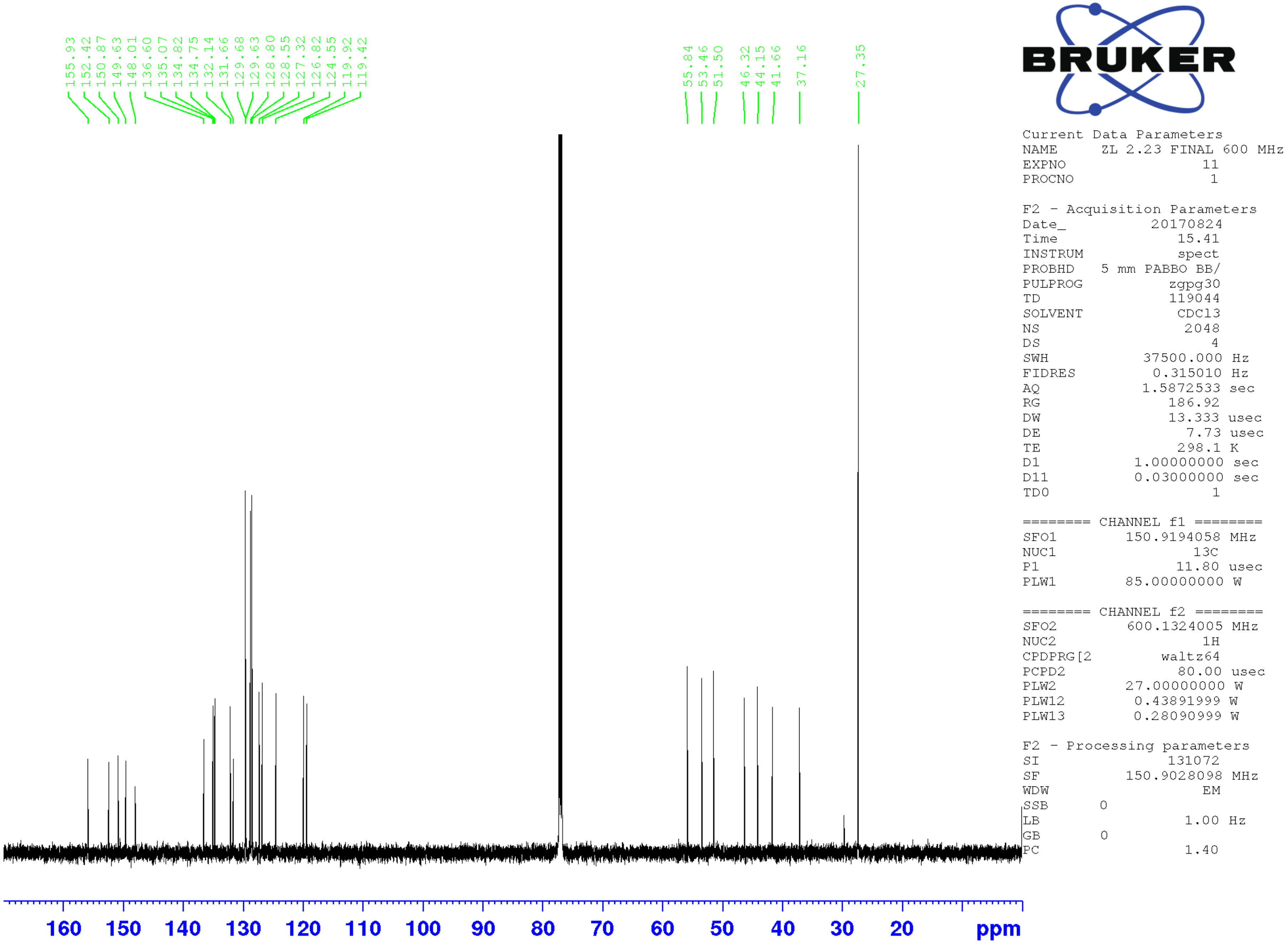

19

^{1}H NMR

600 MHz

$CDCl_3$

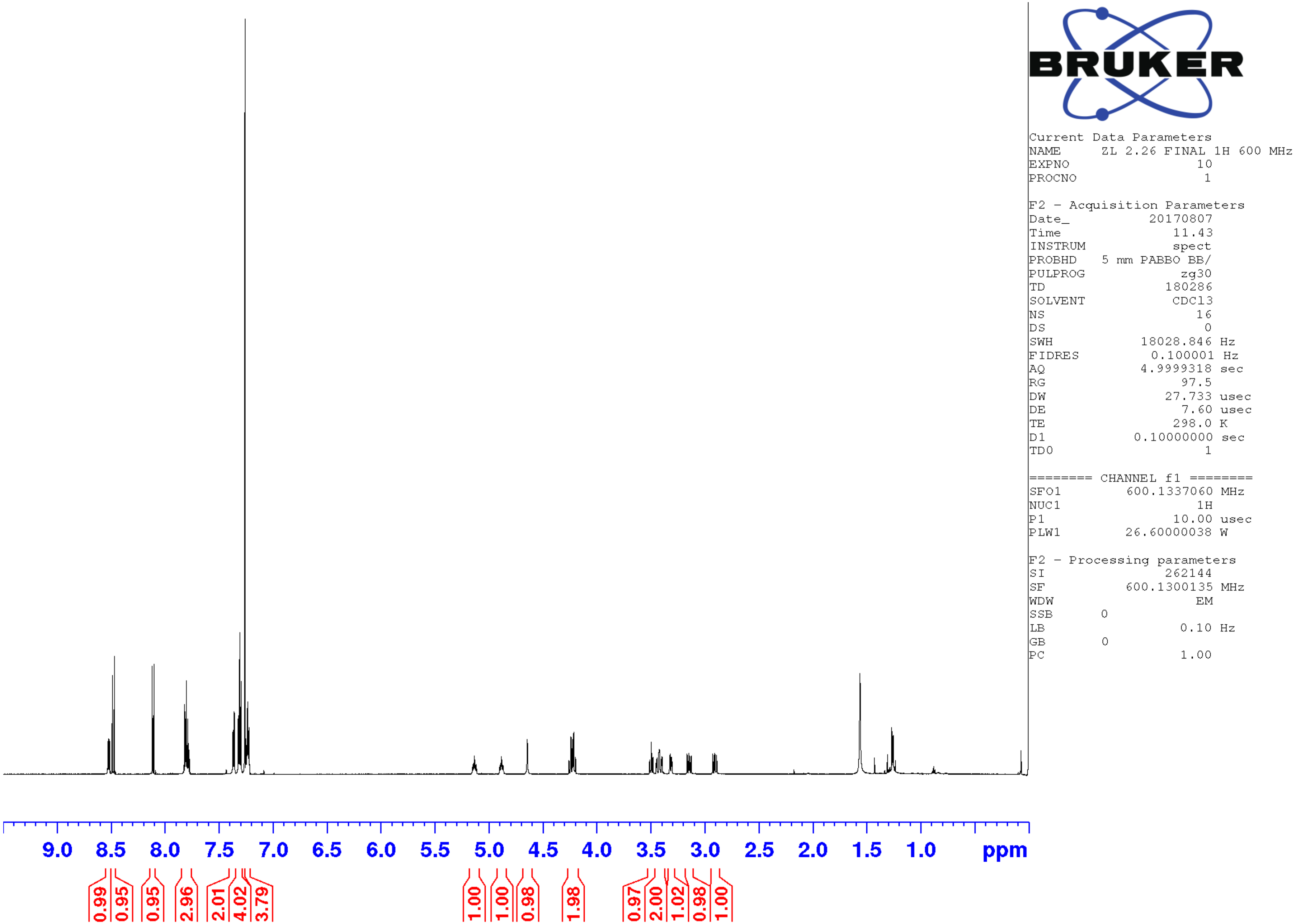

19

^{13}C NMR

151 MHz

$CDCl_3$

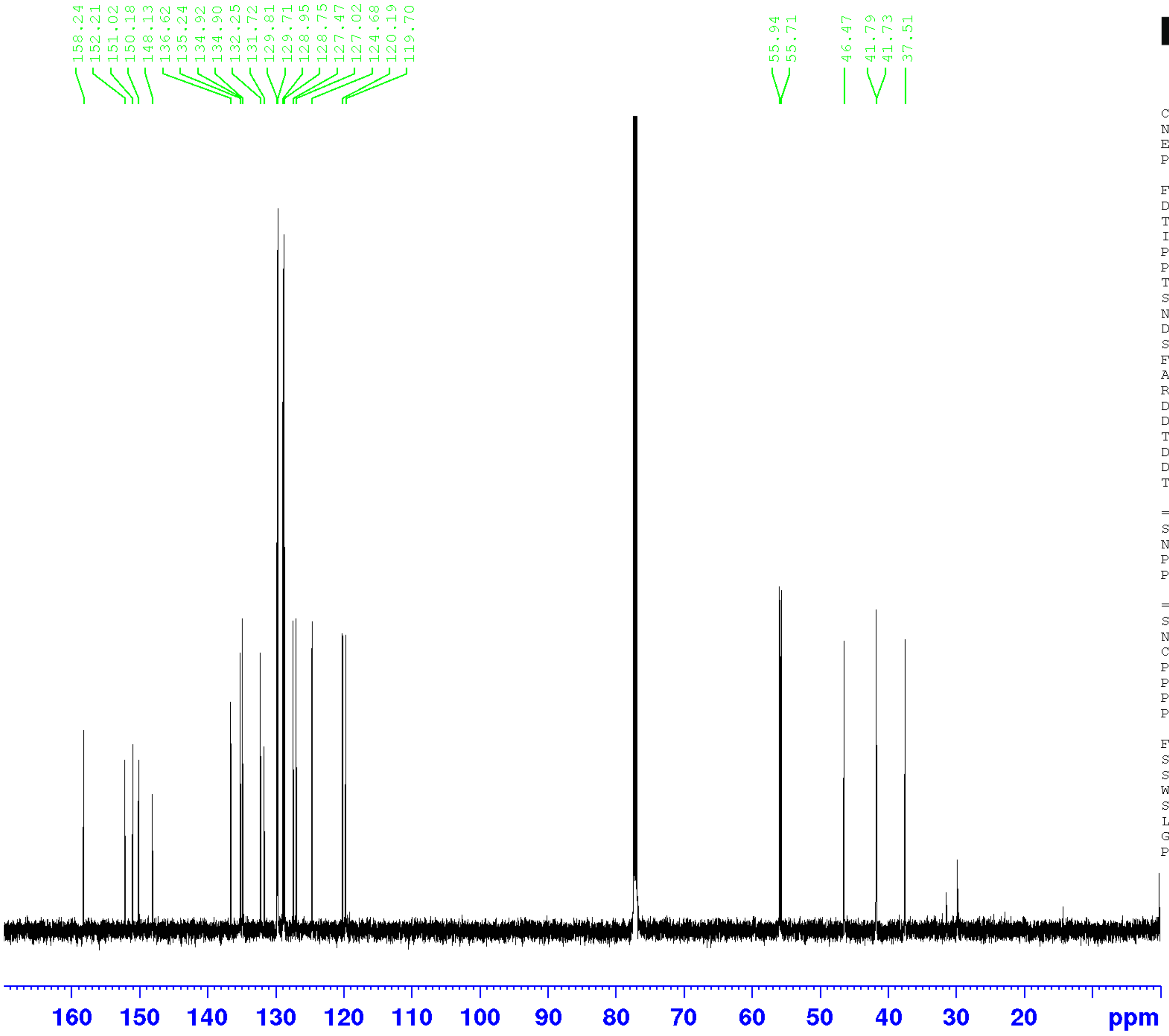


```
Current Data Parameters
NAME      ZL 2.26 FINAL 1H 600 MHz
EXPNO                    20
PROCNO                    1

F2 - Acquisition Parameters
Date_              20170807
Time                  10.15
INSTRUM               spect
PROBHD    5 mm PABBO BB/
PULPROG             zgpg30
TD                  119044
SOLVENT              CDCl3
NS                    1024
DS                       4
SWH            37500.000 Hz
FIDRES          0.315010 Hz
AQ             1.5872533 sec
RG                  186.92
DW                  13.333 usec
DE                    7.73 usec
TE                   298.0 K
D1            1.00000000 sec
D11           0.03000000 sec
TD0                      1

======== CHANNEL f1 ========
SFO1         150.9194058 MHz
NUC1                   13C
P1                   11.80 usec
PLW1          85.00000000 W

======== CHANNEL f2 ========
SFO2         600.1324005 MHz
NUC2                    1H
CPDPRG[2           waltz64
PCPD2                80.00 usec
PLW2          27.00000000 W
PLW12          0.43891999 W
PLW13          0.28090999 W

F2 - Processing parameters
SI                  131072
SF           150.9027911 MHz
WDW                     EM
SSB       0
LB                    1.00 Hz
GB        0
PC                    1.40
```

20

^{1}H NMR

600 MHz

$CDCl_3$

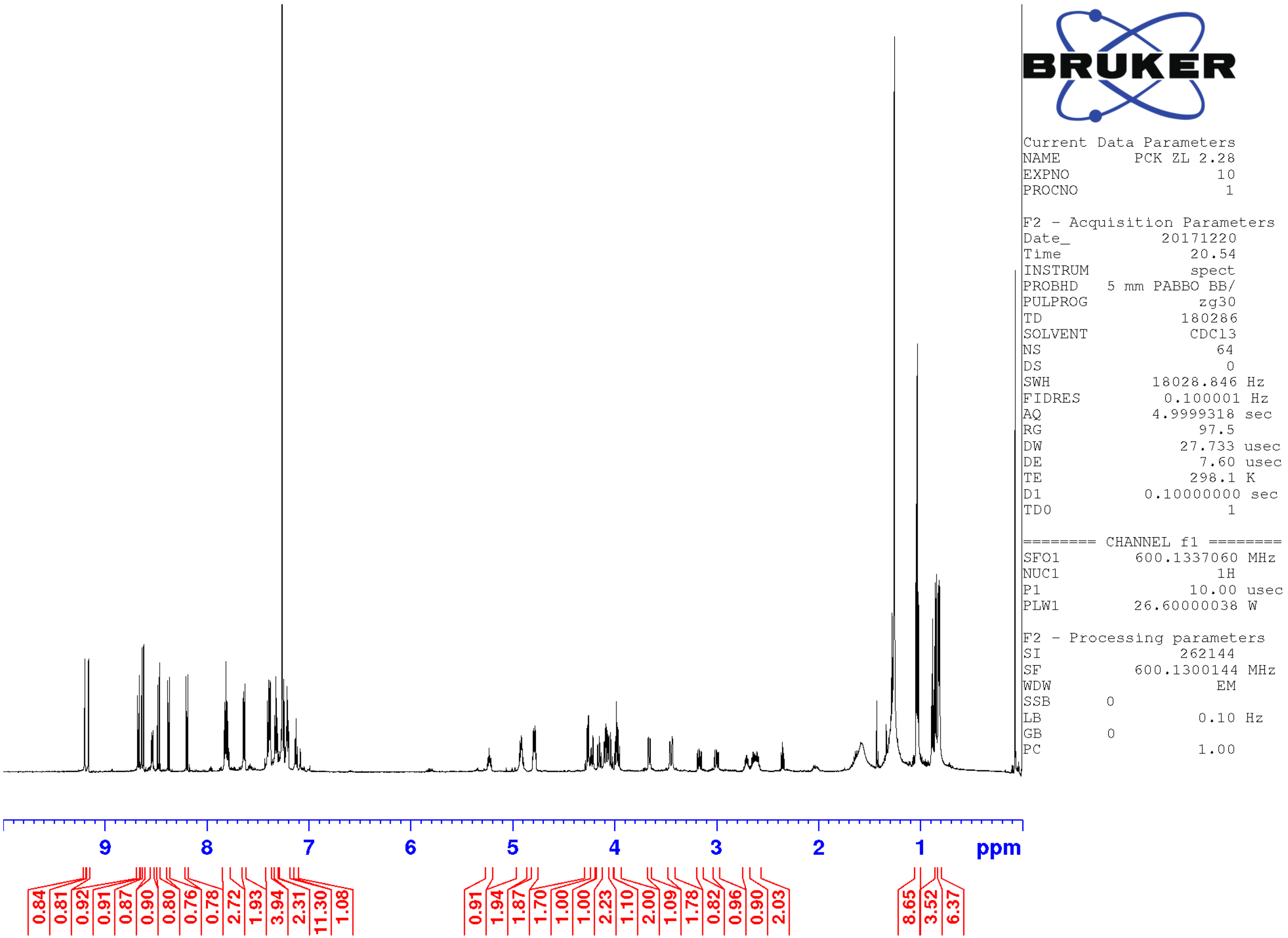

20

^{13}C NMR

151 MHz

$CDCl_3$

*Peak at 29.71 ppm due to H grease

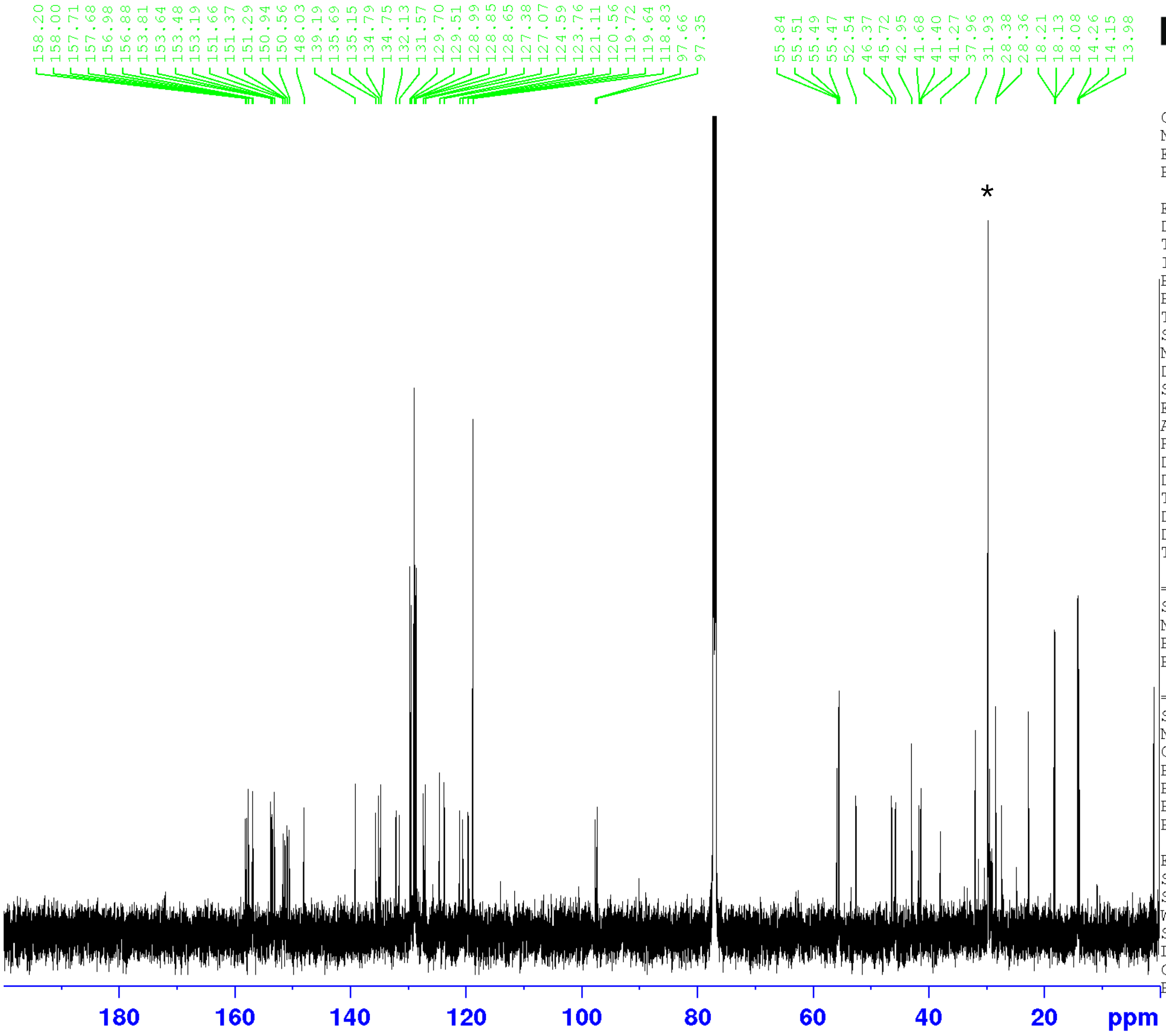


```
Current Data Parameters
NAME            PCK ZL 2.28
EXPNO                    19
PROCNO                    1

F2 - Acquisition Parameters
Date_              20171221
Time                  23.10
INSTRUM               spect
PROBHD     5 mm PABBO BB/
PULPROG             zgpg30
TD                   119044
SOLVENT              CDCl3
NS                     8192
DS                        4
SWH            37500.000 Hz
FIDRES          0.315010 Hz
AQ             1.5872533 sec
RG                   186.92
DW               13.333 usec
DE                 7.73 usec
TE                  298.1 K
D1           1.00000000 sec
D11          0.03000000 sec
TD0                       1

======== CHANNEL f1 ========
SFO1        150.9194058 MHz
NUC1                    13C
P1                11.80 usec
PLW1          85.00000000 W

======== CHANNEL f2 ========
SFO2        600.1324005 MHz
NUC2                     1H
CPDPRG[2            waltz64
PCPD2             80.00 usec
PLW2          27.00000000 W
PLW12          0.43891999 W
PLW13          0.28090999 W

F2 - Processing parameters
SI                   131072
SF          150.9028095 MHz
WDW                      EM
SSB       0
LB                  1.00 Hz
GB        0
PC                     1.40
```

21

^1H NMR

600 MHz

$CDCl_3$

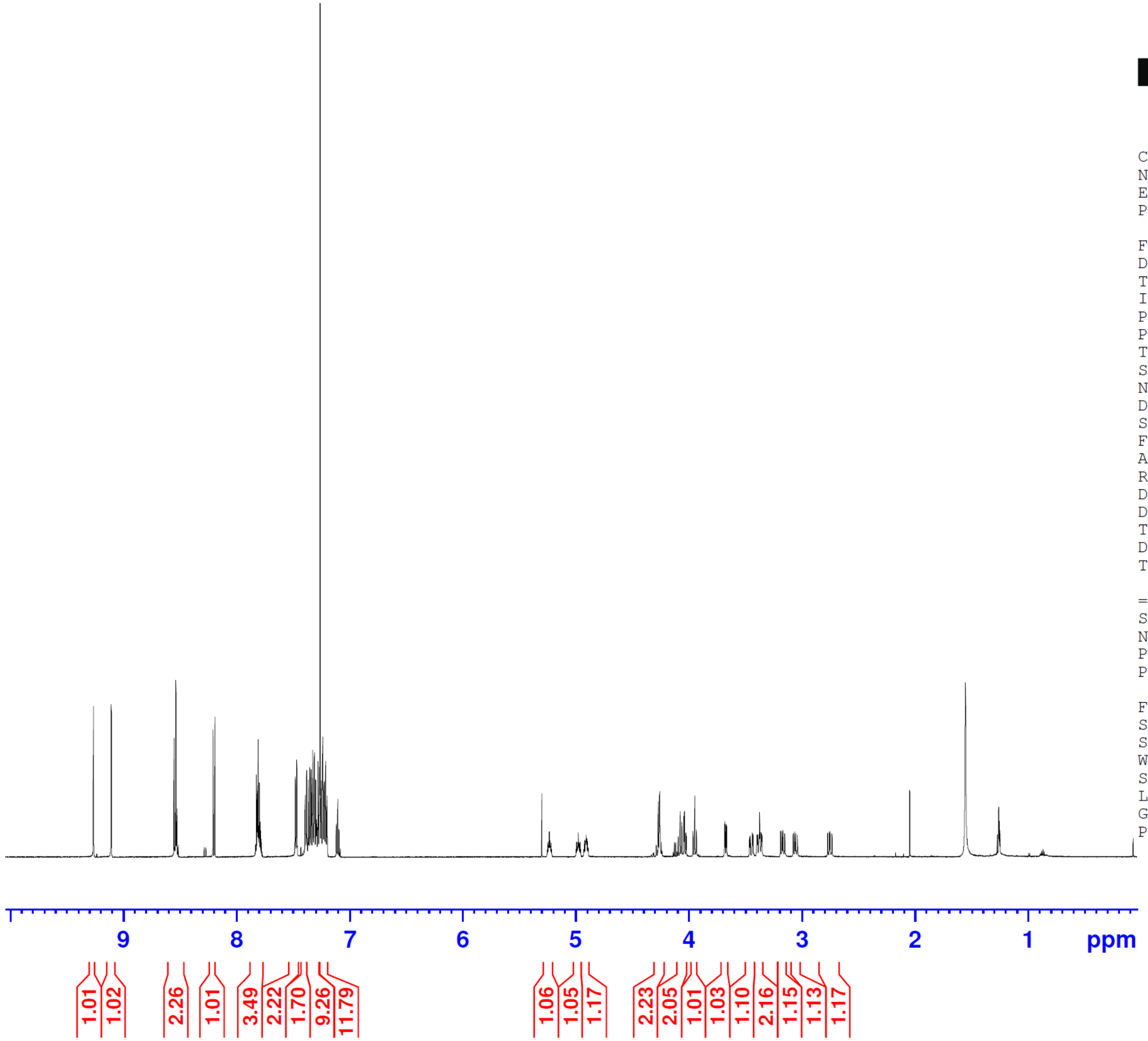

BRUKER

```
Current Data Parameters
NAME          pck 2.04 600
EXPNO                   10
PROCNO                   1

F2 - Acquisition Parameters
Date_             20170505
Time                 23.33
INSTRUM              spect
PROBHD     5 mm PABBO BB/
PULPROG               zg30
TD                  180286
SOLVENT              CDCl3
NS                      16
DS                       0
SWH              18028.846 Hz
FIDRES            0.100001 Hz
AQ               4.9999318 sec
RG                    97.5
DW                  27.733 usec
DE                    7.60 usec
TE                   297.2 K
D1              0.10000000 sec
TD0                      1

======== CHANNEL f1 ========
SFO1           600.1337060 MHz
NUC1                    1H
P1                   10.00 usec
PLW1           26.60000038 W

F2 - Processing parameters
SI                  262144
SF             600.1300139 MHz
WDW                     EM
SSB      0
LB                    0.10 Hz
GB       0
PC                    1.00
```

21

^{13}C NMR

151 MHz

$CDCl_3$

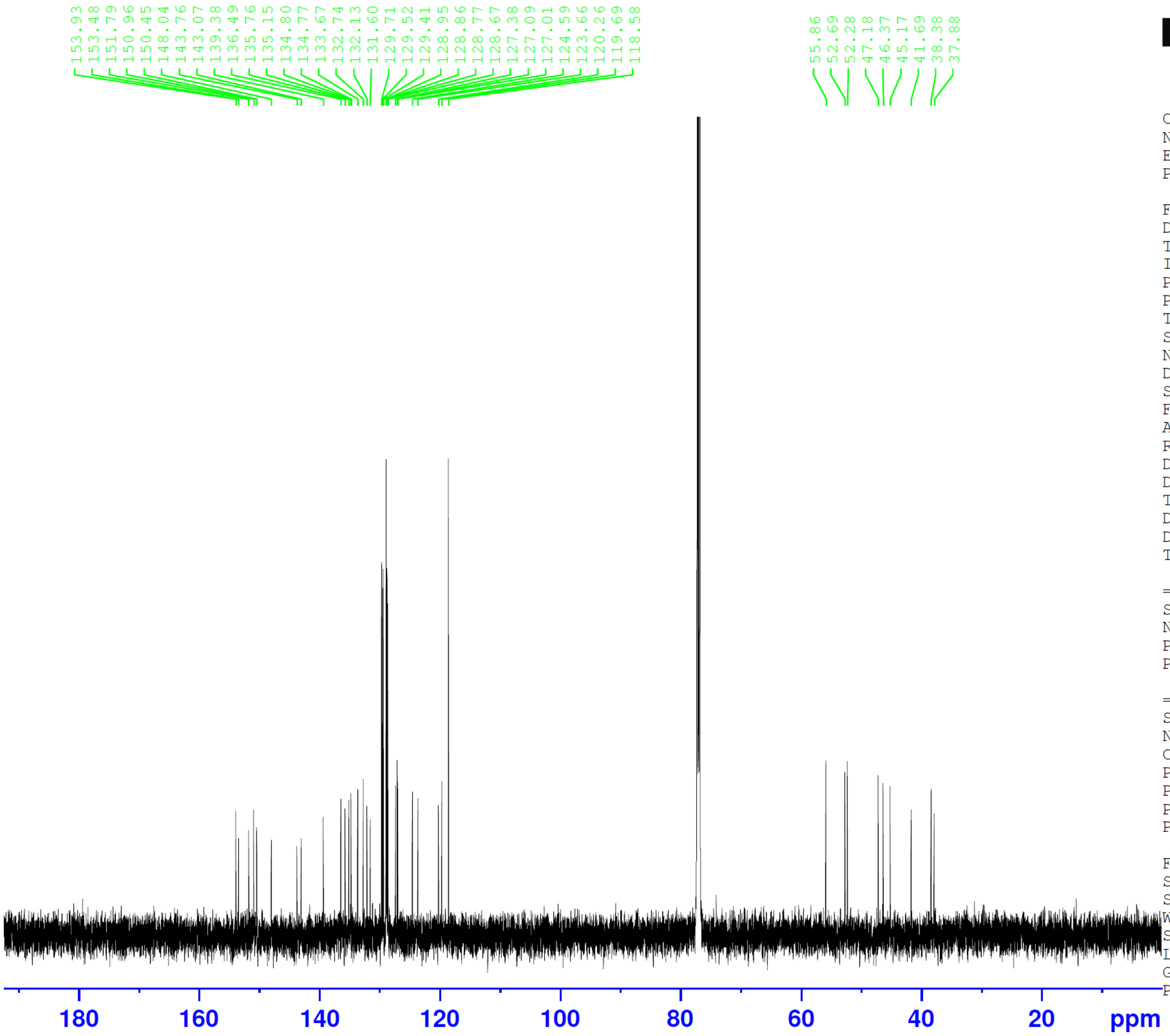

BRUKER

Current Data Parameters
NAME pck 2.04 600
EXPNO 11
PROCNO 1

F2 - Acquisition Parameters
Date_ 20170506
Time 2.35
INSTRUM spect
PROBHD 5 mm PABBO BB/
PULPROG zgpg30
TD 119044
SOLVENT CDCl3
NS 4096
DS 4
SWH 37500.000 Hz
FIDRES 0.315010 Hz
AQ 1.5872533 sec
RG 186.92
DW 13.333 usec
DE 7.73 usec
TE 298.1 K
D1 1.00000000 sec
D11 0.03000000 sec
TD0 1

======== CHANNEL f1 ========
SFO1 150.9194058 MHz
NUC1 13C
P1 11.80 usec
PLW1 85.00000000 W

======== CHANNEL f2 ========
SFO2 600.1324005 MHz
NUC2 1H
CPDPRG[2 waltz64
PCPD2 80.00 usec
PLW2 27.00000000 W
PLW12 0.43891999 W
PLW13 0.28090999 W

F2 - Processing parameters
SI 131072
SF 150.9028085 MHz
WDW EM
SSB 0
LB 1.00 Hz
GB 0
PC 1.40

22

^1H NMR

600 MHz

$CDCl_3$

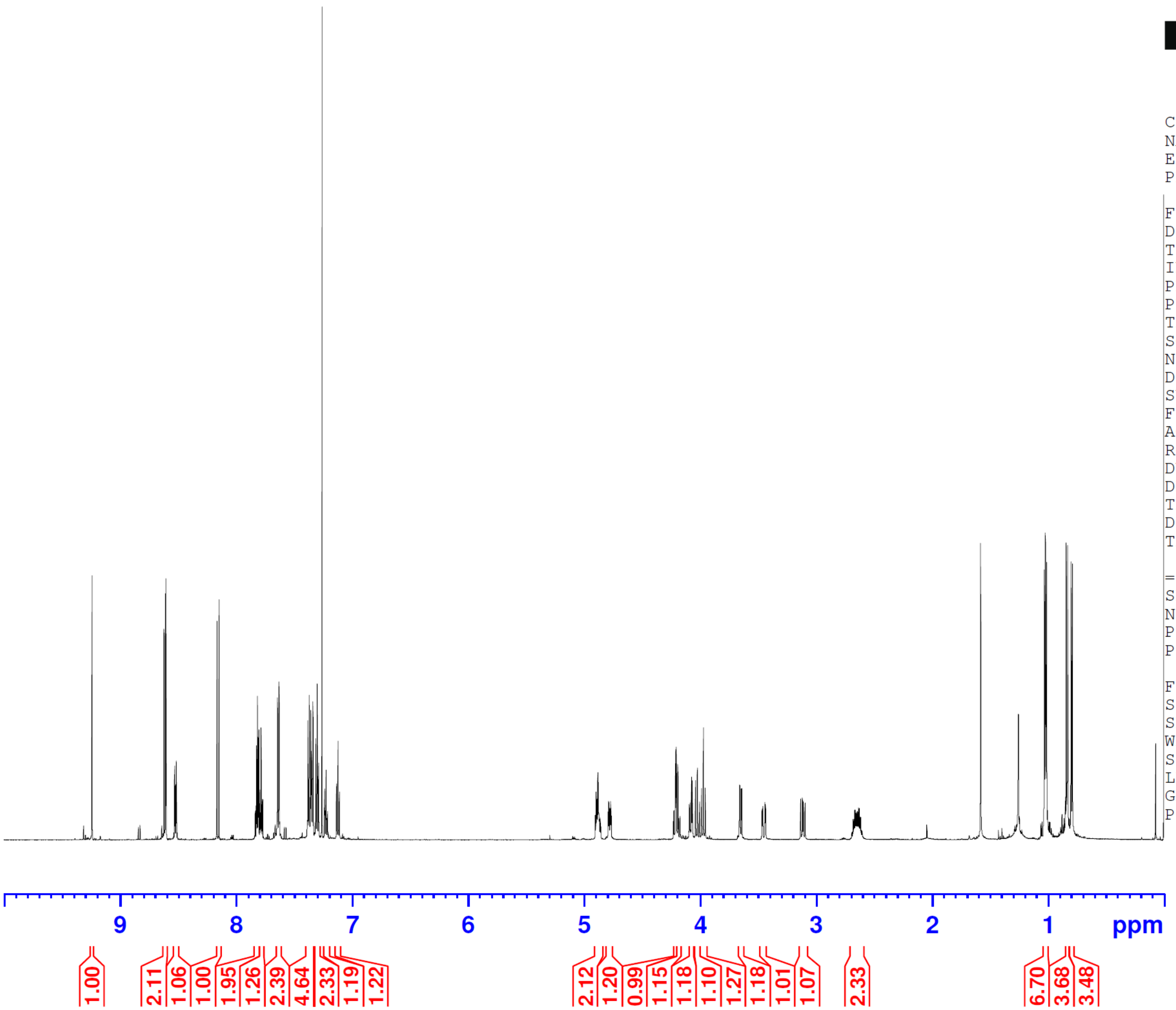

BRUKER

```
Current Data Parameters
NAME       pck 2.85 f19-25
EXPNO                   10
PROCNO                   1

F2 - Acquisition Parameters
Date_             20171020
Time                 10.45
INSTRUM              spect
PROBHD     5 mm PABBO BB/
PULPROG               zg30
TD                  180286
SOLVENT              CDCl3
NS                      16
DS                       0
SWH              18028.846 Hz
FIDRES            0.100001 Hz
AQ               4.9999318 sec
RG                    97.5
DW                  27.733 usec
DE                    7.60 usec
TE                   298.1 K
D1              0.10000000 sec
TD0                      1

======== CHANNEL f1 ========
SFO1           600.1337060 MHz
NUC1                    1H
P1                   10.00 usec
PLW1           26.60000038 W

F2 - Processing parameters
SI                  262144
SF             600.1300137 MHz
WDW                     EM
SSB      0
LB                    0.10 Hz
GB       0
PC                    1.00
```

22

^{13}C NMR

151 MHz

$CDCl_3$

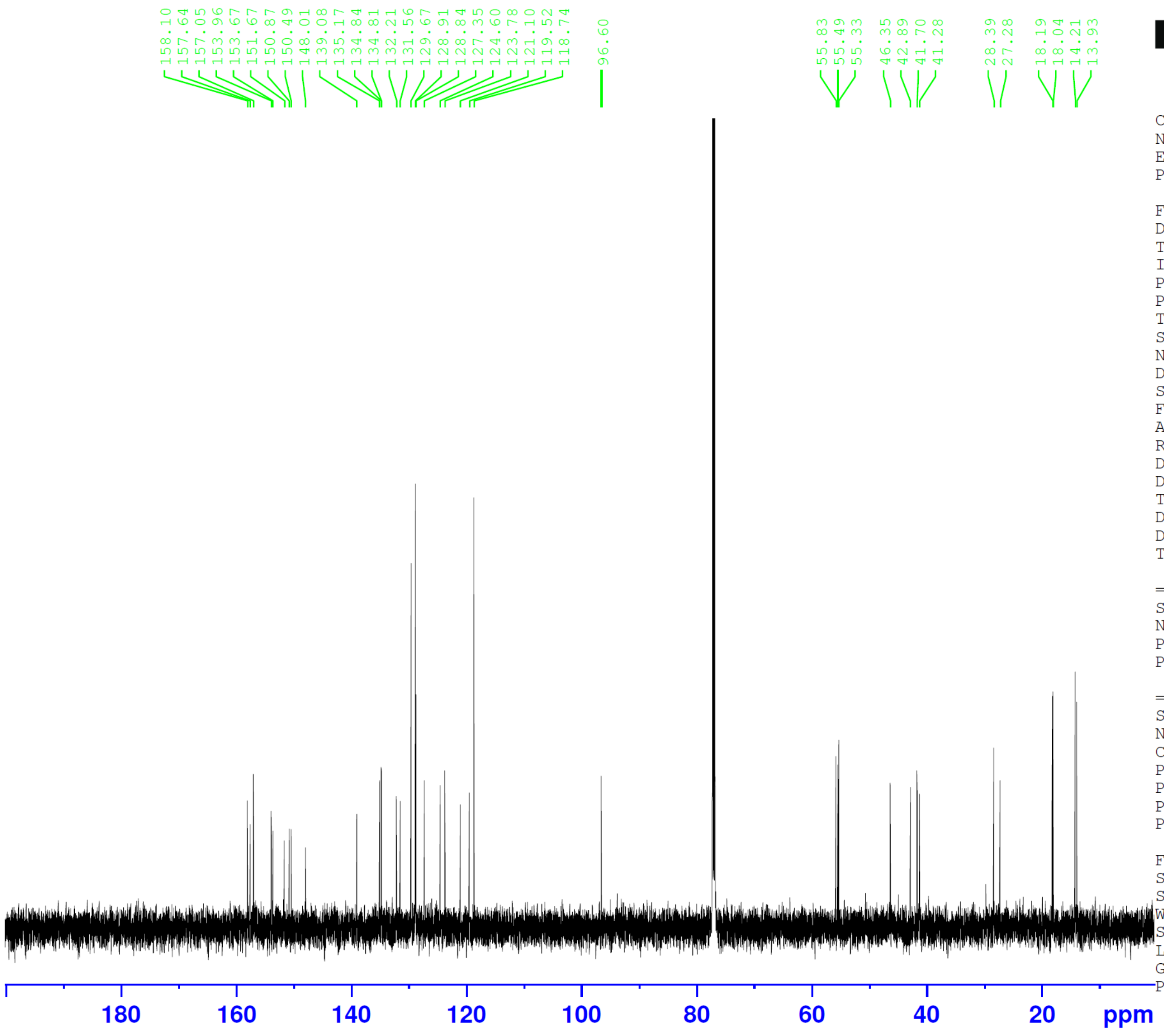


```
Current Data Parameters
NAME      pck 2.85 f19-25
EXPNO                  11
PROCNO                  1

F2 - Acquisition Parameters
Date_            20171020
Time                10.57
INSTRUM             spect
PROBHD    5 mm PABBO BB/
PULPROG           zgpg30
TD                119044
SOLVENT            CDCl3
NS                    512
DS                      4
SWH             37500.000 Hz
FIDRES           0.315010 Hz
AQ              1.5872533 sec
RG                 186.92
DW                 13.333 usec
DE                   7.73 usec
TE                  298.1 K
D1             1.00000000 sec
D11            0.03000000 sec
TD0                     1

======== CHANNEL f1 ========
SFO1          150.9194058 MHz
NUC1                  13C
P1                  11.80 usec
PLW1          85.00000000 W

======== CHANNEL f2 ========
SFO2          600.1324005 MHz
NUC2                   1H
CPDPRG[2          waltz64
PCPD2               80.00 usec
PLW2          27.00000000 W
PLW12          0.43891999 W
PLW13          0.28090999 W

F2 - Processing parameters
SI                 131072
SF            150.9028085 MHz
WDW                    EM
SSB      0
LB                   1.00 Hz
GB       0
PC                   1.40
```
